# Use of pyridazinediones for tuneable and reversible covalent cysteine modification applied to peptides, proteins and hydrogels†

**DOI:** 10.1039/d3sc04976k

**Published:** 2023-11-01

**Authors:** Léa N. C. Rochet, Calise Bahou, Jonathan P. Wojciechowski, Ilias Koutsopetras, Phyllida Britton, Richard J. Spears, Ioanna A. Thanasi, Baihao Shao, Lisha Zhong, Dejan-Krešimir Bučar, Abil E. Aliev, Michael J. Porter, Molly M. Stevens, James R. Baker, Vijay Chudasama

**Affiliations:** a Department of Chemistry, University College London 20 Gordon Street London WC1H 0AJ UK v.chudasama@ucl.ac.uk j.r.baker@ucl.ac.uk; b Department of Materials, Department of Bioengineering, Institute of Biomedical Engineering, Imperial College London London SW7 2AZ UK; c Bio-Functional Chemistry (UMR 7199), Institut du Médicament de Strasbourg, University of Strasbourg 74 Route du Rhin 67400 Illkirch-Graffenstaden France

## Abstract

Reversible cysteine modification has been found to be a useful tool for a plethora of applications such as selective enzymatic inhibition, activity-based protein profiling and/or cargo release from a protein or a material. However, only a limited number of reagents display reliable dynamic/reversible thiol modification and, in most cases, many of these reagents suffer from issues of stability, a lack of modularity and/or poor rate tunability. In this work, we demonstrate the potential of pyridazinediones as novel reversible and tuneable covalent cysteine modifiers. We show that the electrophilicity of pyridazinediones correlates to the rates of the Michael addition and retro-Michael deconjugation reactions, demonstrating that pyridazinediones provide an enticing platform for readily tuneable and reversible thiol addition/release. We explore the regioselectivity of the novel reaction and unveil the reason for the fundamental increased reactivity of aryl bearing pyridazinediones by using DFT calculations and corroborating findings with SCXRD. We also applied this fundamental discovery to making more rapid disulfide rebridging agents in related work. We finally provide the groundwork for potential applications in various areas with exemplification using readily functionalised “clickable” pyridazinediones on clinically relevant cysteine and disulfide conjugated proteins, as well as on a hydrogel material.

## Abstract 2

Substituted pyridazinediones (PDs) enable reversible and tuneable cysteine modification with exemplification on peptides, proteins and hydrogels.

## Graphical abstract

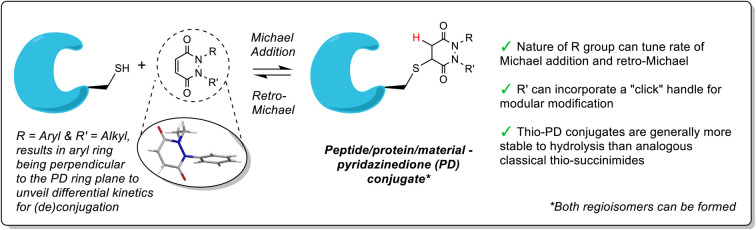

## Introduction

**Fig. 1:**
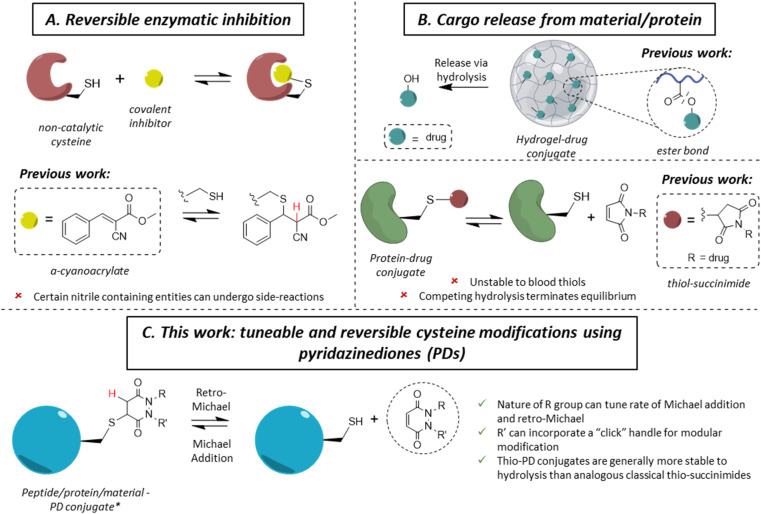
Selected applications of reversible cysteine modification for (A). Reversible enzymatic inhibition and (B) cargo release from a peptide/protein/material; the current technologies developed for these purposes and their potential drawbacks. (C) Displays the work presented herein, using functionalised pyridazinediones (PD) as reversible thiol modification reagents. * Only a single regioisomer is drawn for simplicity, but both regioisomers may form.

The ability to site-specifically modify a protein to append various moieties is a key component of the chemical biologist's toolkit, and it has led to many applications in therapy, imaging and diagnostics.^1–3^ With a low natural abundance (<2%),^4^ and a unique reactivity due to the high nucleophilicity of the thiol side-chain, cysteine is a valuable target amino acid for site-specific protein modification.^5–7^ As a large number of applications of protein conjugates are *in vivo*, significant efforts have been invested in developing methods for the irreversible (*i.e.*, blood stable) site-specific modification of cysteine(s) on various proteins to enable diverse applications in medicine, such as antibody–drug conjugates (ADCs), imaging agents and radioimmunoconjugates.^8–12^ More recently, reversible cysteine modification has been found to be an invaluable tool in a variety of fields, such as selective enzymatic inhibition or the controlled release of cargo from a material or a protein.^13–15^ In the case of the former, non-catalytic cysteines are commonly found in the active sites of enzymes (*e.g.*, in most of the 500 human kinases),^16^ making them an attractive drug target in the field of cancer-associated protein kinases.^17–19^ Historically, one of the main strategies of kinase inhibition relied on the formation of an irreversible covalent bond between a cysteine and an electrophilic Michael acceptor warhead (*e.g.*, acrylamide).^20,21^ However, as these molecules irreversibly bound blood thiols (*e.g.*, glutathione and human serum albumin (HSA)), it led to off-target toxicity.^22,23^ By inserting a nitrile group at the α-carbon position of an acrylamide, Serafimova *et al.* developed a reversible electrophilic inhibitor able to efficiently and selectively inhibit a kinase (Fig. 1A).^14^ We also note that cyanoacrylate-based thiol-reactive moieties have been used for the modification of proteins with azobenzene crosslinkers as photoswitches.^24^ Barring the work on cyanoacrylates,^25^ only a few other reagents have been shown to display selective reversible cysteine modification.^26^ As an example, dihaloacetamides have been found to react with cysteines and, upon hydrolysis at neutral pH, the original cysteine may be released from the cysteine–haloacetamide conjugate, but these reagents are limited by being protein microenvironment specific and the dihaloacetamide is not re-formed post-hydrolysis.^27^ Another area where reversible covalent modification has found application is the release of a drug from a surface/material to a local area or *via* a carrier protein (Fig. 1B). With the example of hydrogel–drug conjugates, it has been shown that covalent approaches to attach and release a drug are preferred among different strategies, as they provide better modularity and time control of release.^28^ Examples of drug release in this context depend mostly on ester bond hydrolysis, disulfide bond exchange and/or a β-elimination mechanism.^29–32^ Similar approaches have been applied for the release of a drug from a peptide and/or protein.^33,34^

Whilst the use of a vinylic cyano group on an acrylamide has unearthed many seminal advances in the field,^14,25^ certain nitrile containing compounds have been shown to react in an irreversible manner with N-terminal cysteines and/or undergo cysteine to lysine transfer,^35,36^ acting as irreversible traps and/or potentially leading to unwanted side-reactions.^37^ Additionally, in the case of cargo release from a material or hydrogel, most of the reported approaches rely on an external stimulus for the release of the cargo (*e.g.*, a very high concentration of thiols, an acidic environment, *etc.*), which is not always desirable. Overall, we highlight that there are only few well understood controllable and reversible cysteine modification strategies known.^26^ Beyond the above examples, perhaps the most common is the thiol-succinimide linkage, which is known to undergo a retro-Michael (RM) reaction (Fig. 1B).^38,39^ However, the rapid kinetics of maleimide-thiol conjugation makes any released maleimide highly susceptible to off-target thiol modification by other biomolecules (*e.g.*, HSA),^40^ thus limiting their use. Moreover, such thiol-succinimide linkages can undergo hydrolysis relatively easily, which is competitive against the retro-Michael pathway.^41^ Such a hydrolysis reaction also terminates (mechanistically) the RM deconjugation pathway completely and renders the modification irreversible. Therefore, there is a clear demand for novel platforms to reversibly modify cysteines in a highly selective manner, while also being able to tune the thiol reactivity profile. Moreover, by developing a more reliable and tuneable system of reversible covalent cysteine modification, new avenues of research could be enabled. For example, whilst activity-based protein profiling (ABPP) has proved to be a valuable research tool,^42–44^ it has been mainly applied using acetamide probes;^45–49^ this assay could gain from the development of novel reversible probes.^13,50,51^

Motivated by the plethora of applications that could be exploited, we previously showed, in a single example, that a pyridazinedione (PD) scaffold displayed promise as a novel reversible covalent cysteine modifier.^52^ Nonetheless, our previous work was limited by: (i) the use of a single PD bearing ethyl groups on each of the nitrogen atoms (*i.e.*, PD 1) with only a single thiol reactivity profile (*i.e.*, there was no data on thiol reactivity tunability), (ii) the absence of any functional or functionalisable PD, (iii) a lack of exemplification, and (iv) no substantive kinetics data. In this manuscript, we synthesise and appraise a new library of PDs to address these limitations. We were able to obtain kinetic data across a range of PD-thiol reactions (including on “clickable” PDs that enabled facile, modular assembly of the conjugates), unearthed PDs with varying profiles of reactivity, and applied our findings to cysteine and disulfide bearing proteins (for exemplification on different scaffolds and to show predictable and tuneable release of PDs) and on a hydrogel (to show controlled release of a cysteine containing peptide).

## Results and discussion

### Synthesis of a library of PDs

**Fig. 2:**
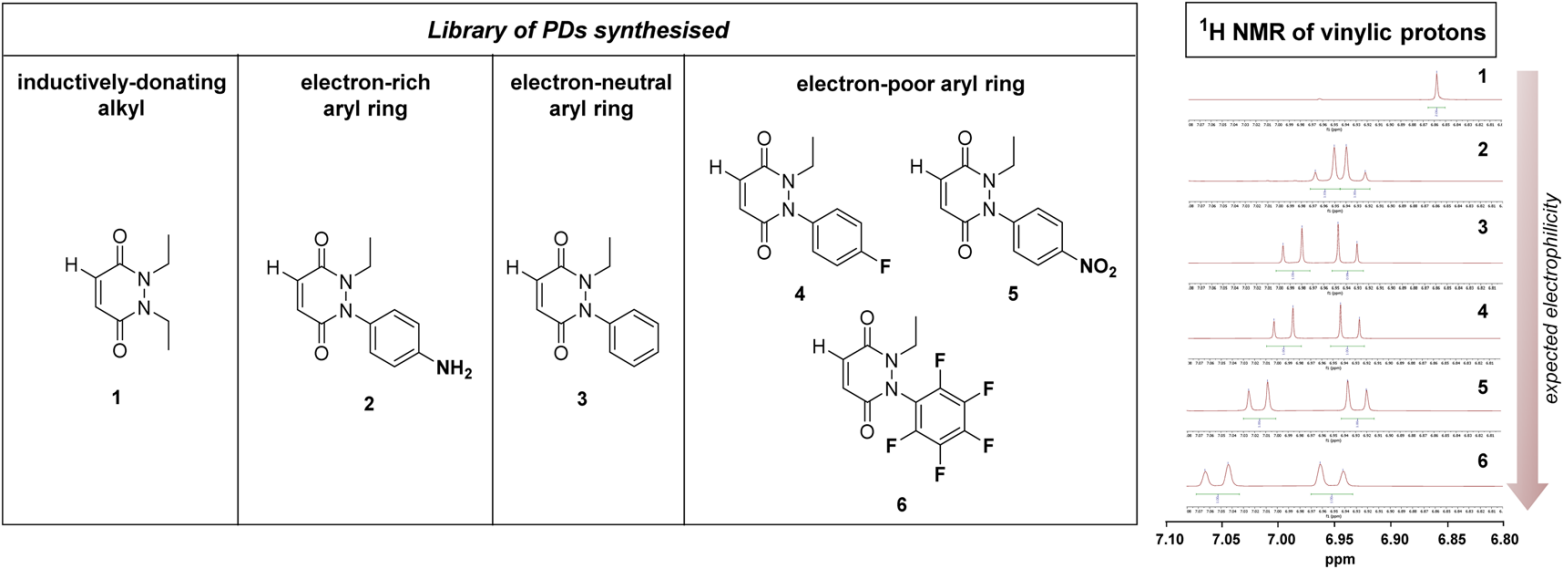
Left: library of synthesised PDs 1–6 sorted by their expected *N*-group substitution electrophilicity ranging from electron-donating group (left) to electron-neutral (middle) to electron-withdrawing (right). Right: zoomed in ^1^H NMR spectra in the vinylic region for PDs 1–6 displaying different chemical shifts that could correlate with their expected electrophilicity.

From the outset, it was hypothesised that modifying at least one of the nitrogen atoms on the PD scaffold (R group in Fig. 1C) with an aromatic π-system could have a significant effect on the PD ring core that would influence its electrophilicity, as well as potentially influencing the acidity of the α-proton in the resultant thiol–PD conjugate (highlighted in red in Fig. 1C). To appraise this theory, as well as to see if the nature of the aryl group had any effect on reactivity, we set out to synthesise various PDs bearing different aromatic groups (*e.g.*, electron-poor, electron-rich and electron-neutral) on one of the nitrogen atoms and an ethyl group on the other nitrogen (PDs 2–6, Fig. 2). To access this range of PDs displaying potentially different electronic properties, we initially exploited the use of commercially available aryl hydrazines to obtain the corresponding ethylated versions by reductive amination using acetaldehyde, followed by condensation (under acidic conditions) with maleic anhydride to yield *N*-Et, *N′*-aryl PDs 3, 4, and 6. We could directly modify the aryl ring of *N-*Et, *N′*-Ph PD 3 by nitration to yield *N-*Et, *N′*-NO_2_Ph PD 5. Reduction of *N-*Et, *N′-*NO_2_ PD 5 yielded *N-*Et, *N′-*NH_2_Ph PD 2. The synthetic details for the synthesis of PDs 2–6 are provided in section 2.1 of the ESI.† It was thought, *prima facie* that the ^1^H NMR chemical shift of the vinylic proton gamma to the aromatic ring on PDs 2–6 (shown in Fig. 2 for emphasis) may provide some insight on the PDs' electrophilicity with the addition of electron withdrawing groups generally resulting in this peak being shifted more downfield (Fig. 2). This data was also useful to compare against PD 1 (Fig. 2).

### Kinetic constants assessed by UV-Vis

**Scheme 1:**
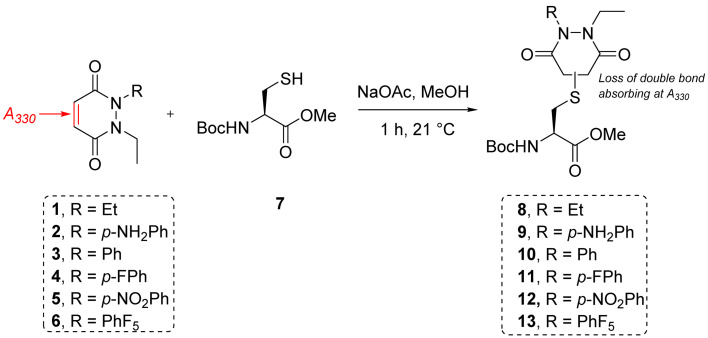
Reaction between small molecule cysteine Boc-Cys-OMe 7 and the library of PDs 1–6 in MeOH using sodium acetate as base to form conjugates Boc-Cys(PD)-OMe 8–13.

To measure and assess the kinetics of thiol–PD reactivity, it was decided to exploit the specific absorbance of the pyridazinedione scaffold at 330 nm, as once it is conjugated to a thiol, this absorbance band usually disappears. As such, it was hypothesised that the evolution of absorbance at 330 nm could be used to track the release of a PD from a thiol–PD conjugate (or addition of a thiol to a PD, respectively) over time. To confirm that it was appropriate to use UV-Vis analysis in this manner, model reactions between a small molecule cysteine Boc-Cys-OMe 7 and the library of PDs 1–6 was carried out (Scheme 1). All PDs reacted to afford resulting conjugates Boc-Cys(PD)-OMe 8–13. These conjugates were isolated, the products confirmed by NMR (see section 2.2 in ESI for details†) and the absorbance at 280 and 330 nm measured. Pleasingly, analyses confirmed that the kinetics of the reaction could be assessed by UV-Vis for most PDs with the extinction coefficient of each PD determined at 280 and 330 nm. However, *N*-Et, *N′*-NO_2_Ph PD 5 retained a high absorbance at 330 nm after conjugation, making it difficult to differentiate from non-conjugated PD. For this reason, this PD was excluded from kinetic assays, but the example of *N*-Et, *N′*-F_5_Ph PD 6 was thought to be adequate as a representative example of a highly electrophilic PD. It was also appreciated from the outset that *N*-Et, *N′*-F_5_Ph PD 6 could display novel reactivity in view of the presence of many highly electronegative fluorine atoms.

**Fig. 3:**
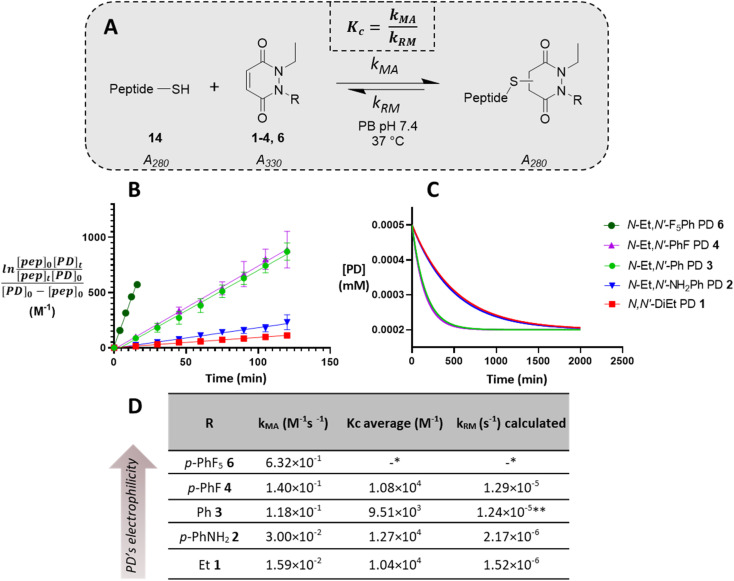
Summary of kinetic assays. (A) Scheme of the reaction between the peptide GCY 14 and the library of PDs 1–4 and 6 in phosphate buffer at pH 7.4. (B) Second-order reaction integrated law plotted for the library of PDs 1–4 and 6 (*n* = 3, some error bars are smaller than markers), slope is *k*_MA_ (M^−1^ min^−1^); (C) normalised time to reach equilibrium for the library of PDs 1–4; (D) Michael addition rate constants (*k*_MA_), equilibrium constants (*K*_c_) and retro Michael rate constants (*k*_RM_) for the library of PDs 1–4 and 6. *These values were not assessed due to stability issue of the PD–GCY conjugate under the conditions of the equilibrium constant kinetic assay. **This value was confirmed experimentally (see section 3.5 in ESI for details†).

To appraise the kinetics, we initially set up a UV-Vis kinetics assay where we measured the disappearance of absorbance at 330 nm to assess the Michael addition (MA) rate constant for the addition of a thiol to the various PDs (Fig. 3A). Using a cysteine-containing tripeptide (GCY 14) as the model thiol, reaction with each PD was set at a high concentration (5 mM). For solubility reasons and to maintain buffering capacity, the assay was run in 100 mM phosphate buffer (PB) pH 7.4 : MeCN (7 : 3). To avoid competing oxidation of the cysteine to its disulfide, the buffer also contained 1 mM EDTA; we note that under these conditions no oxidation of GCY was observed in the time range the assay was carried out (see 3.2 in ESI†). Each assay was repeated in triplicate to ensure reproducibility and negligible deviation was found within the repetitions. Gratifyingly, the assays showed that the MA rate constant increased with the NMR predicted electrophilicity of the PDs (see Fig. 2) with about a 40-fold difference in rate between *N*-Et, *N′-*Et PD 1 and *N*-Et, *N′-*F_5_Ph PD 6. Pleasingly, *N*-Et, *N′-*F_5_Ph PD 6 displayed *ca.* 5-fold quicker addition than the *N*-Et, *N′-*FPh PD 4 (see Fig. 3D and ESI section 3.3†), demonstrating clear tunability of the reaction using different PDs. In addition, it was found that for the highly electrophilic PD 6, an equilibrium was reached rather quickly (within 45 minutes – plateau not plotted in Fig. 3C), at the concentration of the assay.

To gain a better understanding of the reaction equilibrium between the various PDs with model peptide GCY 14, an assay was developed to determine the equilibrium constant, *K*_c_. For this, reaction of GCY with each applicable PD (*i.e.*, PDs 1–4 and 6) was left to incubate in a cuvette for 16 h at 37 °C at a lower concentration (300–800 μM for both the peptide and PD) in PB pH 7.4, in order to best quantify by UV-Vis. It was hypothesised that the equilibrium would be reached within this timeframe for highly electrophilic PDs, and for the PDs that were slower to reach equilibrium we could model the equilibrium based on a projected curve.^53^ Perhaps as expected, the more electrophilic PDs reached equilibrium faster, whilst the EDG-bearing PDs 1 and 2 did not reach equilibrium within the 16 h of the assay. Nonetheless, a very similar *K*_c_, within standard deviation, of *ca.* 1 × 10^4^ M^−1^ was found for most of the library of PDs, implying that the different modifications applied to the core PD scaffold impacted the MA and RM rates to a similar extent. However, it was noted that the exceptionally electron poor PD (*i.e.*, *N*-Et, *N′*-F_5_Ph PD 6) reproducibly exhibited an outlier *K*_c_ value. With a *K*_c_ double that of any other (apparent *K*_c_ of 2 × 10^4^ M^−1^), PD 6 appeared to display a more favoured forward reaction, *i.e.*, towards the thiol-conjugated product. To probe this further, LC-MS analysis in fact revealed that the *N*-Et, *N′*-F_5_Ph PD–GCY conjugate 15, presumably due to its extremely high electrophilicity, was undergoing slow hydrolysis over this time period (see section 3.6 in ESI†). This is in fact the first example of a thiol–PD conjugate undergoing hydrolysis albeit under an extreme circumstance. This hydrolysis was likely to irreversibly “lock” the Michael addition product in place, preventing the retro-Michael reaction taking place, and thus explaining the apparently higher *K*_c_ value. Hence, the RM rate constant for this PD was not obtainable using the aforementioned kinetics assay, but as hydrolysis was not observed in the timescale for the Michael addition reaction (see section 3.6.1 in ESI†), that data is still valid.

From the kinetic assay results of the MA rate constants and the equilibrium constants obtained for PDs 1–4, the values of the RM reaction rate constants were calculated (see Fig. 3D). As expected, a similar trend as the one found for the MA kinetic assay was found for the library of PDs, with the rate of deconjugation positively correlating with the likely acidity of the applicable proton in each thiol–PD conjugate. Finally, to corroborate that the calculated *k*_RM_ were reliable, a kinetic assay to experimentally determine the RM rate constant was designed (see section 3.5 in ESI†). This was carried out on *N*-Et, *N′-*Ph PD–GCY conjugate S14 and it gave an average *k*_RM_ of 1.82 × 10^−5^ s^−1^ (±1.43 × 10^−6^ s^−1^), which is similar to the calculated *k*_RM_ (1.24 × 10^−5^ s^−1^ ± 1.88 × 10^−6^ s^−1^). Overall, we demonstrated that by functionalising the PD scaffold with an aromatic system on one of the nitrogen atoms, we could tune the rate of thiol addition and release. We also carried out DFT calculations of the addition of methanethiolate to the *N*-methyl analogues of 1–4 and 6. The calculated activation energies showed a good correlation with the experimental rate constants for the addition reaction (see section 5 in ESI†).

### Regioselectivity

**Fig. 4:**
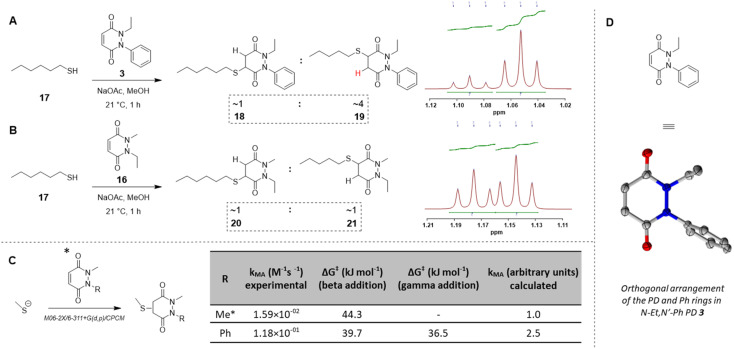
(A) Reaction of *N*-Et, *N*′-Ph PD 3 with *n*-hexanethiol 17 yielded a 1 : 4 regioisomer product ratio of 18 : 19 as assessed by NMR analysis. (B) Reaction of *N-*Me, *N*′-Et PD 16 with *n*-hexanethiol 17 yielded about 1 : 1 regioisomer product ratio of 20 : 21 as assessed by NMR analysis. (C) DFT calculation of activation energy for the reaction between MeSH as the thiolate and either *N*,*N*′-DiMe or *N*-Me, *N*′-PhPD (addition of the thiol ‘beta’ to the phenyl or ‘gamma’ to the phenyl), using M06-2X/6-311+G(d,p)/CPCM level of theory, displaying the favoured product and confirming the observations found experimentally in 3D, 4A, 4B. *Calculations were run with *N*-Me PD as a model PD to simplify the calculations but was expected to behave similarly than *N*-Et PD. (D) The orthogonal arrangement of the PD and Ph rings in *N*-Et, *N*′-Ph PD 3, as observed in its crystal structure (see section 6 in ESI†).

Before moving on to study the reactivity of the aforementioned PDs on proteins and on a material, we were interested to see if functionalisation of the PD played a role in the regioselectivity of the reaction, *i.e.*, whether the cysteine would more likely add beta- or gamma-relative to the aromatic group on the PD. To investigate this, *N*-Et, *N′*-Ph PD 3 and *N*-Me, *N′*-Et PD 16 (see section 2.4 in ESI†) were reacted with a simple model thiol, *n*-hexanethiol 17. Whilst both possible regioisomers were obtained in both reactions (*i.e.*, addition to both sides of the alkene), the ratio of the regioisomers was dependent on the *N*-functionalisation of the PD. Interestingly, it was found that the PD bearing a phenyl group on one of the nitrogens (*i.e.*, PD 3) afforded a *ca.* 4 : 1 gamma/beta regioisomers ratio with preference for gamma addition relative to the phenyl group (as evidenced by NMR, see Fig. 4A and section 2.4 in ESI for details†) whilst the PD bearing similar alkyl groups on both nitrogen atoms (*i.e.*, PD 16) yielded a *ca.* 1 : 1 ratio (Fig. 4B). This suggested that the substituents on the nitrogen atoms have a significant impact on the regioselectivity of the reaction. This trend is in accordance with the hypothesis that the phenyl group is acting as a net electron withdrawing moiety, thus making the adjacent carbonyl group more electron-poor, hence favouring the reaction of a thiol gamma to the phenyl group. DFT calculations proved a useful tool to predict and confirm this experimental observation. Free energies of activation for the addition of methanethiolate to *N-*Me, *N′*-Ph PD were calculated using the method described by Houk^54^ and found to be 39.7 kJ mol^−1^ to afford the beta product 18 and 36.5 kJ mol^−1^ for the gamma product 19, which translates to a *ca.* 1 : 3.7 predicted ratio of regioisomers (Fig. 4C). This preference for gamma addition also somewhat fits with observed RM susceptibility data, *i.e.*, the phenyl group acting as an electron withdrawing moiety making the adjacent carbonyl group more electron-poor, which in turn makes the applicable proton on the major regiosomer 19 (depicted in red) likely to be more acidic than the corresponding one in minor regioisomer 18.

### Determining the underlying reason for the difference in reactivity between *N*-aryl and *N*-alkyl bearing PDs and applications beyond reversible thiol modification

**Fig. 5:**
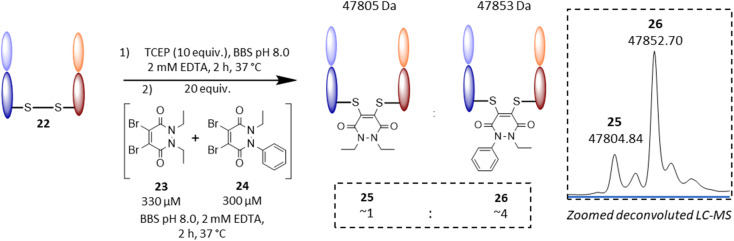
Competitive addition experiment of diBr *N*-Et, *N*′-Et PD 23*vs.* diBr *N*-Et, *N*′-Ph PD 24 to the reduced Fab fragment of Trastuzumab 22. On the right hand side: zoomed deconvoluted LC-MS illustrates re-bridged fab ratio of *ca.* 1 : 4 between 25 and 26.

Following on from these results, we were interested in understanding the underlying difference in reactivity between the aryl and alkyl functionalised PDs in more detail. For this, we optimised the geometry of *N*-Et, *N*′-Ph PD at the M06-2X/6-311+G(d,p)/CPCM(water) level; this calculation indicated that the phenyl group was twisted 67° out of plane from the PD ring. This result implied that the difference we were observing for the different PDs was primarily due to an inductive effect rather than a mesomeric delocalisation effect. To validate the DFT calculations, we obtained crystals of *N*-Et, *N′*-Ph PD 3 and ran single crystal X-ray diffraction (SCXRD) experiments to obtain the structure of this PD (see Fig. 4D, and sections 5 and 6 in ESI†). This confirmed the DFT prediction that the phenyl ring would not be at all coplanar with the PD ring. The crystal structure of PD 3 features two molecules with different conformations in the asymmetric unit; the PD/Ph angles are 74.15(3)° and 87.79(3)°. As well as being highly informative for this current work, this finding could have useful consequences for others working with pyridazinediones in this and other fields.^55–59^ In fact, we decided to explore one of these potentially useful knock-on consequences for the tuning of our previously reported disulfide rebriding reagents. Disulfide rebridging is a popular method for site-selective protein modification that has been applied to a range of fields and in particular for the production of antibody–drug conjugates.^60–63^ As such and owing to our extensive experience of using dibromopyridazinediones (diBr PDs) as disulfide rebridging reagents, we took the opportunity to appraise the thiol reactivity of a diBr *N*-alkyl, *N′*-aryl PD against a well-established diBr *N*-alkyl, *N′*-alkyl PD.^64^ To do this, we chose to use an antibody fragment containing one solvent accessible disulfide bond, *i.e.*, the Fab of Trastuzumab (anti-HER2 antibody) 22, which is a clinically validated antibody model.^65–67^ In a competition experiment of adding a pre-mixed solution of ∼1 : 1 diBr *N*-Et, *N′*-Et PD 23 and diBr *N*-Et, *N′*-Ph PD 24 (see section 4.2.3 in ESI for details†) to the reduced fab fragment, we observed a conjugation ratio of *ca.* 4 : 1 in favour of diBr *N*-Et, *N′*-Ph PD *vs.* diBr *N*-Et, *N′*-Et PD (Fig. 5). This highlights how we have unlocked a fundamental way of increasing the reactivity of pyridazinediones rather than it being specific to nonBr PDs; this could have applications in various contexts.^68–71^

### Synthesis of functionalisable PDs and application to proteins

**Fig. 6:**
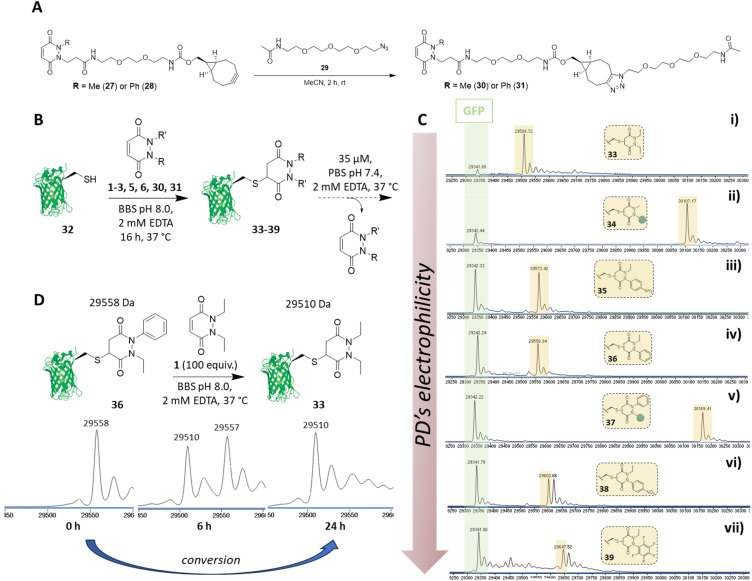
(A) “Click” reaction between amide-PEG2-N_3_29 and BCN bearing PDs 27 and 28 bearing either a methyl (27) or phenyl (28) group for tunability yielding *N*-Me, *N*′-clicked BCN PD 30 and *N*-Ph, *N*′-clicked BCN PD 31. (B) Generic scheme displaying reaction of 7 different PDs with GFP S147C 32 (unmodified expected mass: 29 342 Da) and subsequent release of PD from the formed PD-GFP S147C conjugate, which was monitored by LC-MS. (C) LC-MS timepoint at 24 h after incubation of PD-GFP S147C conjugates 33–39 at pH 7.4 at 37 °C. (i) conjugate *N*,*N*′-DiEt PD-GFP S147C 33, expected mass: 29 510 Da; (ii) conjugate *N*-Me, *N*′-clicked BCN PD-GFP S147C 34, expected mass: 30 108 Da; (iii) conjugate *N*-Et, *N*′-NH_2_Ph PD-GFP S147C 35, expected mass: 29 574 Da; (iv) conjugate *N*-Et, *N*′-Ph PD-GFP S147C 36, expected mass: 29 559 Da; (v) conjugate *N*-Ph, *N*′-clicked BCN PD-GFP S147C 37, expected mass: 30 170 Da; (vi) conjugate *N*-Et, *N*′-NO_2_Ph PD-GFP S147C 38, expected mass: 29 603 Da; (vii) conjugate *N*-Et, *N*′-F_5_Ph PD-GFP S147C 39, expected mass: 29 649 Da; (D) Dynamic modification of GFP S147C 32 starting from conjugate *N*-Et, *N*′-Ph PD-GFP S147C 36 to conjugate *N*,*N*′- DiEt PD-GFP S147C 33 within 24 h (extra peaks are due to Na^+^ adducts).

Before proceeding to investigate the reactivity of the library of PDs on a protein, in view of building in modularity and enabling future applications in a facile manner, we prepared strained alkyne bearing *N*-Me, *N*′-alkylBCN and *N*-Ph, *N*′-alkylBCN PDs 27 and 28 (see section 2.5 in ESI for details†). We chose to use methyl and phenyl functionalisation to avoid any potential issues with hydrolysis and as these PD could be expected to have fairly distinct reaction profiles in view of the above results. After performing “click” reactions with amide-PEG2-N_3_29 to mimic the attachment of a cargo (see Fig. 6A), the kinetic profiles of PDs 30 and 31 were assessed by UV-Vis in the assays developed (see sections 3.3 and 3.4 in ESI for kinetic details†). Consistent with the aforementioned studies, we demonstrated that PDs 30 and 31 displayed relatively slow and fast kinetics (respectively).

We next set about appraising whether the deconjugation profile of the PDs 1–3, 5, 6, 30 and 31 observed in the peptide studies would translate to deconjugation from a protein scaffold. Owing to the very similar kinetics between *N*-Et, *N′*-Ph PD 3 and *N*-Et, *N′*-FPh PD 4 and to simplify, data for PD 4 is not shown in the manuscript but can be found in section 4.1.3 of the ESI. For exemplification on a cysteine bearing protein, we used a cysteine mutant of GFP (*i.e.*, GFP S147C 32). The combination of our experience with working with GFP S147C,^52^ and it only bearing one solvent-accessible cysteine, made it a good model for our proof-of-concept studies on a protein. To begin, PDs 1–3, 5, 6, 30 and 31 were reacted with GFP S147C 32 (post-reduction to remove any disulfide dimer) at 37 °C. Consistent with our kinetic assays on a peptide, less electrophilic PDs (*i.e.*, PDs 1, 2 and 30) displayed slow addition to GFP S147C 32, they required more equivalents of PD and 16 h incubation time at 37 °C to reach completion, whilst the more electrophilic PDs (*i.e.*, PDs 3, 5, 6 and 31) were fully conjugated after 1 h at 37 °C. However, for simplicity and consistency we carried out all conjugations in this study at 37 °C for 16 h (see section 4.1 in the ESI†). After removing the excess of each PD, the formed conjugates 33–39 were left to incubate at 37 °C in PBS buffer (pH 7.4, 2 mM EDTA) at 35 μM and timepoints taken using LC-MS at *t* = 0, 2.5 h, 6.5 h, 24 h, 48 h and 72 h. At this concentration, the deconjugation of PD was expected to be slow enough to readily discriminate between the different PDs but fast enough to reach almost full deconjugation for the most electrophilic PDs within 24 h (calculated from the rate constants obtained previously). We also note that re-oxidation of neat GFP S147C 32 to form GFP S147C disulfide dimer (which would skew LC-MS analysis) took place slowly, as evidenced by SDS-PAGE gel and LC-MS, but was visible post 24 h (see section 4.1 in ESI†); this meant that the 24 h time-point would be a useful reference point to compare deconjugation by LC-MS analysis. Gratifyingly, we found that the PDs displayed a rate of deconjugation comparable to that displayed in our peptide studies. Interestingly, clicked PDs 30 and 31 exhibited a relatively slow and fast rate of deconjugation (respectively), which was attributable to functionalisation of one of the nitrogen atoms with either a methyl or a phenyl group (respectively).‡‡Conjugates of GFP S147 32 with N-Me and N-Ph maleimide were also prepared, appraised and analysed under the same conditions. Both conjugates, unlike the analogous PD-based conjugates, displayed hydrolysis after 24 h, see section 4.1.6 in the ESI for details.† As a reference point, after 24 h, *ca.* 60% of the phenyl PD 31 had been released whereas, at the same time, only *ca.* 20% of otherwise analogous methyl PD 30 was released (see Fig. 6C (ii) and (v)). This implies that we can tune the release of these functionalised PDs, which could prove useful for various applications as detailed above. As observed with *N*-Et, *N′*-F_5_Ph PD-GCY peptide conjugate 15 (see 3.6 in ESI for details†), the highly electron poor nature of the aryl ring on the PD of *N*-Et, *N′*-NO_2_Ph PD-GFP S147C 38 also resulted in some hydrolysis, which accounts for the second peak observed for the conjugate on LC-MS (see Fig. 6C (vi) and (vii), and section 4.1 in the ESI†). The additional masses observed for conjugate *N*-Et, *N′*-F_5_Ph PD-GFP S147C 39 (*i.e.*, 29 439 Da and 29 457 Da) could be tentatively attributed to the formation of GFP S147C-succinic anhydride (+97 Da) and GFP S147C-succinic acid (+115 Da) (respectively), which could be derived from the hydrolysed product of *N*-Et, *N′*-F_5_Ph PD-GFP S147C 39 undergoing cyclisation to form a succinic anhydride, which could then undergo further hydrolysis to form the succinic acid. As mentioned above, these hydrolysed species cannot undergo RM reaction. It is noteworthy that, despite hydrolysis as a competing side-reaction slowing down the release process, *N-*Et, *N′*-NO_2_Ph and *N*-Et, *N′*-F_5_Ph PD-GFP S147C conjugates 38 and 39 still displayed very fast deconjugation with almost full release within the first 24 h. It is also very important to emphasise that during the timeframe of this study (72 h) and under these reaction conditions, no other PD-conjugates underwent hydrolysis and that they displayed similar kinetics to that expected from the aforementioned kinetic studies. Further experiments were also conducted on *N*-Et, *N*′-NO_2_Ph PD-GFP S147C conjugate 38 to assess if hydrolysis could be circumvented at lower pH. To this end, conjugate 38 was synthesised and incubated in buffer at pH 6.0 and LC-MS analysis was carried out after 2 h, 6 h, 21 h, 48 h, 4 days, 5 days and 9 days (see section 4.1.4 in ESI for details,† including parallel reactions at pH 7.4 and 9.0). Pleasingly, under the pH 6.0 conditions, an appreciable amount of PD was still released within 24 h and no apparent hydrolysis was observed even after 9 days, suggesting that hydrolysis could be controlled by pH whilst PDs could still be released under these conditions. Additional experiments were also carried out to assess the dynamic nature of PDs with the free cysteine of GFP S147C 32 (Fig. 6D). For this, *N-*Et, *N*′-Ph PD GFP S147C conjugate 36 was synthesised, isolated and then subjected to a high excess of *N,N*′-DiEt PD 1. This led to the conversion of *N-*Et, *N*′-Ph PD GFP S147C conjugate 36 to *N*,*N*′-DiEt PD GFP S147C conjugate 33 within 24 h (Fig. 6D).

**Fig. 7:**
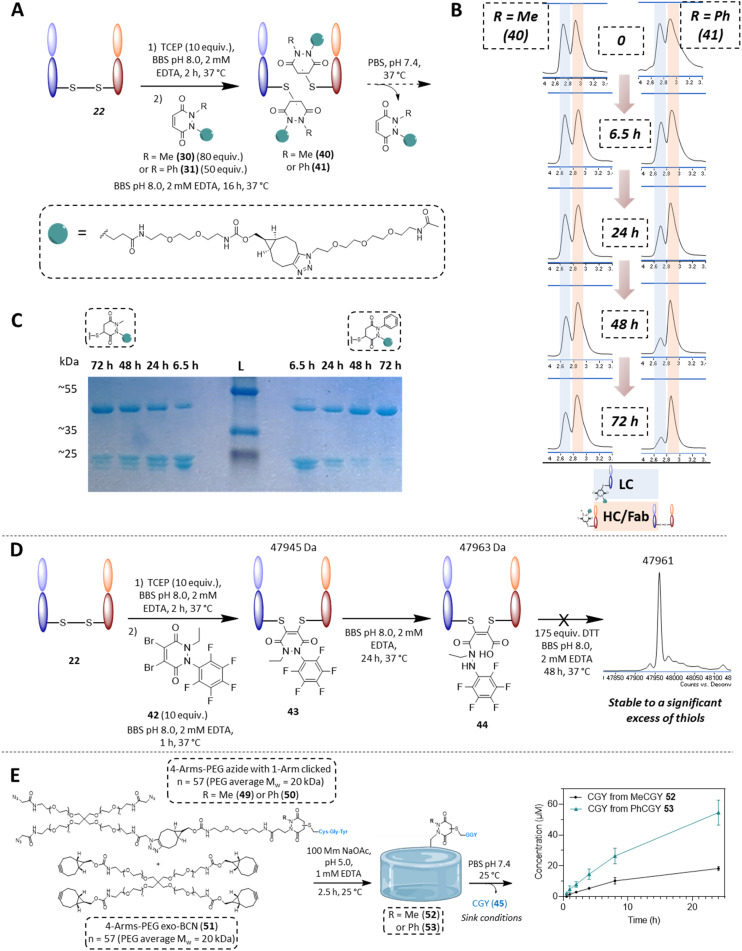
(A) Reaction of PDs 30 and 31 with a Fab fragment 22 and subsequent release of PD from the formed PD-Fab conjugates 40 and 41, which was monitored by LC-MS. (B) HPLC spectra of Fab-PD conjugate 40 (Me) or 41 (Ph) at pH 7.4 at 37 °C after 0 h, 6.5 h, 24 h, 48 h and 72 h of incubation. Highlighted in blue is the peak corresponding to the light chain fragment and highlighted in red is the heavy chain fragment + Fab (these species are inseparable under these conditions). Reduction of the peak highlighted in blue is faster for Fab PD conjugate 41 than Fab PD conjugate 40, demonstrating faster release when using PD 31. (C) SDS-PAGE gel of the release study of PDs from Fab-PD conjugates 40 and 41, expected masses: ∼24 kDa (LC/HC conjugates of Fab-PD) and/or ∼48 kDa for re-oxidised Fab fragment 22. Timepoints of 6.5 h, 24 h, 48 h and 72 h respectively for incubation of Fab-PD conjugates 40 and 41 at pH 7.4 at 37 °C. (D) Application of very electrophilic DiBr *N*-Et, *N*′-F_5_Ph PD 42 to disulfide rebridging of Fab 22, subsequent hydrolysis and stability in a high thiol concentration environment. (E) Deconjugation of CGY peptide 45 from hydrogels 52 or 53 (generated from CGY-MeBCNPD conjugates 47 or CGY-PhBCNPD conjugates 48), monitored by HPLC.

In the context of cargo release from a disulfide bearing protein, we wanted to appraise the conjugation and deconjugation of PDs 30 and 31. We did not include the other PDs for simplicity, as they would almost certainly behave as described on reaction with GFP S147C 32 and as PDs 30 and 31 represent good examples in terms of further applications and modularity. We chose the same aforementioned antibody fragment containing a single solvent accessible disulfide bond, *i.e.*, the Fab of Trastuzumab (anti-HER2 antibody) 22 as our disulfide model protein. Pleasingly, following reduction of its solvent accessible disulfide bond and reaction with PDs 30 and 31, homogeneously conjugated Fab fragments 40 and 41 were obtained (see Fig. 7B and section 4.2.2 in ESI†). Both conjugates 40 and 41 were then left to incubate at 37 °C (post removing any excess PD from solution), at a concentration of 35 μM and in PBS buffer pH 7.4. In this case, no EDTA was added to enable re-oxidation of the Fab disulfide. In contrast to the single cysteine containing protein model, re-oxidation to the native Fab disulfide acted as a trap to remove free thiols from being available for reaction. As expected after 48 h, almost full deconjugation was observed for phenyl functionalised PD 31 whilst a significant amount of the methylated version 30 was still conjugated as observed using LC-MS and densitometry (Fig. 7B and C). This serves to further emphasise that we can exert control over the rate of deconjugation by simply changing the substituent on one of the nitrogen atoms of a PD.

With Fab 22 in hand, we also took the opportunity to find out if our knowledge on the hydrolysis of *N*-Et, *N*′-F_5_Ph PD 6 could be exploited for disulfide rebridging. Previously, we have disclosed that conventional dialkyl bearing PD-disulfide conjugates are stable in blood and in various low thiol concentrations environments, but that these constructs undergo cleavage in a high concentration of thiol.^72^ However, by using a diBr variant of *N*-Et, *N*′-F_5_Ph PD 6 (*i.e.*, PD 42 (see 2.6 in ESI for synthesis details†)), we showed that a PD can undergo efficient disulfide rebridging, followed by quantitative hydrolysis to afford a conjugate (*i.e.*, conjugate 44) that was stable to a high concentration of thiol (Fig. 7D). This provides an elegant complementary reactivity to conventional dialkyl bearing diBr PD disulfide rebridging reagents. The hydrolysed construct was also shown to be stable over a protracted period (*ca.* 2 days) at pH 8.0 (see section 4.2.4 in ESI for details†), and a control study using diBr *N*,*N*′-DiEt PD showed quantitative cleavage in the same high concentration of thiol environment (see 4.2.4 in ESI for details†).

### Exemplification of peptide release from a hydrogel material

Finally, as an example of application for cargo release from a material, using “clickable” BCN-PDs 27 and 28, two BCN bearing PD-CGY conjugates 47 and 48 were synthesised (respectively, see 2.3 in ESI†). These BCN derivatives (47 and 48) were reacted with a 4-Arm PEG azide 46 in a 1 : 1 molar ratio to substitute one arm on average to obtain the corresponding 4-Arm PEG-N_3_-PD-CGY derivatives 49 and 50, whilst leaving three azides available for hydrogel crosslinking (Fig. 7E). This reaction was performed under acidic conditions (30% acetonitrile in 100 mM acetate buffer, pH = 5.0 with 1 mM EDTA) to prevent potential premature retro-Michael deconjugation of the peptides. The corresponding 4-Arm PEG-N_3_-PD-CGY derivatives, 49 and 50 were reacted with a 4-Arm PEG-BCN 51 under acidic conditions (100 mM acetate buffer, pH = 5.0 with 1 mM EDTA) at room temperature to give PEG hydrogels cross-linked 52 and 53*via* Strain-Promoted Azide–Alkyne cycloaddition (SPAAC, see section 4.3 for details†). After washing and equilibrating the hydrogels overnight at 4 °C in acetate buffer, the hydrogels were placed in PBS buffer (pH = 7.4), with the PBS being replaced at the respective timepoints to simulate sink conditions. The concentration of CGY peptide (45) released was measured in triplicate using HPLC *via* a standard curve. The cumulative release data of the CGY peptide in Fig. 7E shows an increase in the amount of CGY peptide released from the Ph-PD derivative (53) compared with the Me-PD derivative (52), which agrees with the trends found in solution. Crucially, the hydrogel release experiments demonstrate that tuning the rate of retro-Michael deconjugation based on electron withdrawing groups even holds true when the PD derivatives are immobilised on a material. Owing to the tuneable and reversible properties of PDs, this has potential applications in the design of materials for controlled release, such as for the temporal display of integrin binding peptides or as a refillable drug depot.^73–75^

## Conclusions

In this work, we demonstrate the potential of pyridazinediones as novel reversible and tuneable covalent cysteine modifiers. By developing modular synthetic strategies, we synthesised a library of *N*-functionalised pyridazinediones bearing electron-donating, electron-neutral and electron-withdrawing aryl groups. We assessed their kinetics profiles in terms of Michael addition, retro-Michael deconjugation and equilibrium constants using UV-Vis assays that were established and optimised in this work. We demonstrated that we were able to correlate the electrophilicity of a pyridazinedione with the rate of Michael addition/retro-Michael deconjugation, and thus demonstrated that pyridazinediones provide an interesting platform for modular, tuneable and reversible thiol addition and release. The regioselectivity of this reaction was investigated with the results suggesting that regioselectivity could be controlled by the nature of the groups on the nitrogen atoms. We further characterised the reasons for the fundamental increased reactivity of aryl bearing pyridazinediones by studying their structure using DFT calculations and corroborating findings by SCXRD experiments. With the phenyl ring of *N*-Et, *N*′-Ph PD 3 twisted by 74°–88° out of the plane of the PD ring, we obtained fundamental insight into the rationale behind our experimental results. We also applied this finding to making our previously reported diBr PD reagents more rapid disulfide rebridging agents. Finally, we laid the groundwork for potential applications in various areas with exemplification of our chemistry on clinically relevant cysteine and disulfide conjugated proteins, as well as on a material hydrogel, by showing different rates of deconjugation with readily functionalised “clickable” PDs on all systems.

## Data availability

The ESI† is available and it contains all the experimental data.

## Author contributions

L. N. C. R. and I. K. synthesised the molecules. L. N. C. R. and C. B. developed the kinetic assays. J. P. W. synthesised the peptides. L. N. C. R. carried out the kinetic assays. L. N. C. R. and A. E. A. performed the regioisomer studies. L. N. C. R. and P. B. generated the crystals. P. B. and D.-K. B. analysed the crystal. M. J. P. performed the DFT calculations. R. J. S. carried out the diBr PD competition experiments on Fab. I. A. T. generated the GFP S147C mutant. L. N. C. R. generated the Fab fragment. L. N. C. R. conducted the deconjugation studies. L. N. C. R. conducted the stability study on Fab. B. S. and L. Z. synthesised the 4-arm-PEG materials for the hydrogels. J. P. W. generated the hydrogel and conducted the release study from the hydrogel. L. N. C. R., C. B., J. R. B. and V. C. conceived the project and designed the project and/or experiments. L. N. C. R. and V. C. co-wrote the manuscript. All authors read and approved the final manuscript.

## Conflicts of interest

There are no conflicts to declare, but we highlight that V.C. and J.R.B. are directors of UCL spin-out ThioLogics.

## Supplementary Material

SC-014-D3SC04976K-s001

SC-014-D3SC04976K-s002
