# Supplementary material for: Use of pyridazinediones for tuneable and reversible covalent cysteine modification applied to peptides, proteins and hydrogels
Source: Chem Sci. 2023 Nov 1;14(47):13743–54. doi: 10.1039/d3sc04976k (PMC10699563; doi:10.1039/d3sc04976k)
Supplement: SC-014-D3SC04976K-s001 [file SC-014-D3SC04976K-s001.pdf]

Electronic Supplementary Material (ESI) for Chemical Science.
This journal is © The Royal Society of Chemistry 2023

Use of pyridazinediones for tuneable and reversible covalent cysteine modification applied to peptides, proteins and hydrogels

Léa N. C. Rochet,[a] Calise Bahou,[a] Jonathan P. Wojciechowski,[b] Ilias Koutsopetras,[c] Phyllida Britton,[a] Richard J. Spears,[a] Ioanna A. Thanasi,[a] Baihao Shao,[b] Lisha Zhong,[b] Dejan-Krešimir Bučar,[a] Abil E. Aliev,[a] Michael J. Porter,[a] Molly M. Stevens,[b] James R. Baker,[a]* and Vijay Chudasama[a]*

a: Department of Chemistry, University College London, 20 Gordon Street, London, WC1H 0AJ, UK.

b: Department of Materials, Department of Bioengineering, Institute of Biomedical Engineering, Imperial College London, London, SW7 2AZ, UK.

c: Bio-Functional Chemistry (UMR 7199), Institut du Médicament de Strasbourg, University of Strasbourg, 74 Route du Rhin, 67400 Illkirch-Graffenstaden, France.

*: Correspondence: v.chudasama@ucl.ac.uk, j.r.baker@ucl.ac.uk

Contents

1. General experimental ***3***

2. Small molecules and peptide synthesis ***10***

2.1 Library of PD synthesis **10**

2.2 PD-cysteine small molecule model conjugates **29**

2.3 Peptides and peptide-PD conjugates **40**

2.4 Regioisomers study **48**

2.5 Clickable and functionalised model PDs **61**

2.6 Disulfide rebridging reagents **81**

3. Kinetic assays on a peptide model ***86***

3.1 Extinction coefficients calculation **86**

3.2 Peptide oxidation study **87**

3.3 Michael Addition kinetic assay **88**

3.4 Equilibrium constant kinetic assay **90**

3.5 Retro Michael kinetic assay **92**

3.6 Stability study of N-Et,N'-F_5Ph PD 6 under kinetic assays conditions **94**

4. Protein/material experiments ***97***

4.1 GFPS147C experiments **97**

4.2 Fab experiments **183**

4.3 Hydrogel experiments **204**

5. DFT calculations ***211***

6. SCXRD experiment ***234***

7. References ***237***

Experimental section

1. General experimental

All chemical reagents were purchased from Sigma Aldrich, Alfa Aesar, Fluorochem, Iris Biotech, AGTC Bioproducts, Chem-Impex International, JenKem Technology or Acros. Compounds and solvents were used as received. Petrol refers to petroleum ether (b.p. 40–60 °C). All reactions were carried out under air, unless stated otherwise, and were monitored using thin layer chromatography (TLC) on pre-coated silica gel plates (254 µm). Flash column chromatography was carried out with pre-loaded GraceResolv™ Silica Flash cartridges (Grace™) or FlashPure EcoFlex catridges (Büchi) on a Biotage® Isolera Spektra One flash chromatography system (Biotage®). ^{1}H NMR spectra were obtained at 300 MHz, 400 MHz, 500 MHz, 600 MHz or 700 MHz. ^{13}C NMR spectra were obtained at 125 MHz, 150 MHz or 175 MHz. All results were obtained using Bruker NMR instruments, the models are as follows: Avance 300, Avance III 400, Avance Neo 500, Avance III 600 and Avance Neo 700. Unless otherwise specified, all samples were run at 25 °C. Chemical shifts (δ) for ^{1}H NMR and ^{13}C NMR are quoted on a parts per million (ppm) scale relative to tetramethylsilane (TMS), calibrated using residual signals of the solvent. Chemical shift (δ) for ^{15}N NMR are quoted relative to liquid NH_3, referenced using the unified scale.[1] Coupling constants (*J* values) are reported in Hertz (Hz) and are reported as *J* couplings between protons. Infrared spectra were obtained on a Perkin Elmer Spectrum 100 FTIR spectrometer operating in ATR mode. Mass spectra were obtained, for synthetic products, from the UCL mass spectroscopy service on a Waters LCT Premier XE (ES) mass spectrometer. For sections 2.3 and 4.3 characterisation data, ^{1}H NMR and ^{13}C NMR spectra were recorded on a JOEL 400 NMR spectrometer, with working frequencies of 400 MHz for ^{1}H nuclei, and 101 MHz for ^{13}C nuclei, respectively; high resolution ESI mass spectra were obtained on a Waters Quattro II ESI mass spectrometer; melting points were measured on a Cole-Parmer® MP80 series Stuart automatic digital melting point apparatus. High X-Ray data were obtained from grown single crystals and then analysed on a four-circle *Agilent SuperNova* (Dual Source) single crystal X-ray diffractometer. All Density Functional Theory calculations were carried out using Gaussian 16, revision C.01.[2]

UV-Vis spectroscopy

UV-Vis spectroscopy was used to determine PDs, peptides, proteins and peptide/protein conjugates concentrations using a NanoDrop OneC spectrophotometer (ThermoScientific) operating at 21 °C or 37 °C. Sample buffer was used as a blank for baseline correction with extinction coefficient ε_{280} = 1,490 M^{-1} cm^{-1} for GCY **14**, ε_{280} = 68,590 M^{-1} cm^{-1} for Trastuzumab Fab **22**, ε_{280} = 20,500 M^{-1} cm^{-1} for GFPS147C **32**.

LCMS analysis – Method 1

Molecular masses of proteins (<40 kDa) were measured at first using an Agilent 6510 QTOF LC-MS system (Agilent, UK). Agilent 1200 HPLC system was equipped with an Agilent PLRP-S, 1000A, 8 µM, 150 mm x 2.1 mm column. 10 µL of a protein sample (diluted to 0.2 mg/mL in LCMS grade water) was separated on the column using mobile phase A (water-0.1% formic acid) and B (acetonitrile-0.1% formic acid) with an eluting gradient (as shown below) at a flow rate of 0.4 mL/min. The oven temperature was maintained at 60 °C.

Time (min)	Solvent A (%)	Solvent B (%)
0	85	15
2	85	15
2.1	68	32
3.0	68	32
8.0	54	48
8.1	5	95
9.0	5	95
9.1	85	15
10	85	15

Agilent 6510 QTOF mass spectrometer was operated in a positive polarity mode, coupled with an ESI ion source. The ion source parameters were set up with a VCap of 4000 V, a gas temperature at 350 °C, a dry gas flow rate at 10 L/min and a nebulizer of 35 psig. MS Tof was acquired under conditions of a fragmentor at 175 V, a skimmer at 65 V and an acquisition rate at 1 spectra/s in a profile mode, within a scan range between 100 and 3100 *m/z*. The data was then analysed by deconvoluting a spectrum to a zero-charge mass spectrum using a maximum entropy deconvolution algorithm within the MassHunter software version B.07.00.

Molecular masses of proteins (<40 kDa) were then measured in a very similar way using an Agilent 6530 QTOF LCMS system (Agilent, UK). Agilent 1290 Infinity II UHPLC system was equipped with an Agilent PLRP-S, 1000 A, 8 µM, 50 mm × 2.1 mm column. 5 µL of a protein sample (diluted to 0.2 mg/mL in LCMS grade water) was separated on the column using mobile phase A (water-0.1% formic acid) and B (acetonitrile-0.1% formic acid) with an eluting gradient (as shown below) at a flow rate of 800 µL/min. The oven temperature was maintained at 60 °C.

LCMS mobile phase gradient for A/B elution:

Time (min)	Solvent A (%)	Solvent B (%)
0	80	20
1	80	20
6.5	40	60
7.5	40	60
7.6	80	20
8.5	80	20

Agilent 6530 QTOF mass spectrometer was operated in a positive polarity mode, coupled with an ESI ion source. The ion source parameters were set up with a VCap of 4000 V, a gas temperature at 350 °C, a dry gas flow rate at 10 L/min and a nebulizer of 35 psig. MS TOF was acquired under conditions of a fragmentor at 175 V, a skimmer at 65 V and an acquisition rate at 1 spectra/s in a profile mode, within a scan range between 100 and 7000 *m/z*. The data were then analysed by deconvoluting a spectrum to a zero-charge mass spectrum using a maximum entropy deconvolution algorithm within the MassHunter software version B.07.00. Deconvoluted spectra were avoided where possible in the quantification of conjugates due to differing ionisation tendencies between species with significantly different masses.

Some PEG contaminations coming from the resin of the purification columns have been sometimes observed on the HPLC spectra (peak eluting at 5.2 min). This does not impact the experimental observations.

When analysis was run on Agilent 6510 instead of Agilent 6530, it would be stated. No significant differences between the analysis ran on the two different instruments were observed, except a higher amount of sodium adducts being visible on Agilent 6530. This does not impact the experimental observations, as both instruments gave similar results overall.

LCMS analyses are shown as mentioned here: TIC LCMS trace (top), non-deconvoluted LC-MS trace (upper middle), wide range deconvoluted MS data (lower middle) and zoom in deconvoluted data (bottom) for each species.

LCMS analysis – Method 2

Molecular masses of proteins (<40 kDa) were measured at first using an Agilent 6510 QTOF LC-MS system (Agilent, UK). Agilent 1200 HPLC system was equipped with an Agilent PLRP-S, 1000A, 8 µM, 150 mm x 2.1 mm column. 10 µL of a protein sample (diluted to 0.2 mg/mL in LCMS grade water) was separated on the column using mobile phase A (water-0.1% formic acid) and B (acetonitrile-0.1% formic acid) with an eluting gradient (as shown below) at a flow rate of 0.4 mL/min. The oven temperature was maintained at 60 °C.

Time (min)	Solvent A (%)	Solvent B (%)
0	85	15
2	85	15
2.1	68	32
3.0	68	32
8.0	54	48
8.1	5	95
9.0	5	95
9.1	85	15
10	85	15

Agilent 6510 QTOF mass spectrometer was operated in a positive polarity mode, coupled with an ESI ion source. The ion source parameters were set up with a VCap of 3500 V, a gas temperature at 350 °C, a dry gas flow rate at 10 L/min and a nebulizer of 35 psig. MS Tof was acquired under conditions of a fragmentor at 350 V, a skimmer at 65 V and an acquisition rate at 0.5 spectra/s in a profile mode, within a scan range between 100 and 7000 *m/z*. The data was then analysed by deconvoluting a spectrum to a zero-charge mass spectrum using a maximum entropy deconvolution algorithm within the MassHunter software version B.07.00.

Molecular masses of proteins (>40 kDa) were then measured using an Agilent 6530 QTOF LCMS system (Agilent, UK). Agilent 1290 Infinity II UHPLC system was equipped with an Agilent PLRP-S, 1000 A, 8 µM, 50 mm × 2.1 mm column. 10 µL of a protein sample (diluted to 5 µM in LCMS grade water) was separated on the column using mobile phase A (water-0.1% formic acid) and B (acetonitrile-0.1% formic acid) with an eluting gradient (as shown below) at a flow rate of 300 µL/min. The oven temperature was maintained at 60 °C.

LCMS mobile phase gradient for A/B elution:

Time (min)	Solvent A (%)	Solvent B (%)
0	80	20
1	80	20
6.5	40	60
7.5	40	60
7.6	80	20
8.5	80	20

Agilent 6530 QTOF mass spectrometer was operated in a positive polarity mode, coupled with an ESI ion source. The ion source parameters were set up with a VCap of 3500 V, a gas

temperature at 350°C, a dry gas flow rate at 10 L/min and a nebulizer of 35 psig. MS TOF was acquired under conditions of a fragmentor at 350 V, a skimmer at 65 V and an acquisition rate at 1 spectra/s in a profile mode, within a scan range between 100 and 7000 *m/z*. The data were then analysed by deconvoluting a spectrum to a zero-charge mass spectrum using a maximum entropy deconvolution algorithm within the MassHunter software version B.07.00.

General sample preparation was carried out by removing the salts of the sample using Zeba™ Spin Desalting column (in LCMS grade water) and diluted the sample to 5 µM in LCMS grade water.
Some PEG contaminations coming from the resin of the purification columns have been sometimes observed on the HPLC spectra (peak eluting at 5.2 min). This does not impact the experimental observations.

When analysis was run on Agilent 6510 instead of Agilent 6530, it would be stated. No significant differences between the analysis ran on the two different instruments were observed, except a higher amount of sodium adducts being visible on Agilent 6530. This does not impact the experimental observations, as both instruments gave similar results overall.

LCMS analyses data are displayed as mentioned here: TIC LCMS trace (top), non-deconvoluted LC-MS trace (upper middle), wide range deconvoluted MS data (lower middle) and zoom in deconvoluted data (bottom) for each species.

LCMS analysis – Method 3
Peptide-PD conjugates for the stability studies were analysed using a Waters Acquity UPLC connected to Waters ACQUITY Single Quad Detector 2 (SQD2). All samples were diluted to a final concentration of 0.1 mg/mL in deionised water and run with the following parameters. Column: Hypersil Gold C4, 1.9 µm, 2.1 µm × 50 µm. Wavelength: 254 nm. Mobile Phase: 95:5 Water (0.1% Formic Acid): MeCN (0.1% Formic Acid) Gradient over 4 min (to 5:95 Water (0.1% Formic Acid): MeCN (0.1% Formic Acid). Flow Rate: 0.6 mL/min. MS Mode: ES+. Scan Range: m/z = 100 – 1000. Scan time: 0.25 s. Data obtained in continuum mode. The electrospray source of the MS was operated with a capillary voltage of 3.5 kV and a cone voltage of 50 V. Nitrogen was used as the nebulizer and desolvation gas at a total flow of 600 L/h. Ion series were generated by integration of the total ion chromatogram (TIC) over the appropriate range.

Preparative HPLC
The CGY and GCY peptides were purified using a Shimadzu Prominence LC-20A Preparative HPLC equipped with a Phenomenex Gemini C18 column (5 µm, 110 Å, 150 × 21.2 mm). Mobile phases: A = milliQ water with 0.1% (v/v) formic acid, B = HPLC grade acetonitrile with 0.1% (v/v) formic acid. Flow rate = 10 mL/min, detection wavelengths = 220 and 280 nm.

Prep-HPLC mobile phase gradient for A/B elution:

Time (min)	Solvent A (%)	Solvent B (%)
0	95	5
5	95	5
20	5	95
25	5	95
25.5	95	5
30	95	5

MALDI

MALDI spectra were obtained using a MALDI-8020 benchtop linear MALDI-TOF mass spectrometer tuned in positive mode. The matrix was 10 mg/mL α-cyano-4-hydroxy-cinnamic acid (CHCA) dissolved in 50% acetonitrile in water (v/v) with 0.1% trifluoroacetic acid. To prepare samples for MALDI, 0.5 µL of the polymer and 0.5 µL of matrix were deposited onto the MALDI target, mixed thoroughly, and allowed to dry at room temperature for 3 minutes. Samples were measured starting at low laser power (15) and increasing until signal was maximised without broadening. Molecular weights were determine based on comparison to Cytochrome c molecular weight standards.

Gel Permeation Chromatography (GPC)

The molecular weight of the PEG-PD-Peptide conjugates (Mn, GPC) and dispersity (Đ) were measured using a 1260 Infinity II GPC MDS system (refractive index detection only) equipped with a PSS GRAM guard column (8 × 50 mm, 10 µm) and two PSS GRAM linear columns (8 × 300 mm, 10 µm, 500–1,000,000 Da). The mobile phase was HPLC-grade *N*,*N*-dimethylformamide with 0.075% (w/v) LiBr. Samples were eluted at 40 °C as eluent with a flow rate of 1 mL/min. Molecular-weight calibration was performed using near-monodisperse poly(methyl methacrylate) standards (EasiVial, Agilent).

SDS-PAGE

Non-reducing glycine-SDS-PAGE at 12% acrylamide running were performed following standard lab procedures. A 4% stacking gel was used and a broad-range MW marker (10–250 kDa, Prestained PageRuler Plus Protein Standards, ThermoScientific) was co-run to estimate protein weights. Samples (8 µL at 12.5 µM) were mixed with loading buffer (2 µL, composition for 5 × SDS: 1 g SDS, 3 mL glycerol, 6 mL 0.5 M Tris buffer pH = 6.8, 2 mg bromophenol blue in 10 mL), heated at 75 °C for 5 min, and centrifuged at 16,000 RPM for 5 min. Samples were subsequently loaded into the wells in a volume of 6 µL. All gels were run at constant 150 V for

15 min, then constant 200 V until complete. Gels were stained using a Coomassie stain for 1 h at 21 °C and left to destain for 16 h.

DFT calculations

All DFT calculations were carried out using Gaussian 16, revision C.01.[2] Structures were optimized at the M06-2X/6-311+G(d,p)/CPCM(H_2O) level (as recommended by Houk *et al.*[3]). Structures were confirmed as minima or transition states by the presence of zero or one imaginary vibrational frequencies respectively. Transition states were shown to link with the relevant minima by means of intrinsic reaction coordinate (IRC) calculations.[4] Calculated coordinates of all structures are provided in section 5, and electronically as zipped .xyz files.

Single Crystal X-Ray Diffraction

SCXRD data were obtained using a four-circle Agilent SuperNova (Dual Source) single crystal X-ray diffractometer using a microfocus *CuKα* x-ray beam (λ= 1.54184 Å) and *a HyPix-Arc 100°* hybrid pixel array detector. The sample temperatures were controlled with an *Oxford Instruments* cryojet. All data were processed using the *CrysAlis^{Pro}* programme package from *Rigaku Oxford Diffraction*.[5] The crystal structures were solved with the *SHELXT* programme,[6] used within the *Olex2* software suite,[7] and refined by least squares on the basis of F^2 with the *SHELXL*[8] programme using the *ShelXle* graphical user interface.[9] All non-hydrogen atoms were refined anisotropically by the full-matrix least-squares method. Hydrogen atoms associated with carbon atoms were refined isotropically [U_{iso}(H) = 1.2Ueq(C)] in geometrically constrained positions.

The crystallographic and refinement parameters of **3** are given in Table S9. The asymmetric unit of the crystal structure of 3 is shown in Figure S11.

2. Small molecules and peptide synthesis

2.1 Library of PD synthesis

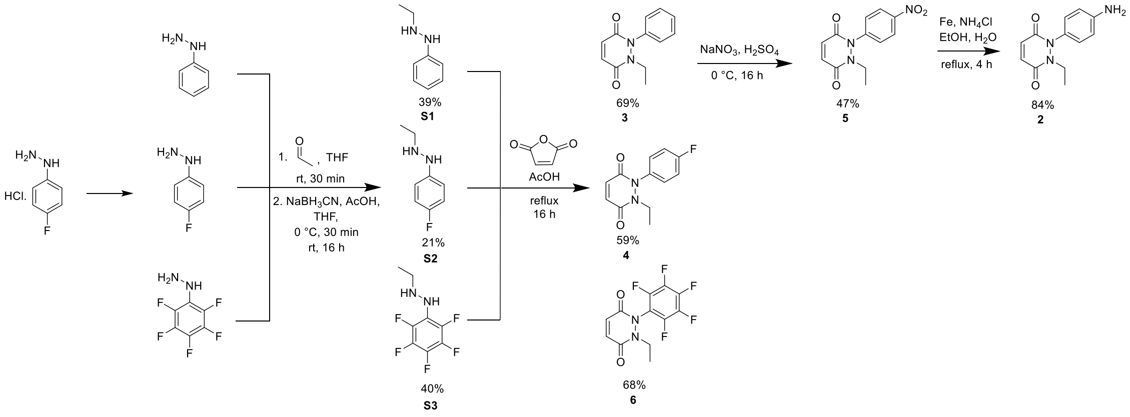

*Scheme S1. Synthesis of the library of PDs **2-6** starting from commercially available hydrazines.*

1,2-Diethyl-1,2-dihydropyridazine-3,6-dione 1[10]

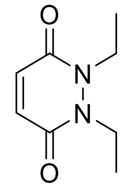

1

To a solution of maleic anhydride (0.51 g, 5.20 mmol) in glacial AcOH (20 mL) was added di-*tert*-butyl 1,2-diethylhydrazine-1,2-dicarboxylate obtained as previously described[11] (1.00 g, 3.46 mmol). The reaction mixture was then heated under reflux with stirring for 16 h. After this time, the reaction mixture was allowed to cool to 21 °C, and the solvent was removed *in vacuo* with toluene co-evaporation (3 x 20 mL, as an azeotrope). Residual toluene was subsequently removed *in vacuo* with chloroform co-evaporation (3 x 20 mL, as an azeotrope). The crude residue was then purified by flash column chromatography (20% to 80% EtOAc/petrol), yielding 1,2-diethyl-1,2-dihydropyridazine-3,6-dione **1** (0.32 g, 1.90 mmol, 55%) as a white solid.

^{1}H NMR (600 MHz, $CDCl_3$) δ 6.87 (s, 2H), 4.13 (q, *J* = 7.1 Hz, 4H), 1.26 (t, *J* = 7.1 Hz, 6H); **^{13}C NMR** (150 MHz, $CDCl_3$) δ 157.6 (C), 134.7 (CH), 40.3 (CH_2), 13.3 (CH_3); **IR** (solid) 2981, 1620, 1452 cm^{-1}.

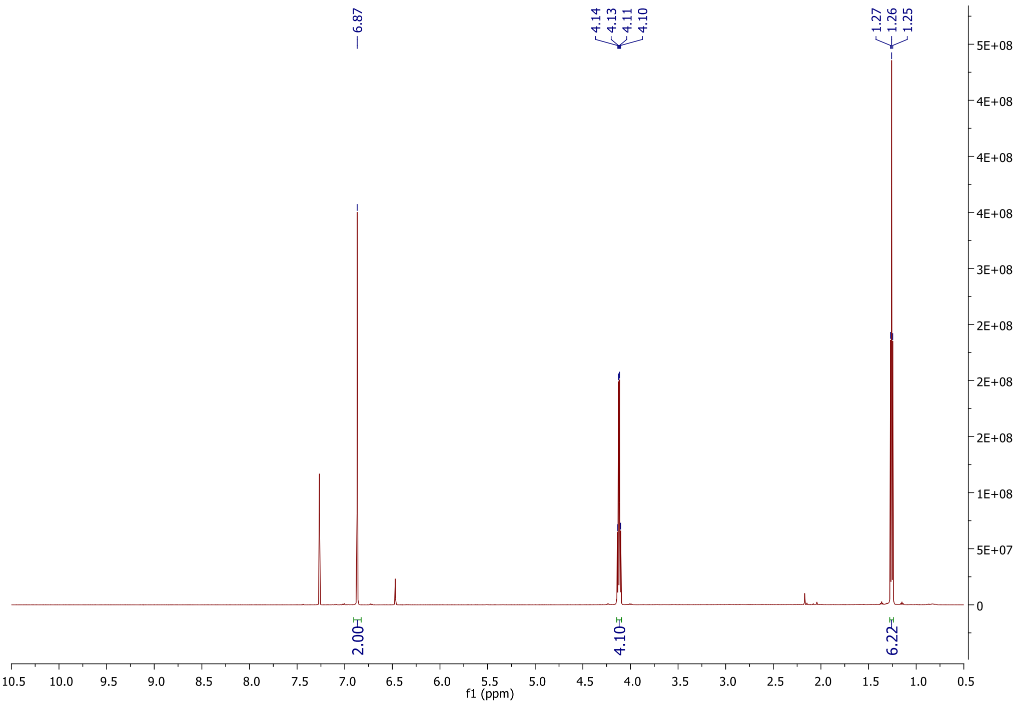
6.87
4.14
4.13
4.11
4.10
1.27
1.26
1.25
2.00
4.10
6.22
f1 (ppm)

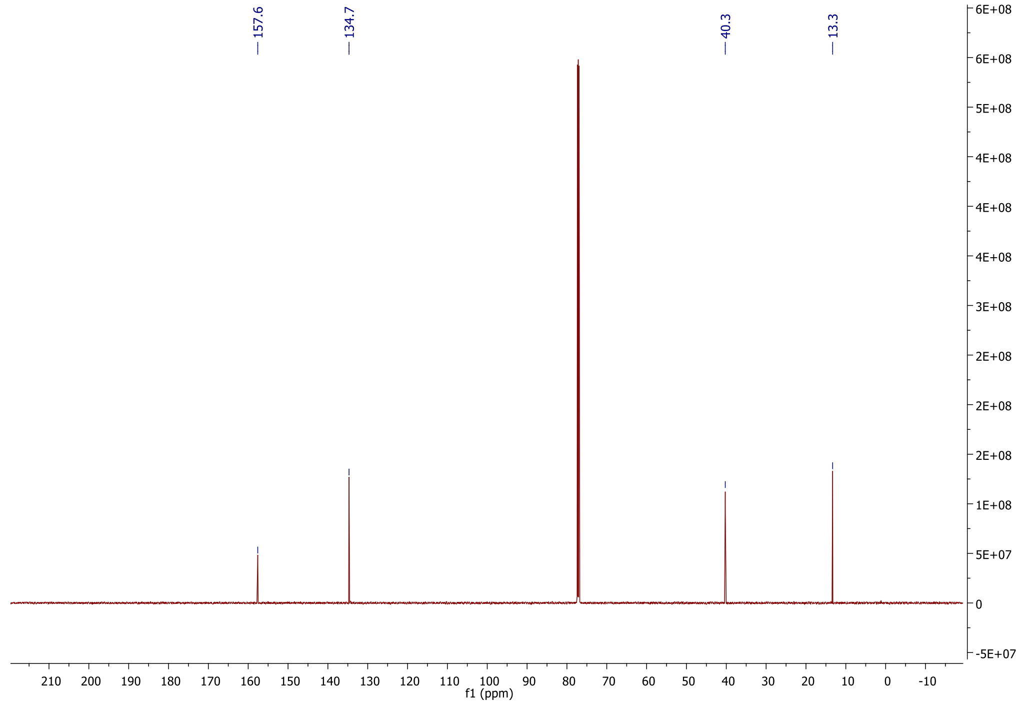
157.6
134.7
40.3
13.3
f1 (ppm)

1-Ethyl-2-phenylhydrazine S1

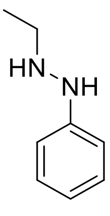

S1

To a solution of phenylhydrazine (1.00 mL, 10.2 mmol) in THF (10 mL) was added a solution of acetaldehyde (2.50 mL, 12.2 mmol, 5 M stock solution in THF) and the reaction was stirred for 30 min at 21 °C. After this time, solvent and excess acetaldehyde were removed *in vacuo*. The reaction mixture was then diluted in THF (60 mL). To this solution was added acetic acid (7 mL) and the solution was cooled to 0 °C. To this cooled solution was added, in small portions, sodium cyanoborohydride (0.96 g, 15.3 mmol). Following this, the reaction mixture was stirred for 15 min at 0 °C, then warmed to 21 °C and stirred for 16 h. After this time, THF and AcOH were removed *in vacuo* with toluene co-evaporation (3 × 20 mL, as an azeotrope). Residual toluene was subsequently removed *in vacuo* with chloroform co-evaporation (3 × 20 mL, as an azeotrope). Purification of the crude residue by flash column chromatography (0 to 40% EtOAc/cyclohexane) afforded 1-ethyl-2-phenylhydrazine **S1** as a yellow solid (551 mg, 3.97 mmol, 39%).

^{1}H NMR (600 MHz, $CDCl_3$, rotamers) δ 7.31 (t, *J* = 7.0 Hz, 3H), 7.06 (t, *J* = 7.0 Hz, 1H), 6.92 (d, *J* = 7.5 Hz, 2H), 6.90 (s, 1H), 3.23-3.12 (m, 2H), 1.30 (t, *J* = 7.2 Hz, 3H); **^{13}C NMR** (150 MHz, $CDCl_3$, rotamers) δ 143.2 (C), 129.9 (CH), 124.1 (CH), 117.4 (CH), 50.2 (CH_2), 9.8 (CH_3); **IR** (solid) 3313, 3106, 2969, 2846, 1610, 1468, 688 cm^{-1}; **LRMS** (ESI) 137 (100, $[M+H]^+$); **HRMS** (ESI) calcd for $C_8H_{13}N_2$ $[M+H]^+$ 137.1073, observed 137.1071.

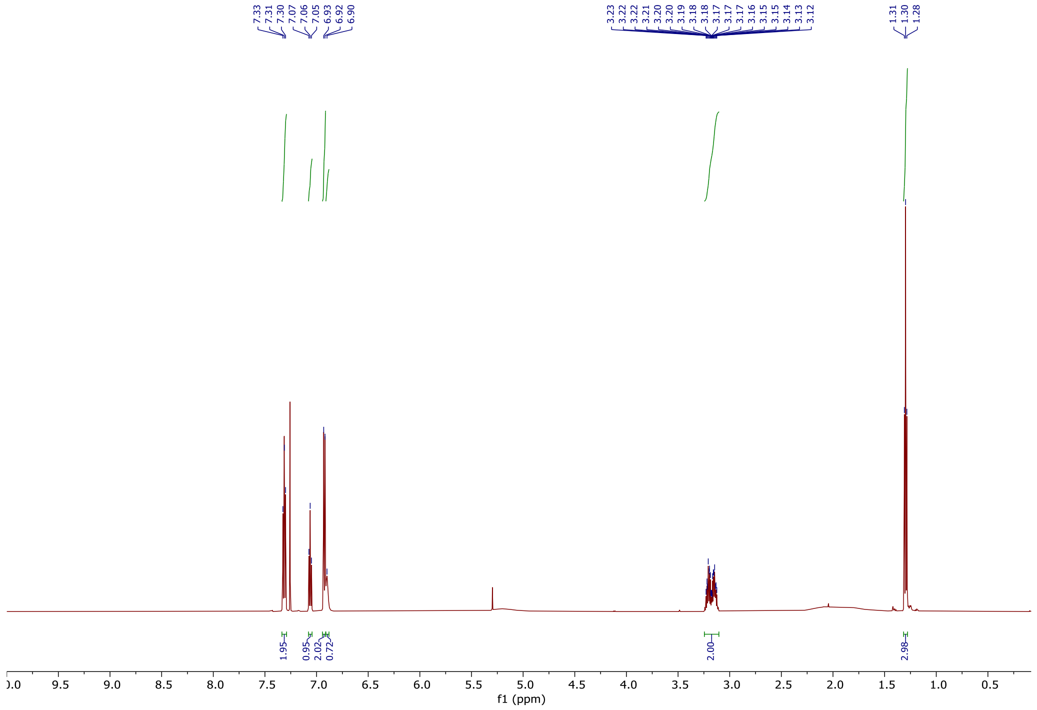
7.33
7.31
7.30
7.07
7.06
7.05
6.93
6.92
6.90
3.23
3.22
3.22
3.21
3.20
3.20
3.19
3.18
3.18
3.17
3.17
3.16
3.15
3.15
3.14
3.13
3.12
1.31
1.30
1.28
1.95
0.95
2.02
0.72
2.00
2.98
f1 (ppm)

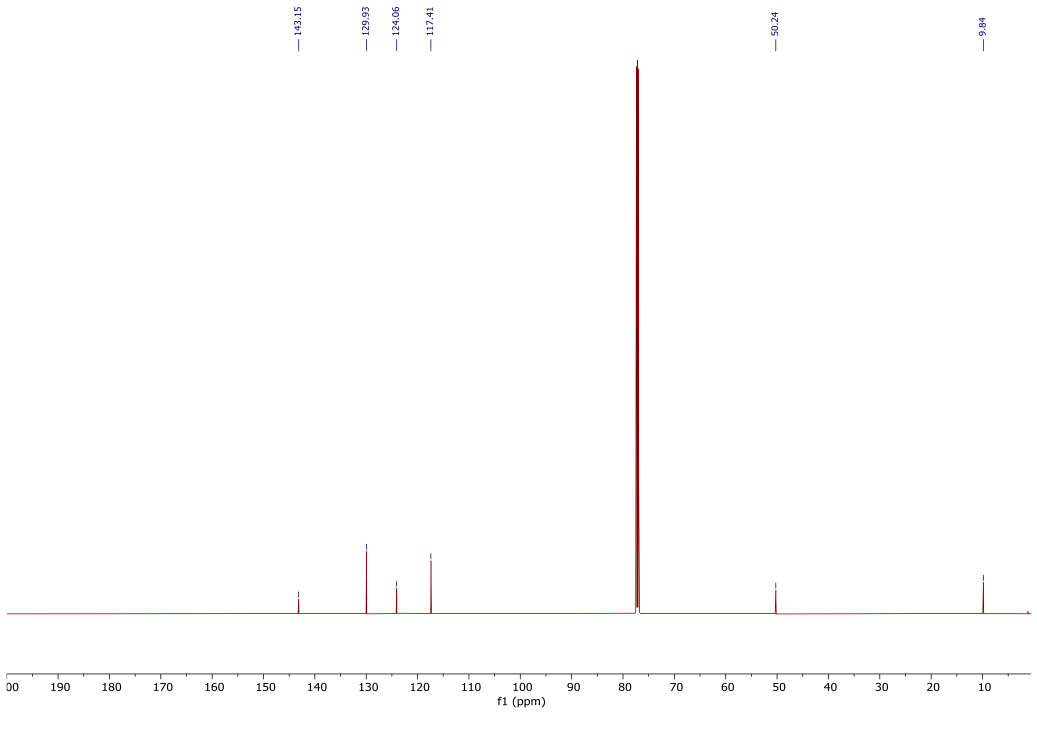
143.15
129.93
124.06
117.41
50.24
9.84
f1 (ppm)

1-Ethyl-2-phenyl-1,2-dihydropyridazine-3,6-dione 3

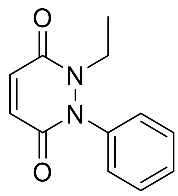

3

To a solution of 1-ethyl-2-phenylhydrazine **S1** (255 mg, 1.87 mmol) in AcOH (10 mL) was added maleic anhydride (219 mg, 2.24 mmol). The reaction mixture was then heated under reflux with stirring for 16 h. After this time, the reaction mixture was allowed to cool to 21 °C, and the solvent was removed *in vacuo* with toluene co-evaporation (3 x 20 mL, as an azeotrope). Residual toluene was subsequently removed *in vacuo* with chloroform co-evaporation (3 x 20 mL, as an azeotrope). Purification of the crude residue by flash column chromatography (0 to 60% EtOAc/cyclohexane) afforded 1-ethyl-2-phenyl-1,2-dihydropyridazine-3,6-dione **3** as a yellow solid (283 mg, 1.31 mmol, 69%).

^{1}H NMR (600 MHz, $CDCl_3$) δ 7.54 (t, *J* = 7.4 Hz, 2H), 7.48 (t, *J* = 7.4 Hz, 1H), 7.37 (d, *J* = 7.0 Hz, 2H), 6.99 (d, *J* = 10.1 Hz, 1H), 6.94 (d, *J* = 10.1 Hz, 1H), 3.74 (q, *J* = 7.0 Hz, 2H), 1.07 (t, *J* = 7.0 Hz, 3H); **^{13}C NMR** (150 MHz, $CDCl_3$) δ 157.9 (C), 157.4 (C), 136.1 (C), 135,9 (CH), 135.0 (CH), 129.9 (CH), 129.9 (CH), 128.5 (CH), 42.1 (CH_2), 12.7 (CH_3); **IR** (solid) 3054, 2930, 1627, 1571, 1419 cm^{-1}; **LRMS** (ESI) 217 (100, $[M+H]^+$); **HRMS** (ESI) calcd for $C_{12}H_{13}N_2O_2$ $[M+H]^+$ 217.0972, observed 217.0971.

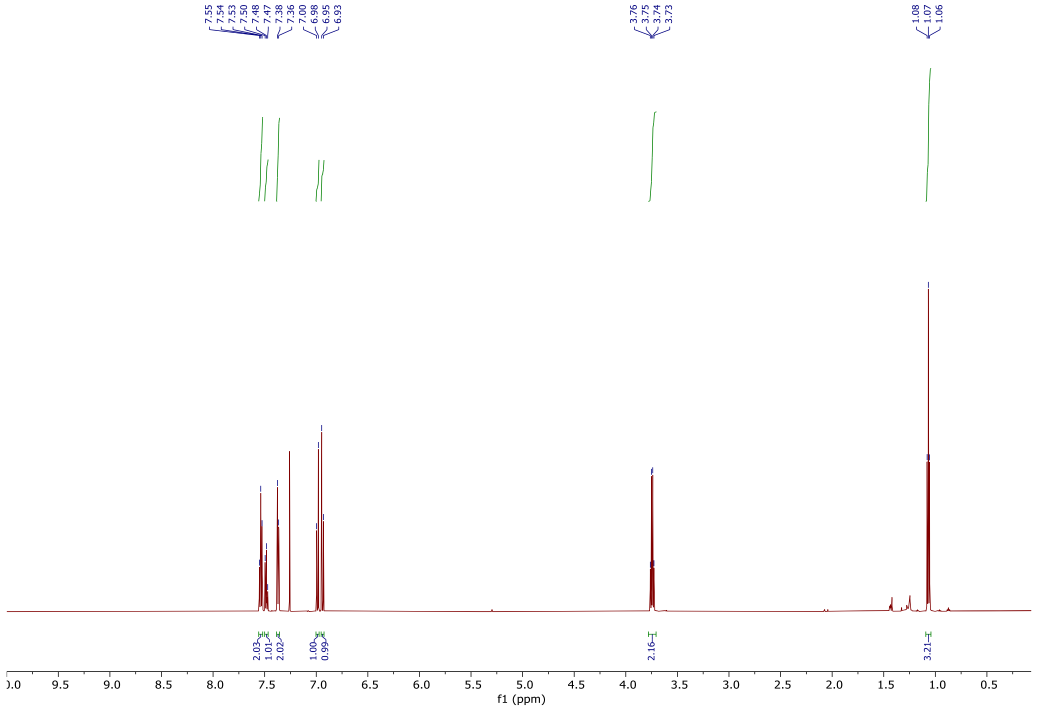
7.55
7.54
7.53
7.50
7.48
7.47
7.38
7.36
7.00
6.98
6.95
6.93
3.76
3.75
3.74
3.73
1.08
1.07
1.06
2.03
1.01
2.02
1.00
0.99
2.16
3.21
f1 (ppm)

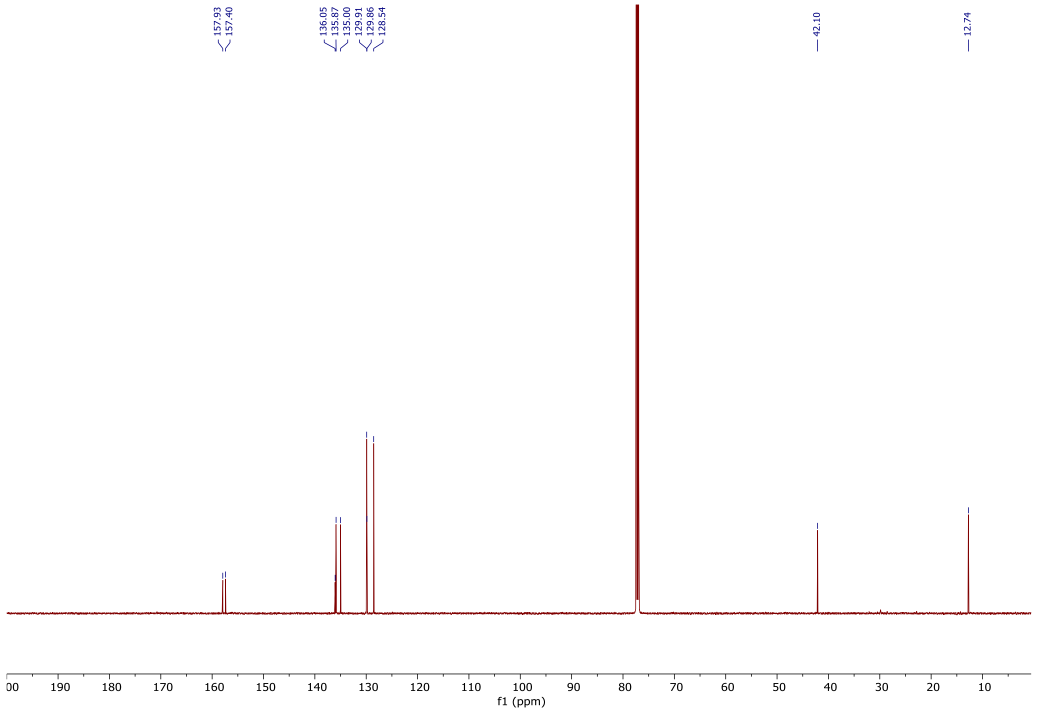
157.93
157.40
136.05
135.87
135.00
129.91
129.86
128.54
42.10
12.74
f1 (ppm)

1-Ethyl-2-(4-fluorophenyl)hydrazine S2

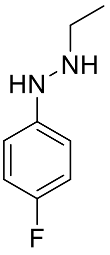

S2

4-Fluorophenylhydrazine hydrochloride (1.00 g, 6.17 mmol) was dissolved in a solution of NaOH (30 mL, 1 M aq.) and left to stir at 21 °C for 30 min. The neutralized product was extracted with EtOAc (3 × 30 mL), dried over $MgSO_4$ and the EtOAc was removed *in vacuo* to afford (4-fluorophenyl)hydrazine. To a solution of (4-fluorophenyl)hydrazine (690 mg, 5.48 mmol) in THF (7 mL) was added a solution of acetaldehyde (1.32 mL, 6.58 mmol, 5 M solution in THF) and the reaction was stirred for 30 min at 21 °C. After this time, the solvent and excess acetaldehyde were removed *in vacuo* and the reaction mixture was re-dissolved in THF (30 mL). To this solution was added acetic acid (5 mL) and the solution was cooled to 0 °C. To this cooled solution was added, in small portions, sodium cyanoborohydride (413 mg, 6.58 mmol). Following this, the reaction was stirred for 15 min at 0 °C, then warmed to 21 °C and stirred for 16 h. After this time, THF and AcOH were removed *in vacuo* with toluene co-evaporation (3 × 20 mL, as an azeotrope). Residual toluene was subsequently removed *in vacuo* with chloroform co-evaporation (3 × 20 mL, as an azeotrope). Purification of the crude residue by flash column chromatography (0 to 40% EtOAc/cyclohexane) afforded 1-ethyl-2-(4-fluorophenyl)hydrazine **S2** as a white solid (199 mg, 1.29 mmol, 21% over two steps).

^{1}H NMR (600 MHz, $CDCl_3$, rotamers) δ 7.01-6.97 (m, 4H), 5.07 (d, *J* = 7.1 Hz, 1H), 3.15-3.06 (m, 2H), 1.29-1.26 (m, 3H); **^{13}C NMR** (150 MHz, $CDCl_3$) δ 160.6 (C), 158.9 (C), 139.0 (CH), 120.8 (CH), 116.7 (CH), 116.5 (CH), 49.7 (CH_2), 9.6 (CH_3); **IR** (solid) 3323, 3098, 2932, 1654, 1328 cm^{-1}; **LRMS** (ESI) 155 (100, $[M+H]^+$); **HRMS** (ESI) calcd for $C_8H_{12}N_2F$ $[M+H]^+$ 155.0979, observed 155.0977.

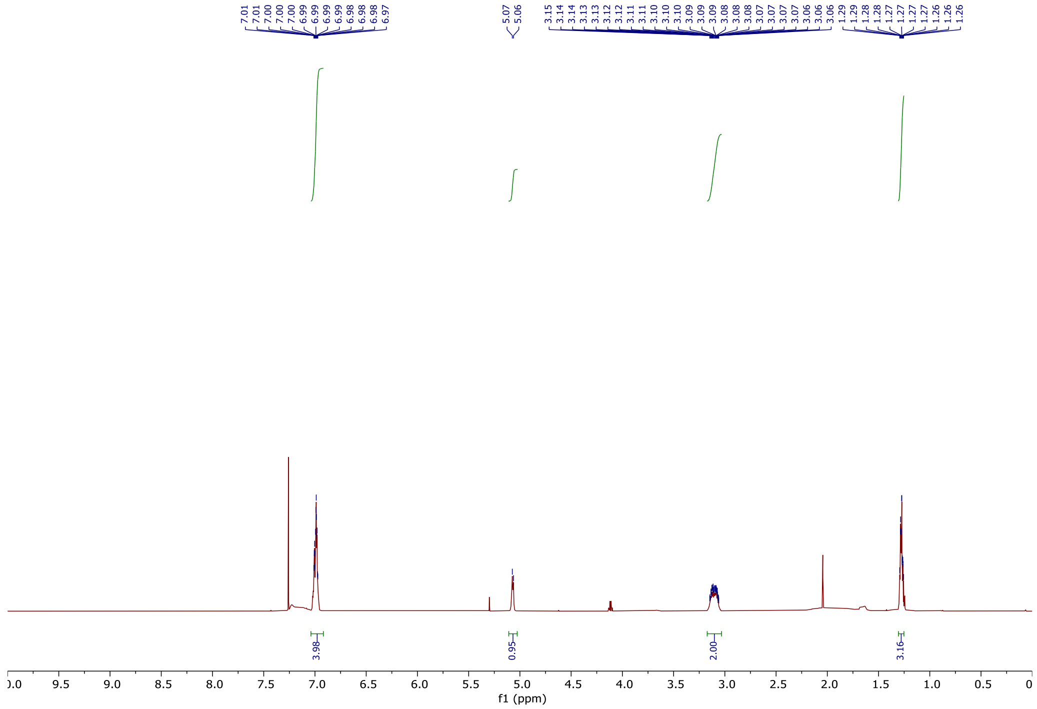
7.01
7.01
7.00
7.00
6.99
6.99
6.99
6.99
6.98
6.98
6.97
5.07
5.06
3.15
3.14
3.14
3.13
3.13
3.12
3.12
3.11
3.11
3.10
3.10
3.10
3.09
3.09
3.08
3.08
3.08
3.07
3.07
3.07
3.06
3.06
1.29
1.29
1.28
1.28
1.27
1.27
1.27
1.27
1.26
1.26
1.26
3.98
0.95
2.00
3.16
f1 (ppm)

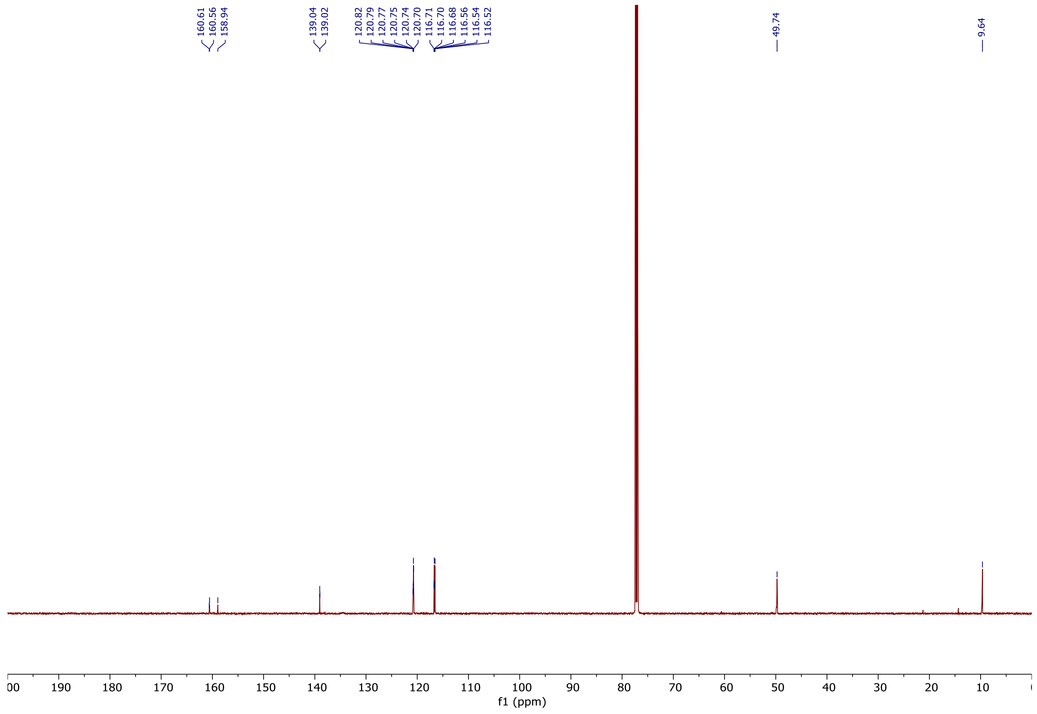
160.61
160.56
158.94
139.04
139.02
120.82
120.79
120.77
120.75
120.74
120.70
116.71
116.70
116.68
116.56
116.54
116.52
49.74
9.64
f1 (ppm)

1-Ethyl-2-(4-fluorophenyl)-1,2-dihydropyridazine-3,6-dione 4

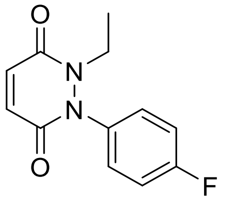

4

To a solution of 1-ethyl-2-(4-fluorophenyl)hydrazine **S2** (85 mg, 0.55 mmol) in AcOH (5 mL) was added maleic anhydride (63 mg, 0.64 mmol). The reaction mixture was then heated under reflux with stirring for 16 h. After this time, the reaction mixture was allowed to cool to 21 °C, and the solvent was removed *in vacuo* with toluene co-evaporation (3 x 20 mL, as an azeotrope). Residual toluene was subsequently removed *in vacuo* with chloroform co-evaporation (3 x 20 mL, as an azeotrope). Purification of the crude residue by flash column chromatography (0 to 60% EtOAc/cyclohexane) afforded 1-ethyl-2-(4-fluorophenyl)-1,2-dihydropyridazine-3,6-dione **4** as a yellow powder (75 mg, 0.32 mmol, 59%).

^{1}H NMR (600 MHz, $CDCl_3$) δ 7.36 (dd, *J* = 4.8, 8.9 Hz, 2H), 7.23 (t, *J* = 8.9 Hz, 2H), 7.00 (d, *J* = 10.1 Hz, 1H), 6.94 (d, *J* = 10.1 Hz, 1H), 3.75 (q, *J* = 7.0 Hz, 2H), 1.07 (t, *J* = 7.0 Hz, 3H); **^{13}C NMR** (150 MHz, $CDCl_3$) δ 163.7 (C), 162.0 (C), 158.1 (C), 157.4 (C), 136.0 (CH), 134.9 (CH), 130.5 (CH), 130.5 (CH), 117.1 (CH), 117.0 (CH), 42.1 (CH_2), 12.7 (CH_3); **IR** (solid) 3049, 2972, 2904, 1639, 1591, 1215 cm^{-1}; **LRMS** (ESI) 235 (100, $[M+H]^+$); **HRMS** (ESI) calcd for $C_{12}H_{12}FN_2O_2$ $[M+H]^+$ 235.0877, observed 235.0872.

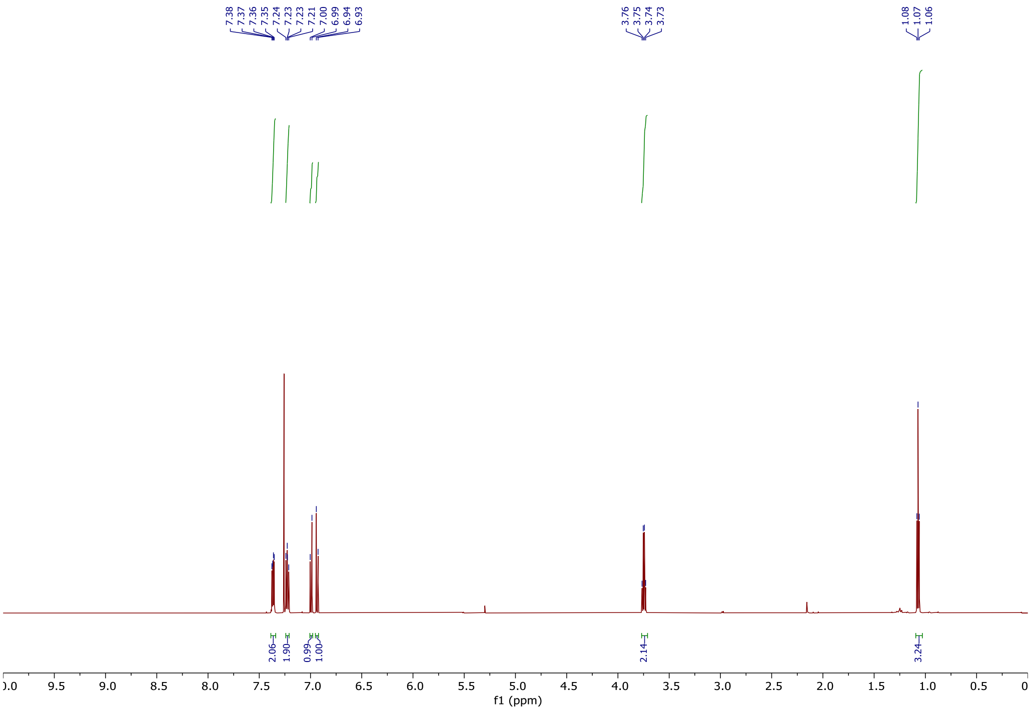

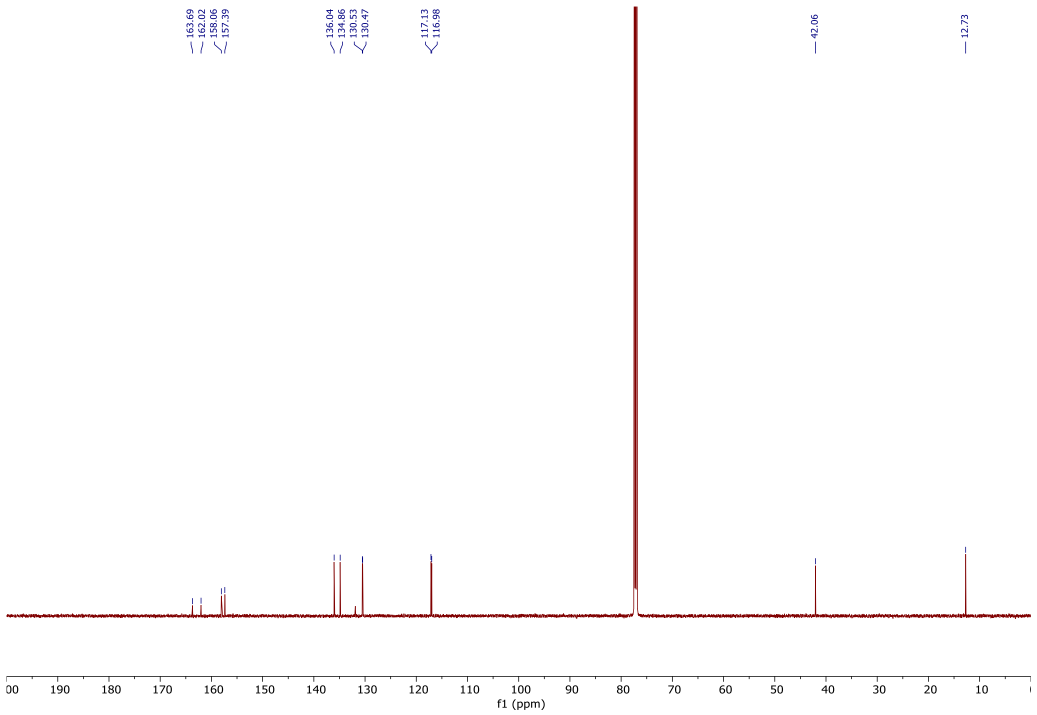
163.69
162.02
158.06
157.39
136.04
134.86
130.53
130.47
117.13
116.98
42.06
12.73
f1 (ppm)

1-Ethyl-2-(perfluorophenyl)hydrazine S3

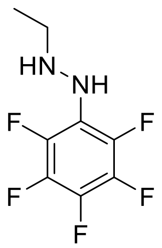

S3

To a solution of pentafluorophenylhydrazine (500 mg, 2.52 mmol) in THF (5 mL) was added a solution of acetaldehyde (0.606 mL, 3.02 mmol, 5 M stock solution in THF) and the reaction was stirred for 30 min at 21 °C. After this time, the solvent and excess acetaldehyde were removed *in vacuo*. The reaction mixture was re-dissolved in THF (10 mL). To this solution was added acetic acid (2 mL) and the solution was cooled to 0 °C. To this cooled solution was added, in small portions, sodium cyanoborohydride (238 mg, 3.79 mmol). Following this, the reaction was stirred for 15 min at 0 °C, then warmed to 21 °C and stirred for 16 h. After this time, THF and AcOH were removed *in vacuo* with toluene co-evaporation (3 × 20 mL, as an azeotrope). Residual toluene was subsequently removed *in vacuo* with chloroform co-evaporation (3 × 20 mL, as an azeotrope). Purification of the crude residue by flash column chromatography (0 to 50% EtOAc/cyclohexane) afforded 1-ethyl-2-(perfluorophenyl)hydrazine as a white powder **S3** (228 mg, 1.01 mmol, 40%).

^{1}H NMR (600 MHz, CD_3OD) δ 3.21-3.15 (m, 2H), 1.38 (t, *J* = 7.2 Hz, 3H); **^{13}C NMR** (150 MHz, CD_3OD) δ 145.2 (C), 143.2 (C), 140.2 (C), 138.1 (C), 120.0 (C), 52.4 (CH_2), 10.3 (CH_3); **IR** (solid) 3325, 3074, 2961, 2841, 1518, 1168 cm^{-1}; **LRMS** (ESI) 227 (100, $[M+H]^+$); **HRMS** (ESI) calcd for $C_8H_8F_5N_2$ $[M+H]^+$ 227.0598, observed 227.0602.

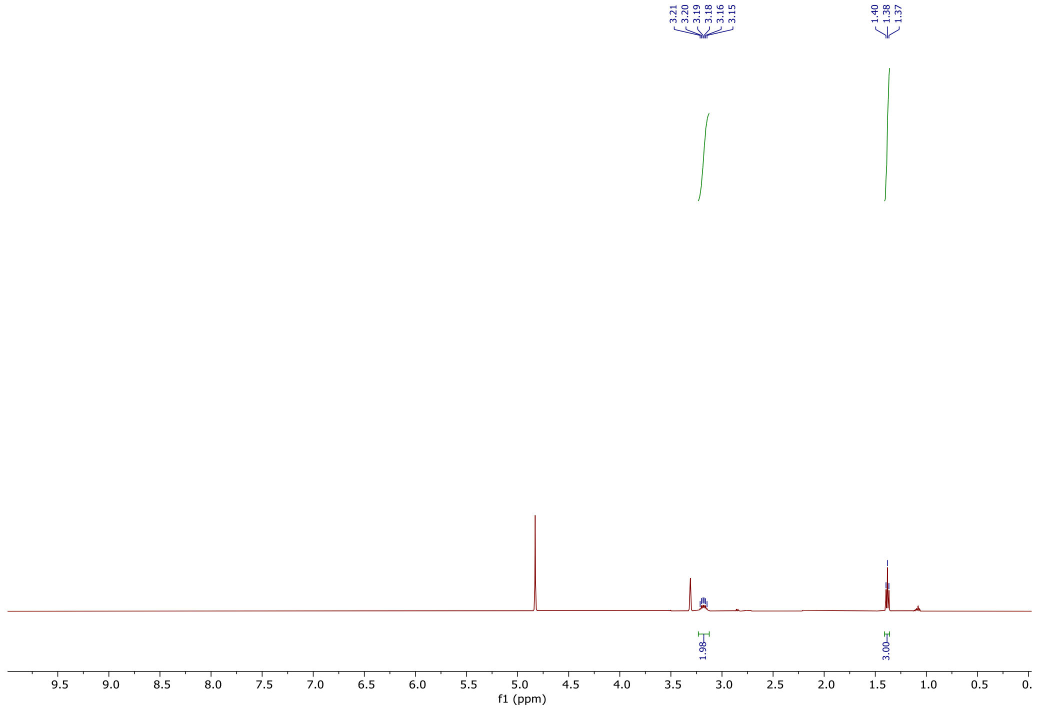

3.21
3.20
3.19
3.18
3.16
3.15
1.40
1.38
1.37
1.98
3.00
9.5 9.0 8.5 8.0 7.5 7.0 6.5 6.0 5.5 5.0 4.5 4.0 3.5 3.0 2.5 2.0 1.5 1.0 0.5
f1 (ppm)

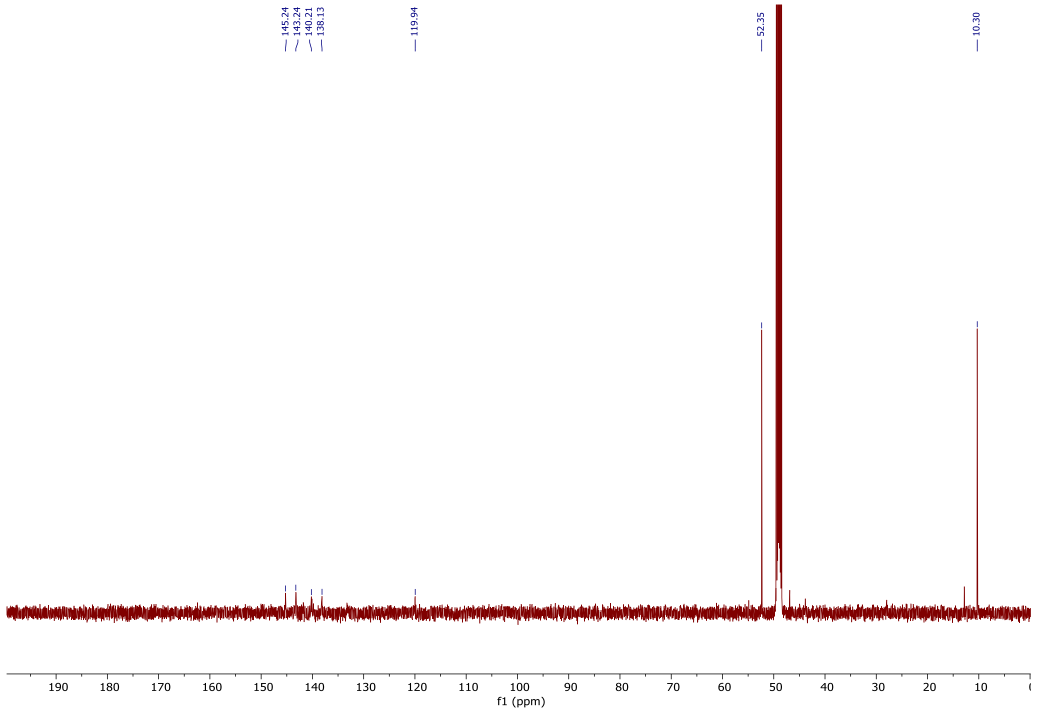

145.24
143.24
140.21
138.13
119.94
52.35
10.30
190 180 170 160 150 140 130 120 110 100 90 80 70 60 50 40 30 20 10
f1 (ppm)

1-Ethyl-2-(perfluorophenyl)-1,2-dihydropyridazine-3,6-dione 6

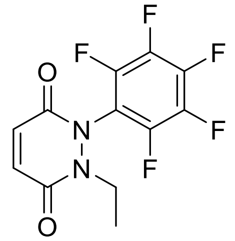

6

To a solution of 1-ethyl-2-(perfluorophenyl)hydrazine **S3** (80 mg, 0.35 mmol) in AcOH (5 mL) was added maleic anhydride (42 mg, 0.42 mmol). The reaction mixture was then heated under reflux with stirring for 16 h. After this time, the reaction mixture was allowed to cool to 21 °C, and the solvent was removed *in vacuo* with toluene co-evaporation (3 x 20 mL, as an azeotrope). Residual toluene was subsequently removed *in vacuo* with chloroform co-evaporation (3 x 20 mL, as an azeotrope). Purification of the crude residue by flash column chromatography (0 to 50% EtOAc/cyclohexane) afforded 1-ethyl-2-(perfluorophenyl)-1,2-dihydropyridazine-3,6-dione **6** as a beige powder (74 mg, 0.24 mmol, 68%).

^{1}H NMR (600 MHz, $CDCl_3$) δ 7.05 (d, *J* = 10.2 Hz, 1H), 6.95 (d, *J* = 10.2 Hz, 1H), 3.75 (q, *J* = 7.1 Hz, 2H), 1.13 (t, *J* = 7.1 Hz, 3H); **^{13}C NMR** (150 MHz, $CDCl_3$) δ 157.3 (C), 156.6 (C), 146.1 (C), 144.1 (C), 142.2 (C), 139.2 (C), 137.4 (CH), 133.5 (CH), 111.1 (C), 42.3 (CH_2), 12.5 (CH_3); **IR** (solid) 3095, 2988, 1649, 1513, 1117 cm^{-1}; **LRMS** (ESI) 307 (100, $[M+H]^+$); **HRMS** (ESI) calcd for $C_{12}H_8F_5N_2O_2$ $[M+H]^+$ 307.5001, observed 307.4995.

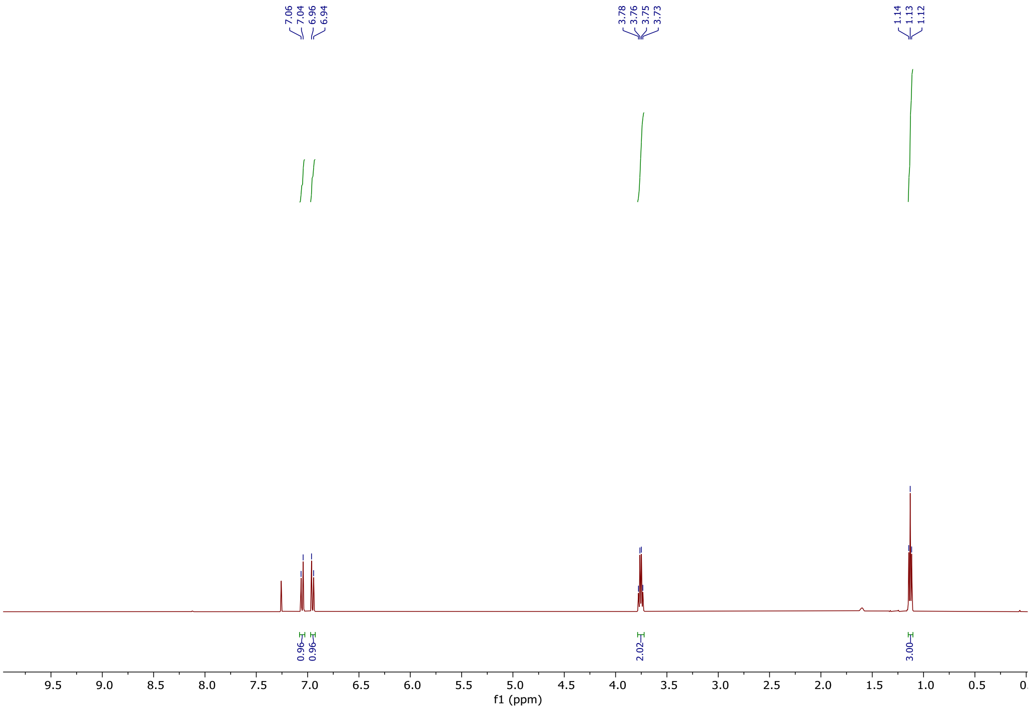

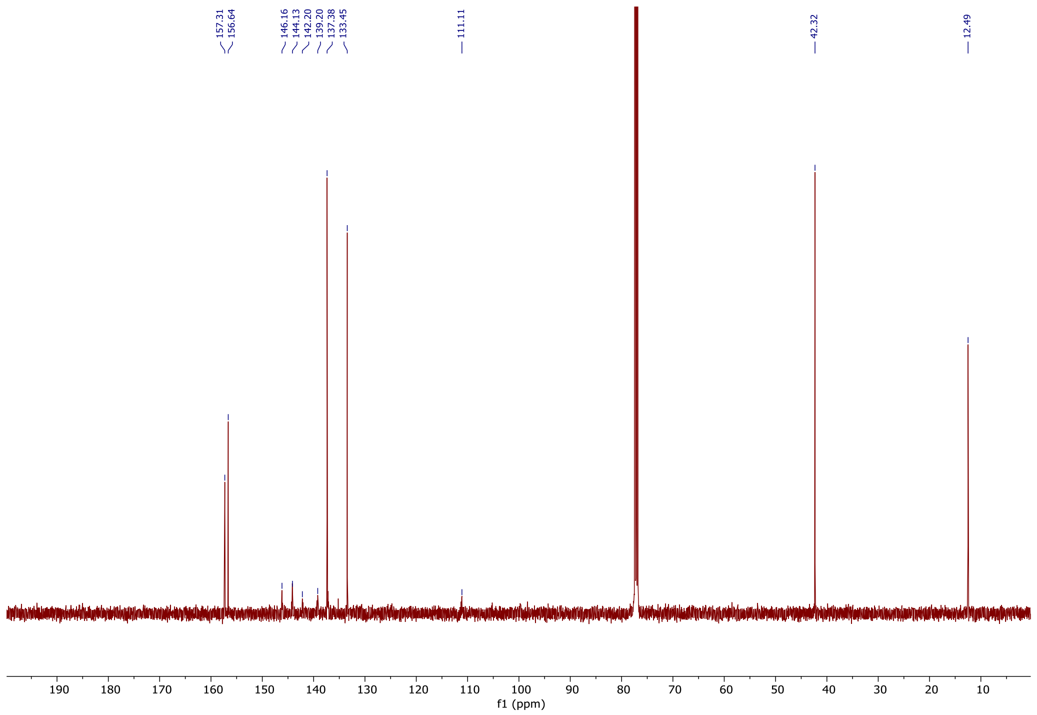
157.31
156.64
146.16
144.13
142.20
139.20
137.38
133.45
111.11
42.32
12.49
190
180
170
160
150
140
130
120
110
100
90
80
70
60
50
40
30
20
10
f1 (ppm)

1-Ethyl-2-(4-nitrophenyl)-1,2-dihydropyridazine-3,6-dione 5

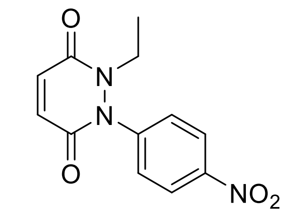

5

To a solution of 1-ethyl-2-phenyl-1,2-dihydropyridazine-3,6-dione **3** (400 mg, 0.35 mmol) in sulfuric acid (2 mL), cooled to 0 °C, was added a solution of sodium nitrate (29.8 mg, 0.35 mmol) in sulfuric acid (1 mL). The reaction mixture was then stirred for 16 h at 0 °C. After this time, the reaction mixture was diluted by the slow addition of water (70 mL). The product was then extracted into EtOAc (3 x 10 mL), the organics combined and dried over $MgSO_4$, and the EtOAc was removed *in vacuo*. Purification of the crude residue by silica gel chromatography (50 to 70% EtOAc/cyclohexane) afforded 1-ethyl-2-(4-nitrophenyl)-1,2-dihydropyridazine-3,6-dione **5** as a yellow solid (230 mg, 0.88 mmol, 47%).

^{1}H NMR (600 MHz, $CDCl_3$) δ 8.39 (d, *J* = 9.0 Hz, 2H), 7.61 (d, *J* = 9.0 Hz, 2H), 7.02 (d, *J* = 10.1 Hz, 1H), 6.93 (d, *J* = 10.1 Hz, 1H), 3.77 (q, *J* = 7.0 Hz, 2H), 1.08 (t, *J* = 7.0 Hz, 3H); **^{13}C NMR** (150 MHz, $CDCl_3$) δ 158.3 (C), 158.0 (C), 147.4 (C), 141.6 (C), 136.5 (CH), 134.8 (CH), 128.5 (CH), 125.0 (CH), 43.0 (CH_2), 12.5 (CH_3); **IR** (solid) 3047, 2952, 1638, 1591, 1523 cm^{-1}; **LRMS** (ESI) 262 (100, $[M+H]^+$); **HRMS** (ESI) calcd for $C_{12}H_{12}N_3O_4$ $[M+H]^+$ 262.0822, observed 262.0816.

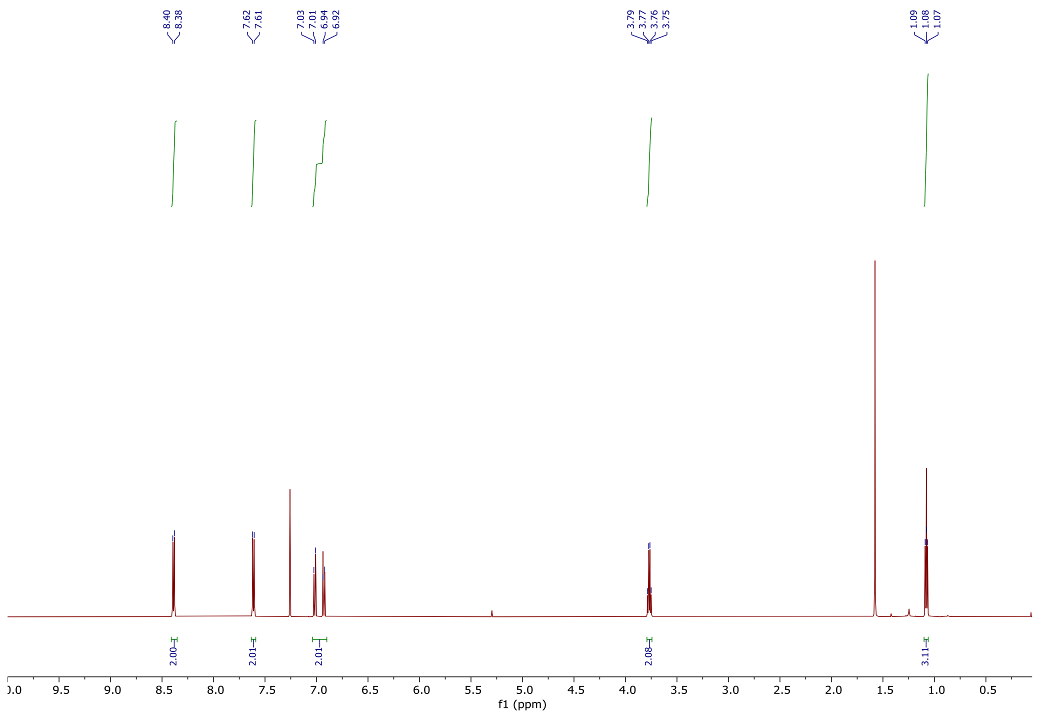

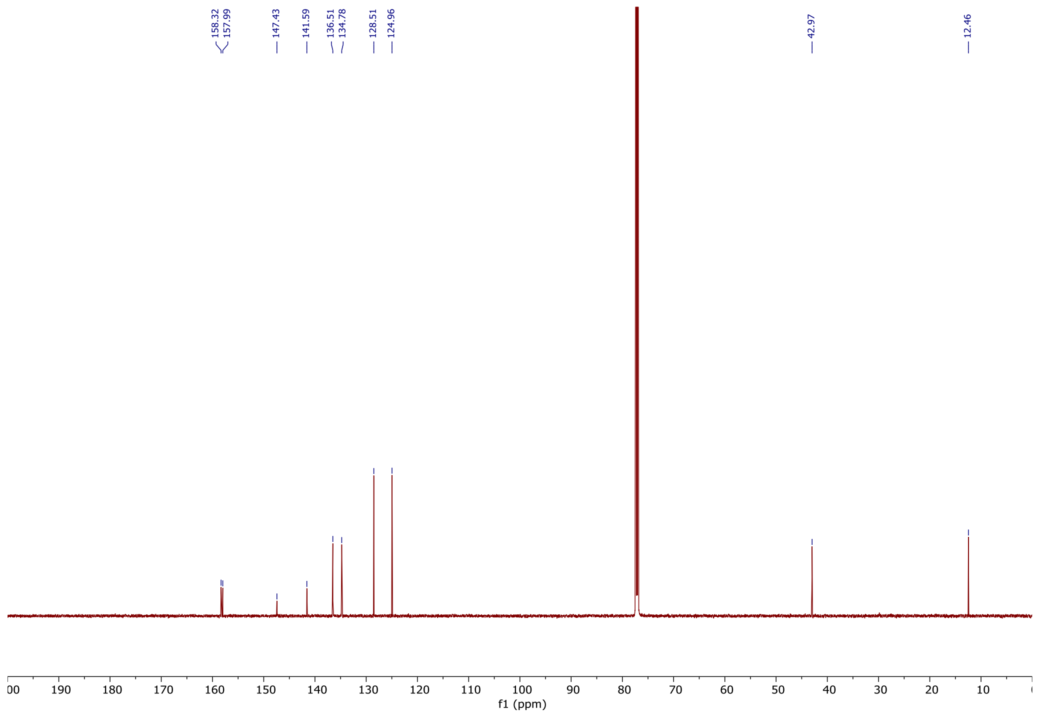
158.32
157.99
147.43
141.59
136.51
134.78
128.51
124.96
42.97
12.46
f1 (ppm)

1-(4-Aminophenyl)-2-ethyl-1,2-dihydropyridazine-3,6-dione 2

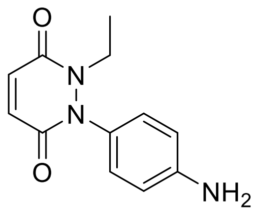

2

To a solution of 1-ethyl-2-(4-nitrophenyl)-1,2-dihydropyridazine-3,6-dione **5** (100 mg, 0.383 mmol) in EtOH/H_2O (3:1 v/v, 8 mL) was added iron powder (107 mg, 1.91 mmol) and ammonium chloride (61.4 mg, 1.15 mmol). The reaction mixture was then heated under reflux for 4 h. After this time, the iron was removed by filtering through celite and the filter cake washed with water (20 mL). The product was then extracted from the filtrate into EtOAc (3 × 10 mL), dried over $MgSO_4$ and the EtOAc was removed *in vacuo*. Purification of the crude residue by flash column chromatography (80 to 100% EtOAc/cyclohexane) afforded 1-(4-aminophenyl)-2-ethyl-1,2-dihydropyridazine-3,6-dione **2** as a brown powder (74 mg, 0.32 mmol, 84%).

^{1}H NMR (600 MHz, $CDCl_3$) δ 7.11 (d, *J* = 8.8 Hz, 2H), 6.97 (d, *J* = 10.1 Hz, 1H), 6.93 (d, *J* = 10.1 Hz, 1H), 6.76 (d, *J* = 8.8 Hz, 2H), 3.94 (s, 2H), 3.78 (q, *J* = 7.0 Hz, 2H), 1.07 (t, *J* = 7.0 Hz, 3H); **^{13}C NMR** (150 MHz, $CDCl_3$) δ 158.1 (C), 157.0 (C), 148.0 (C), 135.6 (CH), 135.0 (CH), 129.9 (CH), 125.8 (C), 115.6 (CH), 41.6 (CH_2), 12.9 (CH_3); **IR** (solid) 3448, 3364, 3063, 2921, 1618, 1600, 1581 cm^{-1}; **LRMS** (ESI) 232 (100, $[M+H]^+$); **HRMS** (ESI) calcd for $C_{12}H_{14}N_3O_2$ $[M+H]^+$ 232.1081, observed 232.1078.

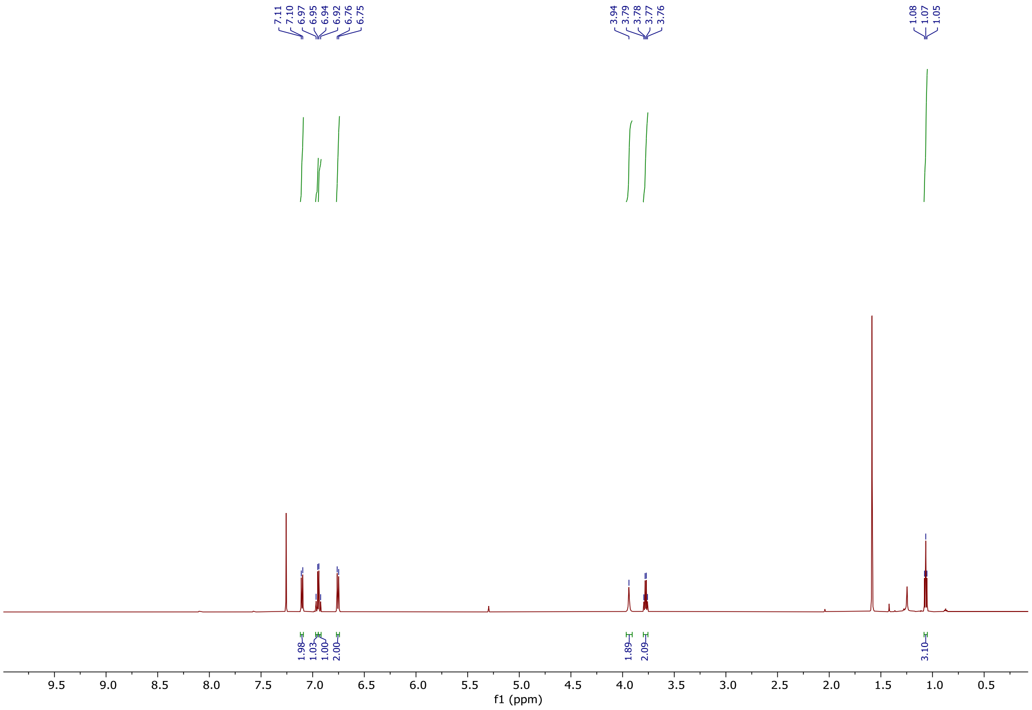

7.11
7.10
6.97
6.95
6.94
6.92
6.76
6.75
3.94
3.79
3.78
3.77
3.76
1.08
1.07
1.05
1.98
1.03
1.00
2.00
1.89
2.09
3.10
f1 (ppm)

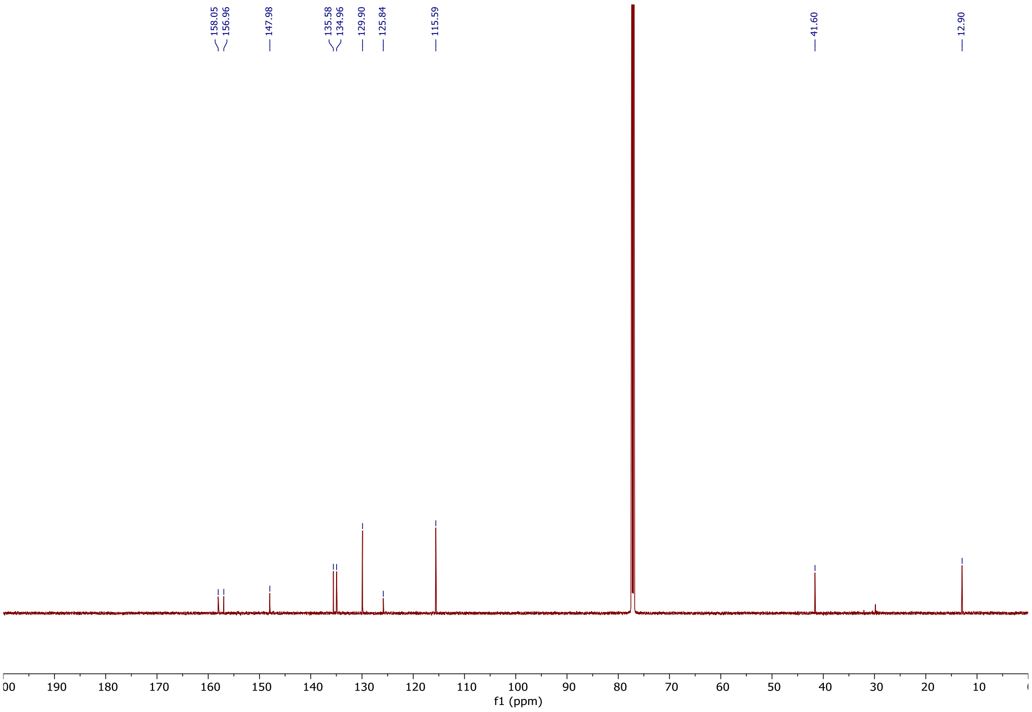

158.05
156.96
147.98
135.58
134.96
129.90
125.84
115.59
41.60
12.90
f1 (ppm)

2.2 PD-cysteine small molecule model conjugates

Methyl *N*-(*tert*-butoxycarbonyl)-*S*-(2-ethyl-3,6-dioxo-1-phenylhexahydropyridazin-4-yl)-*L*-cysteinate 10/ Methyl *N*-(*tert*-butoxycarbonyl)-*S*-(1-ethyl-3,6-dioxo-2-phenylhexahydropyridazin-4-yl)-*L*-cysteinate 10'

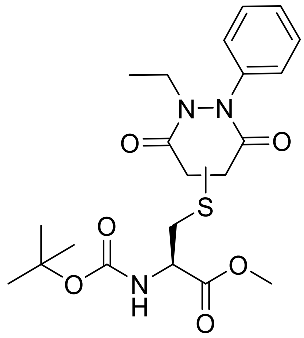

10/10'

To a solution of *N*-(*tert*-butoxycarbonyl)-*L*-cysteine methyl ester **7** (19 µL, 22 mg, 0.093 mmol) in MeOH (0.5 mL) was added 1-ethyl-2-phenyl-1,2-dihydropyridazine-3,6-dione **3** (20 mg, 0.093 mmol) and sodium acetate (23 mg, 0.28 mmol). The reaction mixture was then stirred at 21 °C for 1 h. After this time, the solvent was removed *in vacuo*, and the crude residue was purified by flash column chromatography (20% to 100% EtOAc/cyclohexane) to give methyl *N*-(*tert*-butoxycarbonyl)-*S*-(2-ethyl-3,6-dioxo-1-phenylhexahydropyridazin-4-yl)-*L*-cysteinate **10** and methyl *N*-(*tert*-butoxycarbonyl)-*S*-(1-ethyl-3,6-dioxo-2-phenylhexahydropyridazin-4-yl)-*L*-cysteinate **10'** as a colourless oil (22 mg, 0.048 mmol, 52%) as a mixture of regioisomers and diastereoisomers.

^{1}H NMR (600 MHz, $CDCl_3$, regioisomers, diastereomers, rotamers) δ 7.45-7.42 (m, 2H), 7.39-7.36 (m, 2H), 7.32 (t, *J* = 7.4 Hz, 1H), 5.59-5.43 (m, 1H), 4.66-4.61 (m, 1H), 4.02-3.96 (m, 1H), 3.88-3.81 (m, 1H), 3.78-3.74 (m, 3H), 3.33-3.30 (m, 0.5H), 3.22-3.20 (m, 1H), 3.16-3.12 (dt, *J* = 16.0, 4.4 Hz, 1H), 3.07-3.02 (m, 1.3H), 2.80-2.75 (dt, *J* = 16.1, 4.6 Hz, 1H), 1.45 (m, 9H), 1.08-1.06 (t, *J* = 7.1 Hz, 3H); **^{13}C NMR** (150 MHz, $CDCl_3$, regioisomers, diastereomers, rotamers) δ 171.4 (C), 167.1 (C), 165.9 (C), 155.4 (C), 136.5 (C), 129.3 (CH), 128.0 (CH), 125.5 (CH), 80.5 (C), 53.8 (CH), 53.0 (CH), 41.4 (CH), 41.3 (CH), 39.9 (CH_2), 35.9 (CH_2), 34.5 (CH_2), 34.0 (CH_2), 28.4 (CH_3), 12.1 (CH_3), 11.5 (CH_3); **IR** (thin film) 3351, 2977, 2933, 1667, 1594 cm^{-1}; **LRMS** (ESI) 450 (100, $[M-H]^-$); **HRMS** (ESI) calcd for $C_{21}H_{28}N_3O_6S$ $[M-H]^-$ 450.1704, observed 450.1702.

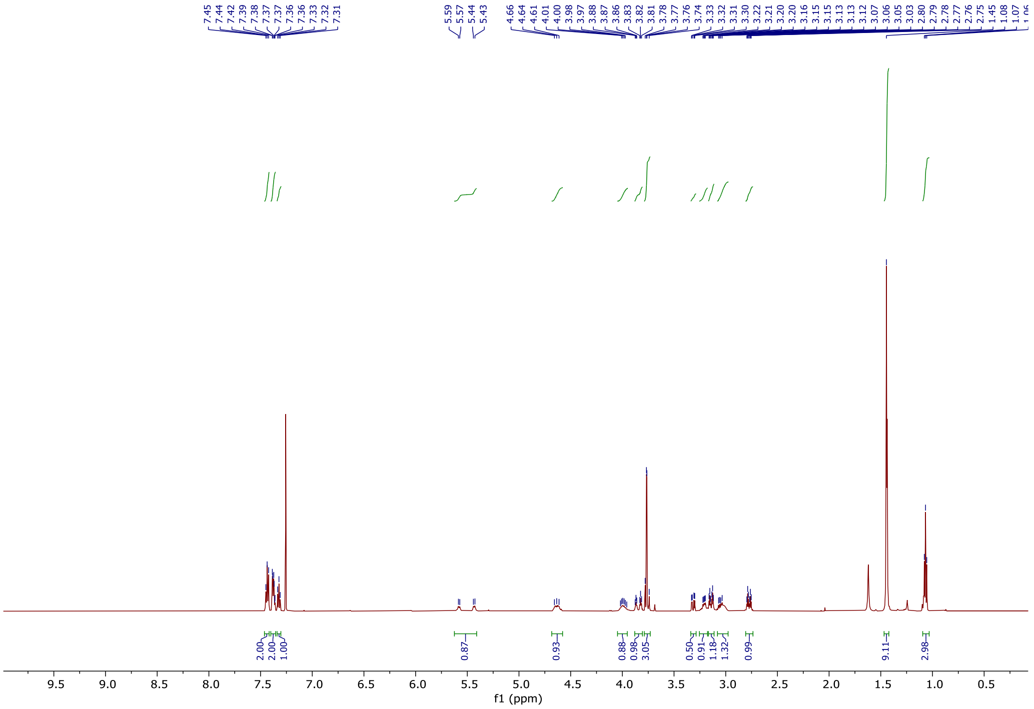

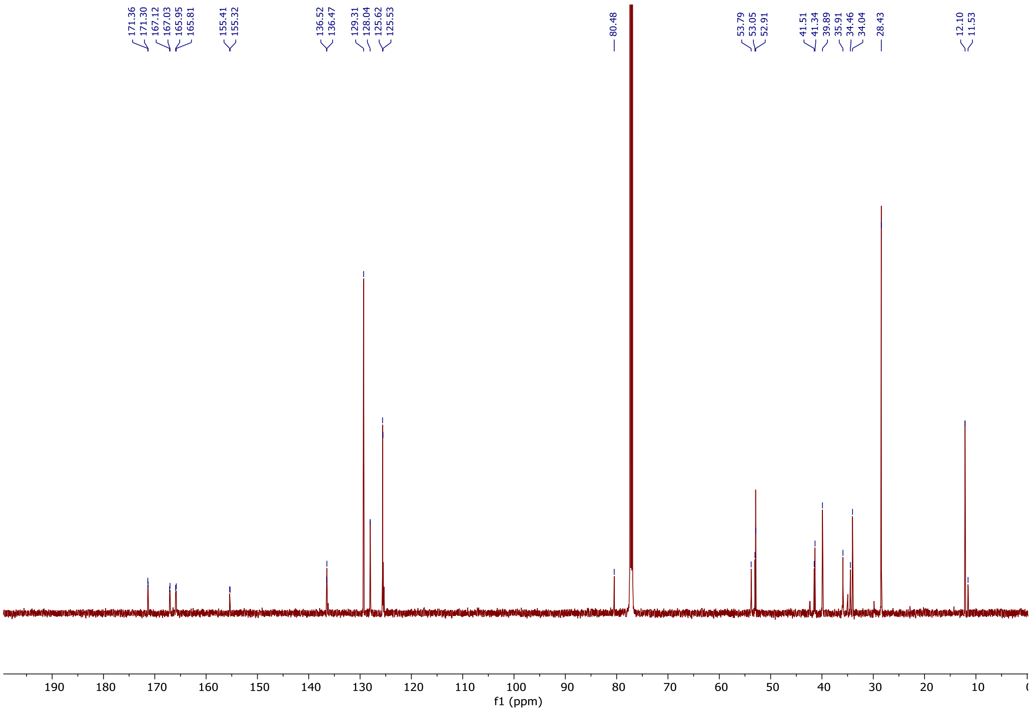

Methyl *N*-(*tert*-butoxycarbonyl)-*S*-(2-ethyl-1-(4-fluorophenyl)-3,6-dioxohexahydropyridazin-4-yl)-*L*-cysteinate 11/ Methyl *N*-(*tert*-butoxycarbonyl)-*S*-(1-ethyl-2-(4-fluorophenyl)-3,6-dioxohexahydropyridazin-4-yl)-*L*-cysteinate 11′

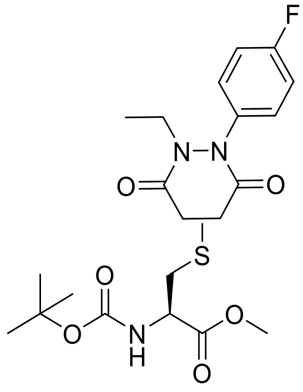

11/11'

To a solution of *N*-(*tert*-butoxycarbonyl)-*L*-cysteine methyl ester **7** (18 µL, 21 mg, 0.085 mmol) in MeOH (0.5 mL) was added 1-ethyl-2-(4-fluorophenyl)-1,2-dihydropyridazine-3,6-dione **4** (20 mg, 0.085 mmol) and sodium acetate (21 mg, 0.26 mmol). The reaction mixture was then stirred at 21 °C for 1 h. After this time, the solvent was removed *in vacuo*, and the crude residue was purified by flash column chromatography (20% to 100% EtOAc/cyclohexane) to give methyl *N*-(*tert*-butoxycarbonyl)-*S*-(2-ethyl-1-(4-fluorophenyl)-3,6-dioxohexahydropyridazin-4-yl)-*L*-cysteinate **11** and methyl *N*-(*tert*-butoxycarbonyl)-*S*-(1-ethyl-2-(4-fluorophenyl)-3,6-dioxohexahydropyridazin-4-yl)-*L*-cysteinate **11′** as a colourless oil (13 mg, 0.027 mmol, 32%) as a mixture of regioisomers and diastereoisomers.

^{1}H NMR (500 MHz, $CDCl_3$, diastereomers, rotamers, regioisomers) δ 7.38-7.35 (m, 2H), 7.13-7.10 (m, 2H), 5.56-5.41 (m, 1H), 4.62-4.60 (m, 1H), 4.03-4.00 (m, 1H), 3.86-3.85 (m, 0.4H), 3.82-3.80 (m, 0.5H), 3.77-3.76 (m, 3H), 3.34-3.30 (m, 0.4H), 3.20 (br s, 1H), 3.16-3.11 (dt, *J* = 16.1, 4.8 Hz, 1H), 3.05-3.01 (m, 0.4H), 2.99-2.95, (m, 1H), 2.79-2.74 (dt, *J* = 16.1, 5.0 Hz, 1H), 1.44-1.43 (m, 9H), 1.07-1.05 (t, *J* = 7.1 Hz, 3H), 1.13-1.11 (t, *J* = 7.1 Hz, 3H); **^{13}C NMR** (150 MHz, $CDCl_3$, diastereomers, rotamers, regioisomers) δ 171.3 (C), 171.3 (C), 167.0 (C), 166.9 (C), 166.1 (C), 165.9 (C), 162.6 (C), 160.9 (C), 155.4 (C), 155.3 (C), 132.5 (CH), 132.4 (CH), 127.7 (CH), 127.6 (CH), 127.6 (CH), 116.4 (CH), 116.2 (CH), 80.6 (C), 53.8 (CH), 53.0 (CH_3), 52.9 (CH_3), 41.4 (CH), 41.2 (CH), 39.9 (CH_2), 39.8 (CH_2), 35.8 (CH_2), 35.7 (CH_2), 34.5 (CH_2), 34.1 (CH_2), 28.4 (CH_3), 12.1 (CH_3); **IR** (thin film) 3349, 2976, 2927, 1699, 1595, 1152 cm^{-1}; **LRMS** (ESI) 492 (20, $[M+Na]^+$, 370 (80, $[M\text{-}Boc+H]^+$); **HRMS** (ESI) calcd for $C_{21}H_{29}FN_3O_6S$ $[M+H]^+$ 470.1761, observed 470.1748.

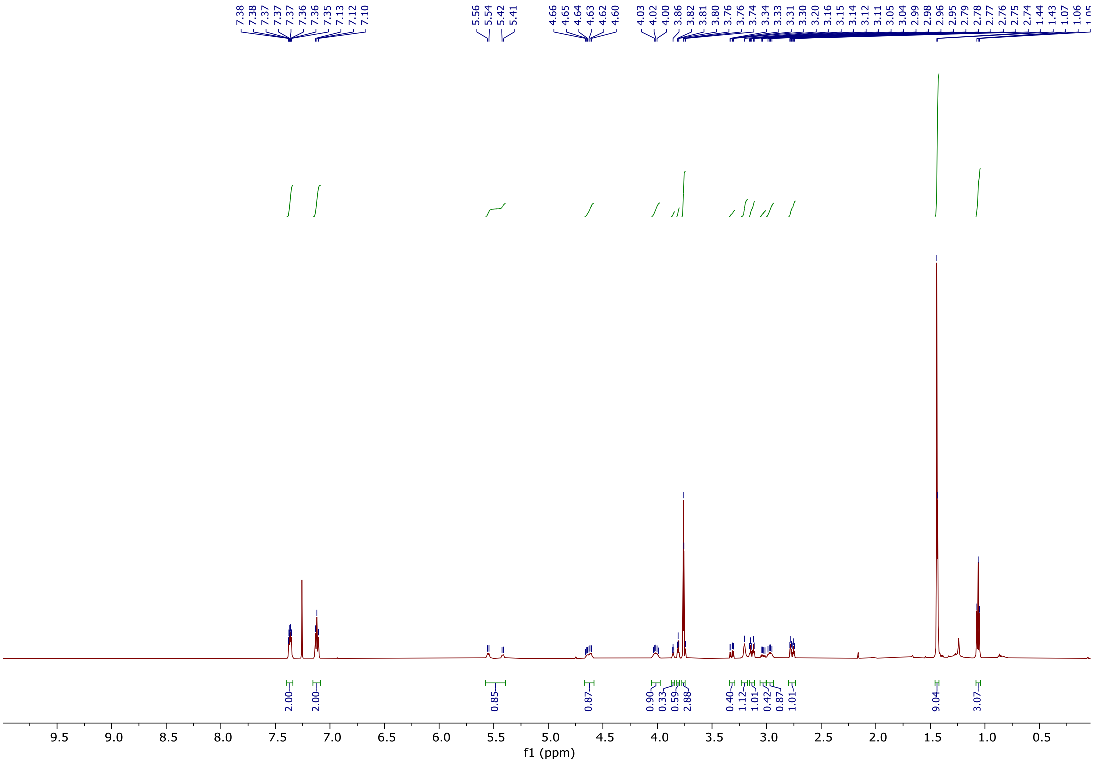

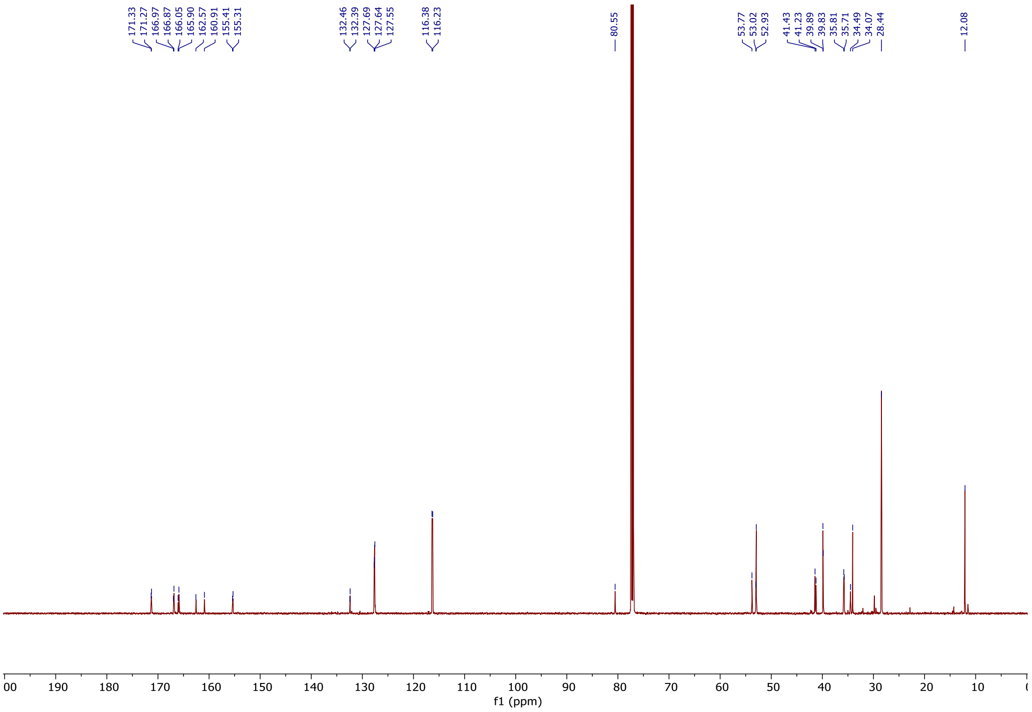

Methyl *N*-(*tert*-butoxycarbonyl)-*S*-(2-ethyl-3,6-dioxo-1-(perfluorophenyl)hexahydropyridazin-4-yl)-*L*-cysteinate 13

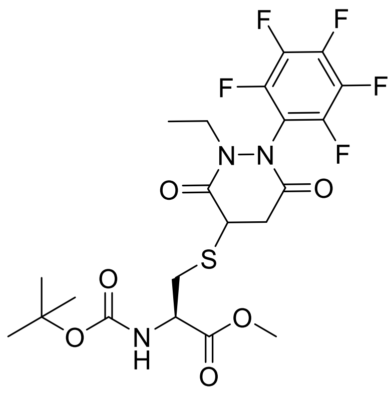

13

To a solution of *N*-(*tert*-butoxycarbonyl)-L-cysteine methyl ester **7** (13.4 µL, 15 mg, 0.065 mmol) in MeOH (0.5 mL) was added 1-ethyl-2-(perfluorophenyl)-1,2-dihydropyridazine-3,6-dione **6** (20 mg, 0.065 mmol) and sodium acetate (16 mg, 0.195 mmol). The reaction mixture was then stirred at 21 °C for 1 h. After this time, the solvent was removed *in vacuo*, and the crude residue was purified by flash column chromatography (20% to 100% EtOAc/cyclohexane) to give methyl *N*-(*tert*-butoxycarbonyl)-*S*-(2-ethyl-3,6-dioxo-1-(perfluorophenyl)hexahydropyridazin-4-yl)-*L*-cysteinate **13** as a colourless oil (8 mg, 0.014 mmol, 21%) as a mixture of diastereoisomers.

^{1}H NMR (500 MHz, $CDCl_3$, diastereomers, rotamers) δ 5.57-5.44 (m, 1H), 4.62-4.55 (m, 1H), 4.10-4.09 (m, 2H), 3.77-3.75 (m, 3H), 3.74-3.73 (m, 0.5 H), 3.71-3.69 (m, 0.5 H) 3.40-3.39 (m, 2H), 3.21-3.18 (m, 0.5 H), 3.14-3.07, (m, 1H), 2.98-2.96, (m, 0.5 H), 2.93-2.89 (m, 1H), 2.62-2.58 (m, 1H), 1.45-1.44 (m, 9H), 1.16-1.15 (m, 3H), 1.13-1.11 (t, *J* = 7.0 Hz, 3H); **^{13}C NMR** (150 MHz, $CDCl_3$, diastereomers, rotamers) δ 171.4 (C), 171.3 (C), 167.5 (C), 167.3 (C), 166.4 (C), 166.3 (C), 155.4 (C), 155.3 (C), 80.4 (C), 53.7 (CH), 52.9 (CH), 52.9 (CH_3), 41.4 (CH), 41.3 (CH), 38.7 (CH_2), 38.7 (CH_2), 38.6 (CH_2), 35.0 (CH_2), 35.0 (CH_2), 34.2 (CH_2), 33.8 (CH_2), 28.4 (CH_3), 12.2 (CH_3), 11.7 (CH_3), 11.7 (CH_3); **IR** (thin film) 3352, 2979, 2934, 1709, 1692, 1515, 1162 cm^{-1}; **LRMS** (ESI) 442 (100, $[M\text{-}Boc+H]^+$); **HRMS** (ESI) calcd for $C_{21}H_{24}F_5N_3O_6S$ $[M+H]^+$ 542.1379, observed 542.1372.

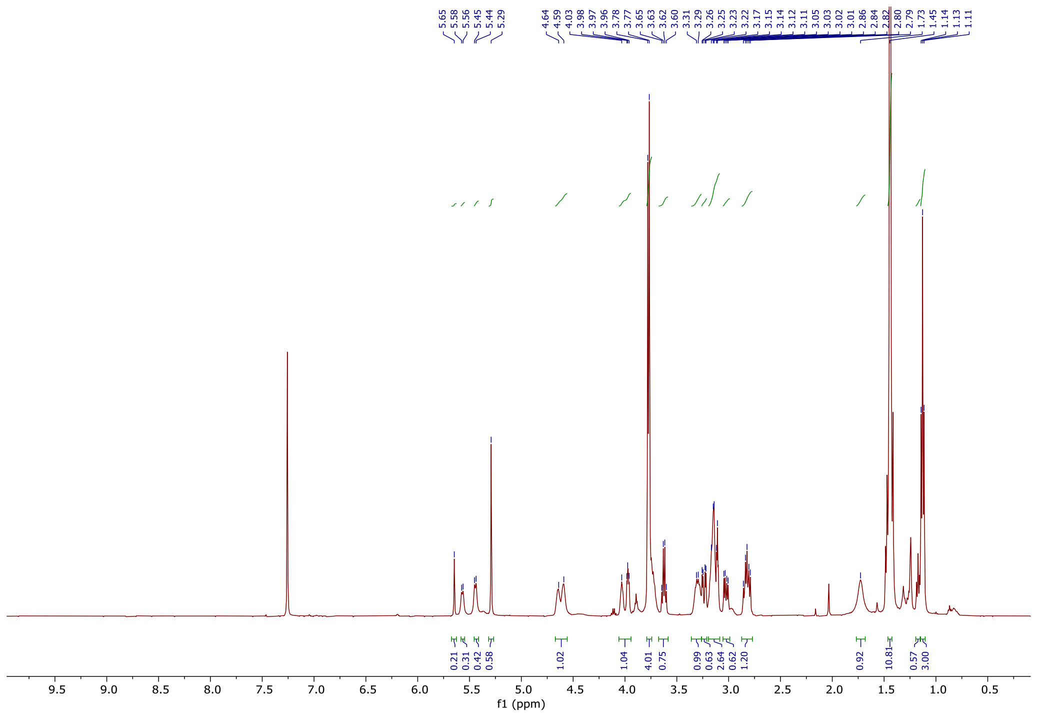
5.65 5.58 5.56 5.45 5.44 5.29 4.64 4.59 4.03 3.98 3.97 3.96 3.78 3.77 3.65 3.63 3.62 3.60 3.31 3.29 3.26 3.25 3.23 3.22 3.17 3.15 3.14 3.12 3.11 3.05 3.03 3.02 3.01 2.86 2.84 2.82 2.80 2.79 1.73 1.45 1.14 1.13 1.11
0.21 0.31 0.42 0.58 1.02 1.04 4.01 0.75 0.99 0.63 2.64 0.62 1.20 0.92 10.81 0.57 3.00
f1 (ppm)

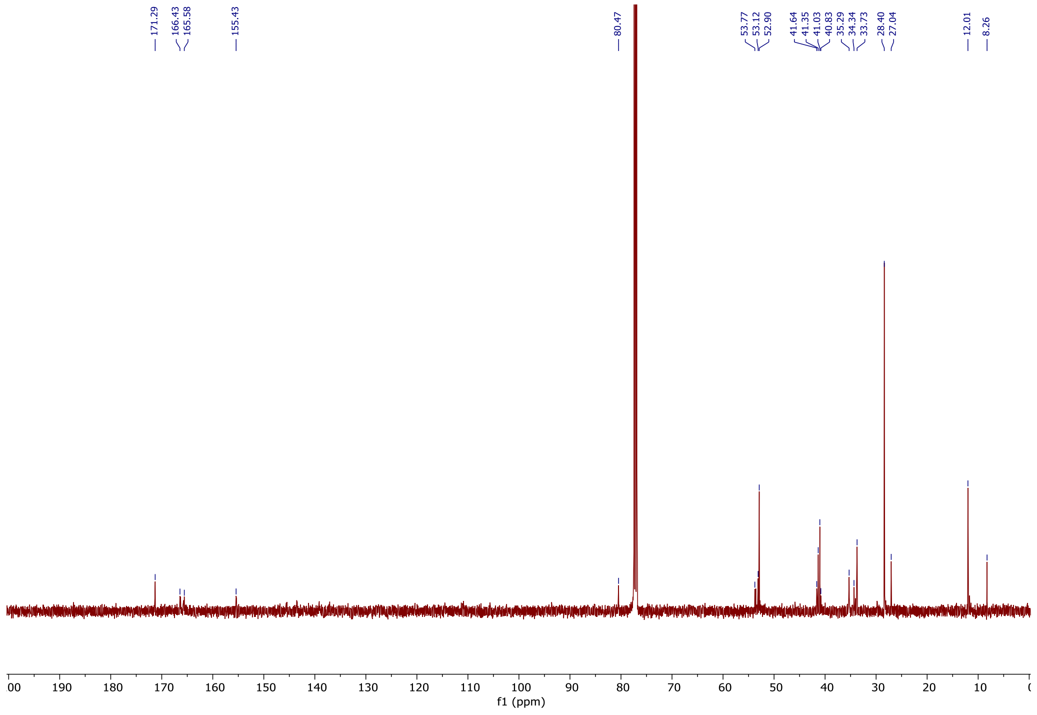
171.29 166.43 165.58 155.43 80.47 53.77 53.12 52.90 41.64 41.35 41.03 40.83 35.29 34.34 33.73 28.40 27.04 12.01 8.26
f1 (ppm)

Methyl *N*-(*tert*-butoxycarbonyl)-*S*-(2-ethyl-1-(4-nitrophenyl)-3,6-dioxohexahydropyridazin-4-yl)-*L*-cysteinate 12

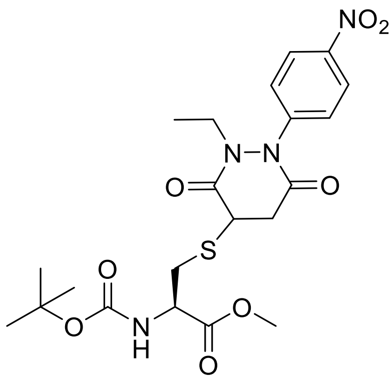

12

To a solution of *N*-(tert-butoxycarbonyl)-*L*-cysteine methyl ester **7** (16 µL, 18 mg, 0.077 mmol) in MeOH (0.5 mL) was added 1-ethyl-2-(4-nitrophenyl)-1,2-dihydropyridazine-3,6-dione **5** (20 mg, 0.077 mmol) and sodium acetate (19 mg, 0.23 mmol). The reaction mixture was then stirred at 21 °C for 1 h. After this time, the solvent was removed *in vacuo*, and the crude residue was purified by flash column chromatography (20% to 100% EtOAc/cyclohexane) to give methyl *N*-(*tert*-butoxycarbonyl)-*S*-(2-ethyl-1-(4-nitrophenyl)-3,6-dioxohexahydropyridazin-4-yl)-*L*-cysteinate **12** as a yellow oil (11 mg, 0.021 mmol, 28%) as a mixture of diastereoisomers.

^{1}H NMR (500 MHz, CD_3CN, diastereomers, rotamers) δ 8.29-8.27 (d, J = 9.3 Hz, 2H), 7.65-7.63 (d, J = 9.3 Hz, 2H), 5.99-5.84 (br d, *J* = ~8 Hz, 1H), 4.49-4.40 (td, *J* = 8.0, 4.6 Hz, 1H), 3.98-3.97 (m, 1H), 3.92-3.91 (t, *J* = 4.4 Hz, 1H), 3.69 (s, 3H), 3.35-3.31 (dd, *J* = 4.2, 16.3 Hz, 1H), 3.26-3.15 (dd, *J* = 14.0, 4.8 Hz, 1H), 3.08-2.93 (dd, *J* = 14.0, 8.1 Hz, 2H), 2.75-2.67 (dd, *J* = 4.6, 16.3 Hz, 1H), 1.41-1.40 (m, 9H), 1.06-1.03 (t, *J* = 7.1 Hz, 3H); **^{13}C NMR** (150 MHz, CD_3CN, diastereomers, rotamers) δ 172.2 (C), 168.7 (C), 167.8 (C), 146.8 (C), 143.4 (C), 125.8 (CH), 125.3 (CH), 80.4 (C), 54.8 (CH), 53.8 (CH), 53.2 (CH_3), 53.1 (CH_2), 42.2 (CH), 41.6 (CH), 41.5 (CH_2), 41.5 (CH_2), 36.8 (CH_2), 36.5 (CH_2), 34.0 (CH_2), 28.5 (CH_3), 12.2 (CH_3); **IR** (thin film) 3337, 2921, 2851, 1678, 1592, 1518, 1327 cm^{-1}; **LRMS** (ESI) 495 (100, $[M-H]^-$); **HRMS** (ESI) calcd for $C_{21}H_{27}N_4O_8S$ $[M-H]^-$ 495.1555, observed 495.1551.

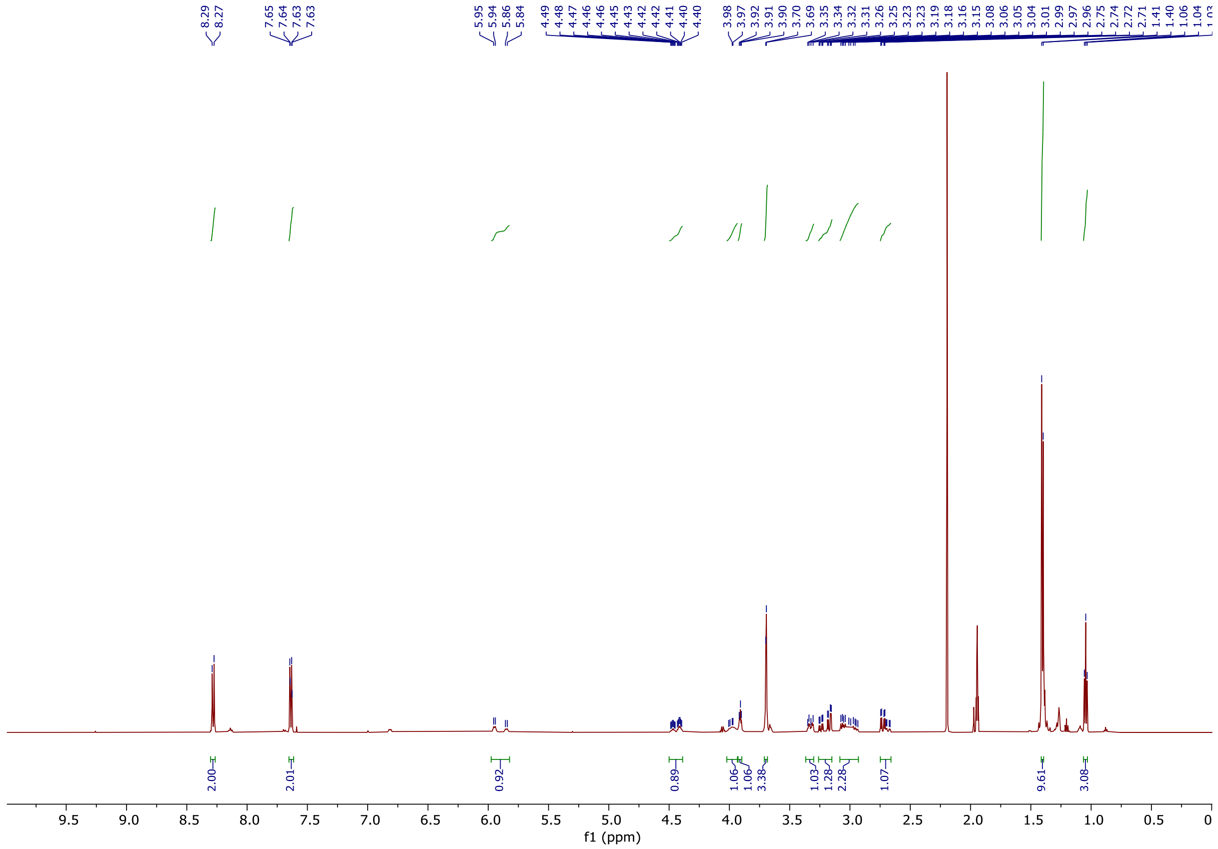

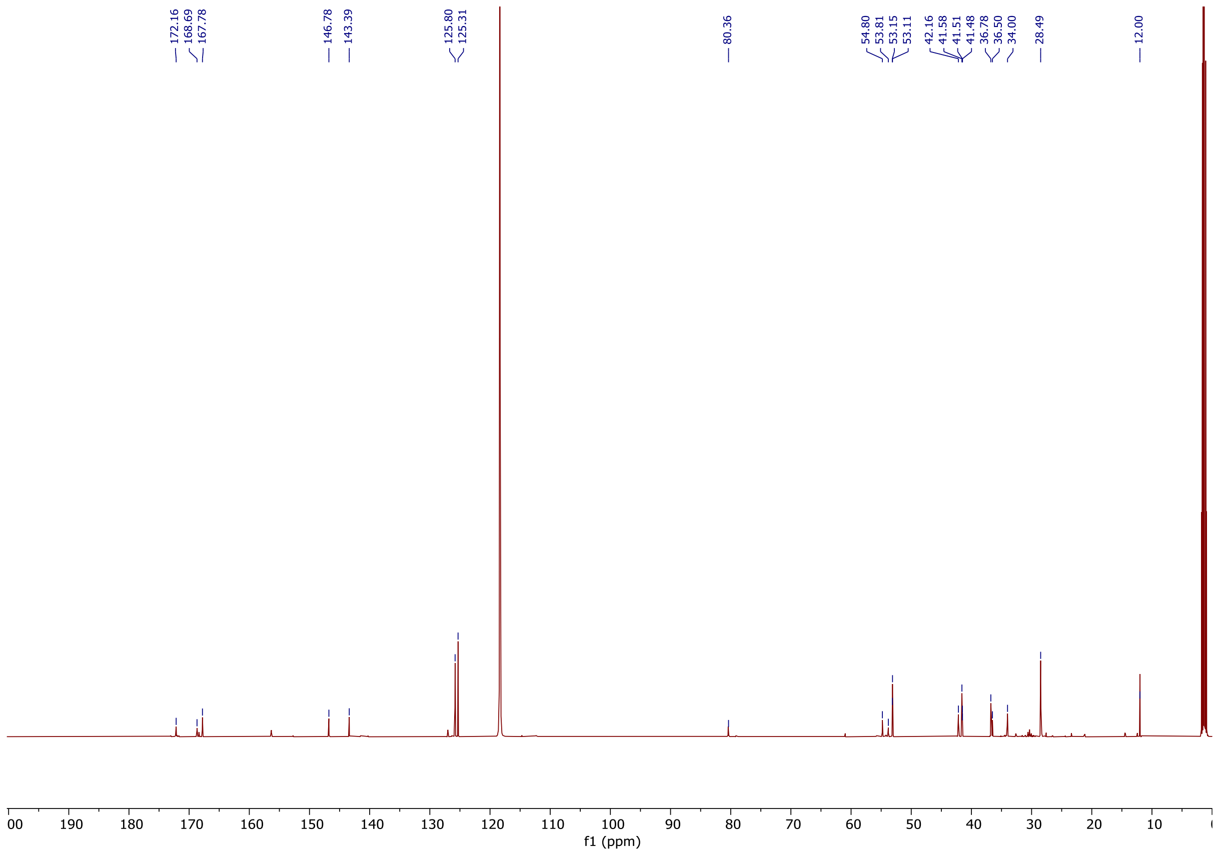

Methyl S-(2-(4-aminophenyl)-1-ethyl-3,6-dioxohexahydropyridazin-4-yl)-N-(*tert*-butoxycarbonyl)-L-cysteinate 9/ Methyl S-(1-(4-aminophenyl)-2-ethyl-3,6-dioxohexahydropyridazin-4-yl)-N-(*tert*-butoxycarbonyl)-L-cysteinate 9'

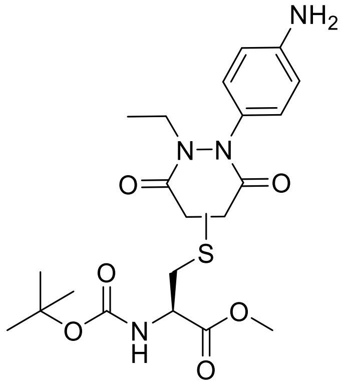

9/9'

To a solution of *N*-(*tert*-butoxycarbonyl)-*L*-cysteine methyl ester **7** (18 µL, 21 mg, 0.087 mmol) in MeOH (0.5 mL) was added 1-(4-aminophenyl)-2-ethyl-1,2-dihydropyridazine-3,6-dione **2** (20 mg, 0.087 mmol) and sodium acetate (21 mg, 0.195 mmol). The reaction mixture was then stirred at 21 °C for 1 h. After this time, the solvent was removed *in vacuo*, and the crude residue was purified by flash column chromatography (20% to 100% EtOAc/cyclohexane) to give methyl *S*-(2-(4-aminophenyl)-1-ethyl-3,6-dioxohexahydropyridazin-4-yl)-*N*-(*tert*-butoxycarbonyl)-*L*-cysteinate **9** and methyl *S*-(1-(4-aminophenyl)-2-ethyl-3,6-dioxohexahydropyridazin-4-yl)-*N*-(*tert*-butoxycarbonyl)-*L*-cysteinate **9'** as a yellow oil (19 mg, 0.041 mmol, 47%) as a mixture of regioisomers and diastereoisomers.

^{1}H NMR (500 MHz, $CDCl_3$, diastereomers, rotamers, regioisomers) δ 7.16-7.1 (m, 2H), 6.73-6.72 (m, 1H), 5.53 (br d, *J* = ~8 Hz, 1H), 4.61 (br. td, *J* = ~8, ~5 Hz, 1H), 3.94-3.89 (m, 1H), 3.84-3.80 (t, *J* = 4.4 Hz, 1H), 3.77 (s, 3H), 3.33-3.27 (dd, *J* = 13.9, 4.5 Hz, 1H) 3.21-3.18 (m, 1H), 3.13-3.09 (dd, *J* = 4.5, 16.1 Hz, 1H), 3.08-3.03 (dd, *J* = 13.9, 6.6 Hz, 1H), 2.76-2.71 (dd, *J* = 4.5, 16.1 Hz, 1H), 1.45-1.44 (m, 9H), 1.07-1.04 (t, *J* = 7.1 Hz, 3H); **^{13}C NMR** (150 MHz, $CDCl_3$, diastereomers, rotamers, regioisomers) δ 171.4 (C), 166.6 (C), 165.8 (C), 155.4 (C), 145.9 (C), 127.6 (CH), 115.8 (CH), 80.5 (C), 53.9 (CH), 53.1 (CH), 52.9 (CH_3), 41.6 (CH) 41.4 (CH), 39.5 (CH_2), 35.7 (CH_2), 34.0 (CH_2), 29.8 (CH_2), 28.4 (CH_3), 12.2 (CH_3); **IR** (thin film) 3458, 3365, 2977, 2927, 1664, 1513, 1350, 1159 cm^{-1}; **LRMS** (ESI) 467 (17, $[M+H]^+$), 411 (17, $[M\text{-}t\text{Bu}+H]^+$), 367 (66, $[M\text{-Boc}+H]^+$); **HRMS** (ESI) calcd for $C_{21}H_{31}N_4O_6S$ $[M+H]^+$ 467.1959, observed 467.1962.

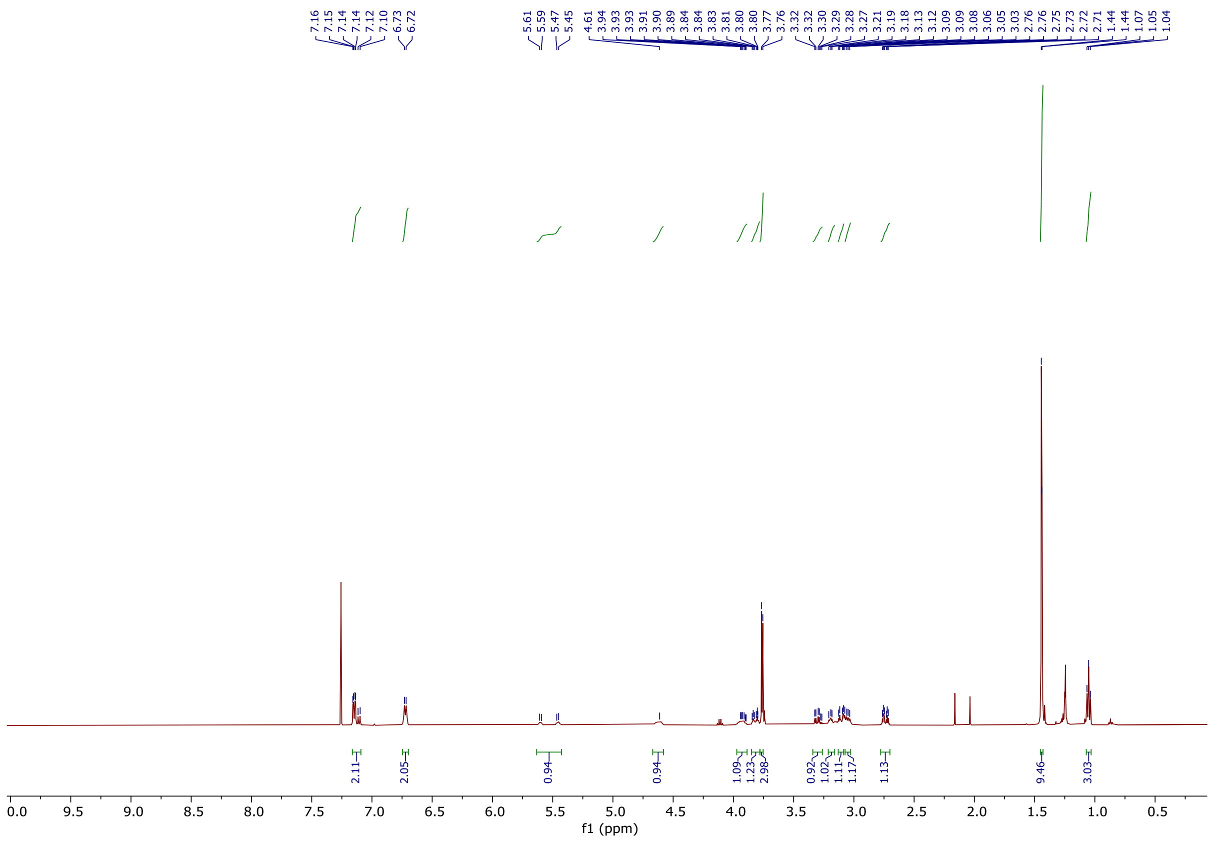

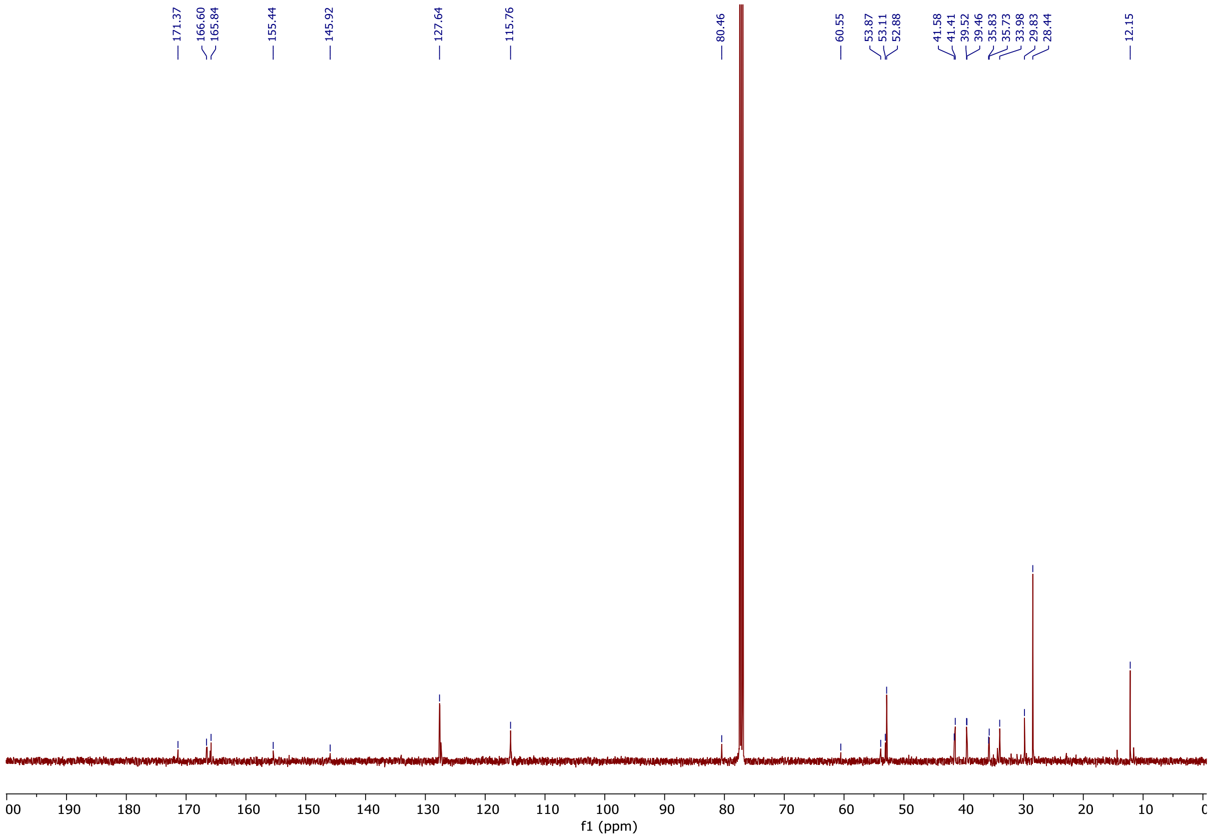

Methyl *N*-(*tert*-butoxycarbonyl)-*S*-(1,2-diethyl-3,6-dioxohexahydropyridazin-4-yl)-*L*-cysteinate 8[12]

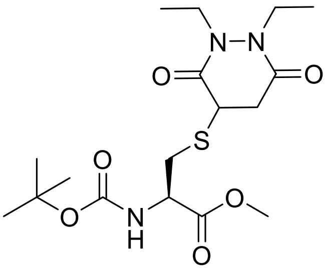

8

To a solution of *N*-(*tert*-butoxycarbonyl)-L-cysteine methyl ester **7** (122 µL, 140 mg, 0.594 mmol) in MeOH (6 mL) was added 1,2-diethyl-1,2-dihydropyridazine-3,6-dione **1** (100 mg, 0.594 mmol) and sodium acetate (146 mg, 1.78 mmol). The reaction mixture was then stirred at 21 °C for 1 h. After this time, the solvent was removed *in vacuo*, and the crude residue was purified by flash column chromatography (20% to 80% EtOAc/petrol) to give methyl *N*-(*tert*-butoxycarbonyl)-(1,2-diethyl-3,6-dioxohexahydropyridazin-4-yl)-L-cysteinate **8** as a colourless oil (128 mg, 0.317 mmol, 54%) as a mixture of diastereoisomers.

^{1}H NMR (700 MHz, $CDCl_3$, diastereomers, rotamers) δ 5.57-5.44 (m, 1H), 4.62-4.55 (m, 1H), 4.10-4.09 (m, 2H), 3.77-3.75 (m, 3H), 3.74-3.73 (m, 0.5 H), 3.71-3.69 (m, 0.5 H) 3.40-3.39 (m, 2H), 3.21-3.18 (m, 0.5 H), 3.14-3.07, (m, 1H), 2.98-2.96, (m, 0.5 H), 2.93-2.89 (m, 1H), 2.62-2.58 (m, 1H), 1.45-1.44 (m, 9H), 1.16-1.15 (m, 3H), 1.13-1.11 (t, *J* = 7.0 Hz, 3H); **^{13}C NMR** (175 MHz, $CDCl_3$, diastereomers, rotamers) δ 171.4 (C), 171.3 (C), 167.5 (C), 167.3 (C), 166.4 (C), 166.3 (C), 155.4 (C), 155.3 (C), 80.4 (C), 53.7 (CH), 52.9 (CH), 52.9 (CH_3), 41.4 (CH), 41.3 (CH), 38.7 (CH_2), 38.7 (CH_2), 38.6 (CH_2), 35.0 (CH_2), 35.0 (CH_2), 34.2 (CH_2), 33.8 (CH_2), 28.4 (CH_3), 12.2 (CH_3), 11.7 (CH_3), 11.7 (CH_3); **IR** (thin film) 3018, 2979, 2937, 1743 cm^{-1}.

2.3 Peptides and peptide-PD conjugates

2.3.1 Peptide synthesis

The tripeptides **14** and **45** were synthesised using standard Fmoc solid phase peptide conditions.

Fmoc amino acids used were: Fmoc-Gly-OH, Fmoc-Cys(Trt)-OH and Fmoc-Tyr(tBu)-OH.

First Coupling

To a 10 mL syringe fitted with a polypropylene frit was added 500 mg of 2-chlorotrityl chloride resin (1.1 mmol/g loading, 200 – 400 mesh, 1% DVB). The resin was washed with dichloromethane (5x5 mL), then allowed to swell in dichloromethane (5 mL) on a shaker for 15 minutes. Meanwhile, a coupling solution containing the first protected Fmoc-amino acid (3 equiv to resin loading) and *N*,*N*-diisopropylethylamine (6 equiv. to resin loading) was prepared in 1:1 (v/v) *N*,*N*-dimethylformamide/dichloromethane (3 mL). The dichloromethane solution was expelled from the syringe, then the coupling solution was added to the resin which was allowed to shake for 16 hours at room temperature. The coupling solution was expelled, then the resin was washed with dichloromethane (5x5 mL). A capping solution containing 0.5:1:9.5 (v/v) *N*,*N*-diisopropylethylamine/methanol/dichloromethane (5 mL) was added to the resin, which was allowed to shake at room temperature for 30 minutes. The resin was then washed with dichloromethane (3x5 mL) and *N*,*N*-dimethylformamide (3x5 mL).

Fmoc deprotections

To the resin was added 20% (v/v) piperidine in *N*,*N*-dimethylformamide, which was allowed react under shaking for 1 minute at room temperature. The piperidine solution was expelled, then 20% (v/v) piperidine in *N*,*N*-dimethylformamide was added to the resin which was allowed to react under shaking for 15 minutes at room temperature. The piperidine solution was expelled, then the resin was washed with *N*,*N*-dimethylformamide (5x5 mL).

Fmoc-amino acid couplings

A coupling solution containing the protected Fmoc-amino acid (3 equiv. to resin loading) and ethyl cyanohydroxyiminoacetate (oxyma), (3 equiv. to resin loading) was prepared in *N*,*N*-dimethylformamide (3 mL). The coupling solution was added to the resin, followed by *N*,*N*′-diisopropylcarbodiimide (3 equiv. to resin loading). The resin was allowed to react under shaking for 30 minutes at room temperature, then the coupling solution was expelled, and the resin washed with *N*,*N*-dimethylformamide (5x5 mL).

Peptide cleavage

The resin was washed with dichloromethane (5x5 mL), then dried with a gentle stream of nitrogen before being dried under vacuum overnight. To the dried resin was added a solution containing 2.5:2.5:1:94 (v/v) 1,2-ethanedithiol/H_2O/triisopropylsilane/trifluoroacetic acid

(5 mL), which was allowed to react under shaking for 3 hours at room temperature. A needle was fitted to the syringe, then the solution was then distributed dropwise into two falcon tubes containing cold diethyl ether (45 mL) to give an off-white precipitate. The precipitate was collected via centrifugation (5000 rcf, 5 minutes, 4 °C), the supernatant removed, and the precipitate washed with cold diethyl ether (45 mL). The precipitate was collected via centrifugation (5000 rcf, 5 minutes, 4 °C), the supernatant removed, and the remaining diethyl ether dried with a gentle stream of N_2. The resulting crude solid was dissolved in 5% (v/v) acetonitrile in H_2O containing 0.1% formic acid (40 mL), filtered through a 0.2 µm filter and purified via preparative-HPLC.

Fractions containing the desired peptide were identified using LC-MS, with the fractions pooled and lyophilised to give the CGY and GCY peptides as fluffy white powders.

Cys-Gly-Tyr 45

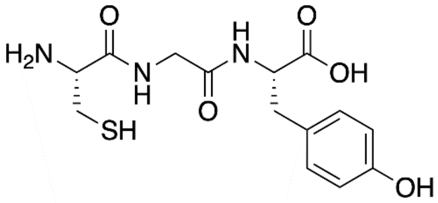

CGY

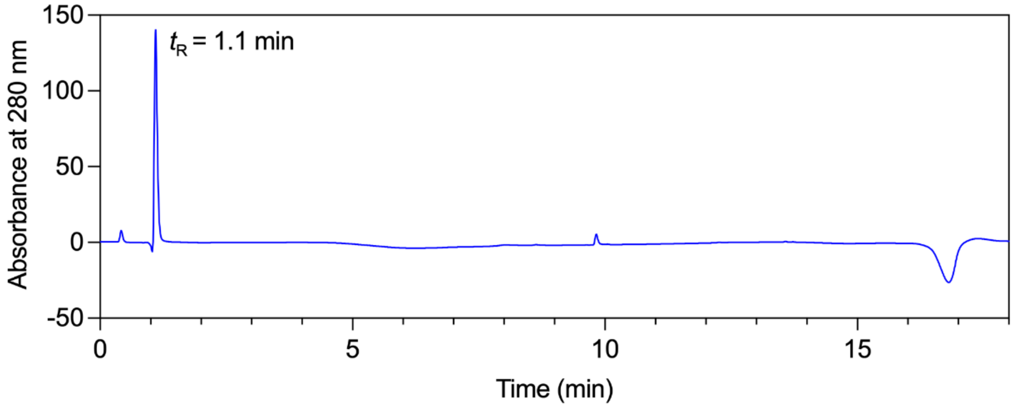

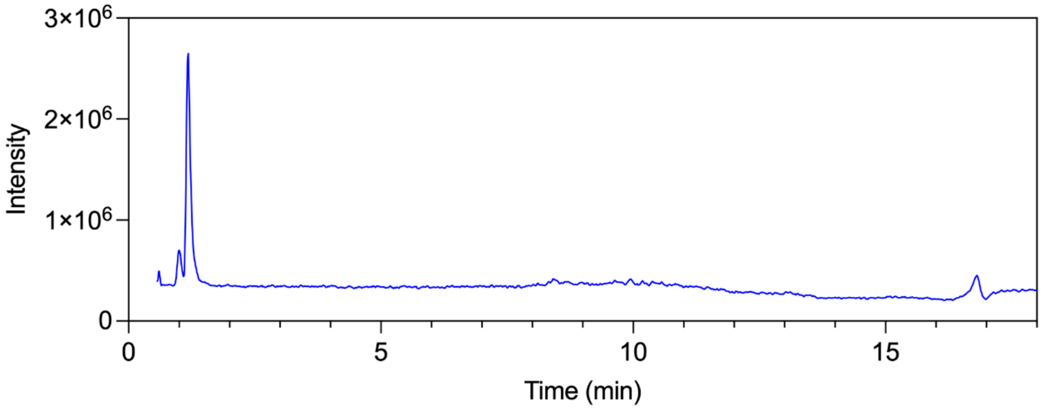

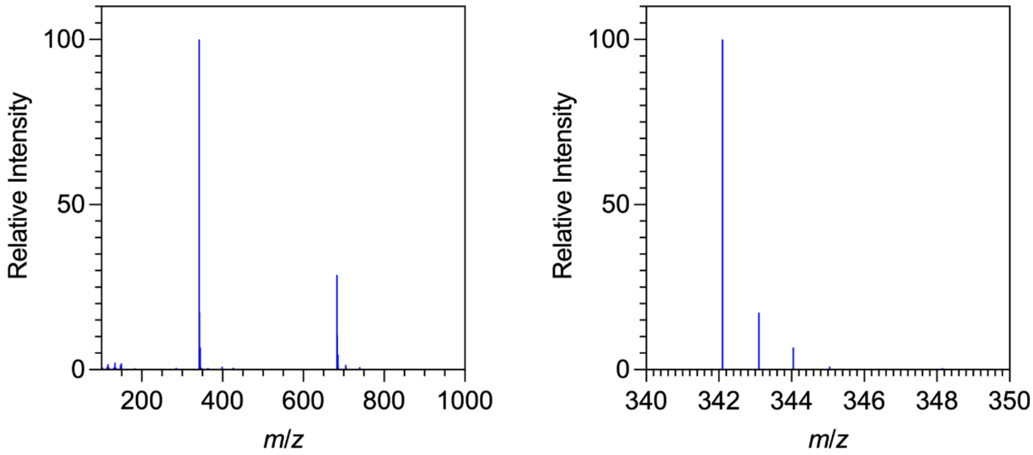

LRMS (ESI) calcd for $C_{14}H_{20}N_3O_5S$ $[M+H]^+$ 342.10 observed 342.10. **HRMS (ESI)** calcd for $C_{14}H_{20}N_3O_5S$ $[M+H]^+$ 342.1118, observed 342.1115.

Gly-Cys-Tyr 14

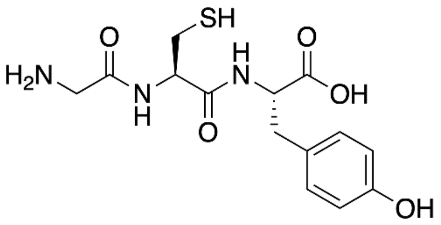

GCY

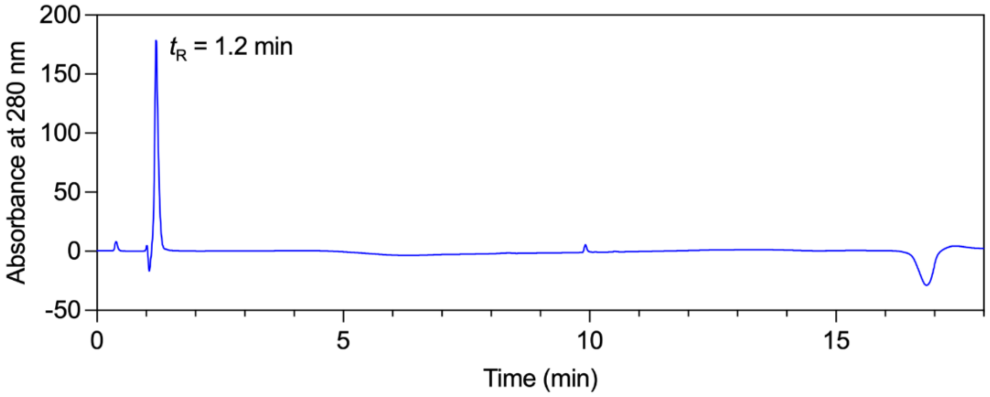

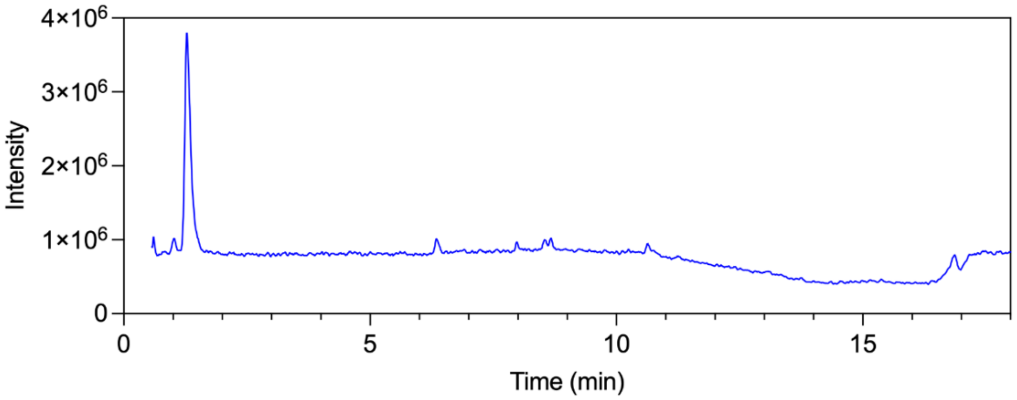

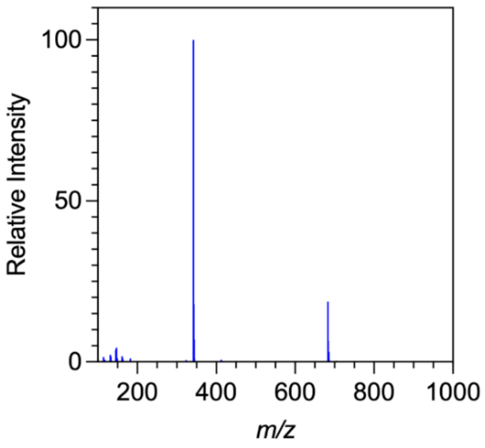

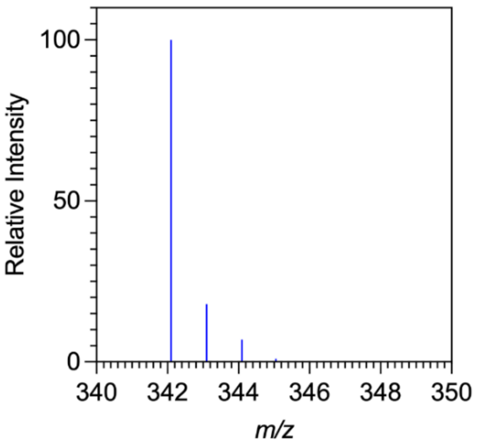

LRMS (ESI) calcd for $C_{14}H_{20}N_3O_5S$ $[M+H]^+$ 342.10 observed 342.10. **HRMS (ESI)** calcd for $C_{14}H_{20}N_3O_5S$ $[M+H]^+$ 342.1118, observed 342.1115.

2.3.2 Peptide-PD conjugates

***S*-(1-Ethyl-3,6-dioxo-2-phenylhexahydropyridazin-4-yl)-*N*-glycyl-*L*-cysteinyl-*L*-tyrosine S14/ *S*-(2-ethyl-3,6-dioxo-1-phenylhexahydropyridazin-4-yl)-*N*-glycyl-*L*-cysteinyl-*L*-tyrosine S14'**

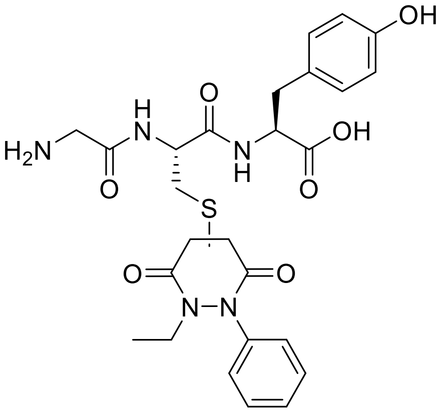

S14/S14'

To a solution of GCY peptide **14** (15 mg, 0.044 mmol) in PB buffer (100 mM phosphate, 5 mM EDTA, pH 7.4, 1 mL) was added 1-ethyl-2-phenyl-1,2-dihydropyridazine-3,6-dione **3** (10 mg, 0.044 mmol) in MeCN (1 mL). The reaction mixture was then stirred at 21 °C for 3 h. After this time, the organic solvent was removed *in vacuo*, and the crude residue was purified by reverse phase flash column chromatography (0% to 50% water/MeCN) to give an inseparable mixture *S*-(1-Ethyl-3,6-dioxo-2-phenylhexahydropyridazin-4-yl)-*N*-glycyl-*L*-cysteinyl-*L*-tyrosine **S14**/ *S*-(2-ethyl-3,6-dioxo-1-phenylhexahydropyridazin-4-yl)-*N*-glycyl-*L*-cysteinyl-*L*-tyrosine **S14'** as a white powder (12 mg, 0.022 mmol, 51%).
IR (thin film) 3286, 2951, 2854, 1682, 1644, 1593, 1512, 1416, 1244 cm^{-1}; **LRMS** (ESI) 558 (100, $[M+H]^+$); **HRMS** (ESI) calcd for $C_{26}H_{32}N_5O_7S$ $[M+H]^+$ 558.2017 observed 558.2005.

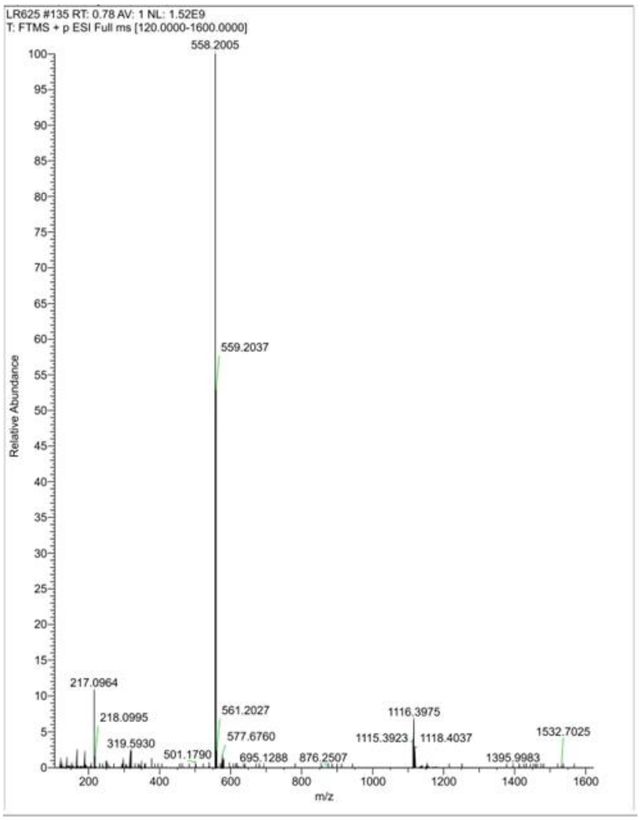
LR625 #135 RT: 0.78 AV: 1 NL: 1.52E9
T: FTMS + p ESI Full ms [120.0000-1600.0000]
558.2005
559.2037
217.0964
218.0995
319.5930
501.1790
561.2027
577.6760
695.1288
876.2507
1116.3975
1115.3923
1118.4037
1395.9983
1532.7025
Relative Abundance
m/z

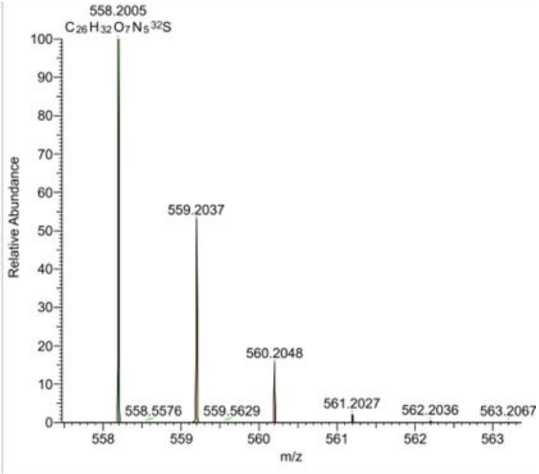
558.2005
$C_{26}H_{32}O_7N_5{}^{32}S$
559.2037
560.2048
558.5576
559.5629
561.2027
562.2036
563.2067
Relative Abundance
m/z

BCN-MePD-CGY 47

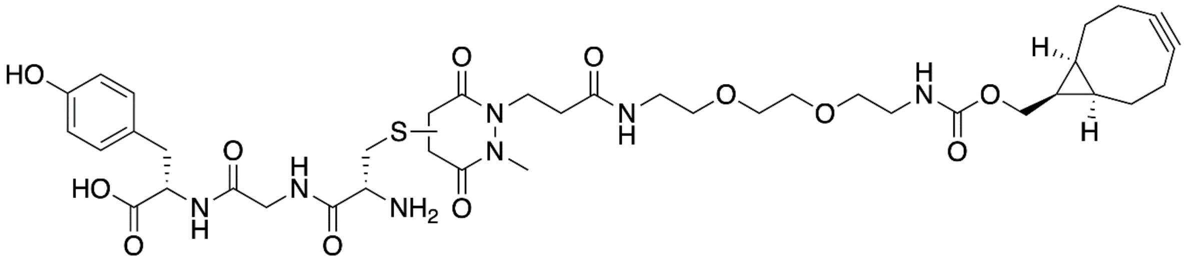

BCN-MePD-CGY

To a solution of CGY peptide (13.5 mg, 0.039 mmol) in phosphate buffer (100 mM, 1 mM EDTA, pH = 7.4, 136 µL) was added the MePD-BCN derivative, **27** (10 mg, 0.019 mmol) in acetonitrile (305 µL). The reaction was stirred at 20 °C for 16 hours then purified via preparative-HPLC. Fractions containing the title compound were identified using LC-MS, pooled, and lyophilized to give the title compound as a white solid (3.0 mg, 0.0035 mmol, 19%).

LRMS (ESI) calcd for $C_{39}H_{55}N_7O_{12}S$ $[M+H]^+$ 846.36, observed 846.2. **HRMS (ESI)** calcd for $C_{39}H_{55}N_7O_{12}S$ $[M+H]^+$ 846.3702, observed 846.3695. An impurity was found upon storage of the compound at -21 ºC, observed 847.3761.

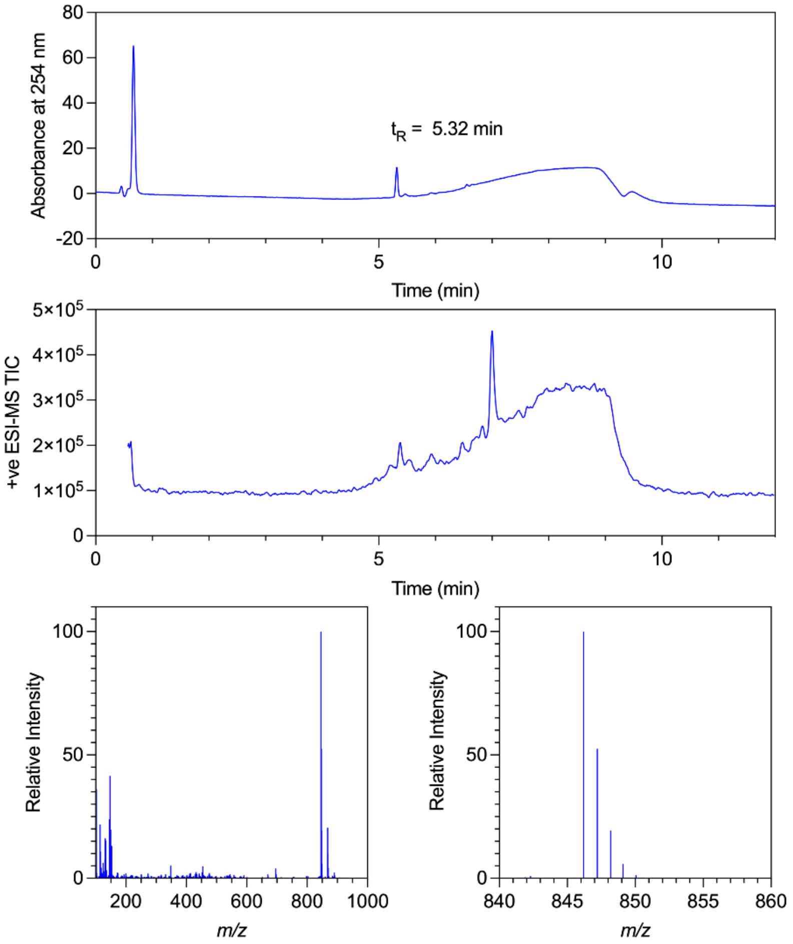

BCN-PhPD-CGY 48

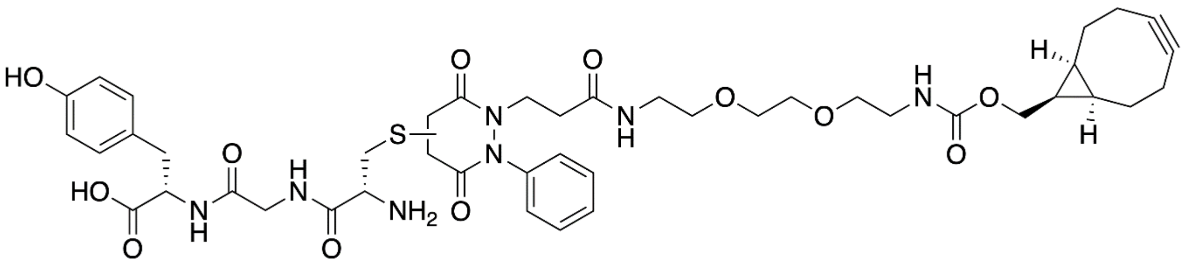

BCN-PhPD-CGY

To a solution of CGY peptide (6.2 mg, 0.018 mmol) in phosphate buffer (100 mM, 1 mM EDTA, pH = 7.4, 62 µL) was added the PhPD-BCN derivative, **27** (5.1 mg, 0.009 mmol) in acetonitrile (400 µL). The reaction was stirred at 20 °C for 16 hours then purified via preparative-HPLC. Fractions containing the title compound were identified using LC-MS, pooled, and lyophilized to give the title compound **48** as a white solid (5.5 mg, 0.0035 mmol, 39%).

LRMS (ESI) calcd for $C_{44}H_{57}N_7O_{12}S$ $[M+H]^+$ 908.38, observed 908.1. **HRMS (ESI)** calcd for $C_{39}H_{55}N_7O_{12}S$ $[M+H]^+$ 908.3859, observed 908.3852. An impurity was found upon storage of the compound at -21 ºC, observed 909.3913.

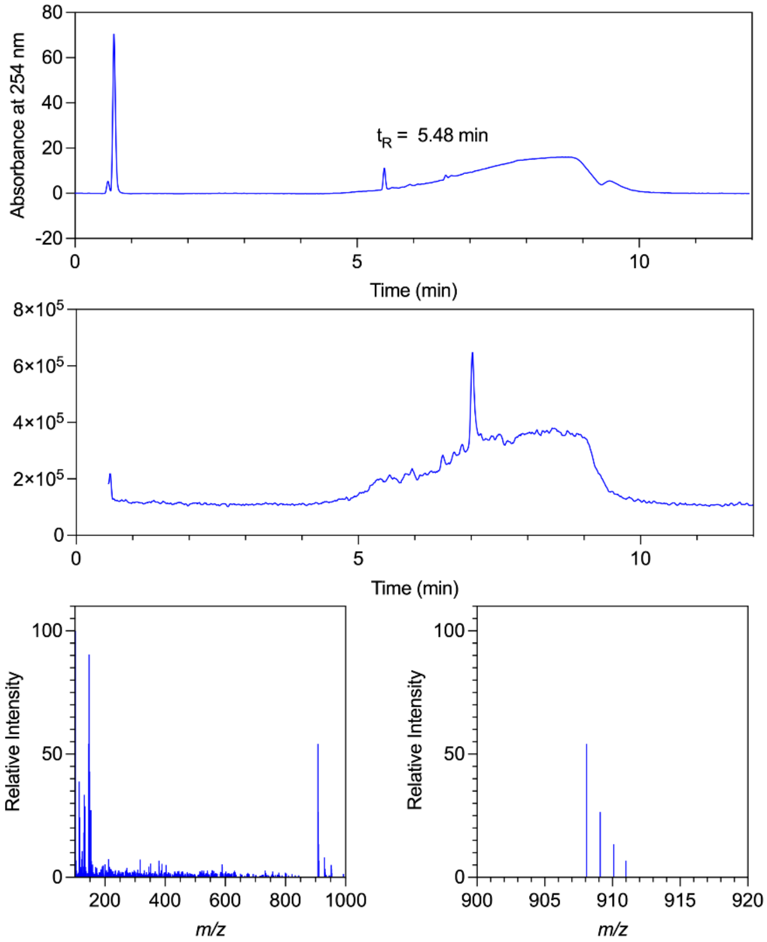

2.4 Regioisomers study

2-Ethyl-4-(hexylthio)-1-phenyltetrahydropyridazine-3,6-dione 19 and 2-ethyl-4-(hexylthio)-2-phenyltetrahydropyridazine-3,6-dione 18

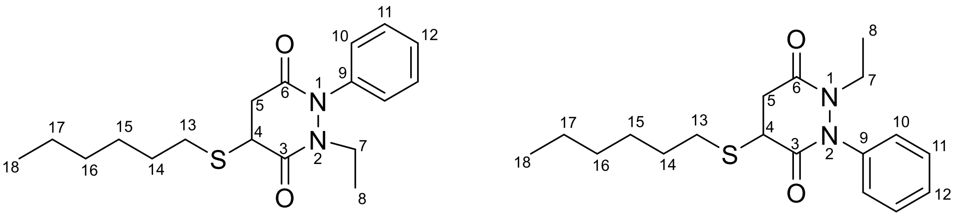

19 **18**

To a solution of n-hexanethiol **17** (33 µL, 0.23 mmol) in MeOH (3 mL) were added 1-ethyl-2-phenyl-1,2-dihydropyridazine-3,6-dione **3** (50 mg, 0.23 mmol) and sodium acetate (57 mg, 0.69 mmol). The reaction mixture was left to stir at 21 °C for 1 h. After this time, the solvent was removed *in vacuo*. The crude residue was then purified by flash column chromatography (20 to 80% EtOAc/cyclohexane) to afford an inseparable mixture of 2-ethyl-4-(hexylthio)-1-phenyltetrahydropyridazine-3,6-dione **19** and 1-ethyl-4-(hexylthio)-2-phenyltetrahydropyridazine-3,6-dione **18** in a 4:1 ratio (73 mg) as a yellow oil.

^{1}H NMR (600 MHz, $CDCl_3$, mixture of regioisomers) δ 7.43-7.29 (m, 5H), 4.09-4.04 (m, 1H), 3.71 (t, *J* = 4.0 Hz, 1H), 3.12-3.12 (m, 1H), 3.02-2.97 (m, 0.18H), 2.95-2.89 (m, 0.8H), 2.80-2.68 (m, 3H), 1.67-1.59 (m, 2H), 1.40-1.36 (m, 2.5H), 1.30-1.25 (m, 4H), 1.09 (t, *J* = 7.1 Hz, 0.5H), 1.05 (t, *J* = 7.1 Hz, 2.4H), 0.88-0.85 (m, 3H); **^{13}C NMR** (150 MHz, CDCl3, mixture of regioisomers) δ 167.5 (C), 166.8 (C), 166.2 (C), 166.2 (C), 136.7 (C), 136.4 (C), 129.3 (CH), 129.2 (CH), 127.9 (CH), 127.8 (CH), 125.6 (CH), 125.4 (CH), 42.1 (CH), 41.2 (CH), 39.8 (CH_2), 39.8 (CH_2), 36.1 (CH_2), 35.1 (CH_2), 32.1 (CH_2), 31.4 (CH_2), 29.8 (CH_2), 29.4 (CH_2), 28.5 (CH_2), 27.0 (CH_2), 22.7 (CH_2), 14.2 (CH_3), 12.1 (CH_3), 11.4 (CH_3); **IR** (thin film) 3074, 2918, 1639, 1407 cm^{-1}; **LRMS** (ESI) 335 (80, $[M+H]^+$), 217 (20, $[MC_{12}H_{12}N_2O_2+H]^+$); **HRMS** (ESI) calcd for $C_{18}H_{27}N_2O_2S$ $[M+H]^+$ 335.1786, observed 335.1788.

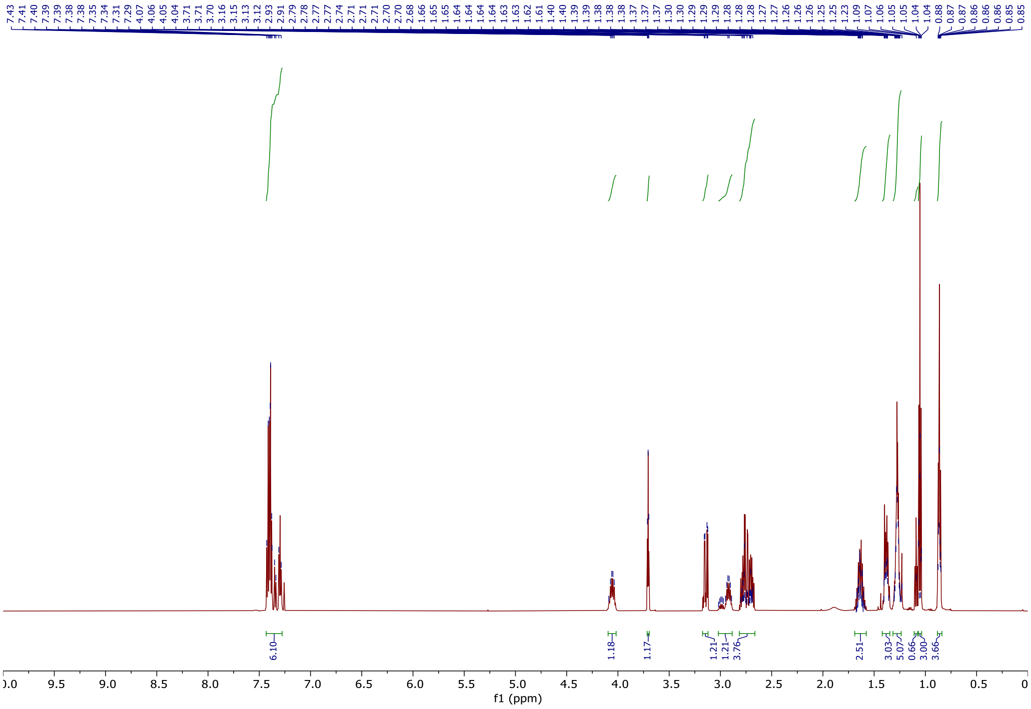
7.43 7.41 7.40 7.39 7.39 7.38 7.38 7.35 7.34 7.31 7.29 4.07 4.06 4.05 4.04 3.71 3.71 3.70 3.16 3.15 3.13 3.12 2.93 2.91 2.79 2.78 2.77 2.77 2.74 2.71 2.71 2.71 2.70 2.68 1.66 1.65 1.65 1.64 1.64 1.64 1.63 1.63 1.62 1.61 1.40 1.40 1.39 1.39 1.38 1.38 1.38 1.37 1.37 1.37 1.30 1.30 1.29 1.29 1.29 1.28 1.28 1.27 1.27 1.26 1.26 1.25 1.25 1.23 1.09 1.07 1.06 1.05 1.05 1.04 1.04 0.88 0.87 0.87 0.86 0.86 0.85 0.85
6.10 1.18 1.17 1.21 1.21 3.76 2.51 3.03 5.07 0.66 3.00 3.66
9.5 9.0 8.5 8.0 7.5 7.0 6.5 6.0 5.5 5.0 4.5 4.0 3.5 3.0 2.5 2.0 1.5 1.0 0.5 0
f1 (ppm)

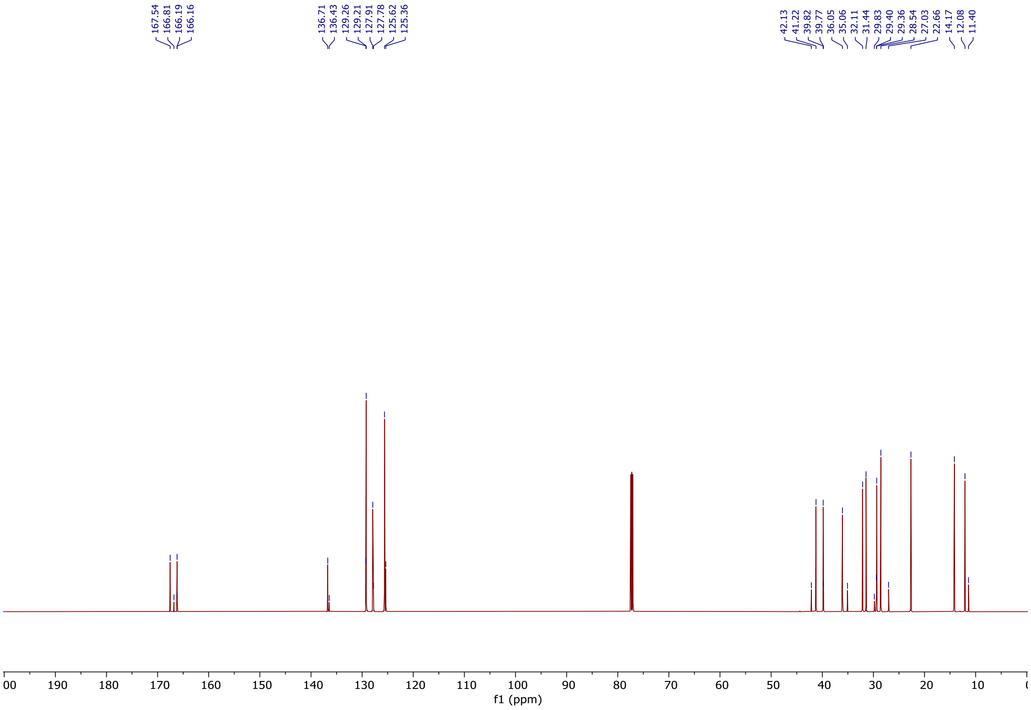
167.54 166.81 166.19 166.16
136.71 136.43 129.26 129.21 127.91 127.78 125.62 125.36
42.13 41.22 39.82 39.77 36.05 35.06 32.11 31.44 29.83 29.40 29.36 28.54 27.03 22.66 14.17 12.08 11.40
190 180 170 160 150 140 130 120 110 100 90 80 70 60 50 40 30 20 10
f1 (ppm)

*Table S1. ^{1}H, ^{13}C and ^{15}N NMR chemical shift assignments for **18** (the minor form in the mixture analysed, ~20%) and **19** (major form in the mixture analysed, ~80%). The two 7-CH2 protons distinguished based on their cis-(7c) and trans-orientation (7t) relative to the 6-CO group. For some of ^{13}C peaks, J couplings with protons were measured using proton-coupled ^{13}C NMR spectra. Where available, the assignments of the coupled protons are included in brackets in columns under "Multiplicity and ...". The assignments of the protons coupled to ^{13}C and ^{15}N sites in HMBC spectra are also included in brackets in columns under "Multiplicity and ...". ^{15}N NMR chemical shifts were measured from ^{1}H, ^{15}N-HMBC spectra.*

	Major (**19**)	Multiplicity and *J* couplings (in Hz) or HMBC cross-peaks with ^{1}H signals (**19**)	Minor (**18**)	Multiplicity and *J* couplings (in Hz) or HMBC cross-peaks with ^{1}H signals (**18**)	Major (**19**)	Minor (**18**)
1-N	156.3	(10-oPh, 5e,7c,7t)	156.6	(8-Me, 5e)	156.3	156.6
2-N	156.0	(8-Me, 4e)	154.8	(10-oPh, 4e, 7c)	156.0	154.8
3-CO	-	ddtd 9.4 (5e), 5.1 (4e), 3.3 (5a), 3.3 (7t), 1.7 (7c)	-	ddd 9.5 (5e), 5.1 (4e), 3.7 (5a)	167.55	166.83
4e-CH	3.714	dd 4.3, 3.7	3.716	Overlapping	41.23	42.14
5a[a]	3.150	dd -15.9, 4.3	3.160	dd -16.0, 4.5	36.06	35.07
5e[a]	2.761	dd -15.9, 3.7	2.739	dd -16.0, 3.9	-	-
6-CO	-	dt 9.4, 6.2	-	Overlapping	166.20	166.16
7c-CH to CO	4.066	dq -14.2, 7.1	4.078	dq -14.2, 7.1	39.83	39.79
7t-CH_2 to CO	2.931	dq -14.2, 7.1	3.002	dq -14.2, 7.1	-	-
8-Me	1.064	t 7.1	1.101	t 7.1	12.09	11.41
9-Cq	-				136.71	136.43
10-oPh	7.394		7.353		125.63	125.36
11-mPh	7.425		7.425		129.22	129.27
12-pPh	7.310		7.308		127.93	127.80
13-CH_2	2.789		2.801		32.12	32.13
13-CH_2	2.704		2.712		-	-
14-CH_2	1.643		1.643		29.36	29.40
15-CH_2	1.386		1.386		28.55	28.55
16-CH_2	1.280		1.280		31.44	31.45
17-CH_2	1.283		1.283		22.67	22.67
18-Me	0.873		0.879		14.17	14.17

[a]Assigned using ^{13}C satellites in the ^{1}H NMR spectrum. $^{1}J_{CH}$ couplings measured from the proton-coupled ^{13}C NMR spectrum are different for 5a and 5e protons, ^{1}J(C5,H5a)=129.7 Hz and ^{1}J(C5,H5e)=140.7 Hz.

Di-*tert*-butyl 1-ethyl-2-methylhydrazine-1,2-dicarboxylate S4

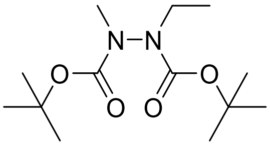

S4

To a solution of di-*tert*-butyl-1-methylhydrazine-1,2-dicarboxylate (500 mg, 2.03 mmol) in DMF, was added bromoethane (758 µL, 10.1 mmol) and caesium carbonate (3.31 g, 10.1 mmol). The reaction mixture was then left to stir at 21 °C for 16 h. After this time, the solvent was removed *in vacuo* with toluene co-evaporation (3 x 20 mL, as an azeotrope). Residual toluene was subsequently removed *in vacuo* with chloroform co-evaporation (3 x 20 mL, as an azeotrope). Purification of the crude residue by flash column chromatography (50 to 100% EtOAc/cyclohexane) afforded di-*tert*-butyl 1-ethyl-2-methylhydrazine-1,2-dicarboxylate **S4** (311 mg, 1.13 mmol, 56%) as a yellow oil.

^{1}H NMR (400 MHz, $CDCl_3$, rotamers) δ 3.51-3.38 (m, 2H), 3.02 (s, 3H), 1.51-1.43 (m, 18H), 1.15 (t, *J* = 7.3 Hz, 3H); **^{13}C NMR** (150 MHz, $CDCl_3$) δ 154.5 (C), 80.8 (C), 80.6 (C), 43.0 (CH_2), 36.8 (CH_3), 28.4 (CH_3), 28.3 (CH_3), 28.1 (CH_3), 12.9 (CH_3); **IR** (thin film) 2977, 2932, 1677, 1367, 1169 cm^{-1}; **LRMS** (ESI) 275 (15, $[M+H]^+$), 219 (20, $[M\text{-}t\text{Bu}+H]^+$), 163 (65, $[M\text{-}2t\text{Bu}+H]^+$); **HRMS** (ESI) calcd for $C_{13}H_{27}N_2O_4$ $[M+H]^+$ 275.1965, observed 275.1965.

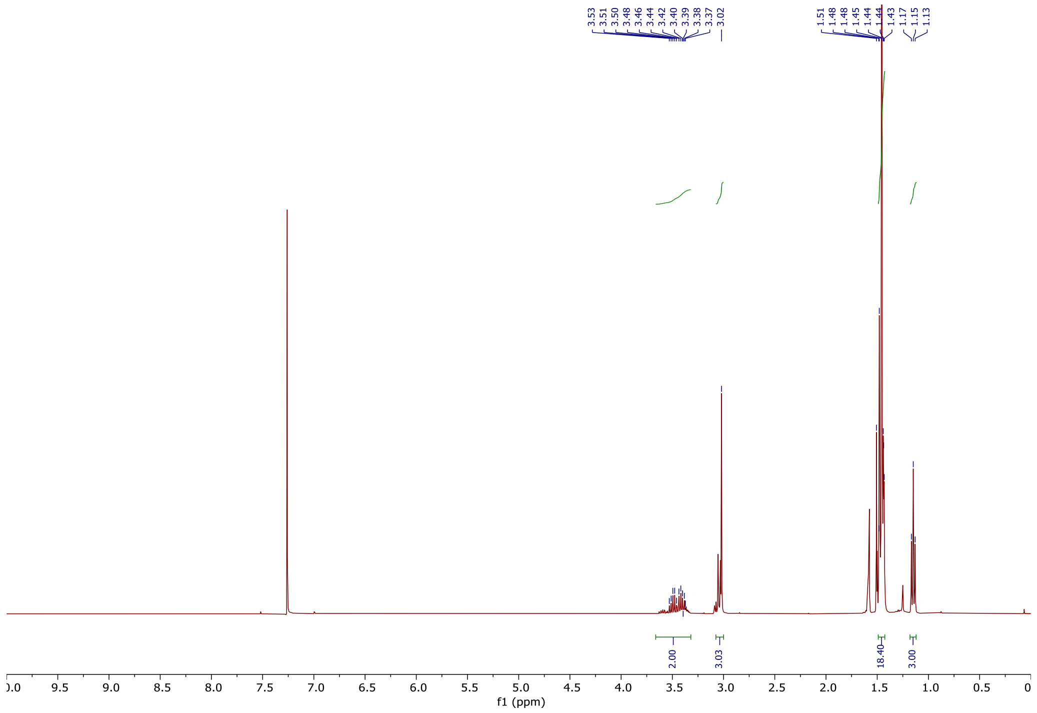
3.53
3.51
3.50
3.48
3.46
3.44
3.42
3.40
3.39
3.38
3.37
3.02
1.51
1.48
1.48
1.45
1.44
1.44
1.43
1.17
1.15
1.13
2.00
3.03
18.40
3.00
f1 (ppm)

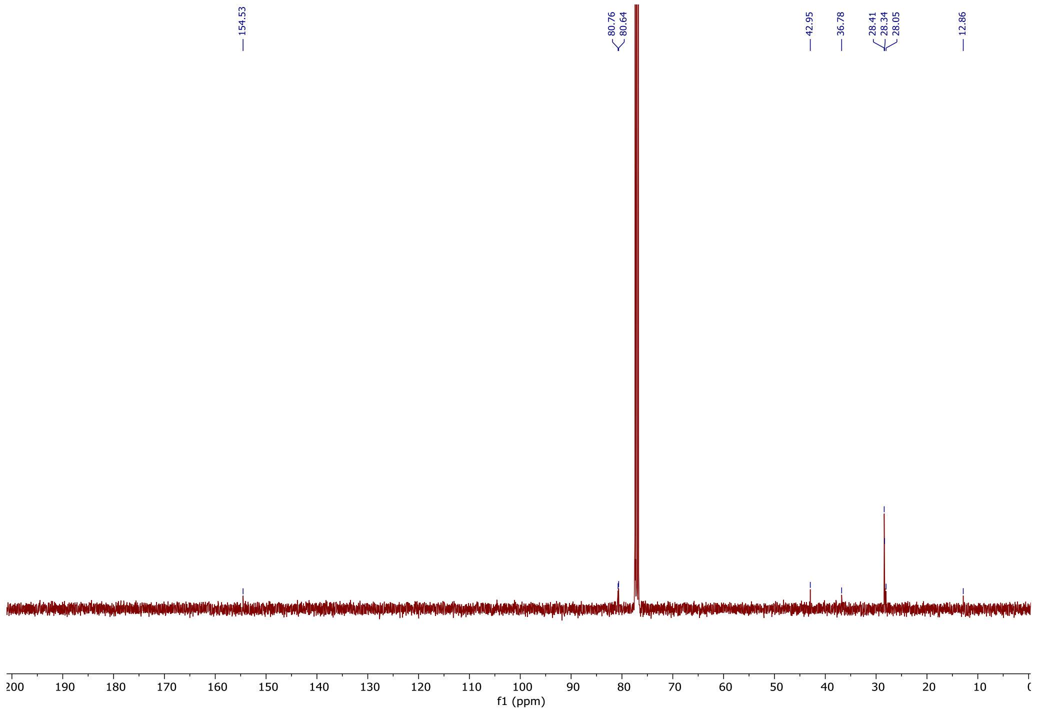
154.53
80.76
80.64
42.95
36.78
28.41
28.34
28.05
12.86
f1 (ppm)

1-Ethyl-2-methyl-1,2-dihydropyridazine-3,6-dione 16

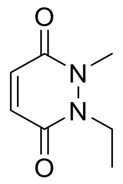

16

To a solution of di-*tert*-butyl 1-ethyl-2-methylhydrazine-1,2-dicarboxylate **S4** (275 mg, 1.00 mmol) in AcOH (10 mL) was added maleic anhydride (147 mg, 1.50 mmol). The reaction mixture was then heated under reflux with stirring for 16 h. After this time, the reaction mixture was allowed to cool to 21 °C, and the solvent was removed *in vacuo* with toluene co-evaporation (3 x 20 mL, as an azeotrope). Residual toluene was subsequently removed *in vacuo* with chloroform co-evaporation (3 x 20 mL, as an azeotrope). Purification of the crude residue by flash column chromatography (50 to 100% EtOAc/cyclohexane) afforded 1-ethyl-2-methyl-1,2-dihydropyridazine-3,6-dione **16** (130 mg, 0.84 mmol, 84%) as a beige solid.

^{1}H NMR (600 MHz, $CDCl_3$) δ 6.87 (s, 2H), 4.16 (q, *J* = 7.1 Hz, 2H), 3.60 (s, 3H), 1.28 (t, *J* = 7.1 Hz, 3H); **^{13}C NMR** (150 MHz, $CDCl_3$) δ 157.4 (C), 156.9 (C), 134.7 (CH), 134.5 (CH), 40.9 (CH_2), 32.7 (CH_3), 13.4 (CH_3); **IR** (solid) 2920, 1632, 1582 cm^{-1}; **LRMS** (ESI) 155 (100, [M]$^{+}$); **HRMS** (ESI) calcd for $C_7H_{11}N_2O_2$ [M+H]$^{+}$ 155.0815, observed 155.0815.

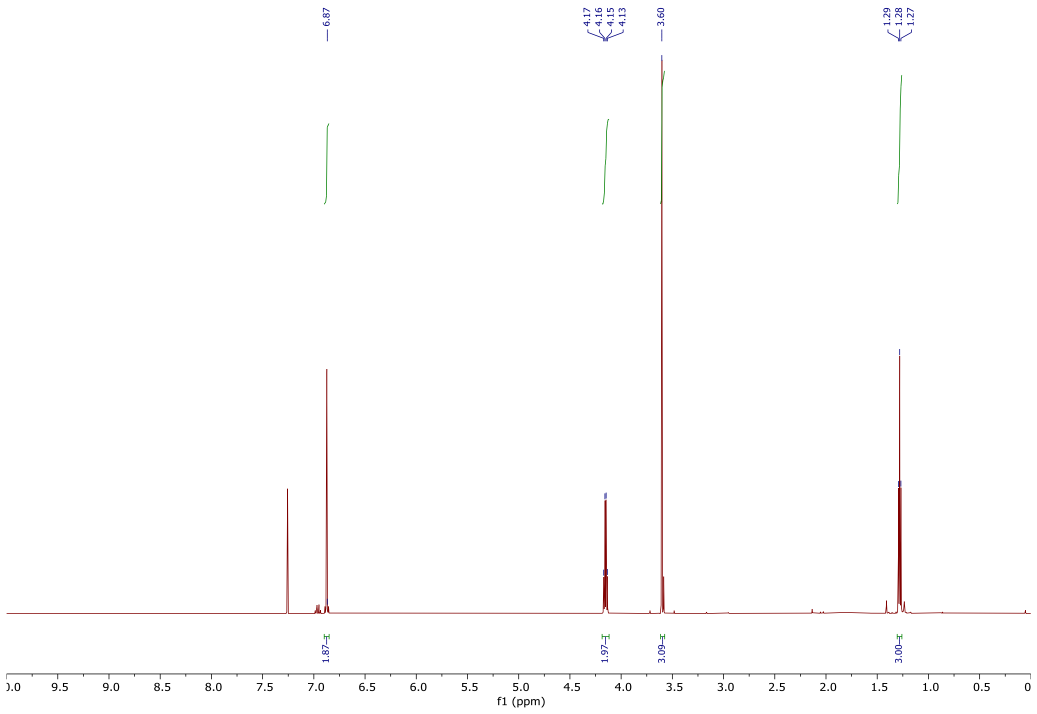

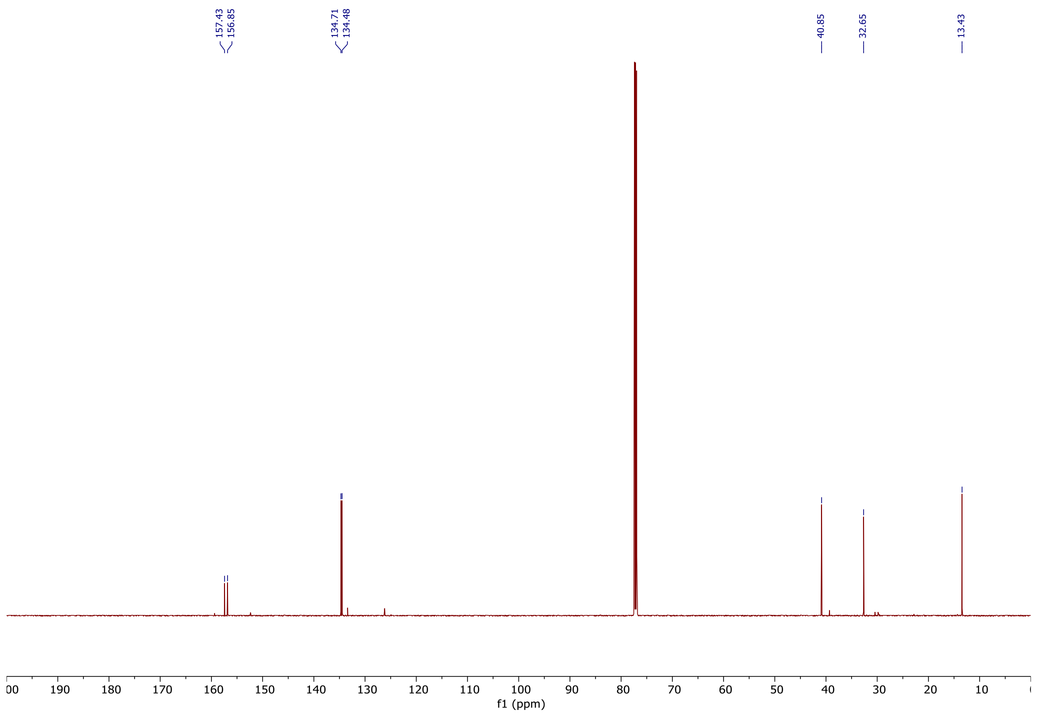
157.43
156.85
134.71
134.48
40.85
32.65
13.43
00
190
180
170
160
150
140
130
120
110
100
90
80
70
60
50
40
30
20
10
f1 (ppm)

2-Ethyl-4-(hexylthio)-1-methyltetrahydropyridazine-3,6-dione 20

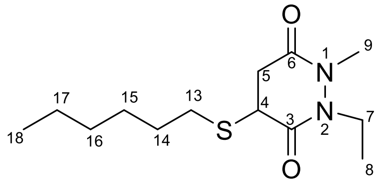

20

To a solution of n-hexanethiol **17** (38 µL, 0.32 mmol) in MeOH (3 mL) were added 1-ethyl-2-phenyl-1,2-dihydropyridazine-3,6-dione **16** (50 mg, 0.32 mmol) and sodium acetate (80 mg, 0.97 mmol). The reaction mixture was left to stir at 21 °C for 1 h. After this time, the solvent was removed *in vacuo* and a crude ^{1}H NMR of the reaction was taken (600 MHz, CD_3OD, see below).)Purification of the crude residue by flash column chromatography (20 to 80% EtOAc/cyclohexane) enabled the separation of both regioisomers obtained in a 1:1 ratio and yielded 2-ethyl-4-(hexylthio)-1-phenyltetrahydropyridazine-3,6-dione **20** (35 mg, 0.13 mmol, 41%) as a yellow oil.

^{1}H NMR (600 MHz, CD_3OD) δ 4.14-4.08 (m, 1H), 3.67 (t, *J* = 3.9 Hz, 1H), 3.47-3.41 (m, 1H), 3.20 (s, 3H), 3.13 (dd, *J* = 16.3, 4.3 Hz, 1H), 2.74-2.62 (m, 2H), 2.55 (dd, *J* = 16.3, 3.5 Hz, 1H), 1.65-1.58 (m, 2H), 1.42-1.37 (m, 2H), 1.33-1.32 (p, *J* = 7.7 Hz, 4H), 1.14 (t, *J* = 7.0 Hz, 3H), 0.91 (t, *J* = 7.1 Hz, 3H); **^{13}C NMR** (150 MHz, CD_3OD) δ 169,8 (C), 169.3 (C), 42.2 (CH), 40.4 (CH_2), 35.7 (CH_2), 33.5 (CH_3), 32.5 (CH_2), 32.4 (CH_2), 30.4 (CH_2), 29.4 (CH_2), 23.6 (CH_2), 14.3 (CH_3), 12.3 (CH_3); **IR** (thin film) 2954, 2926, 2870, 2856, 1664, 1376 cm^{-1}; **LRMS** (ESI) 273 (100, $[M]^+$); **HRMS** (ESI) calcd for $C_{13}H_{25}N_2O_2S$ $[M+H]^+$ 273.1631, observed 273.1628.

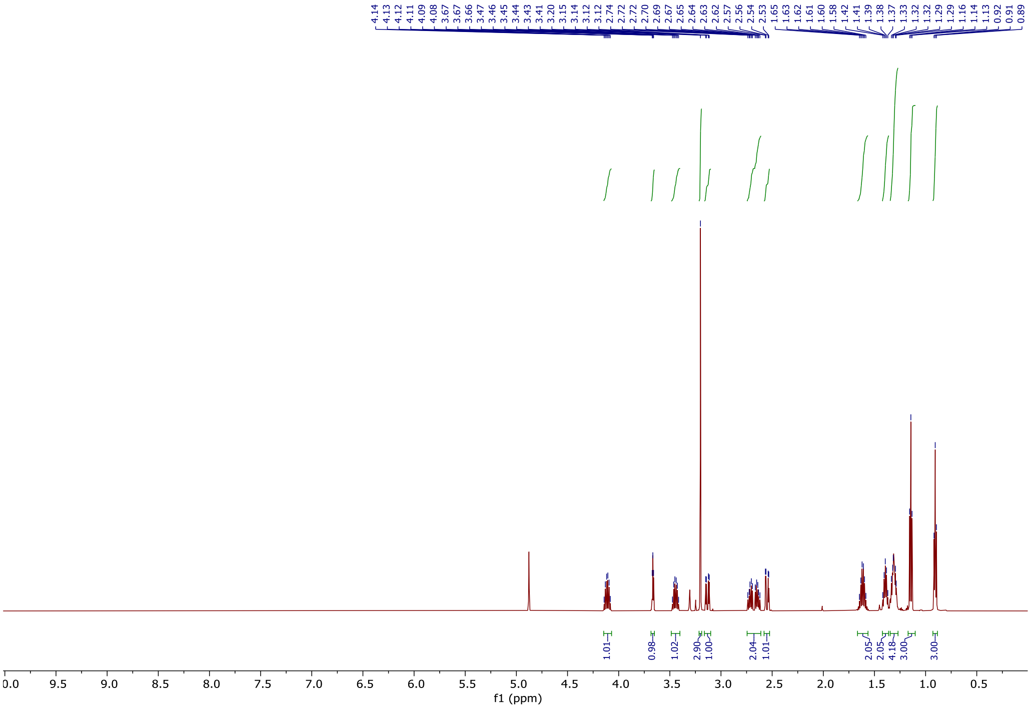

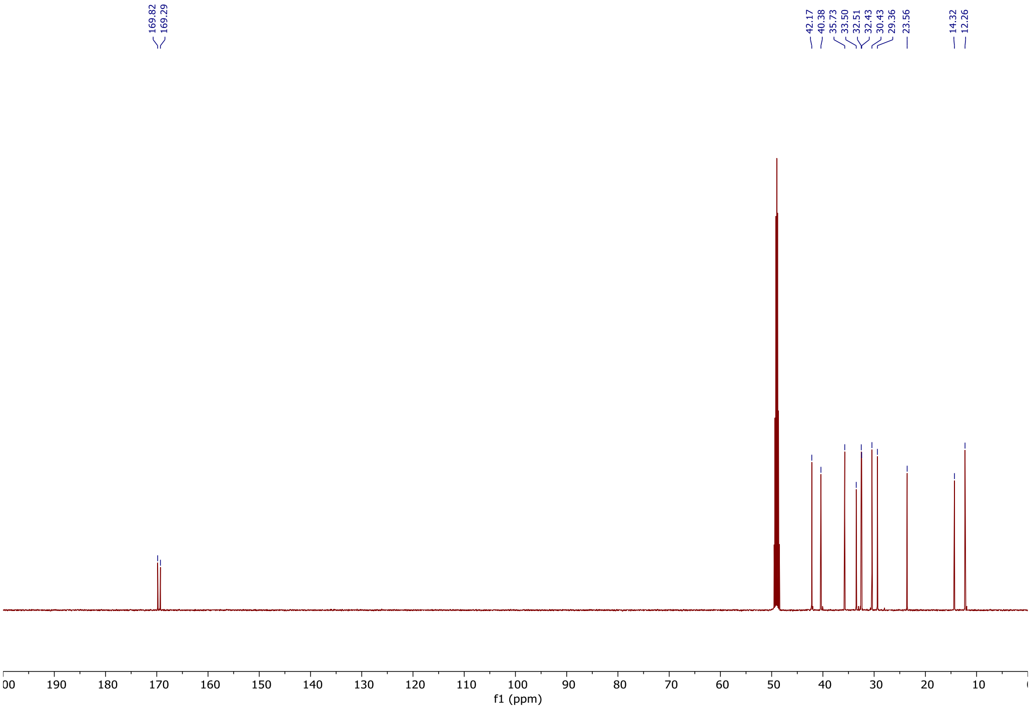

*Table S2. ^{1}H, ^{13}C and ^{15}N NMR chemical shift assignments for **20**. The two 7-CH2 protons distinguished based on their cis-(7c) and trans-orientation (7t) relative to the 6-CO group. For some of ^{13}C peaks, J couplings with protons were measured using proton-coupled ^{13}C NMR spectra. The assignments of the protons coupled to ^{13}C and ^{15}N sites in HMBC spectra are included in brackets in columns under "Multiplicity and …". ^{15}N NMR chemical shifts were measured from ^{1}H,^{15}N-HMBC spectra.*

	δ_H / ppm	Multiplicity and *J* couplings (in Hz) or HMBC cross-peaks with ^{1}H signals (in brackets)	δ_C / ppm	$^{1}J_{CH}$/ Hz	Multiplicity of ^{13}C signals in proton-coupled ^{13}C spectra and $^{n}J_{CH}$ values (in Hz)
1-N	-	(7c, 7t,9, 5e)	140.1	-	
2-N	-	(4e, 7t, 9, 8)	156.3	-	
3-CO	-		169.24	-	
4-CH	3.657	dd 4.3, 3.6	42.09	152.1	quintet 3.8
5a[a]	3.122	dd -16.3, 4.3	35.68	130.9	d 2.8
5e[a]	2.545	dd -16.3, 3.6	-	140.3	
6-CO	-	dt 9.4, 6.2	169.80		
7c-CH to CO	4.104	dq -14.5, 7.1	40.32	141.2	q 4.4
7t-CH_2 to CO	3.440	dq -14.5, 7.1	-	141.2	
8-Me	1.140	t 7.1	12.26	127.6	dd 3.6, 2.5
9-Me	3.195		33.47	140.9	-
13-CH_2	2.709	ddd -12.8, 8.1, 6.5	32.45	139.4	
13-CH_2	2.638	ddd -12.8, 8.1, 6.7	-		
14-CH_2	1.610		30.41	127.1	
15-CH_2	1.390		29.38	124.2	
16-CH_2	1.303		32.48	124.1	
17-CH_2	1.312		23.58	125.9	
18-Me	0.900		14.36	124.5	

[a]Assigned using ^{13}C satellites in the ^{1}H NMR spectrum. $^{1}J_{CH}$ couplings measured from the proton-coupled ^{13}C NMR spectrum are different for 5a and 5e protons, $^{1}J(C5,H5a)=130.9$ Hz and $^{1}J(C5,H5e)=140.3$ Hz.

^{1}H NMR of the crude product (top: unzoomed version of the spectra shown in Figure 4B of the manuscript, bottom: zoomed version between 4.5 and 0.5 ppm), displaying a regioisomer ratio of about 1:1.2:

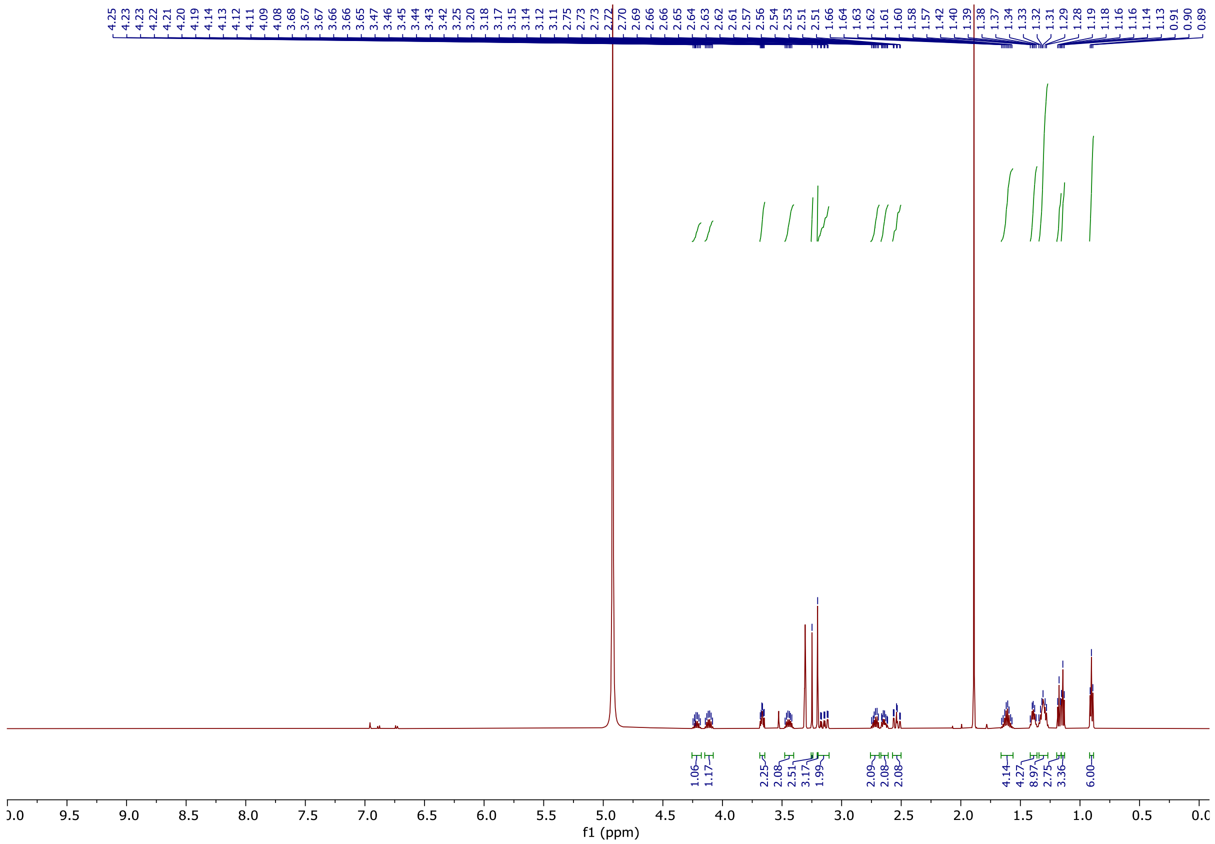

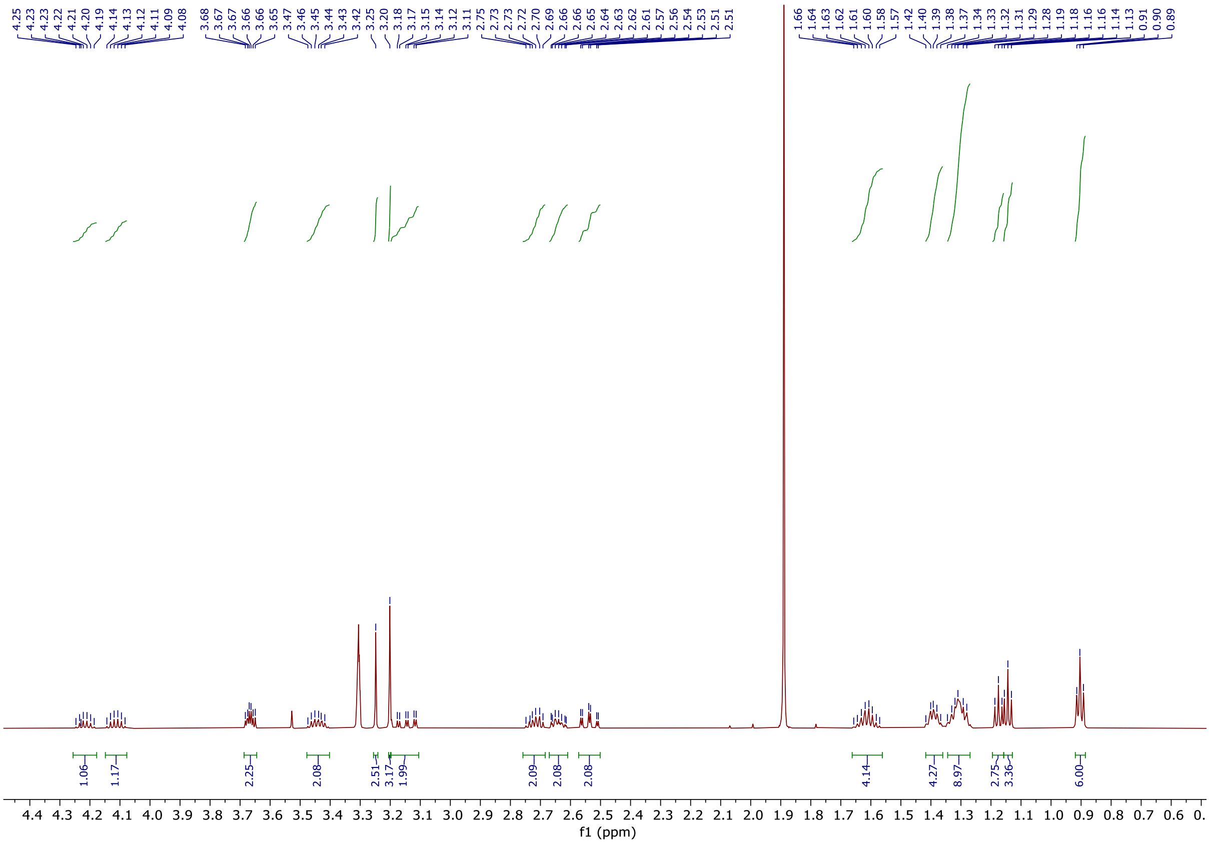

1-Ethyl-4-(hexylthio)-2-methyltetrahydropyridazine-3,6-dione 21

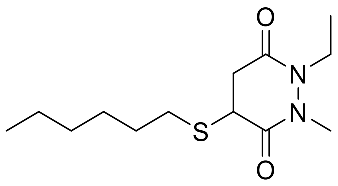

21

To a solution of n-hexanethiol **17** (38 µL, 0.32 mmol) in MeOH (3 mL) were added 1-ethyl-2-phenyl-1,2-dihydropyridazine-3,6-dione **16** (50 mg, 0.32 mmol) and sodium acetate (80 mg, 0.97 mmol). The reaction mixture was left to stir at 21 °C for 1 h. After this time, the solvent was removed *in vacuo* and a crude ^{1}H NMR of the reaction was taken (600 MHz, CD_3OD, see above). A flash column chromatography (20 to 80% EtOAc/cyclohexane) enabled the separation of both regioisomers obtained in a 1:1 ratio and yielded 1-ethyl-4-(hexylthio)-2-phenyltetrahydropyridazine-3,6-dione **21** (38 mg, 0.14 mmol, 44%) as a yellow oil.

^{1}H NMR (600 MHz, $CDCl_3$) δ 4.24-4.18 (m, 1H), 3.60 (t, *J* = 4.2 Hz, 1H), 3.41-3.35 (m, 1H), 3.23 (s, 3H), 2.99 (dd, *J* = 4.6, 16.0 Hz, 1H), 2.73-2.69 (m, 1H), 2.63-2.58 (m, 2H), 1.62-1.56 (m, 2H), 1.37-1.32 (m, 2H), 1.29-1.24 (m, 4H), 1.18 (t, *J* = 7.2 Hz, 3H), 0.86 (t, *J* = 7.0 Hz, 3H); **^{13}C NMR** (150 MHz, $CDCl_3$) δ 167,7 (C), 166.7 (C), 41.0 (CH), 39.1 (CH_2), 35.3 (CH_2), 32.7 (CH_2), 32.0 (CH_2), 31.4 (CH_2), 29.3 (CH_3), 28.5 (CH_2), 22.6 (CH_2), 14.2 (CH_3), 11.7 (CH_3); **IR** (thin film) 2954, 2926, 2870, 2856, 1664, 1376 cm^{-1}; **LRMS** (ESI) 273 (100, [M]$^{+}$); **HRMS** (ESI) calcd for $C_{13}H_{25}N_2O_2S$ [M+H]$^{+}$ 273.1631, observed 273.1628.

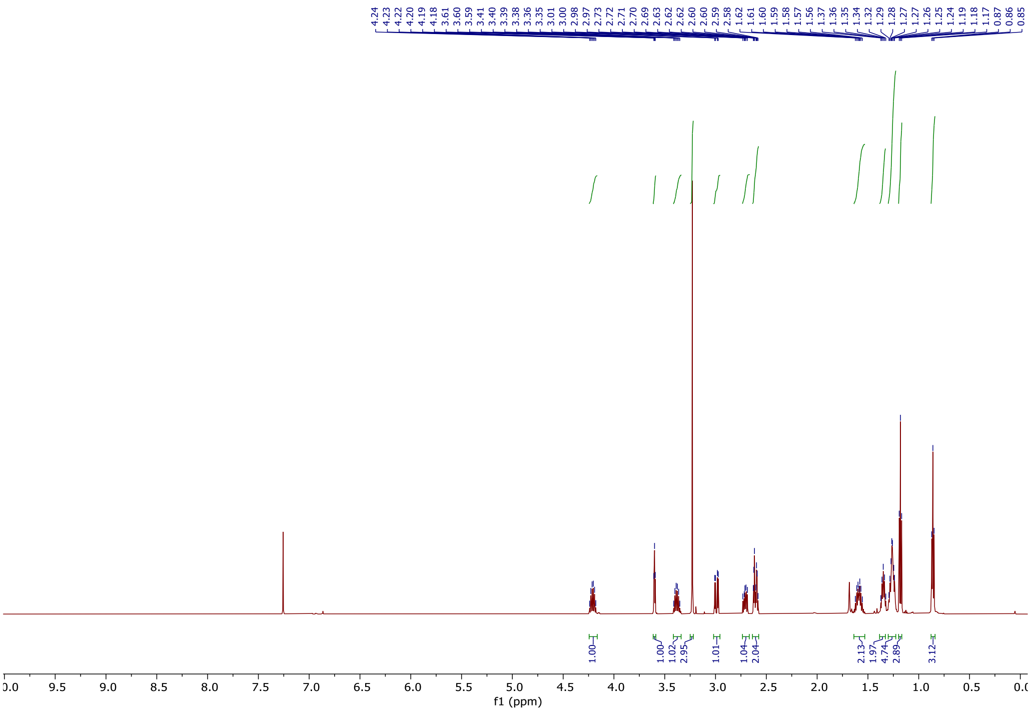

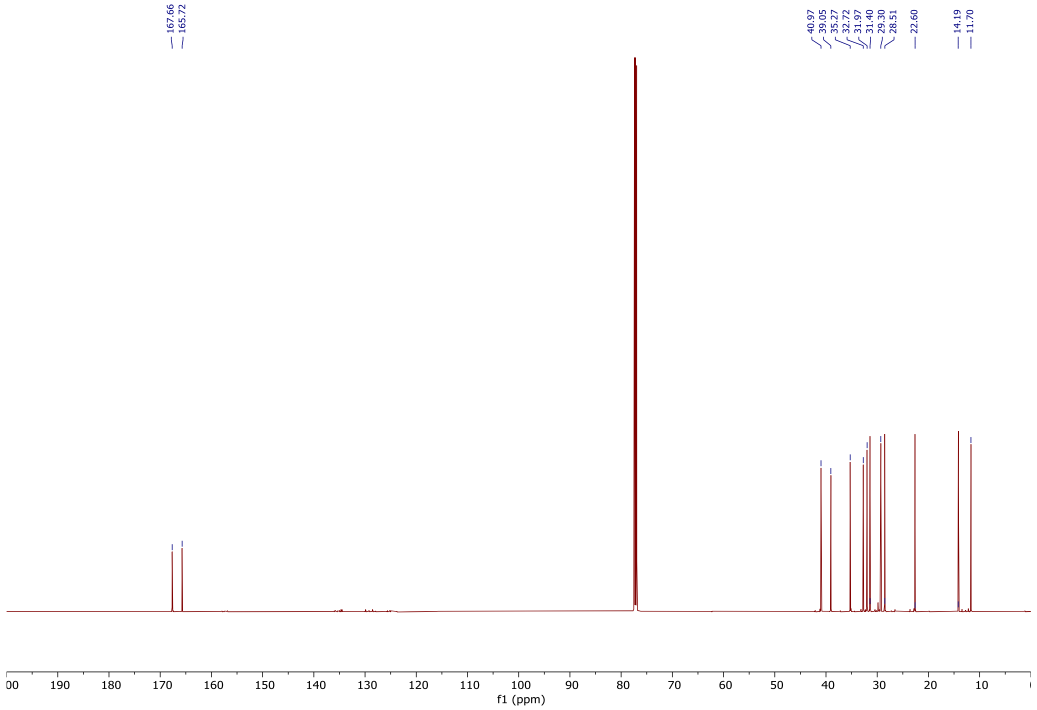

167.66
165.72
40.97
39.05
35.27
32.72
31.97
31.40
29.30
28.51
22.60
14.19
11.70
190
180
170
160
150
140
130
120
110
100
90
80
70
60
50
40
30
20
10
f1 (ppm)

2.5 Clickable and functionalised model PDs

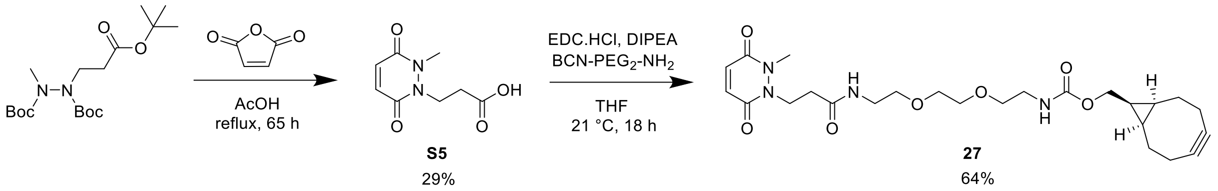

*Scheme S2. Synthesis of N-Me,N'-BCN PD **27***

3-(2-Methyl-3,6-dioxo-3,6-dihydropyridazin-1(2*H*)-yl)propanoic acid S5

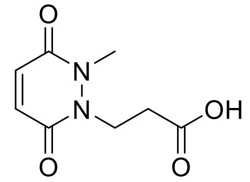

S5

Maleic anhydride (0.68 g, 6.89 mmol) was dissolved in AcOH (65 mL) and the reaction mixture heated under reflux for 30 min. After this time, to this solution, was added di-*tert*-butyl-1-(3-(tert-butoxy)-3-oxopropyl)-2-methylhydrazine-1,2- dicarboxylate obtained according to the procedure previously reported[11] (2.15 g, 5.74 mmol) and the reaction was heated under reflux for 16 h. After this, the reaction mixture was concentrated *in vacuo* with toluene co-evaporation (3 × 30 mL, as an azeotrope) and the crude residue purified by flash column chromatography (0 to 20% MeOH/EtOAc (1% AcOH)) to afford 3-(2-methyl-3,6-dioxo-3,6-dihydropyridazin-1(2H)-yl)propanoic acid **S5** (330 mg, 1.67 mmol, 29%) as a beige solid.
^{1}H NMR (500 MHz, DMSO-d$_6$) δ 12.44 (s, 1H), 6.92 (q, *J* = 3.3 Hz, 2H), 4.22 (t, *J* = 9.0 Hz, 2H), 3.51 (s, 3H), 2.58 (t, *J* = 9.0 Hz, 2H); **^{13}C NMR** (150 MHz, DMSO-d$_6$) δ 171.9 (C), 156.6 (C), 156.5 (C), 134.5 (CH), 134.2 (CH), 41.2 (CH$_3$), 32.5 (CH$_2$), 32.0 (CH$_2$); **IR** (solid) 3077, 2918, 1715, 1594, 1560 cm^{-1}; **LRMS** (ESI) 199 (100, [M+H]$^+$); **HRMS** (ESI) calcd for $C_8H_{11}N_2O_4$ [M+H]$^+$ 199.0719 observed 199.0715.

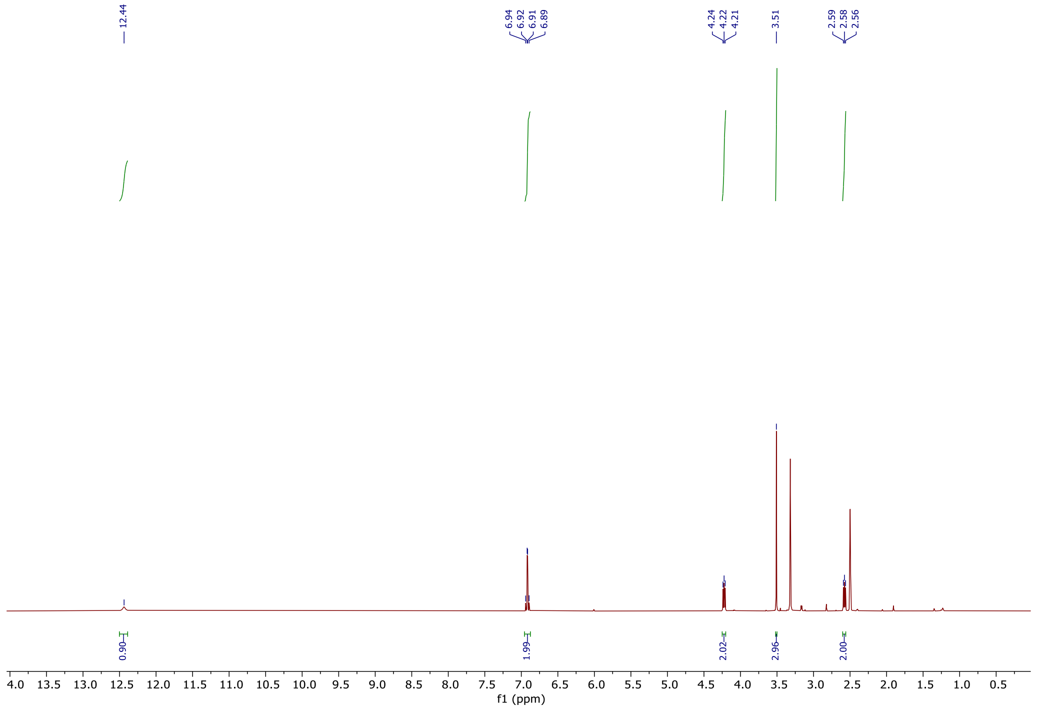
12.44
6.94
6.92
6.91
6.89
4.24
4.22
4.21
3.51
2.59
2.58
2.56
0.90
1.99
2.02
2.96
2.00
f1 (ppm)

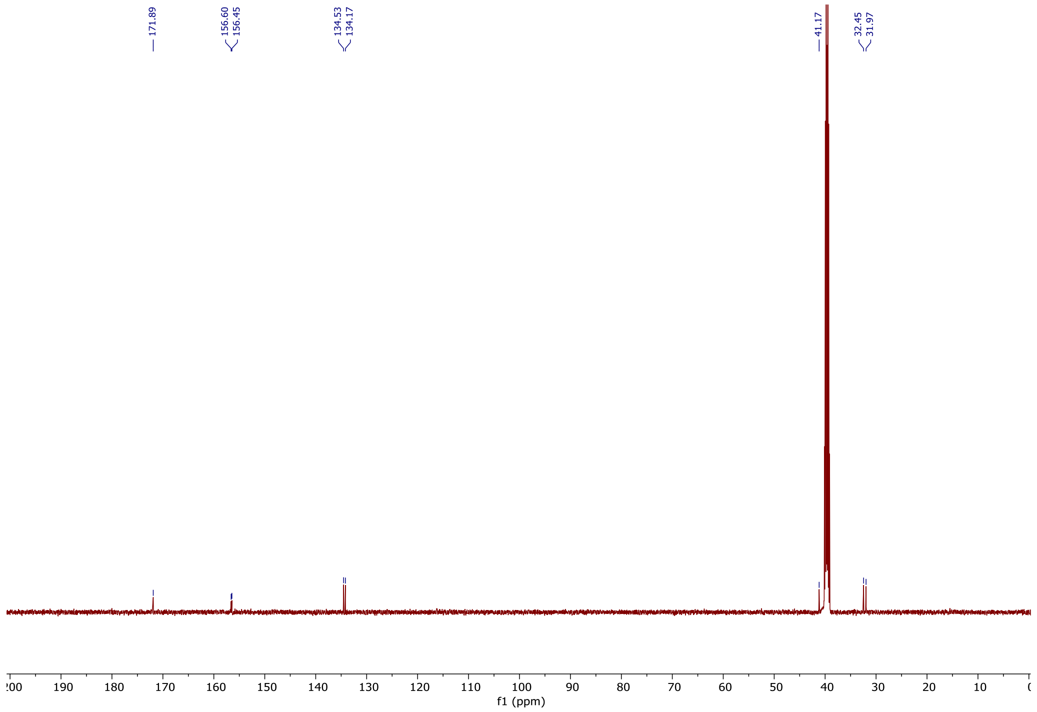
171.89
156.60
156.45
134.53
134.17
41.17
32.45
31.97
f1 (ppm)

((1*R*,8*S*,9*s*)-Bicyclo[6.1.0]non-4-yn-9-yl)methyl (2-(2-(2-(3-(2-methyl-3,6-dioxo-3,6-dihydropyridazin-1(2*H*)-yl)propanamido)ethoxy)ethoxy)ethyl)carbamate 27

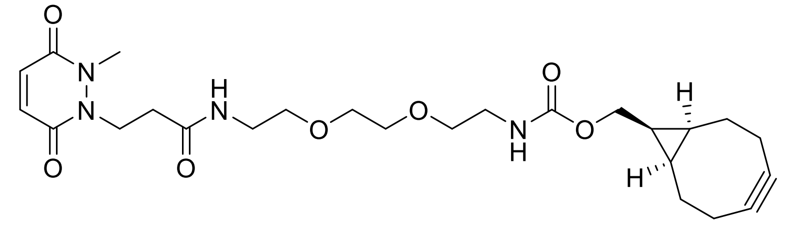

27

To a solution of 3-(2-methyl-3,6-dioxo-3,6-dihydropyridazin-1(2*H*)-yl)propanoic acid **S5** (28 mg, 0.14 mmol) in THF (5 mL), was added 1-ethyl-3-carbodiimide hydrochloride (31 mg, 0.16 mmol). The heterogeneous reaction mixture was left to stir at 0 °C for 30 min. After this time, *N*-[(1*R*,8*S*,9*S*)-bicyclo[6.1.0]non-4-yn-9-ylmethyloxycarbonyl]-1,8-diamino-3,6-dioxaoctane (50 mg, 0.16 mmol), pre-dissolved in THF (1 mL), was added, followed by the dropwise addition of *N*,*N*-diisopropylethylamine (27 µL, 0.14 mmol). The flask was then flushed with argon and the reaction mixture was stirred at 21 °C for 16 h. Following this, the THF was removed *in vacuo* and the crude residue partitioned between $CHCl_3$ (50 mL) and water (30 mL). The organic layer was washed with water (2 × 30 mL) and saturated aq. K_2CO_3 (30 mL), dried over $MgSO_4$ and concentrated *in vacuo.* Purification of the crude residue by flash column chromatography (0% to 20% MeOH/EtOAc) afforded ((1*R*,8*S*,9*S*)-bicyclo[6.1.0]non-4-yn-9-yl)methyl(2-(2-(2-(3-(2-methyl-3,6-dioxo-3,6-dihydropyridazin-1(2*H*) yl)propanamido)ethoxy)ethoxy)ethyl)carbamate **27** (43 mg, 0.085 mmol, 64%) as an orange oil.

^{1}H NMR (500 MHz, $CDCl_3$) δ 7.69 (s, 0.2H), 6.88 (q, *J* = 10.2 Hz, 2H), 6.36 (s, 0.7H), 5.79 (s, 0.3H), 5.33 (s, 0.7H), 4.38 (t, *J* = 7.2 Hz, 2H), 4.14 (dd, *J* = 8.1 Hz, 2H), 3.71 – 3.29 (m, 16H), 2.59 (t, *J* = 7.1 Hz, 2H), 2.35 – 2.13 (m, 6H), 1.24 (s, 5H), 0.94 (d, *J* = 9.2 Hz, 2H); **^{13}C NMR** (150 MHz, $CDCl_3$) δ 169.3 (C), 157.3 (C), 157.2 (C), 157.0 (C), 135.0 (C), 134.3 (C), 99.0 (CH), 96.3 (CH), 70.4 (CH), 70.3 (CH), 69.7 (CH), 63.0 (CH_2), 42.7 (CH_2), 40.9 (CH_2), 39.5 (CH_2), 34.4 (CH_2), 32.1 (CH_2), 30.4 (CH_2), 30.1 (CH_2), 29.8 (CH_2), 29.5 (CH_2), 29.2 (CH_2), 22.8 (CH_2), 21.6 (CH_2), 20.2 (CH_3), 17.9 (CH_2), 14.3 (CH_2); **IR** (thin film) 3311, 3073, 2917, 2850, 1708, 1629, 1537, 1247 cm^{-1}; **LRMS** (ESI) 505 (100, $[M+H]^+$). **HRMS** (ESI) calcd for $C_{25}H_{37}N_4O_7$ $[M+H]^+$, 505.2657 observed 502.2652.

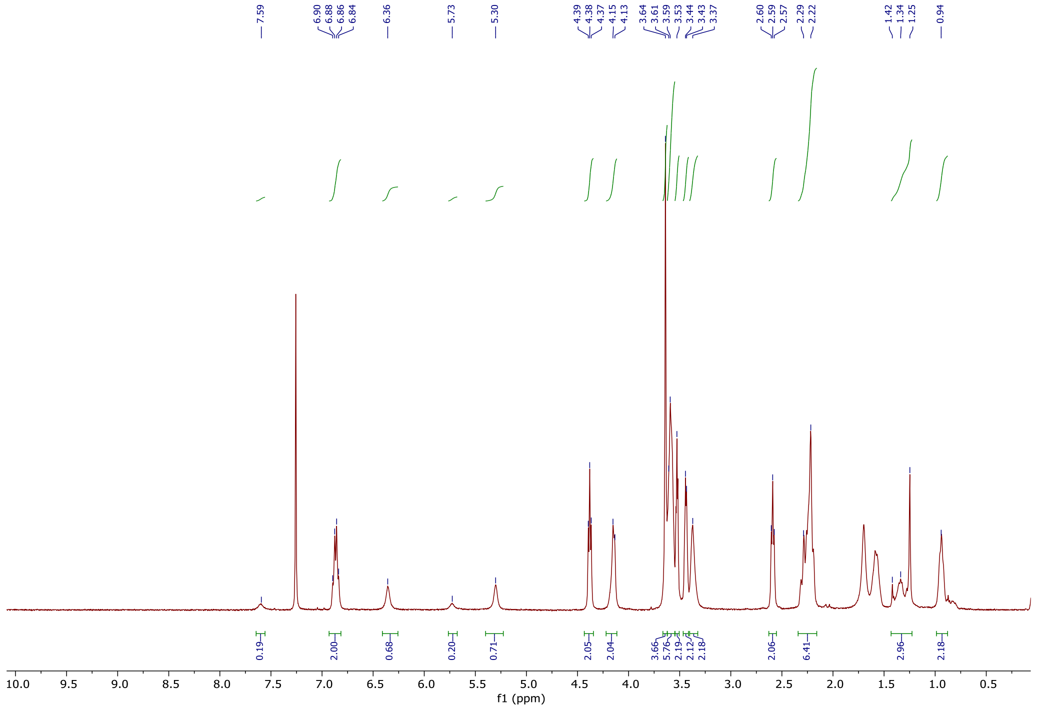
7.59
6.90
6.88
6.86
6.84
6.36
5.73
5.30
4.39
4.38
4.37
4.15
4.13
3.64
3.61
3.59
3.53
3.44
3.43
3.37
2.60
2.59
2.57
2.29
2.22
1.42
1.34
1.25
0.94
0.19
2.00
0.68
0.20
0.71
2.05
2.04
3.66
5.76
2.19
2.12
2.18
2.06
6.41
2.96
2.18
10.0 9.5 9.0 8.5 8.0 7.5 7.0 6.5 6.0 5.5 5.0 4.5 4.0 3.5 3.0 2.5 2.0 1.5 1.0 0.5
f1 (ppm)

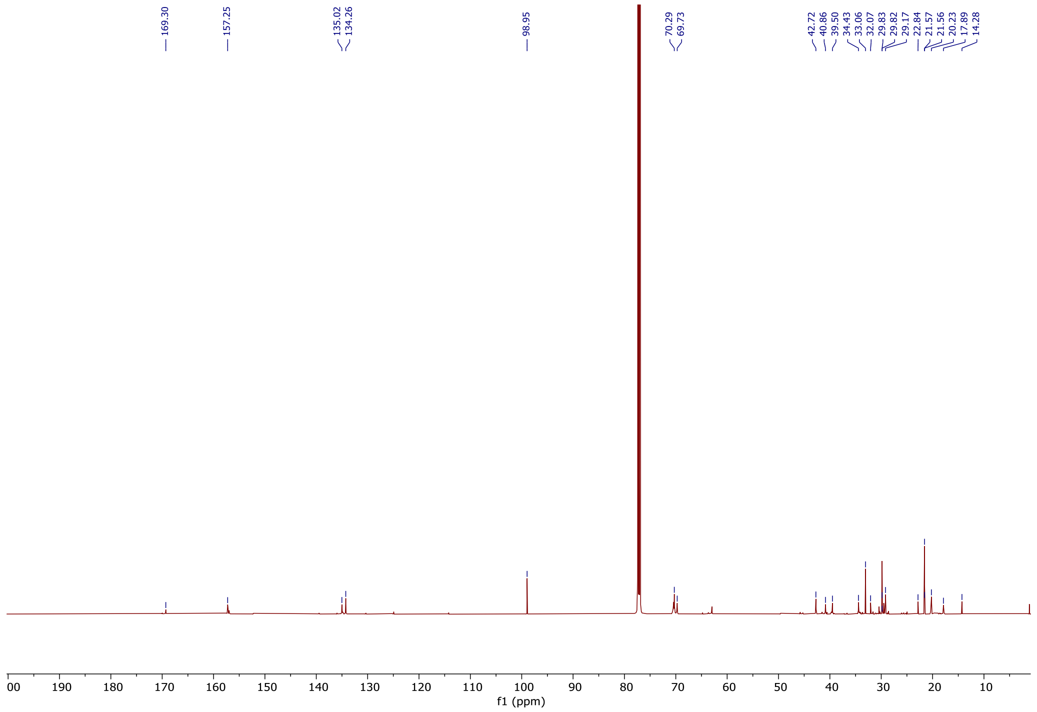
169.30
157.25
135.02
134.26
98.95
70.29
69.73
42.72
40.86
39.50
34.43
33.06
32.07
29.83
29.82
29.17
22.84
21.57
21.56
20.23
17.89
14.28
00 190 180 170 160 150 140 130 120 110 100 90 80 70 60 50 40 30 20 10
f1 (ppm)

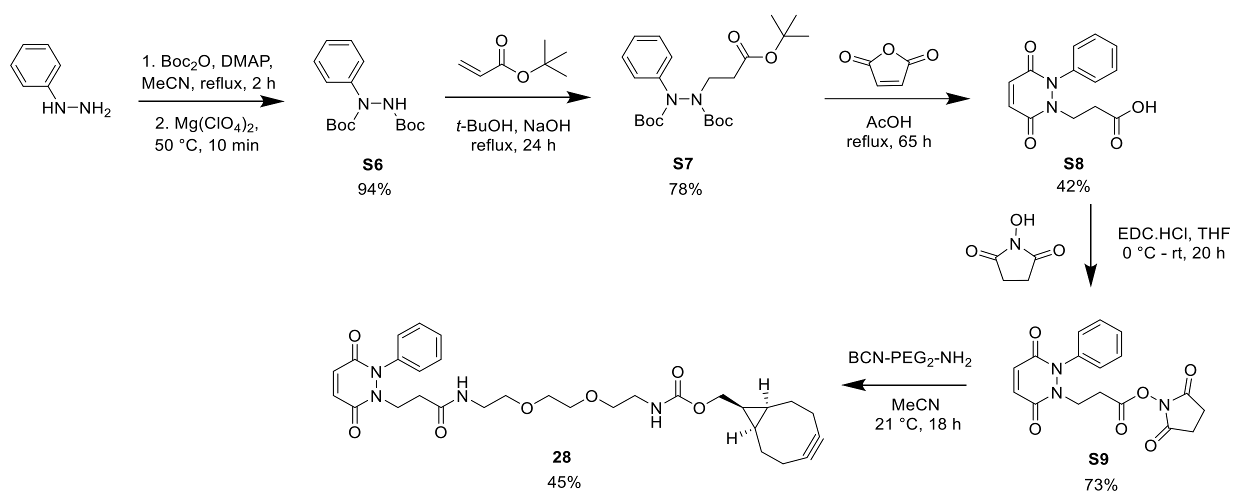

*Scheme S3. Synthesis of N-Ph,N'-BCN PD **28***

Di-*tert*-butyl 1-phenylhydrazine-1,2-dicarboxylate S6

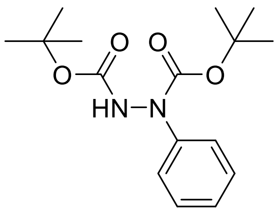

S6

To a solution of phenylhydrazine (0.99 mL, 10 mmol) in MeCN (27 mL), was added di-*tert*-butyl dicarbonate (9.2 g, 42 mmol) and 4-dimethylaminopyridine (5 mg, 0.042 mmol), and the reaction was stirred at 60 °C for 2 h. After this time, the reaction temperature was reduced to 50 °C, and to the solution was added magnesium perchlorate (0.45 g, 2 mmol), and the reaction was left to stir at 50 °C for 10 min. The reaction mixture was then cooled to 21 °C and then quenched by the addition of a 1:3 mixture $Na_2S_2O_4$ (sat. aq.)/brine (100 mL). The resulting biphasic solution was extracted into EtOAc (2 × 100 mL). The combined organics were washed with sat. aq. $NaHCO_3$ (150 mL) and brine (150 mL), dried over Na_2SO_4 and concentrated *in vacuo*. The crude product was slurried in EtOH at -10 °C and then filtered to afford di-*tert*-butyl 1-phenylhydrazine-1,2-dicarboxylate as a white solid **S6** (2.9 g, 9.4 mmol, 94%).

^{1}H NMR (600 MHz, $CDCl_3$, rotamers) δ 7.41 (s, 2H), 7.31 (t, *J* = 7.8 Hz, 2H), 7.15 (t, *J* = 7.4 Hz, 1H), 6.76 (s, 1H), 1.49 (s, 18H); **^{13}C NMR** (150 MHz, $CDCl_3$) δ 155.5 (C), 153.8 (C), 142.3 (C), 128.6 (CH), 125.7 (CH), 123.8 (CH), 82.4 (C), 81.7 (C), 28.3 (CH_3); **IR** (solid) 3283, 2980, 1713, 1509, 1249, 1148 cm^{-1}; **LRMS** (ESI) 153 (83, $[M-_tBu-Boc+H]^+$), 197 (17, $[M-2t\text{Bu}+H]^+$); **HRMS** (ESI) calcd for $C_{16}H_{25}N_2O_4$ $[M+H]^+$ 309.1809 observed 309.1801.

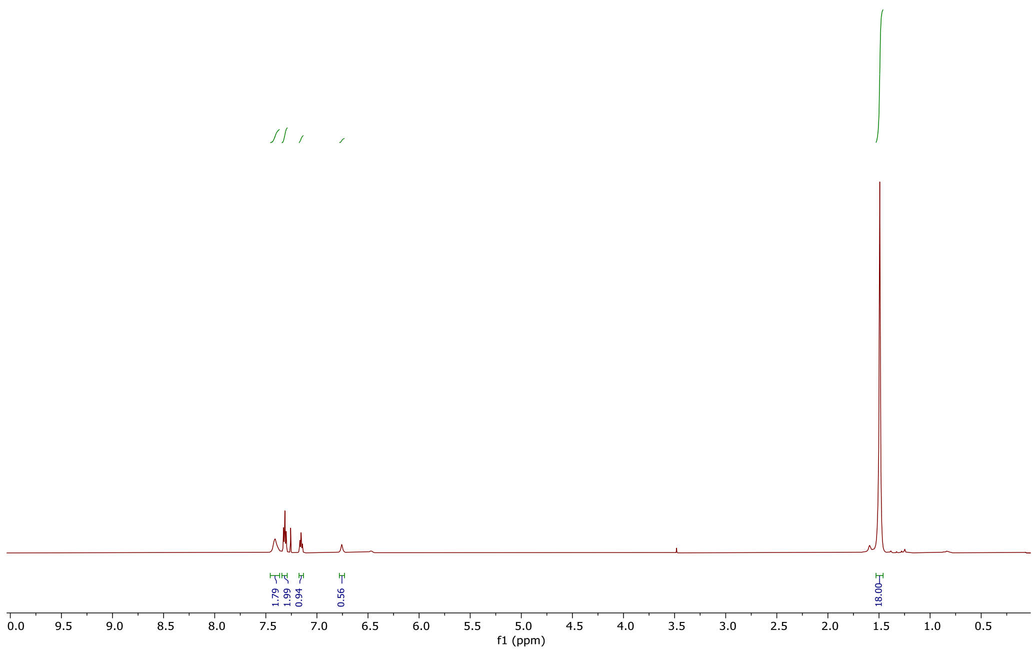
1.79
1.99
0.94
0.56
18.00
0.0 9.5 9.0 8.5 8.0 7.5 7.0 6.5 6.0 5.5 5.0 4.5 4.0 3.5 3.0 2.5 2.0 1.5 1.0 0.5
f1 (ppm)

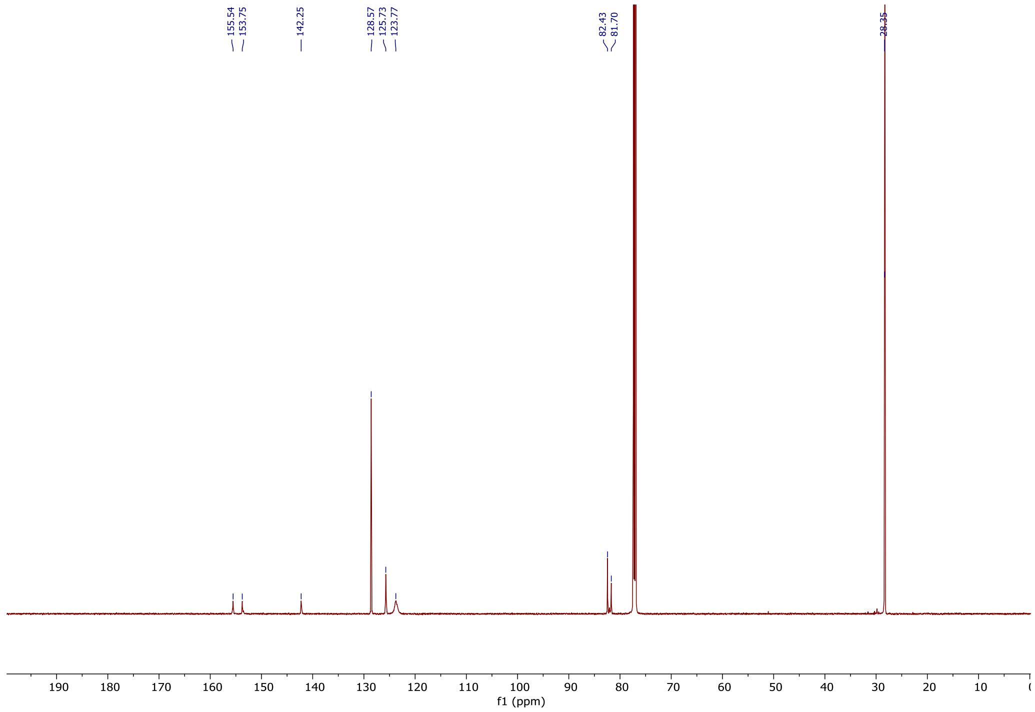
155.54
153.75
142.25
128.57
125.73
123.77
82.43
81.70
28.35
190 180 170 160 150 140 130 120 110 100 90 80 70 60 50 40 30 20 10
f1 (ppm)

Di-*tert*-butyl 1-(3-(*tert*-butoxy)-3-oxopropyl)-2-phenylhydrazine-1,2-dicarboxylate S7

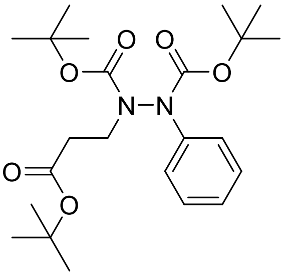

S7

To a solution of di-*tert*-butyl-1-phenylhydrazine-1,2-dicarboxylate **S6** (500 mg, 1.62 mmol) in *tert*-butanol (2.6 mL), was added 2 M NaOH (0.05 mL) and the reaction mixture was stirred at 21 °C for 10 min. After this time, to the solution, was added *tert*-butyl acrylate (0.706 mL, 4.86 mmol) and the reaction mixture was heated at 80 °C for 72 h. After this time, the solvent was removed *in vacuo.* The crude residue was then dissolved in EtOAc (100 mL), washed with water (3 × 50 mL), dried over Na_2SO_4 and concentrated *in vacuo* to afford di-*tert*-butyl 1-(3-(*tert*-butoxy)-3-oxopropyl)-2-phenylhydrazine-1,2-dicarboxylate **S7** (549 mg, 1.26 mmol, 78%) as a yellow oil.

^{1}H NMR (600 MHz, $CDCl_3$) δ 7.32 (dt, *J* = 15.1, 8.1 Hz, 4H), 7.17 – 7.11 (m, 1H), 3.79 – 3.66 (m, 2H), 2.59 – 2.44 (m, 2H), 1.53 (d, 9H), 1.47 (d, 9H), 1.38 (d, *J* = 7.3 Hz, 9H); **^{13}C NMR** (150 MHz, $CDCl_3$) δ 170.9 (C), 153.0 (C), 141.1 (C), 128.6 (CH), 125.5 (CH), 122.5 (CH), 82.3 (C), 81.9 (C), 46.7 (C), 45.5 (C), 33.9 (C), 28.4 (CH_2), 28.4 (CH_3), 28.4 (CH_3), 28.3 (CH_2), 28.2 (CH_3); **IR** (thin film) 3370, 2977, 2931, 1722, 1368, 1152 cm^{-1}; **LRMS** (ESI) 437 (100, $[M+H]^+$); **HRMS** (ESI) calcd for $C_{23}H_{36}N_2O_6$ $[M+H]^+$ 437.2646 observed 437.2640.

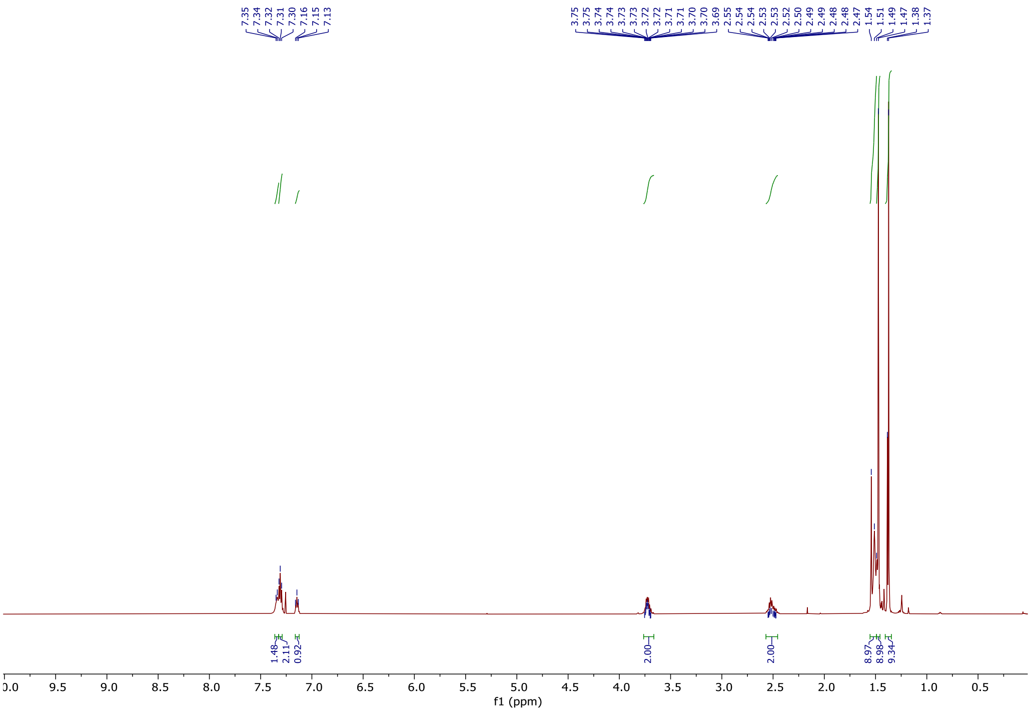

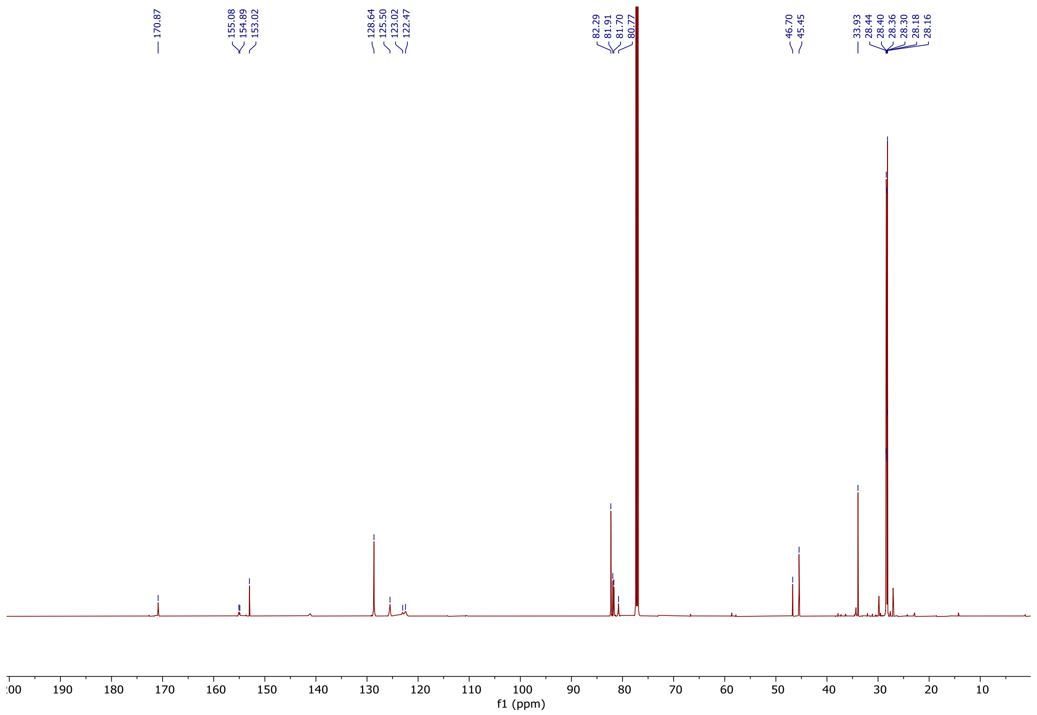

170.87
155.08
154.89
153.02
128.64
125.50
123.02
122.47
82.29
81.91
81.70
80.77
46.70
45.45
33.93
28.44
28.40
28.36
28.30
28.18
28.16
200
190
180
170
160
150
140
130
120
110
100
90
80
70
60
50
40
30
20
10
f1 (ppm)

3-(3,6-Dioxo-2-phenyl-3,6-dihydropyridazin-1(2*H*)-yl)propanoic acid S8

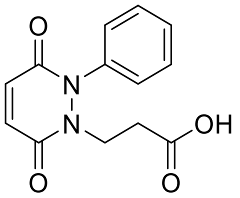

S8

Maleic anhydride (76 mg, 0.78 mmol) was dissolved in AcOH (5 mL) and heated under reflux for 30 min. After this time, to this solution, was added di-*tert*-butyl-1-(3-(tert-butoxy)-3-oxopropyl)-2-phenylhydrazine-1,2-dicarboxylate **S7** (200 mg, 0.46 mmol) and the reaction heated under reflux for 16 h. Following this, the reaction mixture was concentrated *in vacuo* with toluene co-evaporation (3 × 30 mL, as an azeotrope) and the crude residue purified by flash column chromatography (0 to 20% MeOH/EtOAc (1% AcOH)) to afford 3-(3,6-dioxo-2-phenyl-3,6-dihydropyridazin-1(2*H*)-yl)propanoic acid **S8** (50 mg, 0.19 mmol, 42%) as a white solid.

^{1}H NMR (600 MHz, DMSO-d$_6$) δ 12.39 (s, 1H), 7.56 (t, 2H), 7.50 (t, 3H), 7.07 (d, *J* = 10.1 Hz, 1H), 7.02 (d, *J* = 10.1 Hz, 1H), 3.72 (t, *J* = 7.5 Hz, 2H), 2.39 (t, *J* = 7.4 Hz, 2H); **^{13}C NMR** (150 MHz, DMSO-d$_6$) δ 171.6 (C), 157.3 (C), 157.0 (C), 136.1 (C), 135.6 (CH), 135.0 (CH), 129.5 (CH), 129.4 (CH), 128.8 (CH), 42.2 (CH$_2$), 31.2 (CH$_2$); **IR** (solid) 3174, 2969, 1704, 1620, 1414 cm^{-1}. **LRMS** (ESI) 259 (100, [M-H]$^-$); **HRMS** (ESI) calcd for $C_{13}H_{11}N_2O_4$ [M-H]$^-$ 259.0724 observed 259.0724.

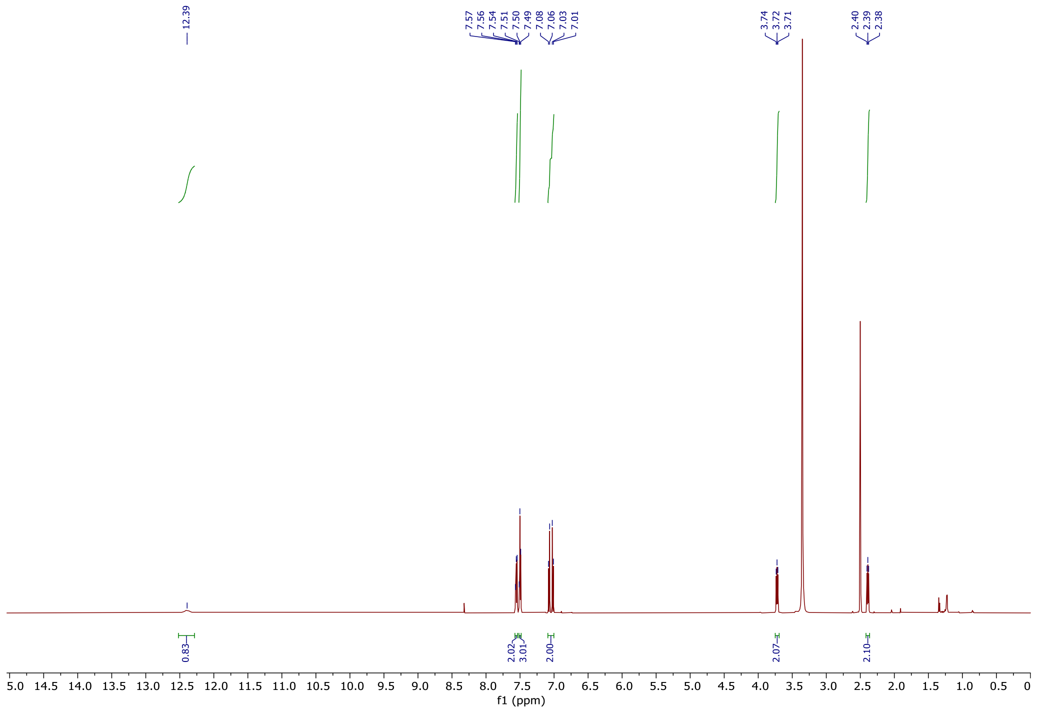

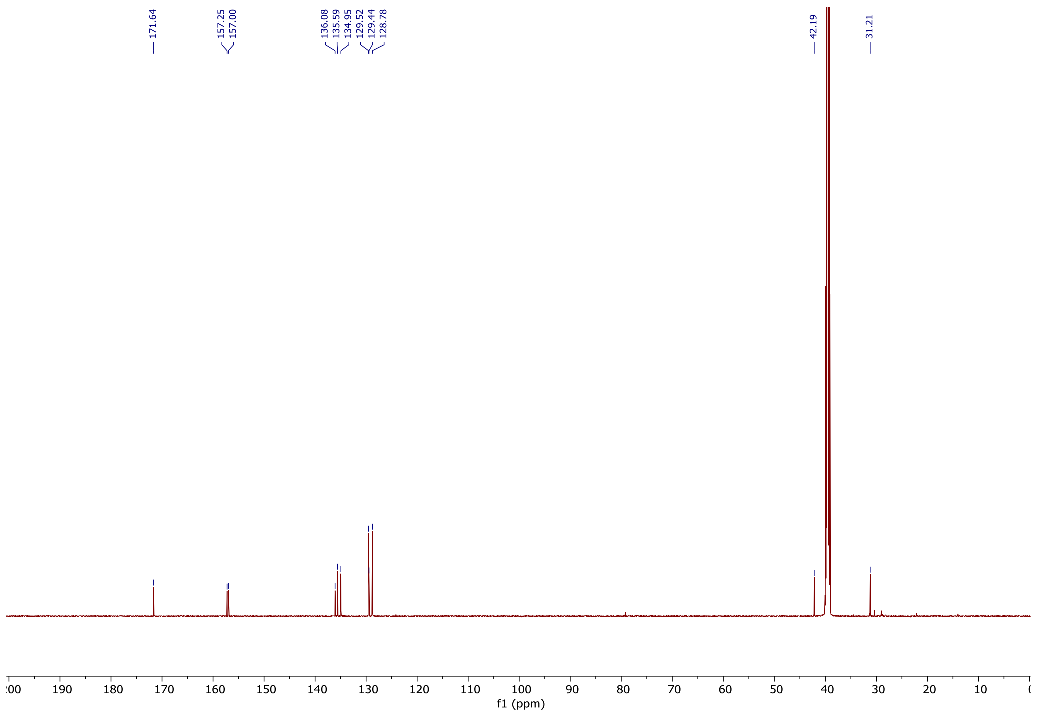
171.64
157.25
157.00
136.08
135.59
134.95
129.52
129.44
128.78
42.19
31.21
f1 (ppm)

2,5-Dioxopyrrolidin-1-yl 3-(3,6-dioxo-2-phenyl-3,6-dihydropyridazin-1(2*H*)-yl)propanoate S9

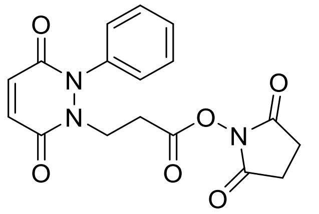

S9

To a solution of 3-(3,6-dioxo-2-phenyl-3,6-dihydropyridazin-1(2*H*)-yl)propanoic acid **S8** (45 mg, 0.27 mmol) in THF (6.2 mL), pre-cooled to 0 °C, was added 1-ethyl-3-carbodiimide hydrochloride (36.46 mg, 0.19 mmol, 1.1 equiv.). The homogenous solution was then stirred at 0 °C for 30 min. Following this, to the solution, was added *N*-hydroxysuccinimide (30 mg, 0.26 mmol) and the reaction stirred at 21 °C for 16 h. The newly formed heterogenous mixture was then filtered through a celite pad, washed with EtOAc (3 × 10 mL) and the filtrate was concentrated *in vacuo*. Purification of the crude residue by flash column chromatography (50% to 100% EtOAc/cHex) afforded 2,5-dioxopyrrolidin-1-yl 3-(3,6-dioxo-2-phenyl-3,6-dihydropyridazin-1(2*H*)-yl)propanoate **S9** (45 mg, 0.13 mmol, 73%) as a beige solid.

^{1}H NMR (600 MHz, $CDCl_3$) δ 7.55 (t, *J* = 7.6 Hz, 2H), 7.49 (t, *J* = 7.5 Hz, 1H), 7.37 (d, *J* = 8.6 Hz, 2H), 6.97 (dd, 2H), 4.05 (t, *J* = 7.1 Hz, 2H), 2.87 (t, *J* = 7.1 Hz, 2H), 2.80 (s, 4H); **^{13}C NMR** (151 MHz, $CDCl_3$) δ 168.8 (C), 165.7 (C), 158.0 (C), 157.8 (C), 135.8 (CH), 135.7 (CH), 135.4 (CH), 130.2 (CH), 130.1 (CH), 128.3 (CH), 128.2 (CH), 42.1 (CH_2), 28.6 (CH_2), 25.7 (CH_2); **IR** (solid) 3359, 3069, 2978, 1702, 1618, 1508 cm^{-1}; **LRMS** (ESI) 358 (6, $[M+H]^+$, 283 (30, $[MC_{13}H_{12}N_2O_4+Na]^+$), 261 (64, $[MC_{13}H_{12}N_2O_4+H]^+$); **HRMS** (ESI) calcd for $C_{17}H_{16}N_3O_6$ $[M+H]^+$ 358.1034, observed 358.1030.

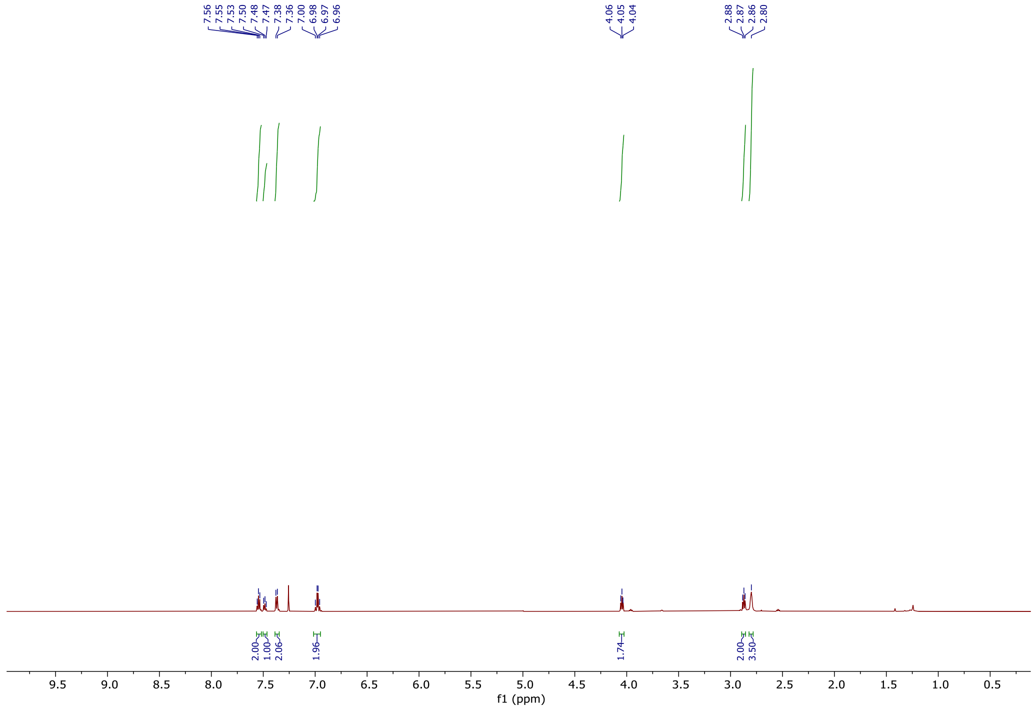
7.56
7.55
7.53
7.50
7.48
7.47
7.38
7.36
7.00
6.98
6.97
6.96
4.06
4.05
4.04
2.88
2.87
2.86
2.80
2.00
1.00
2.06
1.96
1.74
2.00
3.50
f1 (ppm)

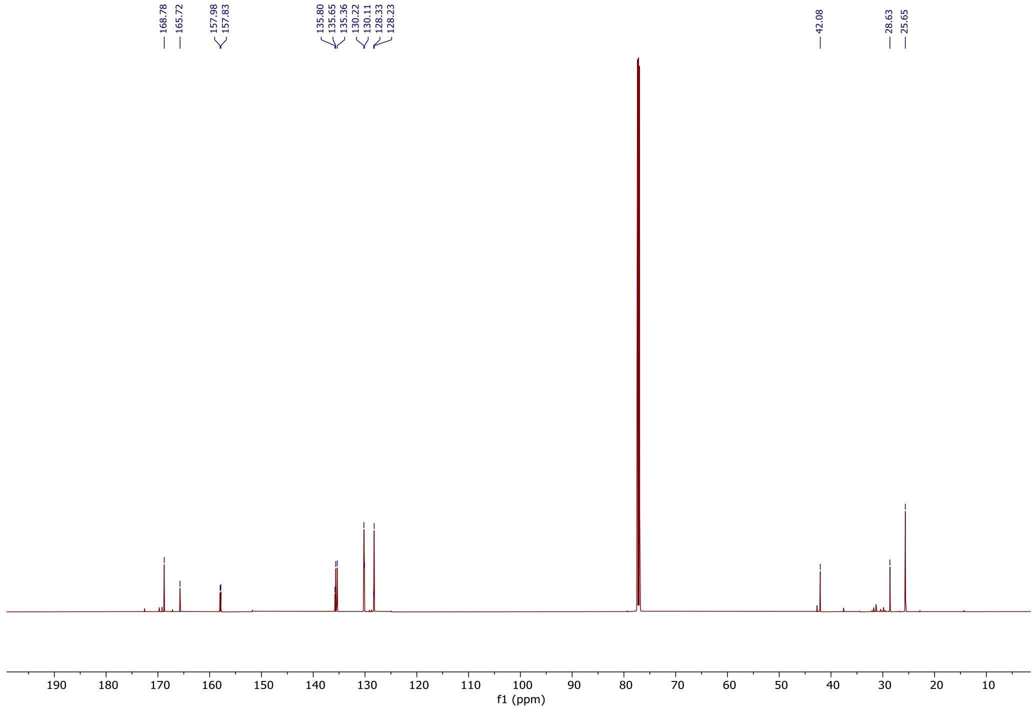
168.78
165.72
157.98
157.83
135.80
135.65
135.36
130.22
130.11
128.33
128.23
42.08
28.63
25.65
f1 (ppm)

((1*R*,8*S*,9*S*)-Bicyclo[6.1.0]non-4-yn-9-yl)methyl (2-(2-(2-(3-(3,6-dioxo-2-phenyl-3,6-dihydropyridazin-1(2*H*)-yl)propanamido)ethoxy)ethoxy)ethyl)carbamate 28

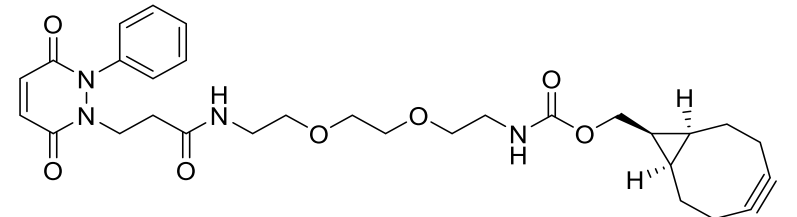

28

To a solution of 2,5-dioxopyrrolidin-1-yl-3-(3,6-dioxo-2-phenyl-3,6-dihydropyridazin-1(2*H*)-yl)propanoate **S9** (33.92 mg, 0.095 mmol) in MeCN (6.2 mL), was added *N*-[(1*R*,8*S*,9*S*)-bicyclo[6.1.0]non-4-yn-9-ylmethyloxycarbonyl]-1,8-diamino-3,6-dioxaoctane (30.8 mg, 0.095 mmol), and the reaction was stirred at 21 °C for 16 h. After this time, the MeCN was removed *in vacuo* and the crude residue was partitioned between $CHCl_3$ (50 mL) and water (30 mL). The organic layer was washed with water (30 mL) and sat. aq. K2CO3 (30 mL), dried over MgSO4 and concentrated *in vacuo*. Purification of the crude residue *via* flash column chromatography (0% to 15% MeOH/EtOAc) afforded ((1*R*,8*S*,9*S*)-bicyclo[6.1.0]non-4-yn-9-yl)phenyl(2-(2-(2-(3-(2-methyl-3,6-dioxo-3,6-dihydropyridazin-1(2*H*)yl)propanamido)ethoxy) ethoxy)ethyl)carbamate **28** (24 mg, 0.042 mmol, 45%) as an orange oil.

^{1}H NMR (600 MHz, $CDCl_3$) δ 7.54 (t, *J* = 7.6 Hz, 2H), 7.48 (t, *J* = 7.8 Hz, 1H), 7.37 (d, *J* = 7.7 Hz, 2H), 6.97 (dd, *J* = 10.1, 10.1 Hz, 2H), 6.33 (s, 0.6H), 5.35 (s, 0.6H), 4.22 – 4.10 (m, 2H), 3.97 (t, *J* = 7.3 Hz, 2H), 3.66 – 3-58 (m, 6H), 3.49 (t, *J* = 5.0 Hz, 2H), 3.38-3.36 (m, 4H), 2.42 (t, *J* = 7.4 Hz, 2H), 2.31 – 2.19 (m, 6H), 1.42 – 1.24 (m, 4H), 0.93 (t, *J* = 9.9 Hz, 2H); **^{13}C NMR** (151 MHz, $CDCl_3$) δ 171.8 (C), 169.2 (C), 157.9 (C), 157.0 (C), 135.8 (CH), 135.4 (CH), 130.1 (C), 128.4 (CH), 99.0 (C), 70.3 (CH), 69.8 (CH), 63.0 (CH), 43.9 (CH), 40.9 (CH_2), 39.4 (CH_2), 33.9 (CH_2), 29.8 (CH_2), 29.2 (CH_2), 25.6 (CH_2), 22.8 (CH_2), 21.6 (CH_2), 20.2 (CH_2), 17.9 (CH_2), 14.3 (CH); **IR** (thin film) 3318, 3068, 2918, 1640, 1528, 1248 cm^{-1}; **LRMS** (ESI) 567 (100, $[M+H]^+$); **HRMS** (ESI) calcd for $C_{30}H_{39}N_4O_7$ $[M+H]^+$ 567.2813 observed 567.2810.

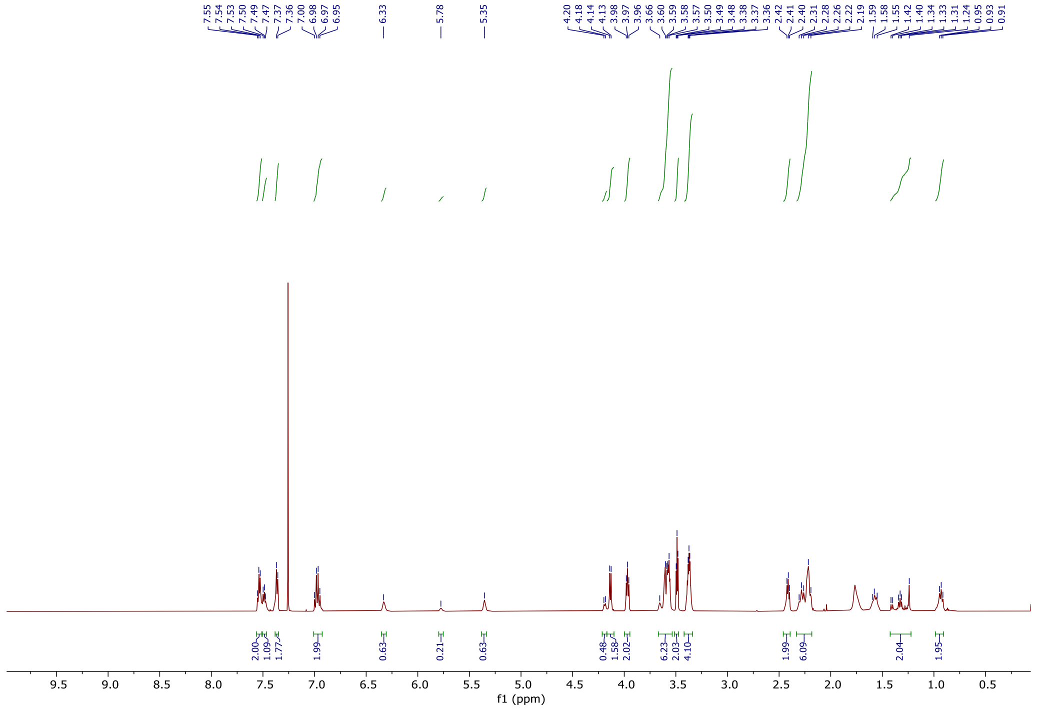

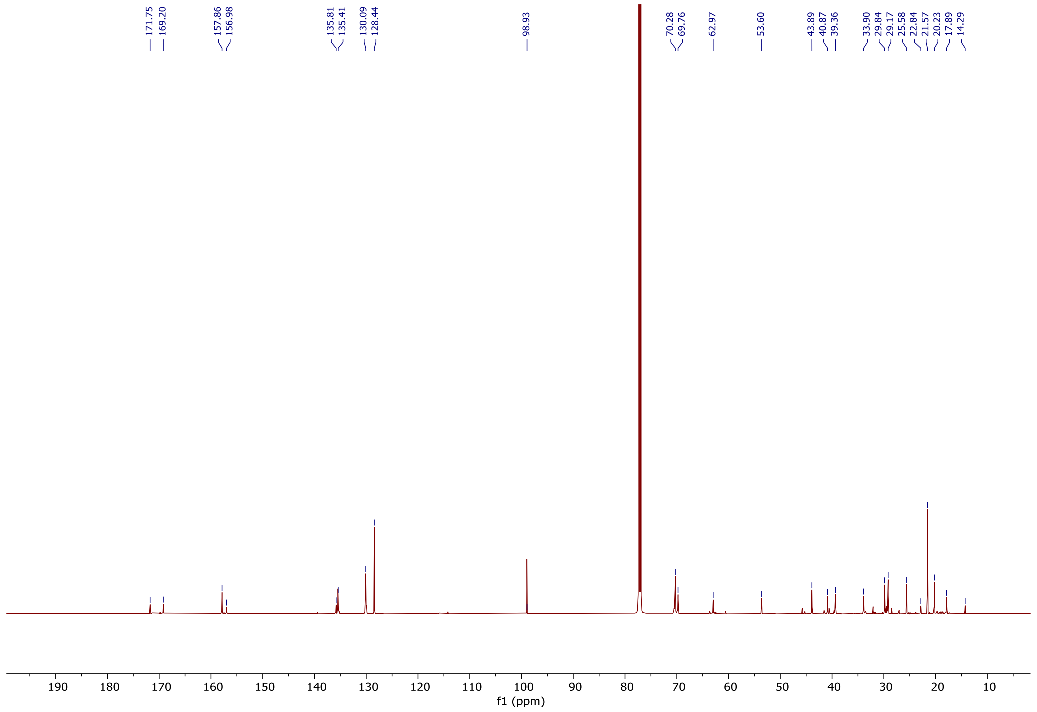

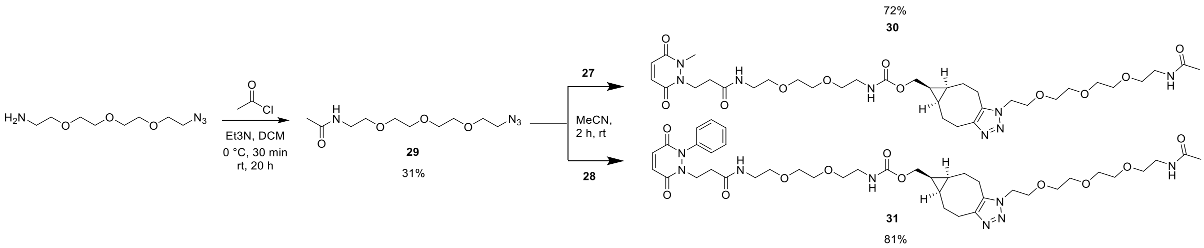

*Scheme S4. Synthesis of functionalised model PDs **30** and **31***

***N*-(*2*-(*2*-(*2*-(*2*-Azidoethoxy)ethoxy)ethoxy)ethyl)acetamide 29**

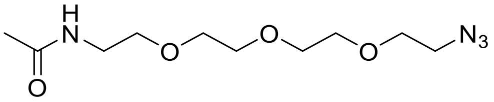

29

To a solution of 11-azido-3,6,9-trioxaundecan-1-amine (200 mg, 182 µL, 0.917 mmol) in DCM (2 mL) was added Et_3N (143 µL, 1.01 mmol) and the reaction mixture was cooled to 0 °C. Following this, to the solution, was added acetyl chloride (73 µL, 1.01 mmol), and the reaction mixture was left to stir at 0 °C for 30 min. The reaction mixture was then allowed to warm to 21 °C and left to stir for 20 h. After this time, the crude mixture was diluted by the addition of DCM (10 mL) and quenched with the addition of a solution of sat. aq. $NaHCO_3$ (10 mL). The product was then extracted into DCM (3 × 10 mL). The organics were then combined, washed with brine (30 mL) and dried over $MgSO_4$. Solvent was removed *in vacuo* before purification. Purification of the crude product *via* flash column chromatography (0% to 20% MeOH/EtOAc, dry load using DCM and celate, product eluted around 10%) afforded *N*-(*2*-(*2*-(*2*-(*2*-azidoethoxy)ethoxy)ethoxy)ethyl)acetamide **29** (74 mg, 0.284 mmol, 31%) as a colourless oil.

^{1}H NMR (500 MHz, CD_3OD) δ 3.68 – 3.65 (m, 8H), 3.62-3.61 (m, 2H), 3.54 (t, *J* = 5.5 Hz, 2H), 3.38 (t, *J* = 5.0 Hz, 2H), 3.35 (t, *J* = 5.5 Hz, 2H), 1.95 (s, 3H); **^{13}C NMR** (150 MHz, CD_3OD) δ 173.3 (C), 71.6 (CH_2), 71.6 (CH_2), 71.5 (CH_2), 71.3 (CH_2), 71.1 (CH_2), 70.5 (CH_2), 51.8 (CH_2), 40.5 (CH_2), 22.5 (CH_3); **IR** (thin film) 3298, 2916, 2864, 2100, 1654, 1451, 1105 cm^{-1}; **LRMS** (ESI) 261 (100, $[M+H]^+$); **HRMS** (ESI) calcd for $C_{10}H_{21}N_4O_4$ $[M+H]^+$ 261.1557, observed 261.1557.

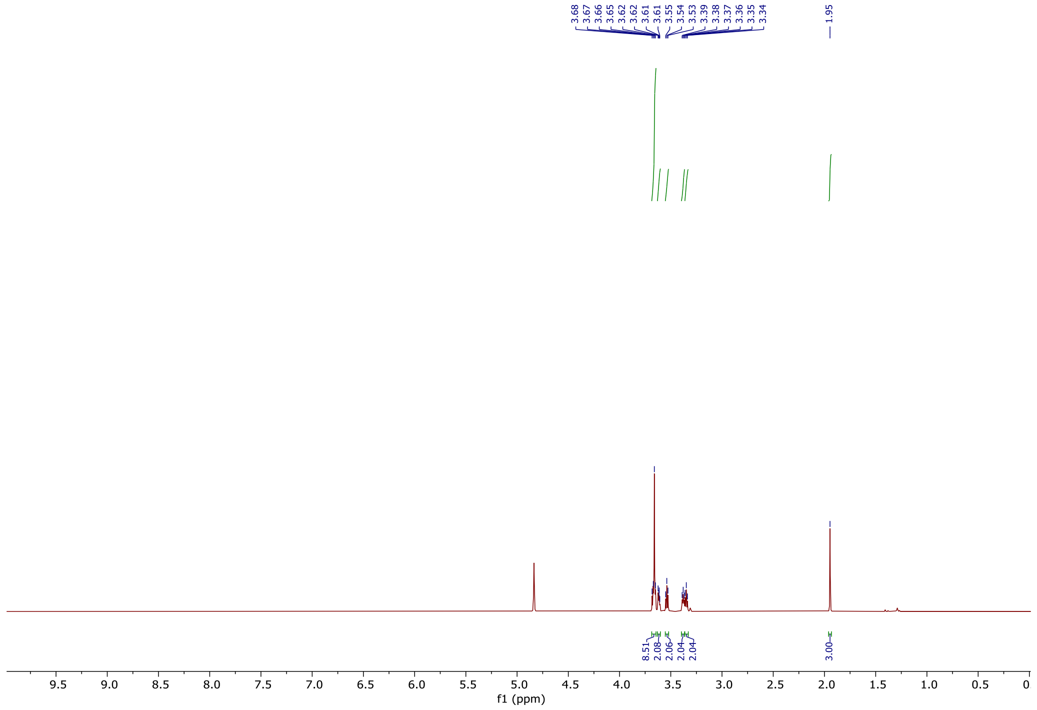

3.68
3.67
3.66
3.65
3.62
3.62
3.61
3.61
3.55
3.54
3.53
3.39
3.38
3.37
3.36
3.35
3.34
1.95
8.51
2.08
2.06
2.04
2.04
3.00
9.5
9.0
8.5
8.0
7.5
7.0
6.5
6.0
5.5
5.0
4.5
4.0
3.5
3.0
2.5
2.0
1.5
1.0
0.5
f1 (ppm)

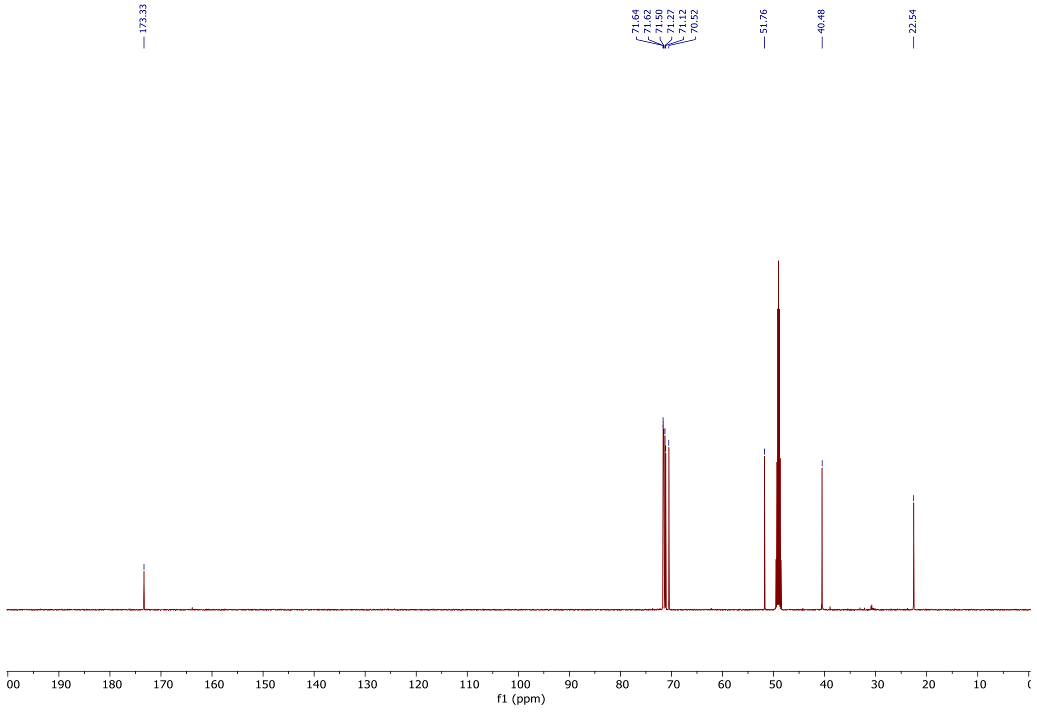

173.33
71.64
71.62
71.50
71.27
71.12
70.52
51.76
40.48
22.54
190
180
170
160
150
140
130
120
110
100
90
80
70
60
50
40
30
20
10
f1 (ppm)

((5a*R*,6*S*,6a*S*)-1-(2-Oxo-6,9,12-trioxa-3-azatetradecan-14-yl)-1,4,5,5a,6,6a,7,8-octahydrocyclopropa[5,6]cycloocta[1,2-*d*][1,2,3]triazol-6-yl)methyl (2-(2-(2-(3-(2-methyl-3,6-dioxo-3,6-dihydropyridazin-1(2*H*)yl)propanamido)ethoxy)ethoxy)ethyl)carbamate 30

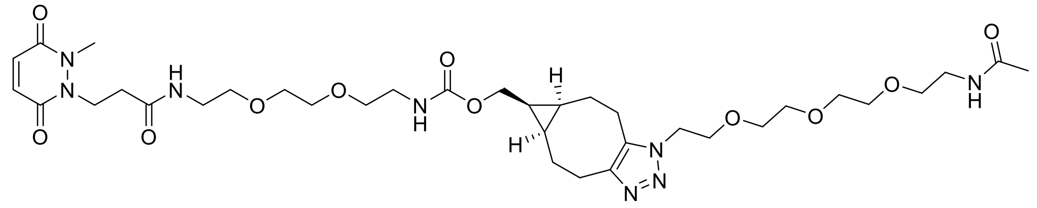

30

To a solution of *N*-(*2*-(*2*-(*2*-(*2*-azidoethoxy)ethoxy)ethoxy)ethyl)acetamide **29** (10 mg, 0.038 mmol) in MeCN (1 mL), was added ((1*R*,8*S*,9*S*)-bicyclo[6.1.0]non-4-yn-9-yl)methyl(2-(2-(2-(3-(2-methyl-3,6-dioxo-3,6-dihydropyridazin-1(2*H*)-yl)propanamido)ethoxy)ethoxy)ethyl)carbamate **27** (20 mg, 0.038 mmol) in MeCN (1 mL). The reaction mixture was left to stir at 21 °C for 2 h under air. After this time, solvent was removed *in vacuo* and the crude product was purified using flash chromatography (reverse phase column, 0 to 100% acetonitrile/water) to afford ((5a*R*,6*S*,6a*S*)-1-(2-oxo-6,9,12-trioxa-3-azatetradecan-14-yl)-1,4,5,5a,6,6a,7,8-octahydrocyclopropa[5,6]cycloocta[1,2-*d*][1,2,3]triazol-6-yl)methyl(2-(2-(2-(3-(2-methyl-3,6-dioxo-3,6-dihydropyridazin-1(2*H*)yl)propanamido)ethoxy)ethoxy)ethyl)carbamate **30** (21 mg, 0.028 mmol, 72%) as a colourless oil.

^{1}H NMR (500 MHz, CD_3Cl) δ 6.89-6.83 (m, 2H), 6.62 (m, 0.6H), 6.43 (m, 0.8H), 5.43 (m, 0.6H), 4.40-4.35 (m, 4H), 4.15-4.09 (m, 2H), 3.86 (t, *J* = 5.2 Hz, 2H), 3.63 (m, 3H), 3.59-3.51 (m, 17H), 3.42 (t, *J* = 5.3 Hz, 4H), 3.36 (t, *J* = 5.3 Hz, 2H), 3.11-3.07 (m, 1H), 3.00-2.95 (m, 1H), 2.89-2.84 (m, 1H), 2.73-2.67 (m, 1H), 2.60-2.57 (m, 2H), 2.23-2.19 (m, 2H), 2.07 (s, 3H), 1.97 (s, 3H) 1.57-1.55 (m, 2H), 1.22-1.18 (m, 1H), 1.03-1.01 (m, 2H); **^{13}C NMR** (150 MHz, CD_3Cl) δ 170.5 (C), 169.4 (C), 157.3 (C), 157.0 (C), 144.5 (C), 135.0 (C), 134.3 (CH), 70.7 (CH_2), 70.6 (CH_2), 70.6 (CH_2), 70.3 (CH_2), 70.2 (CH_2), 70.0 (CH_2), 69.8 (CH_2), 62.8 (CH_2), 47.9 (CH), 42.7 (CH_2), 40.9 (CH_2), 39.4 (CH_2), 34.4 (CH_2), 33.1 (CH_2), 26.0 (CH_3), 23.3 (CH_2), 23.2 (CH_2), 22.8 (CH_2), 22.3 (CH_2), 20.1 (CH_2), 19.6 (CH_3), 17.9 (CH); **IR** (thin film) 3311, 3069, 2918, 2867, 1640, 1250 cm^{-1}; **LRMS** (ESI) 765 (100, $[M+H]^+$); **HRMS** (ESI) calcd for $C_{35}H_{56}N_8O_{11}$ $[M+H]^+$ 765.4141, observed 765.4141.

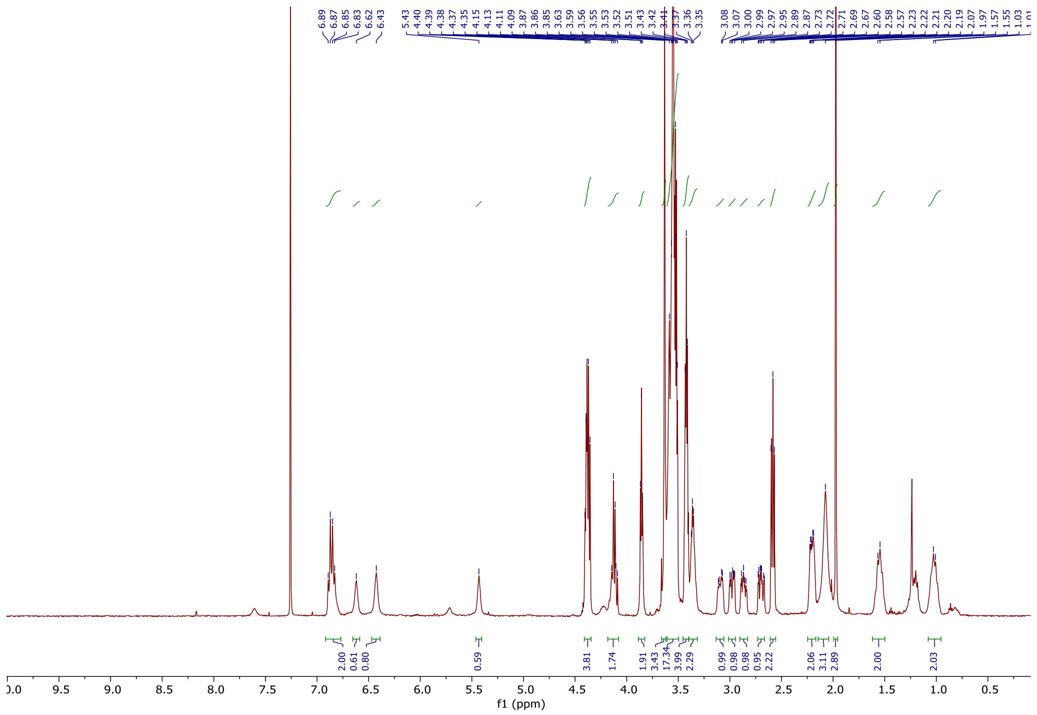

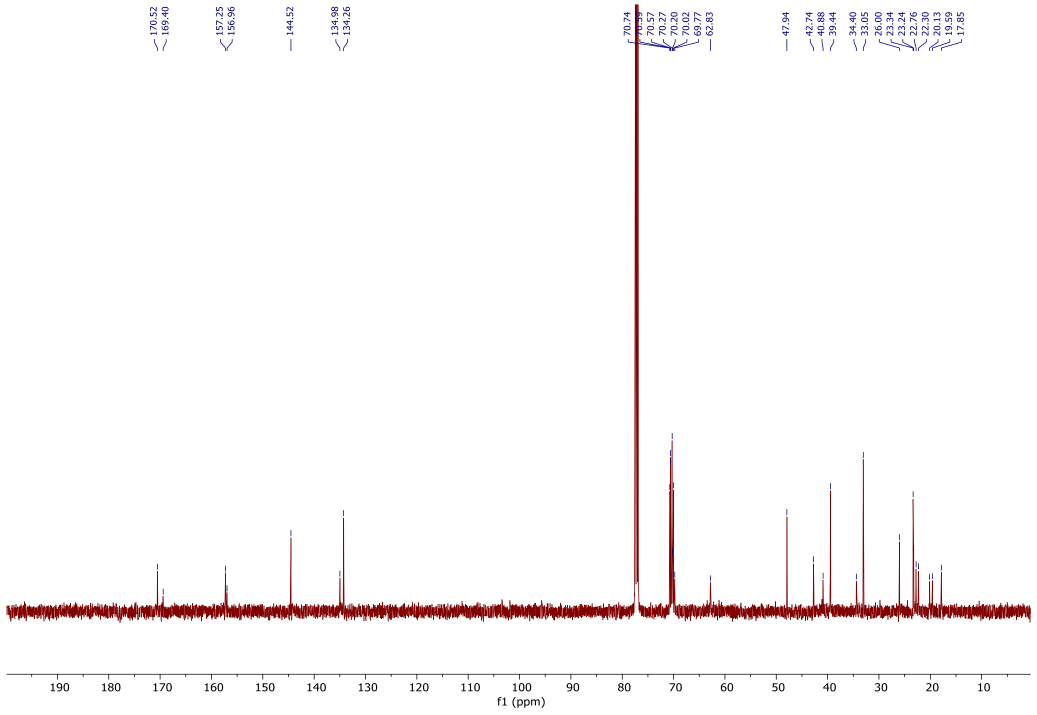

((5a*R*,6*S*,6a*S*)-1-(2-Oxo-6,9,12-trioxa-3-azatetradecan-14-yl)-1,4,5,5a,6,6a,7,8-octahydrocyclopropa[5,6]cycloocta[1,2-*d*][1,2,3]triazol-6-yl)methyl (2-(2-(2-(3-(3,6-dioxo-2-phenyl-3,6-dihydropyridazin-1(2*H*)-yl)propanamido)ethoxy)ethoxy)ethyl)carbamate 31

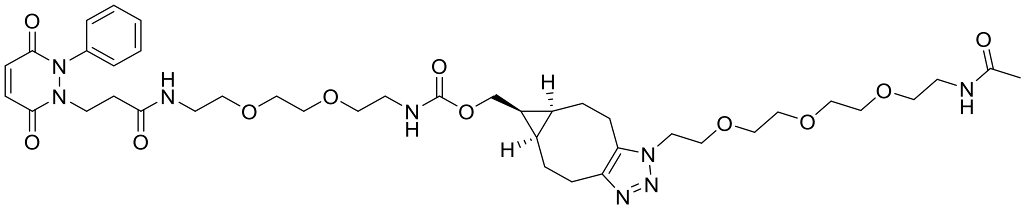

31

To a solution of *N*-(*2*-(*2*-(*2*-(*2*-azidoethoxy)ethoxy)ethoxy)ethyl)acetamide **29** (10 mg, 0.038 mmol) in MeCN (1 mL), was added ((1*R*,8*S*,9*S*)-bicyclo[6.1.0]non-4-yn-9-yl)phenyl(2-(2-(2-(3-(2-methyl-3,6-dioxo-3,6-dihydropyridazin-1(2*H*)yl)propanamido)ethoxy) ethoxy)ethyl)carbamate **28** (22 mg, 0.038 mmol) in MeCN (1 mL). The reaction mixture was left to stir at 21 °C for 2 h. After this time, the solvent was removed *in vacuo* and the crude residue was purified using flash chromatography (reverse phase column, 0 to 100% acetonitrile/water) to afford ((5a*R*,6*S*,6a*S*)-1-(2-oxo-6,9,12-trioxa-3-azatetradecan-14-yl)-1,4,5,5a,6,6a,7,8-octahydrocyclopropa[5,6]cycloocta[1,2-*d*][1,2,3]triazol-6-yl)methyl(2-(2-(2-(3-(3,6-dioxo-2-phenyl-3,6-dihydropyridazin-1(2*H*)-yl)propanamido)ethoxy)ethoxy)ethyl)carbamate **31** (26 mg, 0.031 mmol, 81%) as a yellow oil.

^{1}H NMR (500 MHz, CD_3Cl) δ 7.54-7.52 (m, 2H), 7.49-7.46 (m, 1H), 7.36-7.35 (m, 2H), 6.99-6.94 (m, 2H), 6.60 (m, 1H), 6.54 (m, 1H), 5.76 (m, 1H), 4.40-4.38 (m, 2H), 4.13-4.10 (m, 2H), 3.96 (t, *J* = 5.2 Hz, 2H), 3.85 (t, *J* = 5.5 Hz, 2H), 3.59-3.52 (m, 16H), 3.48 (t, *J* = 5.3 Hz, 2H), 3.42 (q, *J* = 5.3 Hz, 2H), 3.37-3.35 (m, 4H), 3.10-3.07 (m, 1H), 2.99-2.95 (m, 1H), 2.88-2.83 (m, 1H), 2.71-2.67 (m, 1H), 2.41 (m, 2H), 2.22-2.19 (m, 2H), 1.97 (s, 3H) 1.58-1.52 (m, 2H), 1.07-1.02 (m, 2H); **^{13}C NMR** (150 MHz, CD_3Cl) δ 171.2 (C), 135.4 (C), 130.0 (C), 128.3 (C), 70.7 (CH_2), 70.5 (CH_2), 70.2 (CH_2), 70.1 (CH_2), 70.0 (CH_2), 60.4 (CH_2), 53.5 (CH_2), 50.9 (CH), 47.9 (CH), 43.8 (CH_2), 40.9 (CH_2), 40.8 (CH_2), 39.4 (CH_2), 39.3 (CH), 29.7 (CH_2), 25.9 (CH_3), 23.3 (CH_2), 23.2 (CH_2), 21.1 (CH_2), 14.2 (CH). **IR** (thin film) 3325, 3064, 2921, 1646, 1542, 1257 cm^{-1}; **LRMS** (ESI) 827 (100, $[M+H]^+$); **HRMS** (ESI) calcd for $C_{40}H_{59}N_8O_{11}$ $[M+H]^+$ 827.4294, observed 827.4298.

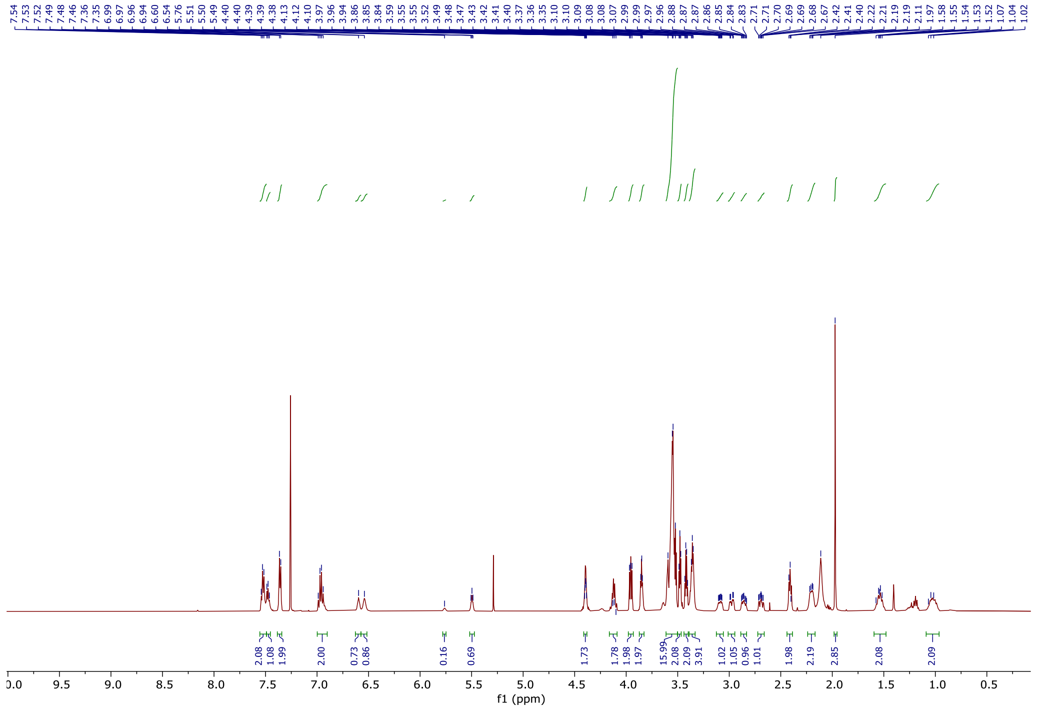

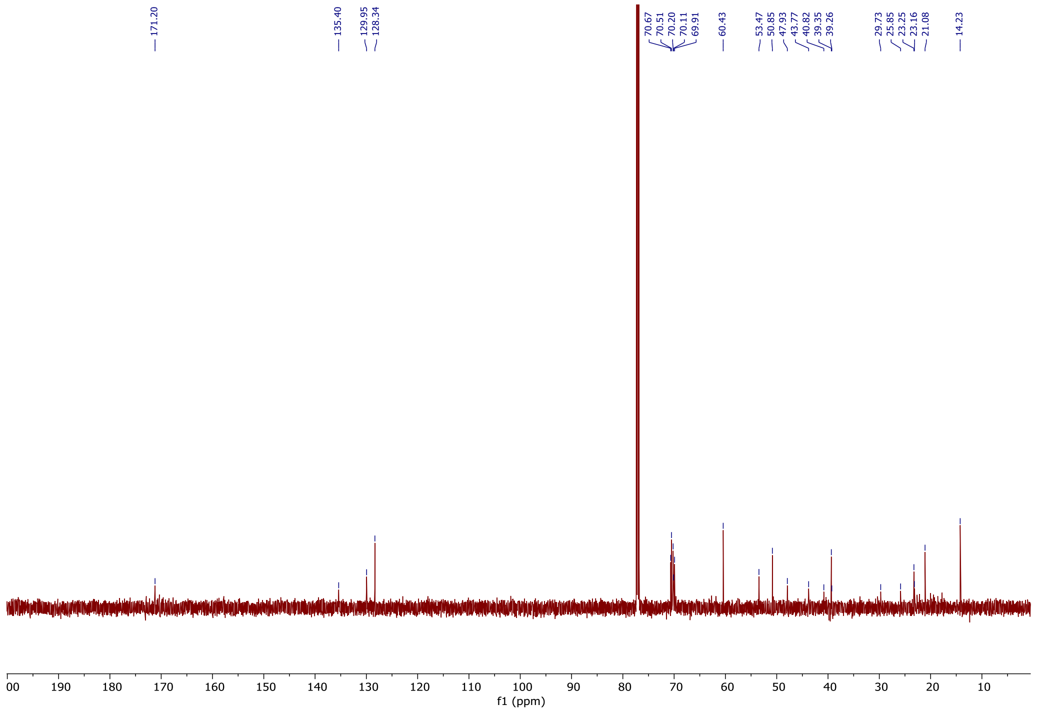

2.6 Disulfide rebridging reagents

4,5-Dibromo-1,2-diethyl-1,2-dihydropyridazine-3,6-dione 23[10, 11]

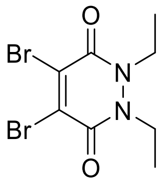

23

Dibromomaleic acid (274 mg, 1.00 mmol) was dissolved in AcOH (10 mL) and heated under reflux for 30 min. After this time, di-tert-butyl 1,2-diethylhydrazine-1,2-dicarboxylate obtained as previously described[11] (347 mg, 1.20 mmol) was added and the resultant mixture was heated under reflux for a further 4 h. After this time, the reaction mixture was concentrated *in vacuo* with toluene co-evaporation (3 × 20 mL, as an azeotrope). Purification of the crude residue by flash column chromatography (30% to 70% EtOAc/petrol) yielded 4,5-dibromo-1,2-diethyl-1,2-dihydropyridazine-3,6-dione **23** (267 mg, 0.819 mmol, 82%) as a yellow solid.

m.p. 110–115 °C; **^{1}H NMR** (500 MHz, $CDCl_3$) δ 4.17 (q, *J* = 7.1 Hz, 4H), 1.29 (t, *J* = 7.1 Hz, 6H); **^{13}C NMR** (125 MHz, $CDCl_3$) δ 153.2 (C), 136.1 (C), 42.4 (CH2), 13.1 (CH3); **IR** (solid) 2979, 2937, 2873, 1629, 1574 cm^{-1}.

4,5-Dibromo-1-ethyl-2-phenyl-1,2-dihydropyridazine-3,6-dione 24

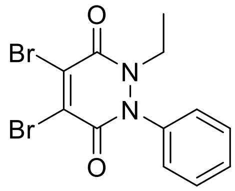

24

Dibromomaleic acid (274 mg, 1.00 mmol) was dissolved in AcOH (10 mL) and heated under reflux for 30 min. After this time, 1-ethyl-2-phenylhydrazine **S1** (163 mg, 1.20 mmol) was added, and the resultant mixture was heated under reflux for a further 5 h. After this time, the reaction mixture was concentrated *in vacuo* with toluene co-evaporation (3 × 20 mL, as an azeotrope). Purification of the crude residue by flash column chromatography (30% to 70% EtOAc/petrol) yielded 4,5-Dibromo-1-ethyl-2-phenyl-1,2-dihydropyridazine-3,6-dione **24** (267 mg, 0.819 mmol, 82%) as a beige solid.

^{1}H NMR (400 MHz, $CDCl_3$) δ 7.56-7.50 (m, 3H), 7.38 (d, *J* = 8.0 Hz, 2H), 3.82 (q, *J* = 7.0 Hz, 2H), 1.10 (t, *J* = 7.1 Hz, 3H); **^{13}C NMR** (125 MHz, $CDCl_3$) δ 153.4 (C), 152.9 (C), 137.2 (C), 136.2 (C), 135.9 (C), 130.2 (CH), 129.4 (CH), 129.9 (CH), 128.2 (CH), 44.1 (CH_2), 12.6 (CH_3); **IR** (solid) 3067, 2986, 2912, 2875, 1630, 1573, 699 cm^{-1}. **LRMS** (ESI) 377 (25, $[M^{81}Br^{81}Br+H]^+$) 375 (50, $[M^{79}Br^{81}Br+H]^+$), 373 (25, $[M^{79}Br^{79}Br+H]^+$). **HRMS** calcd for $C_{12}H_{11}Br_2N_2O_2$ $[M^{79}Br^{79}+H]^+$ 372.9182, observed 372.9181.

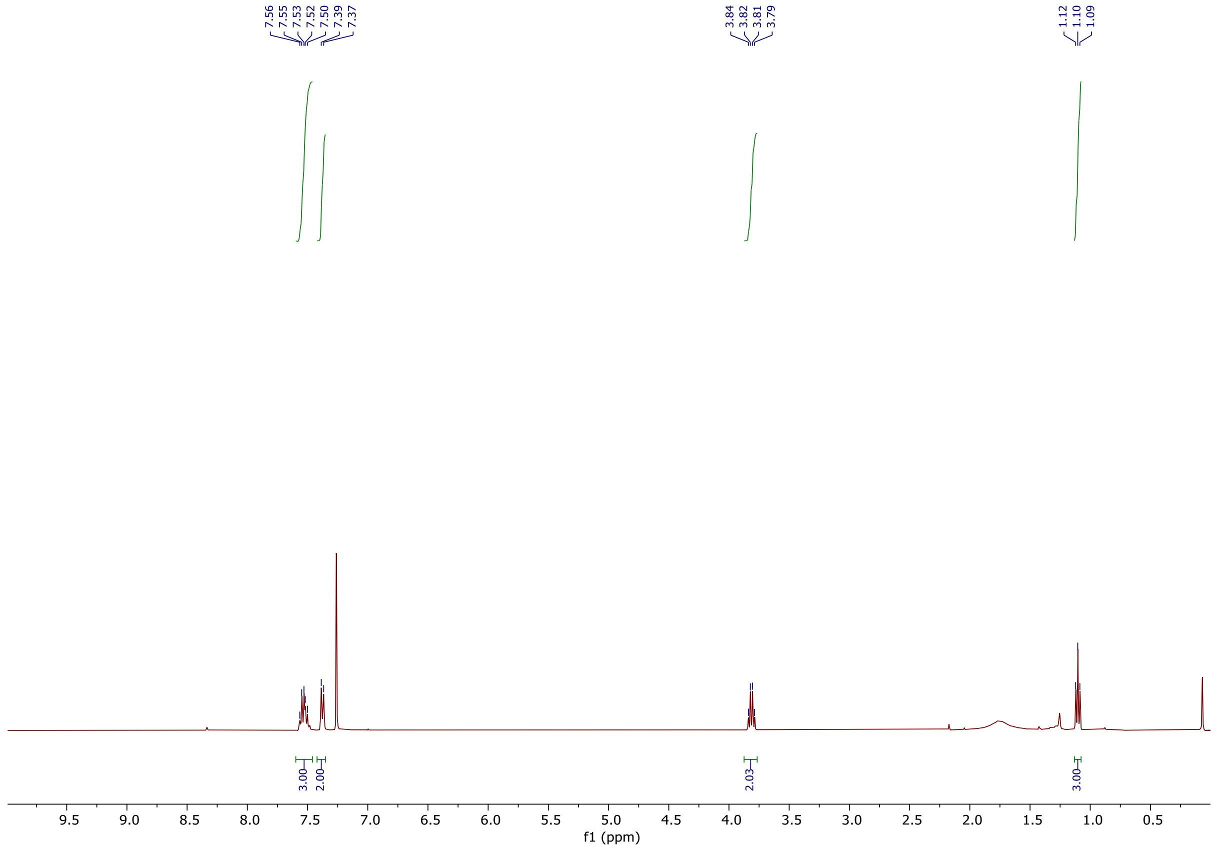
7.56
7.55
7.53
7.52
7.50
7.39
7.37
3.84
3.82
3.81
3.79
1.12
1.10
1.09
3.00
2.00
2.03
3.00
9.5 9.0 8.5 8.0 7.5 7.0 6.5 6.0 5.5 5.0 4.5 4.0 3.5 3.0 2.5 2.0 1.5 1.0 0.5
f1 (ppm)

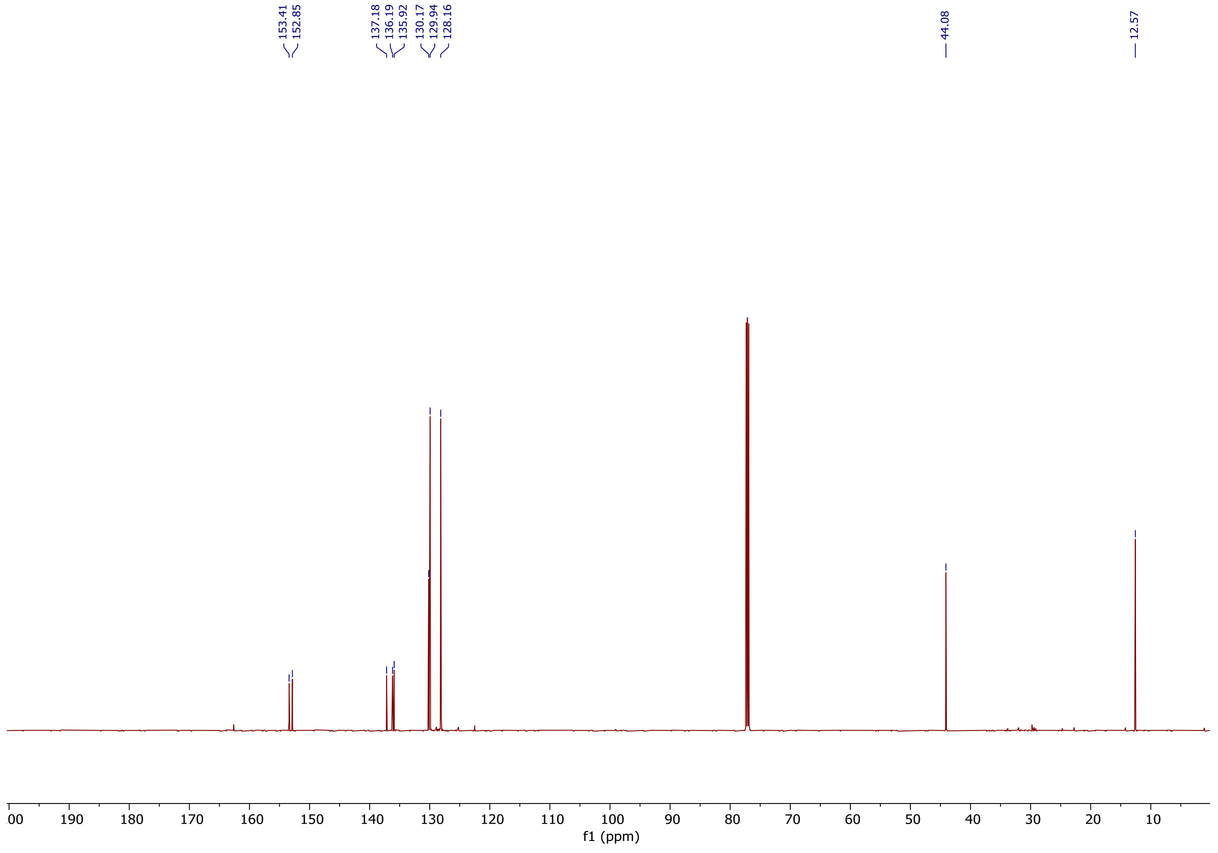
153.41
152.85
137.18
136.19
135.92
130.17
129.94
128.16
44.08
12.57
00 190 180 170 160 150 140 130 120 110 100 90 80 70 60 50 40 30 20 10
f1 (ppm)

4,5-Dibromo-1-ethyl-2-(perfluorophenyl)-1,2-dihydropyridazine-3,6-dione 42

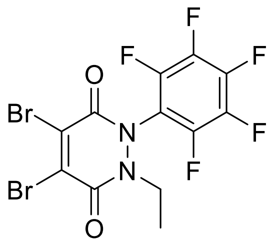

42

Dibromomaleic acid (78 mg, 0.28 mmol) was dissolved in AcOH (4 mL) and heated under reflux for 30 min. After this time, 1-ethyl-2-(perfluorophenyl)hydrazine **S3** (77 mg, 0.34 mmol) was added, and the resultant mixture was heated under reflux for a further 16 h. After this time, the reaction mixture was concentrated *in vacuo* with toluene co-evaporation (3 × 30 mL, as an azeotrope). Purification of the crude residue by flash column chromatography (0% to 50% EtOAc/cyclohexane) afforded 4,5-dibromo-1-ethyl-2-(perfluorophenyl)-1,2-dihydropyridazine-3,6-dione **42** (76 mg, 0.17 mmol, 51%) as a yellow solid.

^{1}H NMR (700 MHz, MeOD) δ 4.43 (t, *J* = 7.3 Hz, 2H), 3.68 (s, 3H), 2.73 (t, *J* = 7.3 Hz, 2H). **^{13}C NMR** (150 MHz, MeOD) δ 152.9 (C), 152.2 (C), 145.7 (C), 144.0 (C), 139.0 (C), 134.3 (C), 110.9 (C), 44.2 (CH_2), 12.4 (CH_3); **IR** (solid) 2917, 1649, 1512, 1280 cm^{-1}; **LRMS** (ESI) 467 (25, [$M^{81}Br^{81}Br+H]^+$) 465 (50, [$M^{79}Br^{81}Br+H]^+$), 463 (25, [$M^{79}Br^{79}Br+H]^+$). **HRMS** calcd for $C_{12}H_6Br_2F_5N_2O_2$ [$M^{79}Br^{81}+H]^+$ 464.8638, observed 464.8684.

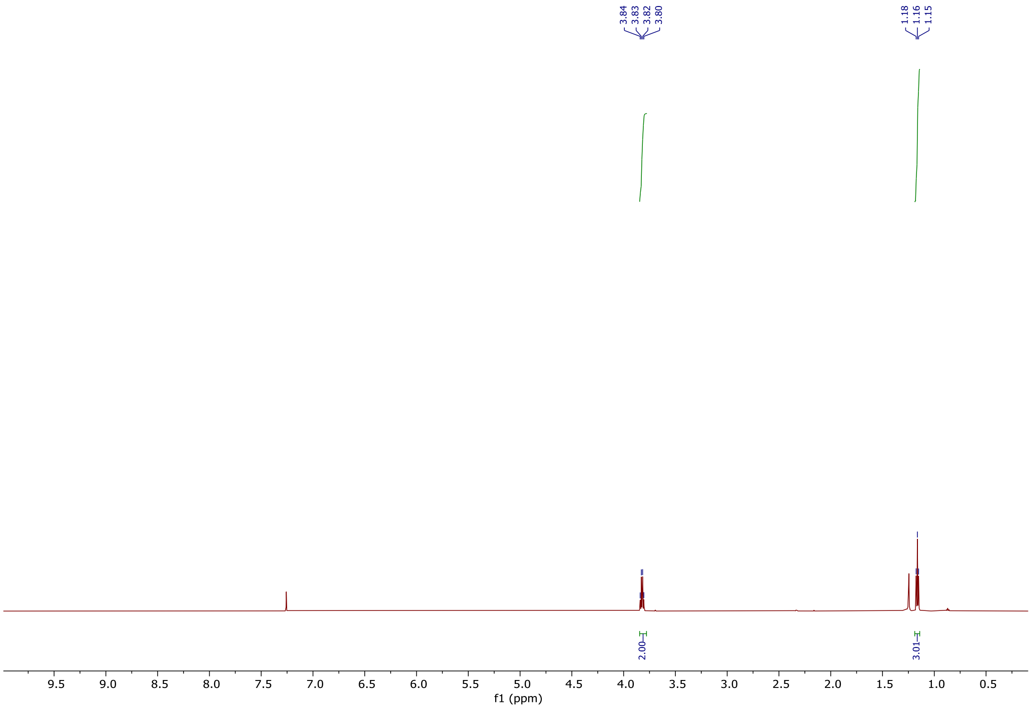

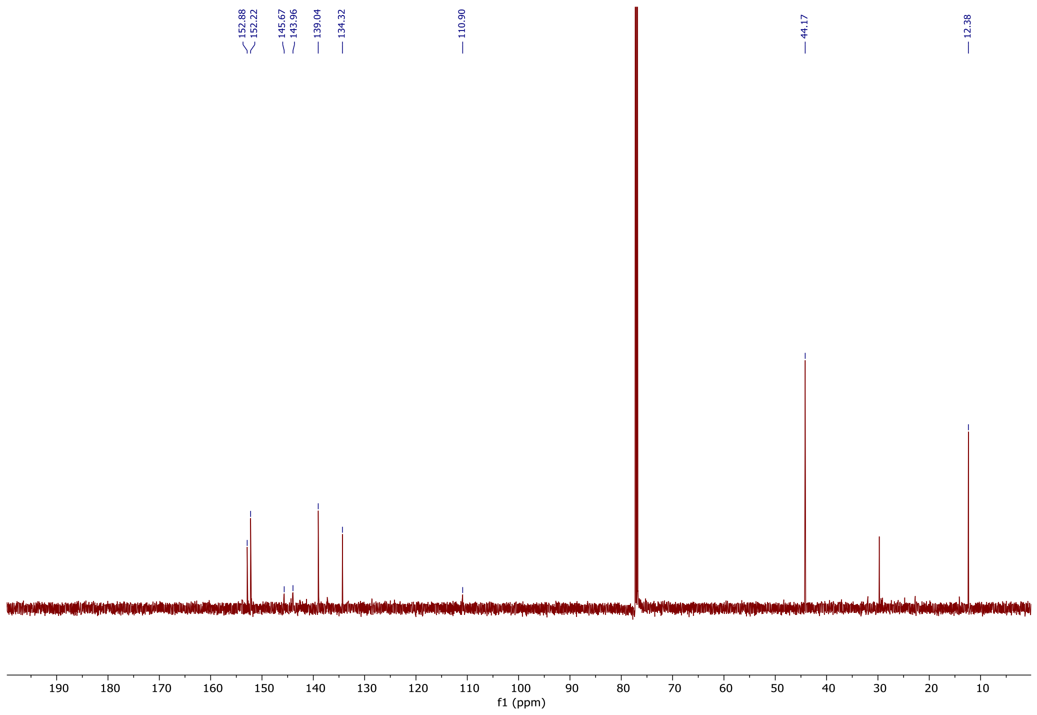
152.88
152.22
145.67
143.96
139.04
134.32
110.90
44.17
12.38
190 180 170 160 150 140 130 120 110 100 90 80 70 60 50 40 30 20 10
f1 (ppm)

3. *Kinetic assays on a peptide model*

3.1 *Extinction coefficients calculation*

A series of dilution for each PD **1-6, 30, 31** and each PD-cys **8-13** were carried out in MeCN to obtain the corresponding extinction coefficient following Beer-Lambert law. The correction factor of each PD at 280 nm was calculated, by estimating the average ratio of $\varepsilon_{280nm}/\varepsilon_{330nm}$ at each concentration that the absorbances were measured at (see Table S3).

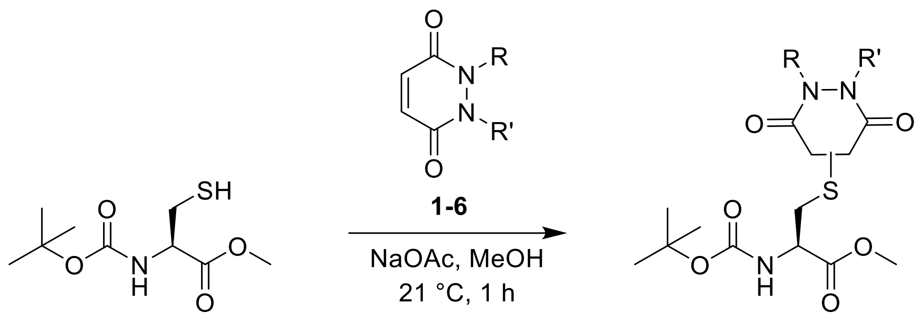

*Table S3. Yield of conjugation reaction, extinction coefficients of PD alone **1**-6 as well as the correction factor at 280 nm, and extinction coefficients of PD conjugated **8-13.** *This extinction coefficient prevented this PD kinetics to be studied using UV-Vis. ** CGY-PD **47** and **48** absorbances assessed in this case.*

R, R'	ε_{280nm} [PD] ($M^{-1}.cm^{-1}$)	ε_{330nm} [PD] ($M^{-1}.cm^{-1}$)	Correction factor 280 nm	Cys-PD conjugate	ε_{280nm} [cys-PD] ($M^{-1}.cm^{-1}$)	ε_{330nm} [cys-PD] ($M^{-1}.cm^{-1}$)
Et, Et **(1)**	516	2262	0.228	Et, Et **(8)**	345	7
Et, Ph **(3)**	769	2481	0.245	Et, Ph **(10)**	1211	57
Et, *p*-$PhNO_2$ **(5)**	3341	5427	0.616	Et, *p*-$PhNO_2$ **(12)**	2817	8059*
Et, *p*-$PhNH_2$ **(2)**	1508	2433	0.616	Et, *p*-$PhNH_2$ **(9)**	4578	51
Et, *p*-PhF **(4)**	822	3013	0.274	Et, *p*-PhF **(11)**	504	38
Et, PhF_5 **(6)**	1036	2874	0.365	Et, PhF_5 **(13)**	1081	41
Me, BCN clicked **(30)**	490	1754	0.228	Me, BCN clicked **(47)**	1699**	ca. 0**
Ph, BCN clicked **(31)**	682	2427	0.260	Ph, BCN clicked **(48)**	2328**	Ca. 0**

3.2 *Peptide oxidation study*

A solution of GCY **14** was prepared (1 mM, PB 100 mM, 5 mM EDTA, 3 mL) and was incubated at 37 °C for 90 min. Aliquots (50 µL) were taken at 15 min intervals, treated with DTNB solution (50 µL, 20 mM, MeCN, 20 equiv.) and left at 21 °C for 5 additional minutes before UV-Vis analysis. Absorbances A_{412} were obtained and used to calculate the free thiol concentration ([S-H]). For the qualitative analysis, percentage change in thiol concentration was plot against time, and significant oxidation was highlighted.

Compound	ε_{412}
TNB (from DTNB)	14150

	**Free Thiol Concentration [S-H] (M)**
**Time**	**CGY**
**0**	5.94E-04
**15**	5.30E-04
**30**	4.95E-04
**45**	4.73E-04
**60**	4.81E-04
**75**	4.73E-04
**90**	4.31E-04

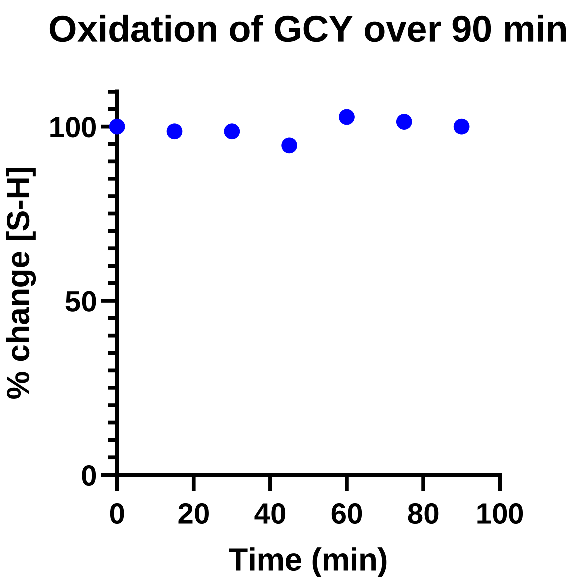

*Graph S1. Percentage of oxidation of GCY **14** after 90 min.*

3.3 Michael Addition kinetic assay

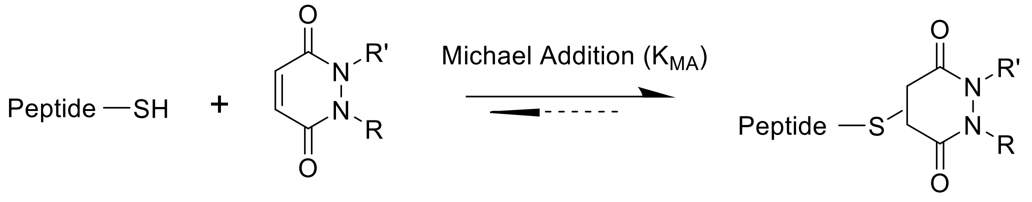

14 **1-4, 6, 30** and **31**

Kinetic assays were performed using Nanodrop™ One^{C}. Fresh stocks of peptide GCY **14** and PD **1-4, 6, 30** and **31** were prepared at respectively 25 mM in PB buffer (100 mM, pH 7.4, 5 mM EDTA) and 50 mM in MeCN, and were mixed together to a final concentration of 5 mM each in 7:3 PB buffer:MeCN solution (1 mL). An initial timepoint, t = 0 (in triplicate), was taken and the reaction was then left to incubated at 37 °C under constant agitation (300 rpm). Timepoints were measured in triplicate at 4-15 min time intervals depending on the PD tested. To obtain an optimal UV output, for each timepoint, samples were first diluted (1:10) before measurement in a 7:3 PB/MeCN solution. After blanking with 7:3 PB/MeCN solution, the absorbance was taken at 280 and 330 nm, and concentrations of PD ($[PD]_0$ and $[PD]_t$) and peptide ($[pep]_0$ and $[pep]_t$) were obtained using the absorbance measured at t = 0 and subsequently. PD concentration was determined using the absorbance at 330 nm and the extinction coefficients reported in Table S3. The absorbance at 280 nm was first corrected by using the correction factors reported in Table S3 and the corrected absorbance at 280 nm was used to assess concentration of the peptide at t = 0, using an extinction coefficient of $\varepsilon = 1490\ M^{-1}cm^{-1}$. Assuming a closed system, hence a 1:1 reaction between PD and peptide, $[pep]_t$ was calculated by subtracting the change in PD concentration from $[pep]_0$ ($[pep]_t = [pep]_0 - ([PD]_0 - [PD]_t)$). PD and peptide concentrations were then applied in the integrated rate law and graphs were plotted to determine k_{MA}, as shown below.

$$\frac{\ln \frac{[pep]_o\,[PD]_t}{[pep]_t[PD]_0}}{[PD]_0 - [pep]_0} = k_{MA}t, \text{where } k_{MA} = slope$$

Equation S1. Integrated rate law of second order Michael Addition reaction, used to determine k_{MA}.

*Table S4. Summary table of k_{MA} values found for the library of PD synthesised **1-4, 6, 30** and **31**.*

R, R'	k_{MA} ($M^{-1}\cdot s^{-1}$)	Standard deviation
Et, Et **(1)**	1.59E-02	3.62E-03
Et, $PhNH_2$ **(2)**	3.00E-02	7.58E-03
Et, Ph **(3)**	1.18E-01	1.11E-02
Et, PhF **(4)**	1.40E-01	2.63E-02
Et, PhF_5 **(6)**	6.32E-01	3.68E-03
Me, BCN clicked **(30)**	2.64E-02	9.47E-04
Ph, BCN clicked **(31)**	1.70E-01	1.13E-02

Summary

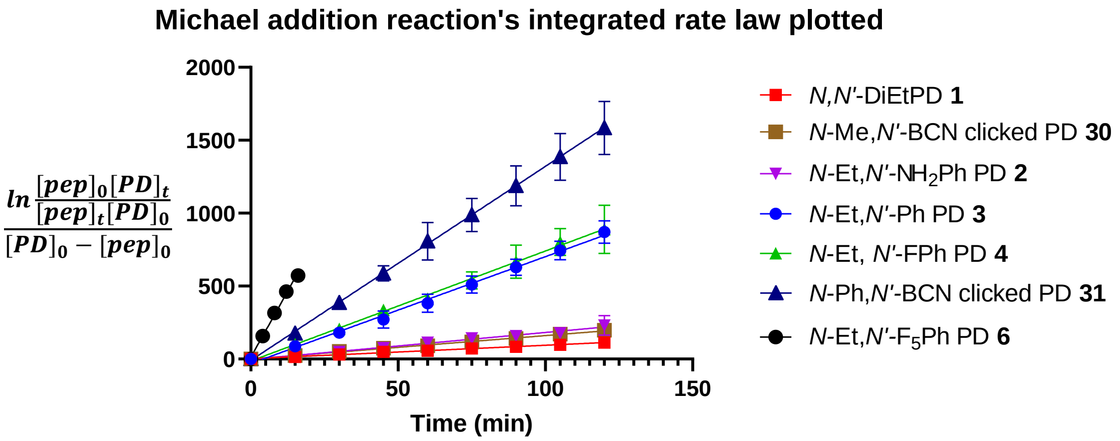

*Graph S2. Summary of Michael-Addition rate constant for the library of PDs synthesised **1-4, 6, 30** and **31** with the standard deviation obtained over 3 repeats.*

3.4 *Equilibrium constant kinetic assay*

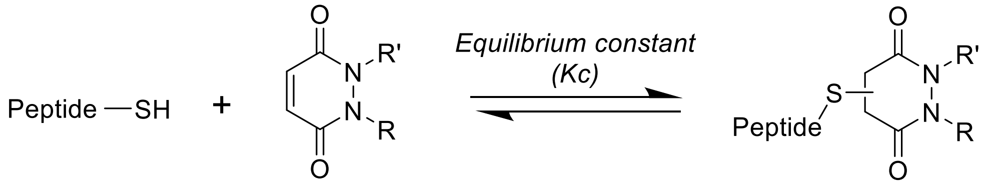

A_{280} | A_{330} | A_{280}

14 | **1-4**, **30** and **31**

Kinetic assays were performed using Nanodrop™ One[C]. For each replicate, fresh stocks of peptide GCY **14** and PD **1-4, 30** and **31** were made at a respective concentration of 25 mM in PB buffer (100 mM, pH 7.4, 5 mM EDTA) for the peptide and 50 mM in MeCN for the PD. PB buffer was used as blank. After the cuvette reached 37 °C, a solution (3 mL) of peptide **14** and PD **1-4, 30** and **31** for each PD was prepared at a desired final concentration of 0.5 mM, concentration necessary for an optimal absorbance output. The solution was then stirred in a cuvette for about 16 h at 37 °C. Initial UV-Vis measurements at 280 and 330 nm were taken after 1 min, then every 5 min during an average time of 16 h. Concentrations of PD ($[PD]_0$ and $[PD]_{eq}$), peptide ($[pep]_0$ and $[pep]_{eq}$) and peptide-PD conjugate at equilibrium ($[PD\text{-}pep]_{eq}$) were then obtained at each timepoint.

PD concentration was determined using the absorbance at 330 nm and the extinction coefficients reported in Table S3. It was corrected with a correction factor due to evaporation. PD concentration at equilibrium was assessed by plotting corrected PD concentration against time, extrapolated using a one phase decay equation (GraphPad Prism) and taking the value of the plateau.

The absorbance at 280 nm was first corrected by using the correction factor reported in Table S3 to remove PD absorption at 280 nm and the corrected absorbance at 280 nm was used to determine concentration of the peptide at t = 0, using an extinction coefficient of $\varepsilon = 1490\ M^{-1}cm^{-1}$.

Assuming a closed system, hence a 1:1 reaction between PD and peptide, $[pep\text{-}PD]_{eq}$ was calculated by subtracting $[PD]_0$ to the concentration of PD at equilibrium ($[PD\text{-}pep]_{eq} = [PD]_0 - [PD]_{eq}$). The concentration of peptide at equilibrium was obtained by removing $[pep\text{-}PD]_{eq}$ to the concentration of peptide at t = 0 ($[pep]_{eq} = [pep]_0 - [pep\text{-}PD]_{eq}$). Having in-hands each compound concentrations at equilibrium, Kc could be determined using the equilibrium equation (Equation S2).

$$K_c = \frac{[PD - cys]_{eq}}{[PD]_{eq}[cys]_{eq}} = \frac{([PD]_0 - [PD]_{eq})}{[PD]_{eq}([pep]_0 - ([PD]_0 - [PD]_{eq}))}$$

Equation S2. Equilibrium constant equation depending on $[PD]_0$, $[PD]_{eq}$ and $[pep]_0$

Summary

*Table S5. Summary table of Kc found for the library of PD synthesised **1-4, 30** and **31**.*

**R, R'**	**Kc average (M^{-1})**	**Standard deviation**
Et, Et **(1)**	10483	165
Et, $PhNH_2$ **(2)**	12698	2282
Et, Ph **(3)**	9514	1261
Et, PhF **(4)**	10800	512
Me, BCN clicked **(30)**	9893	870
Ph, BCN clicked **(31)**	13014	1638

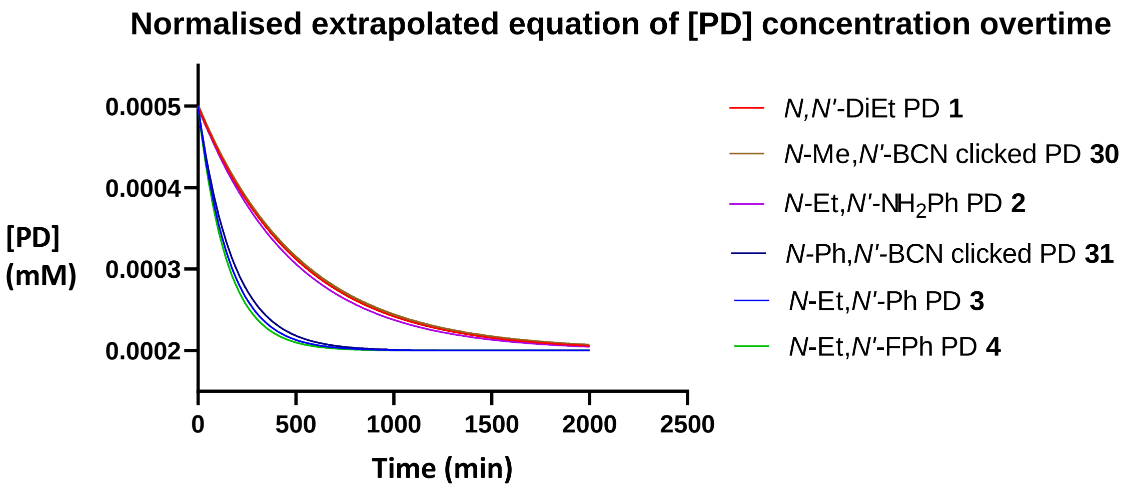

*Graph S3. Normalised and extrapolated equation found for [PD] concentration overtime for each PD **1-4, 30** and **31** synthesised.*

3.5 Retro Michael kinetic assay

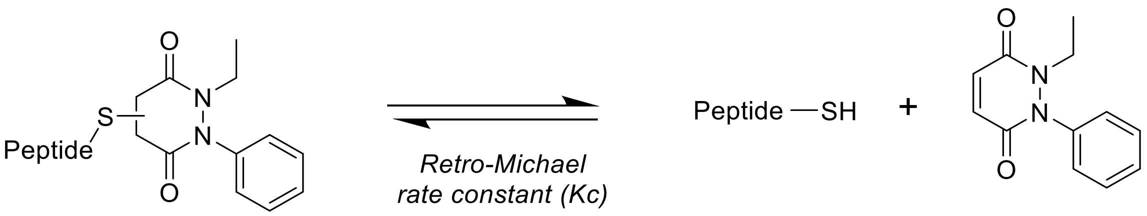

A_{280} A_{280} A_{330}

S14/S14' **14** **3**

Kinetic assays were performed using Nanodrop™ One^{C}. For each replicate, fresh stock of peptide GCY-PD conjugate **S14/S14'** was prepared at a concentration of 1.5 mM in PB buffer (100 mM, pH 7.4, 5 mM EDTA). The reaction was left to incubate at 37 °C under constant agitation (300 rpm). Timepoints were measured in triplicate every 15-20 min over 2 h. To obtain an optimal UV output, samples were first diluted (1:4) before measurement in PB buffer. After blanking with PB buffer, the absorbance was monitored at 280 and 330 nm, and concentrations of GCY-PD **14** ($[PD\text{-}pep]_0$) and PD ($[PD]_t$) were assessed at each timepoint. GCY-PD concentration at t = 0 was determined using the absorbance at 280 nm and corrected with the absorbance at 330 nm and the correction factor reported in Table S3. Assuming a closed system, the concentration of PD released over time was calculated using the absorbance at 330 nm subtracted with the absorbance at 330 nm at t = 0. $[PD\text{-}pep]_t$ was determined by subtracting $[PD]_t$ to $[PD\text{-}pep]_0$. Having in-hands [PD-pep] over time, k_{RM} was obtained by plotting the integrated rate law of the reaction.

$$\ln\left(\frac{[PD - pep]_t}{[PD - pep]_0}\right) = -k_{RM}t, \text{where } k_{RM} = slope$$

Equation S3. Integrated rate law of pseudo first order Retro-Michael reaction, used to determine k_{RM}.

Summary

*Table S6. Summary table of k_{RM} found for EtPhPD-GCY conjugate **S14**, with standard deviation, compared to k_{RM} calculated.*

R, R'	k_{RM} experimental average (s^{-1})	Standard deviation	Repeats	k_{RM} calculated (s^{-1})
Et, Ph (**3**)	1.82E-05	1.43E-06	3	1.24E-05 ± 0.88 E-05

.

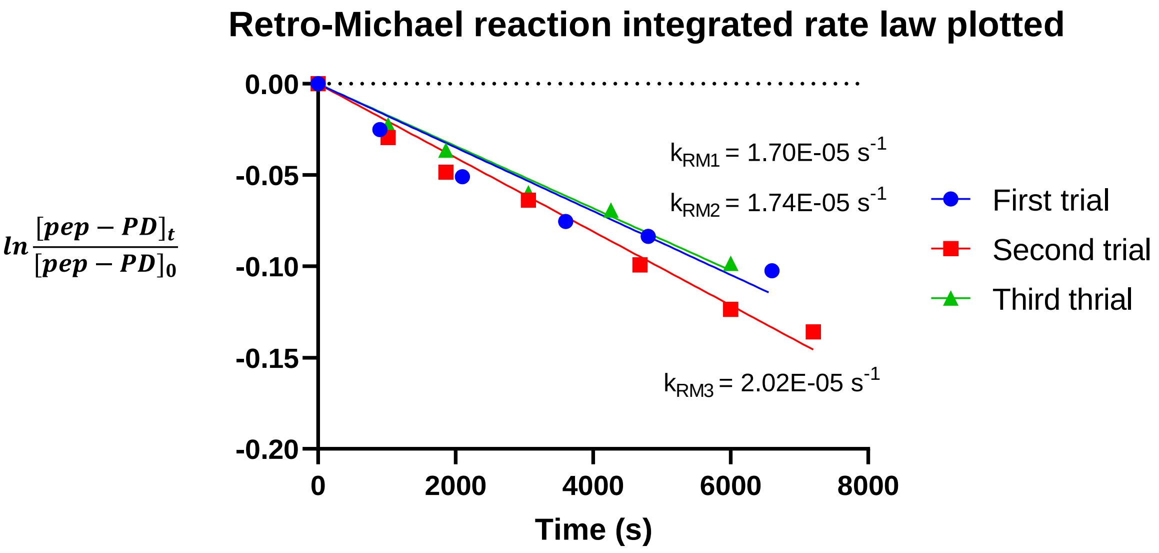

Graph S4. RM integrated rate law plotted with k_{RM} found for each repeat for GCY-PD ***S14/S14'****..*

3.6 *Stability study of N-Et,N'-F$_5$Ph PD 6 under kinetic assays conditions*

3.6.1 N-Et,N'-F$_5$Ph PD 6 stability under k_{MA} kinetic assay conditions

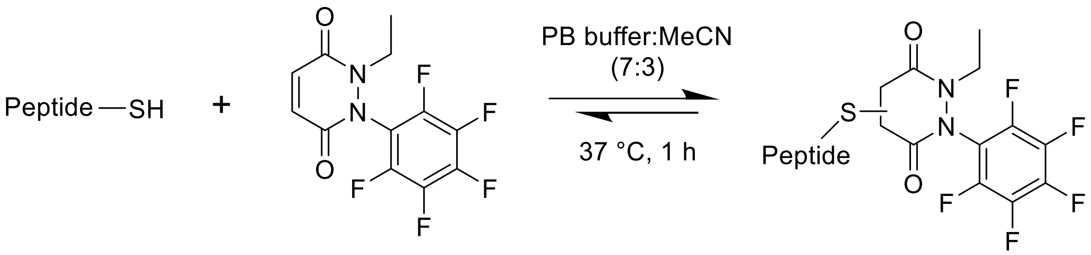

14 (5 mM) **6** (5 mM) **15**

Fresh stocks of peptide GCY **14** and PD **6** were made at a respective concentration of 25 mM in PB buffer (100 mM, pH 7.4, 5 mM EDTA) for the peptide and 50 mM in MeCN for the PD. To a solution of PB buffer (500 µL) and MeCN (200 µL) were added 200 µL of peptide and 100 µL of PD for a final concentration of 5 mM of both. The reaction mixture was mixed and incubated for 1 h at 37 °C. LCMS analysis was quickly (i.e., in the 10 minutes following LC-MS sample preparation) carried out after 1 h.

Expected masses: 341 Da (GCY); 307 Da (PD); 648 Da (GCY + PD)
Observed masses: 648 Da; 307 Da; 348 Da

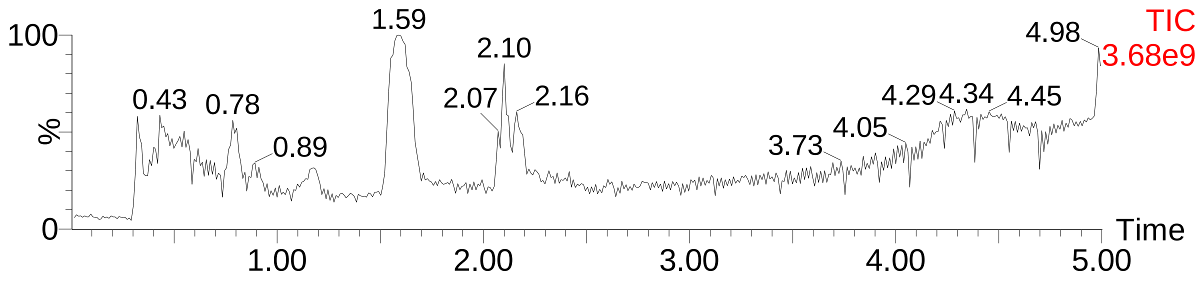

1.52-1.75 min:

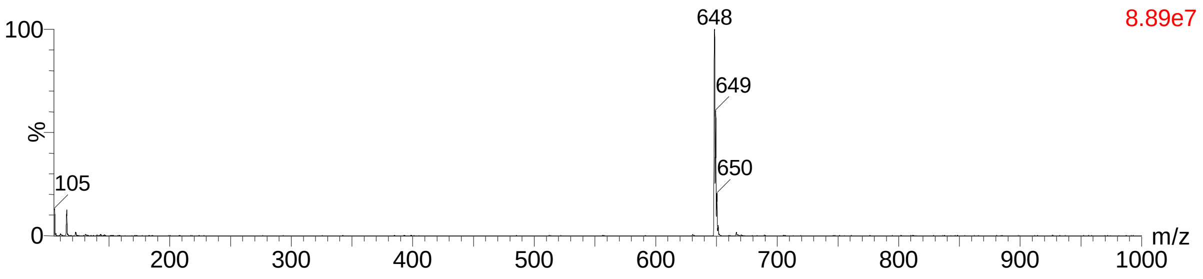

2.07-2.25 min:

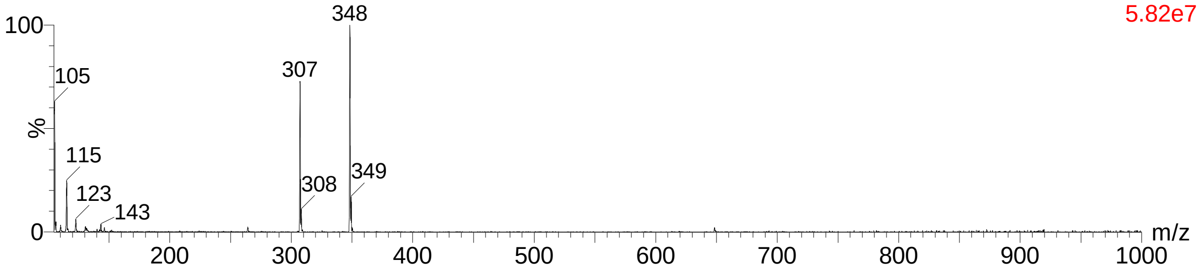

*Figure S1. LCMS analysis of GCY peptide **14** + PD **6** after 1 h in PB buffer and acetonitrile at 37 °C, under k_{MA} kinetic assay conditions.*

3.6.2 N-Et,N'-F_5Ph PD 6 stability under Kc kinetic assay conditions

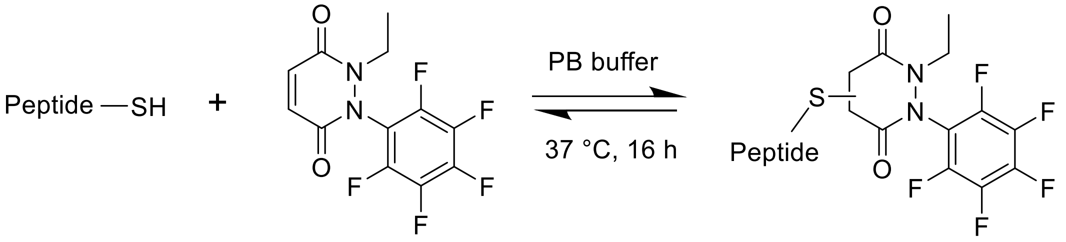

Fresh stocks of peptide GCY **14** and PD **6** were made at a respective concentration of 25 mM in PB buffer (100 mM, pH 7.4, 5 mM EDTA) for the peptide and 50 mM in MeCN for the PD. To 1.358 mL of PB buffer were added 28 µL of peptide and 14 µL of PD for a final concentration of 0.5 mM of both. The reaction mixture was mixed and incubated for 16 h at 37 °C. LCMS analysis was quickly (i.e., in the 10 minutes following LC-MS sample preparation) carried out after 16 h.

Expected masses: 341 Da (GCY); 307 Da (PD); 648 Da (GCY + PD); 666 Da (GCY + PD hydrolysed); 325 Da (hydrolysed PD)
Observed masses: 648 Da; 666 Da; 307 Da; 325 Da; 348 Da

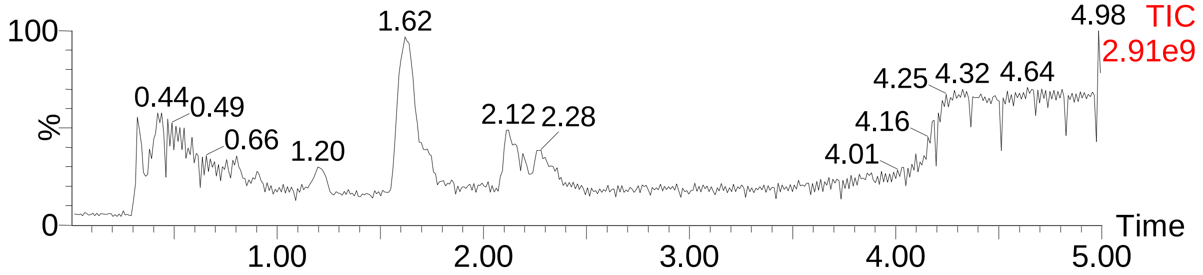

1.57-1.83 min:

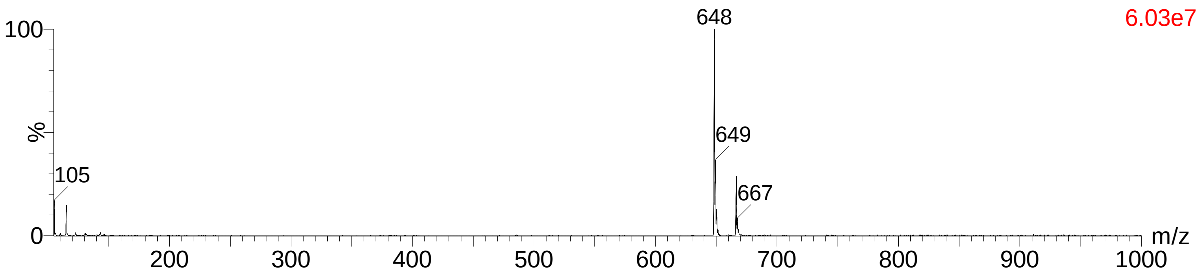

2.08-2.45 min:

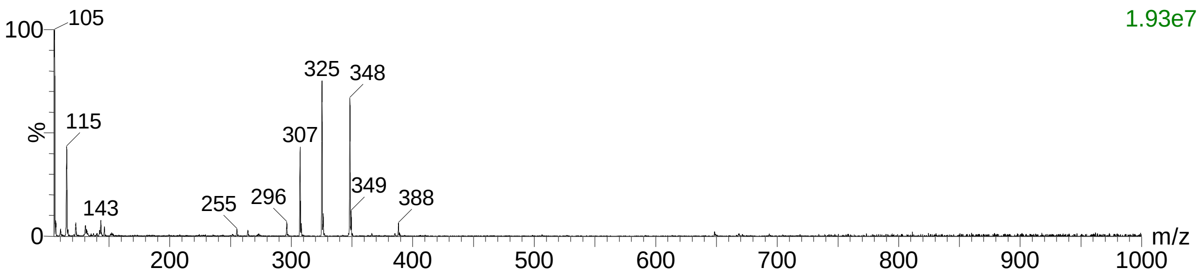

*Figure S2. LCMS analysis of GCY peptide **14** + PD **6** after 16 h in PB buffer at 37 °C, under Kc kinetic assay conditions.*

4. Protein/material experiments

4.1 GFPS147C experiments

4.1.1 GFPS147C reduction

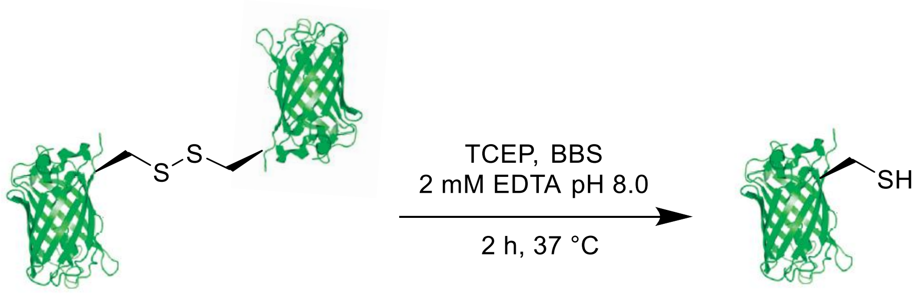

S10 **32**

To a solution of dimer GFPS147C **S10** obtained as previously described[13] (300 µL, 100 µM in BBS buffer (25 mM sodium borate, 25 mM NaCl, 2 mM EDTA, pH 8.0), 0.030 µmol) was added TCEP·HCl (18 µL, 50 mM in BBS buffer (pH 8), 50 equiv., 1.5 µmol). The mixture was incubated for 120 min at 37 °C under constant agitation (300 rpm). Excess TCEP was removed by buffer-exchange in BBS EDTA buffer using 2 × Zeba™ Spin Desalting columns to yield GFPS147C monomer **32** (0.024 µmol, 80%). Expected masses: 29,342 Da. Observed masses: 29,343 Da; 29,525 Da (GFP artifact).

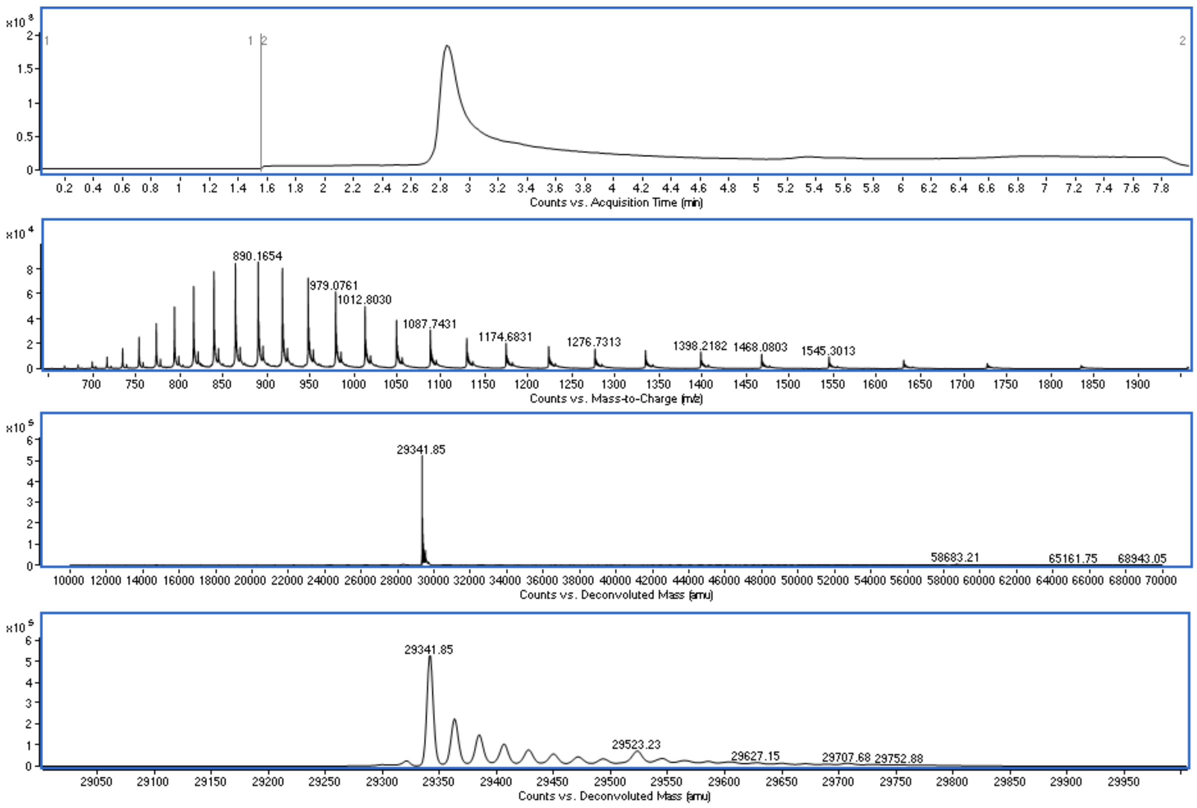

4.1.2 GFPS147C conjugation to PDs (general procedure)

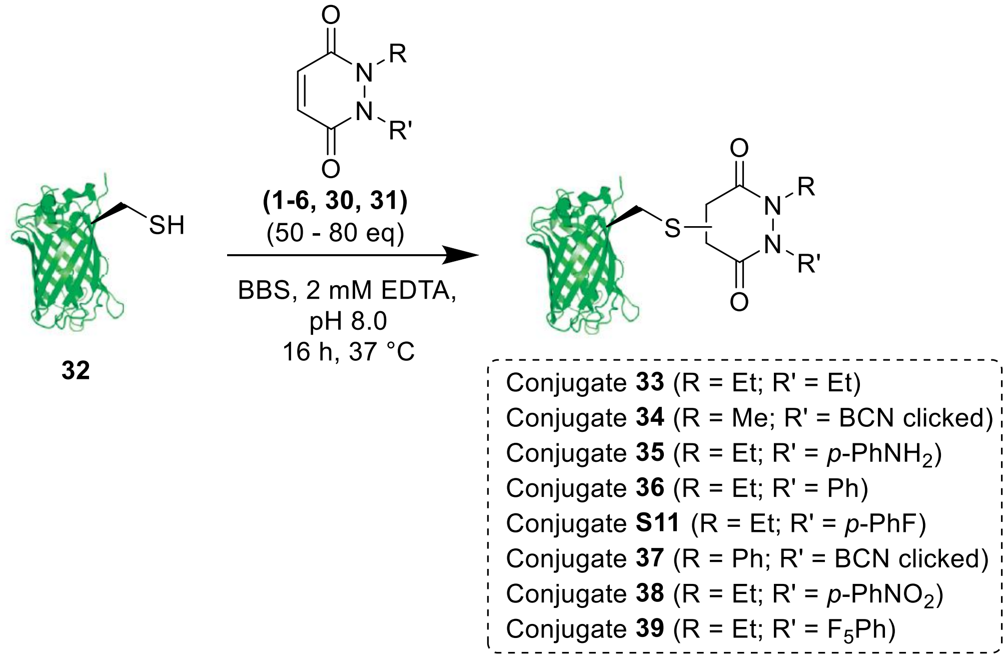

Procedure: To a solution of reduced GFPS147C **32** (80 µL, 60 µM in BBS EDTA, 0.0048 µmol) was added a PD derivative (4.8 µL, 50 mM in MeCN, 50 equiv., 0.24 µmol for **3-6** and **31** or 7.7 µL, 50 mM in MeCN, 80 equiv., 0.38 µmol for **1, 2** and **30**) and the reaction was incubated at 37 °C for 16 h.

Analysis: After this time, the volume was adjusted to 100 µL and excess of small molecules was removed using Zeba^{TM} Spin Desalting column in LCMS grade water. LCMS analysis was carried out quickly after removal of the small molecules (within 10 min) to avoid deconjugation of the PD from the GFPS147C-PD conjugate during analysis.

Conjugates **34**-**38** were analysed using Agilent 6510. Conjugates **33**, **S11** and **39** were run on Agilent 6530.

Conjugate ***33****, t = 0 h*

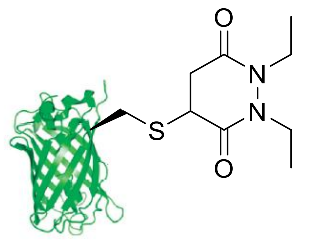

33

Expected mass: 29,511 Da. Observed mass: 29,510 Da.

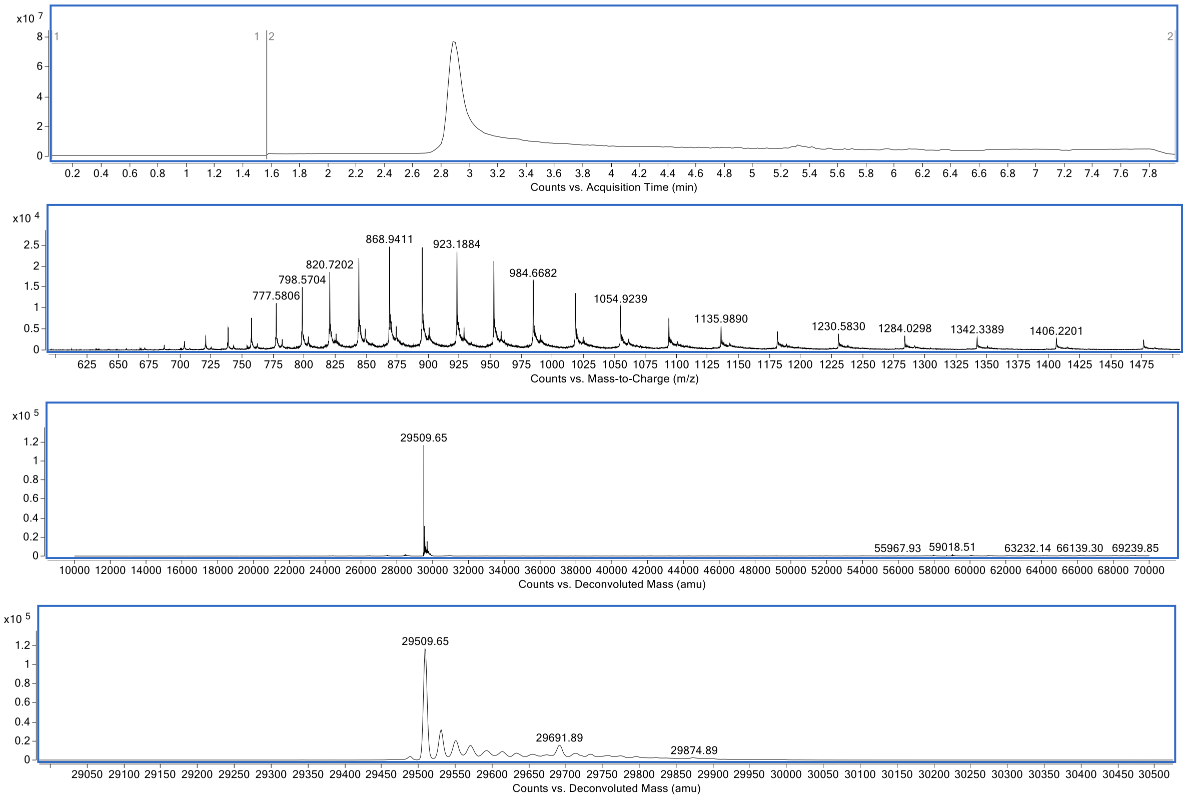

*Conjugate **34**, t = 0 h*

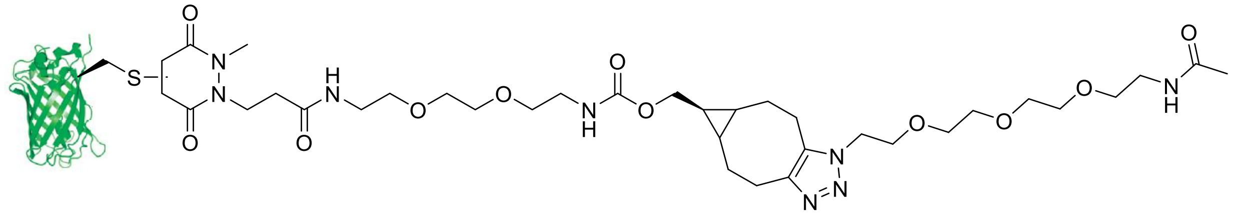

34

Expected mass: 30,108 Da. Observed mass: 30,107 Da.

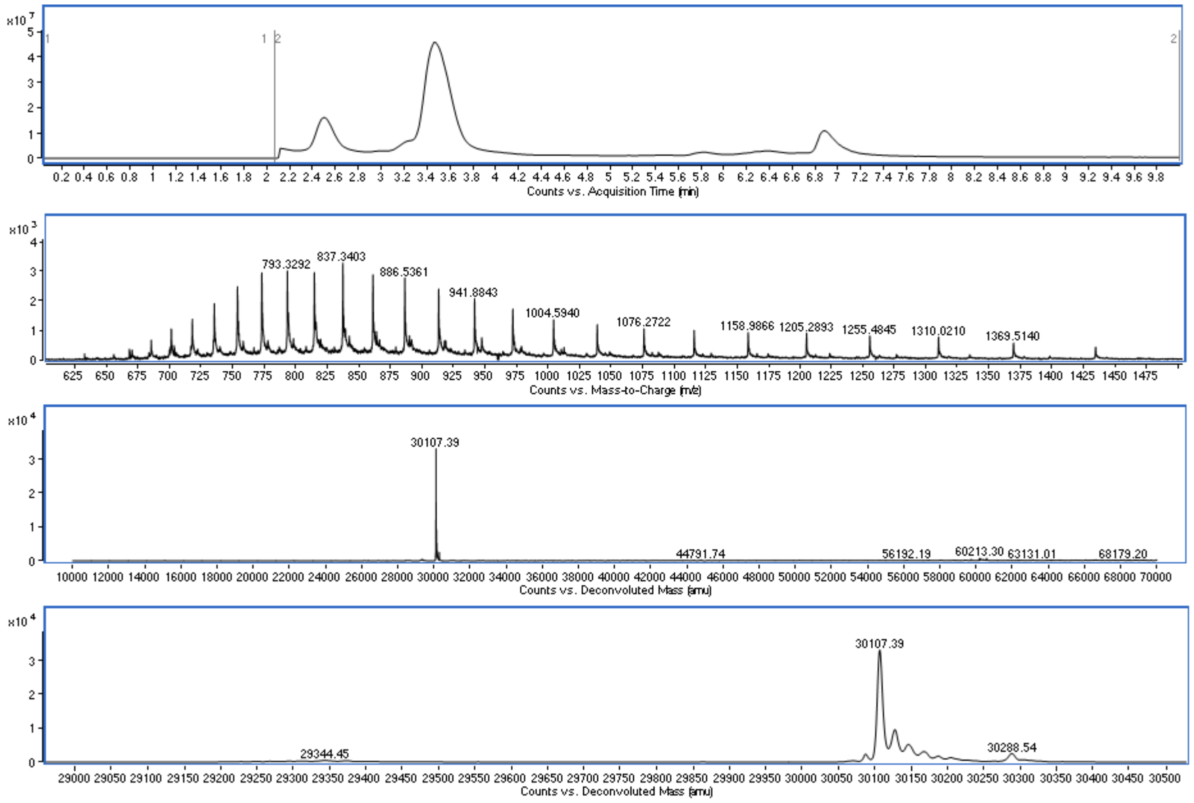

*Conjugate **35**, t = 0 h*

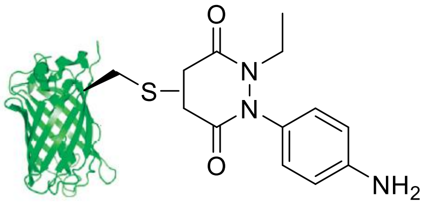

35

Expected mass: 29,574 Da. Observed mass: 29,574 Da.

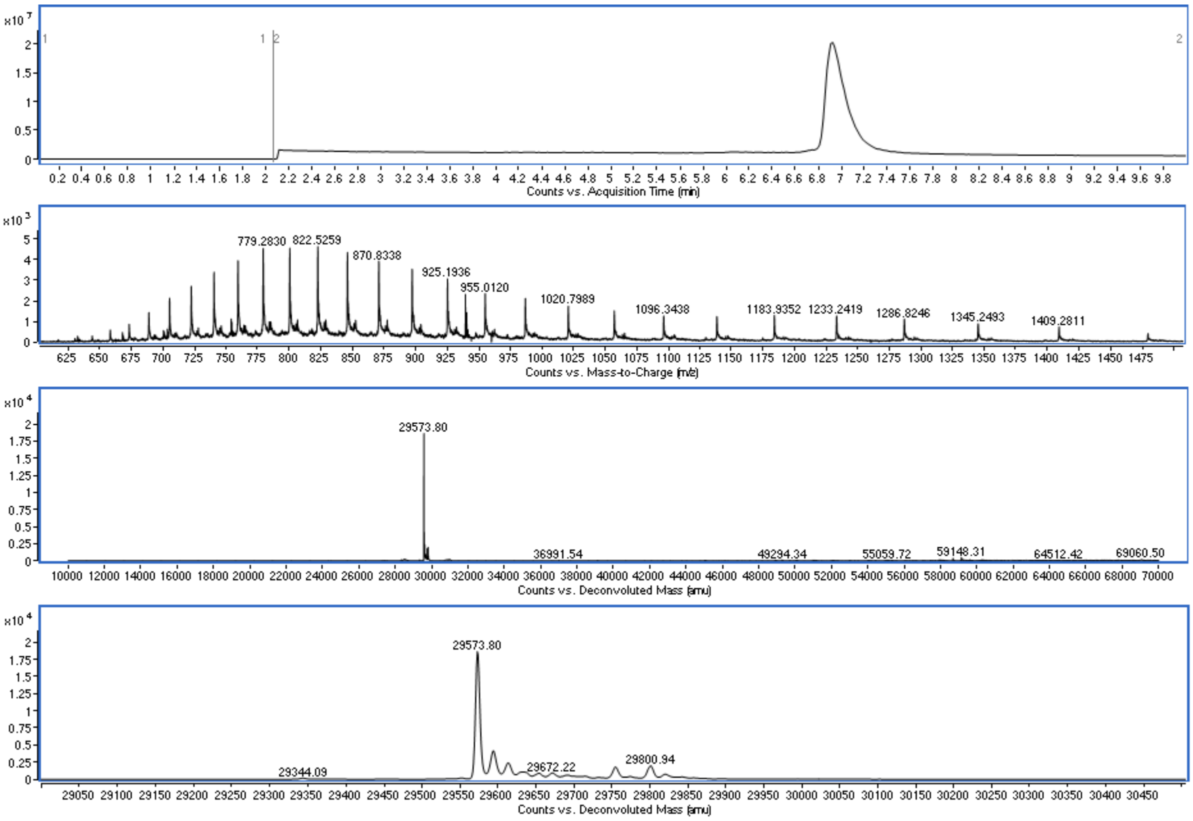

*Conjugate **36**, t = 0 h*

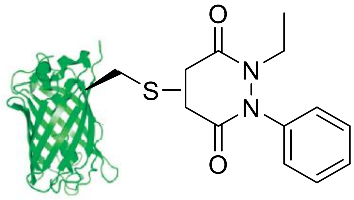

36

Expected mass: 29,559 Da. Observed mass: 29,559 Da.

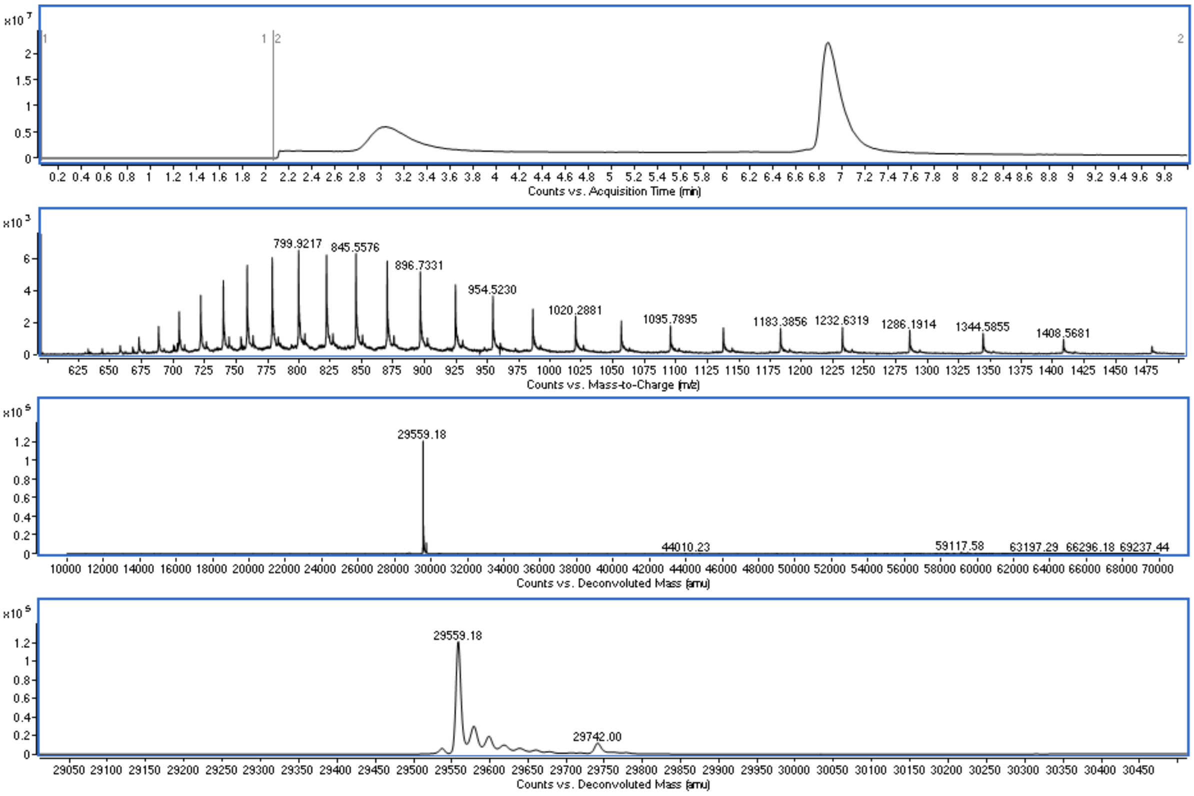

*Conjugate **S11**, t = 0 h*

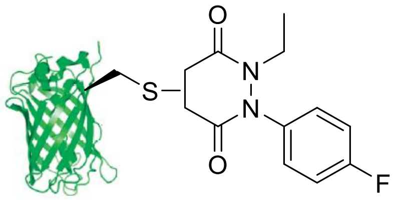

S11

Expected mass: 29,577 Da. Observed mass: 29,576 Da.

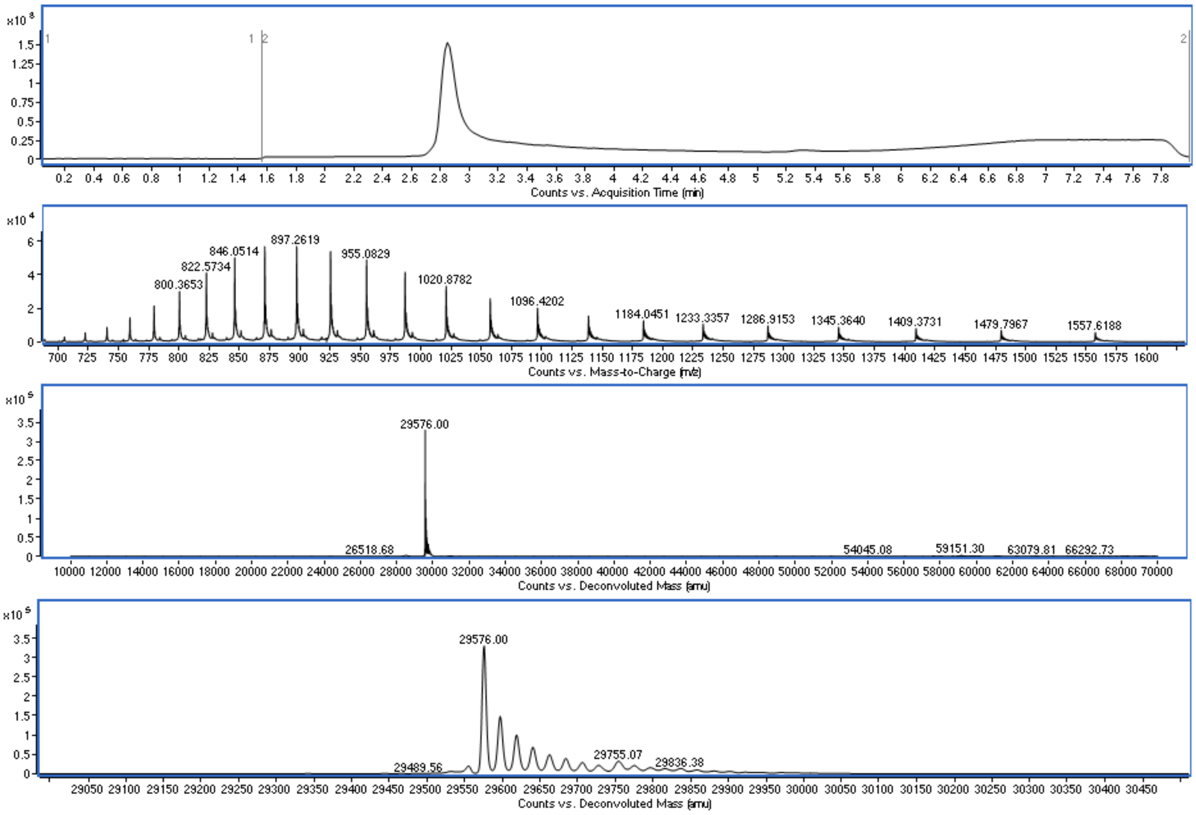

*Conjugate **37**, t = 0 h*

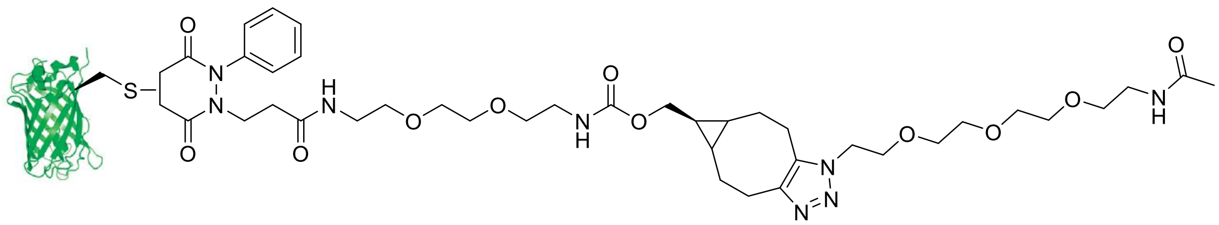

37

Expected mass: 30,170 Da. Observed mass: 30,170 Da.

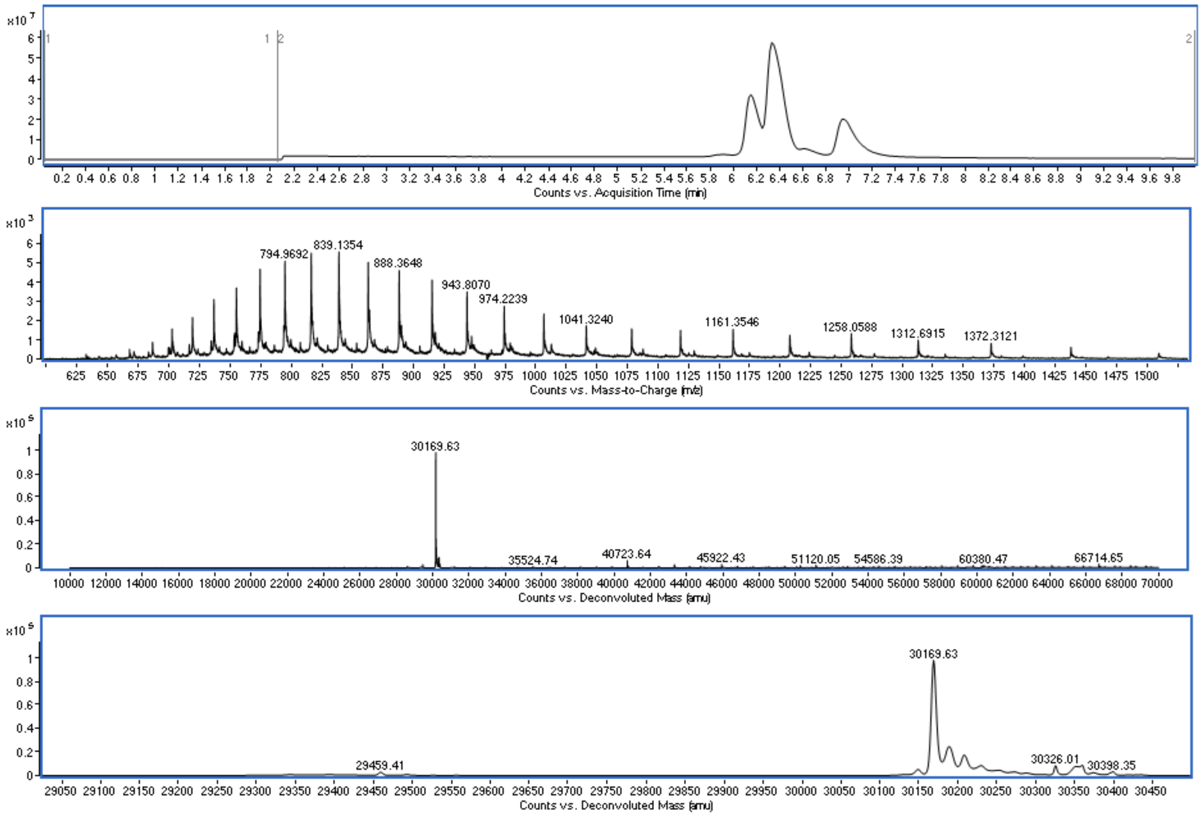

*Conjugate **38**, t = 0 h*

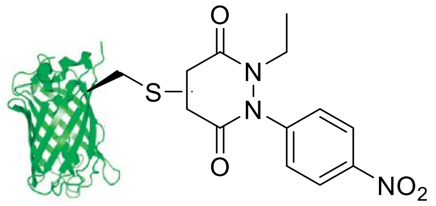

38

Expected mass: 29,604 Da. Observed mass: 29,604 Da.

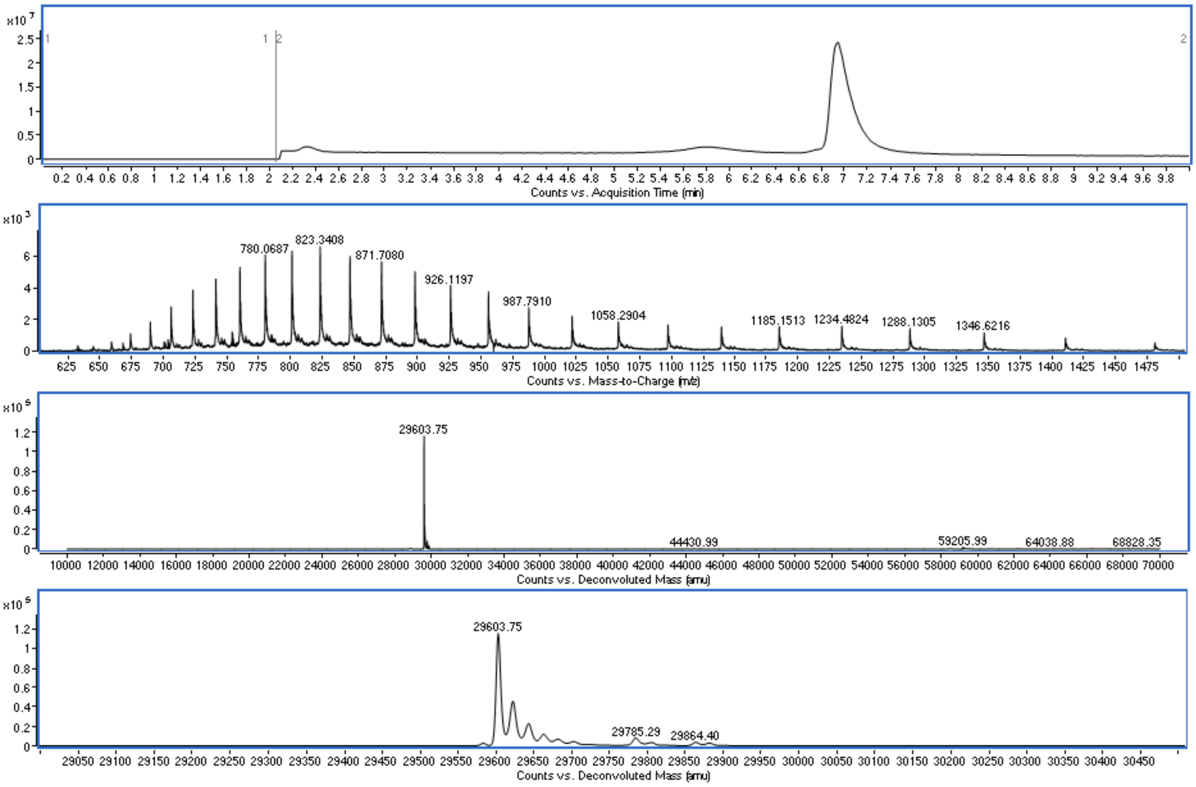

*Conjugate **39**, t = 0 h*

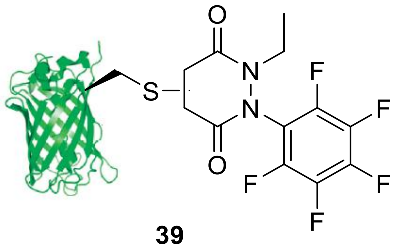

39

Expected mass: 29,649 Da. Observed mass: 29,648 Da.

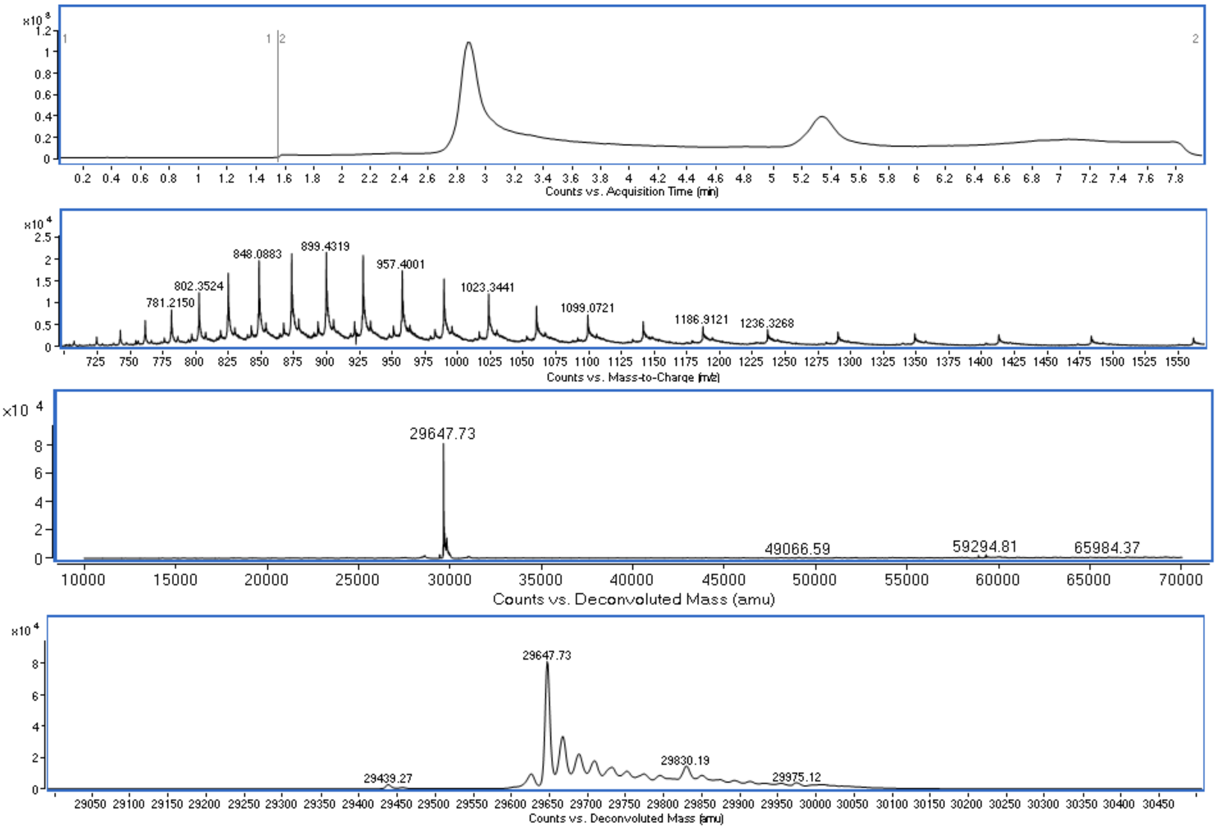

4.1.3 Release study of PD from PD-GFPS147C conjugates

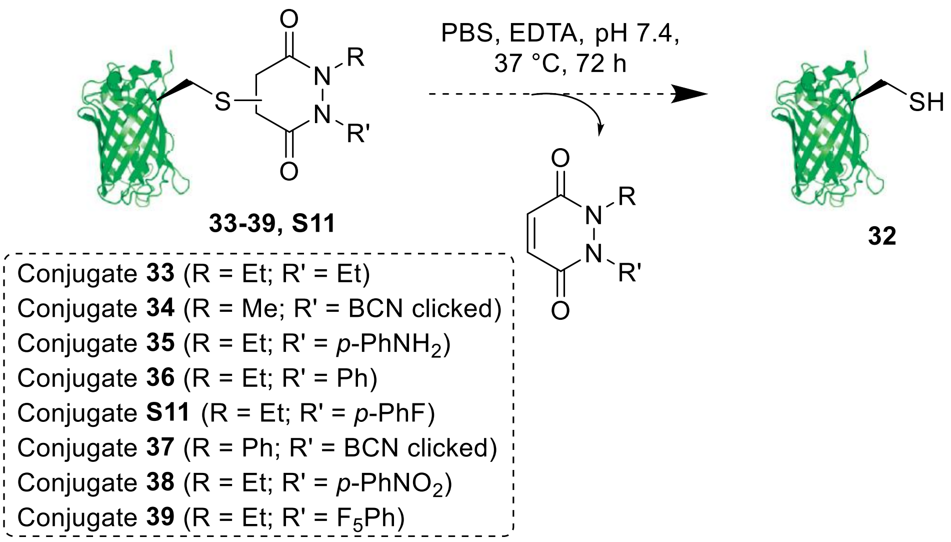

Following the formation of GFPS147C-PD conjugates **33-39** and **S11** described in the procedure in section 4.1.2, the complete removal of the excess of small molecule was carried out by using 2 × Zeba^{TM} Spin Desalting column in PBS pH 7.4, 2 mM EDTA. The concentration of the conjugates **33**-**39** and **S11** was adjusted to 35 µM and the solution left to incubate under constant agitation (300 rpm) at 37 °C. LC-MS analysis was taken after 2.5 h, 6.5 h, 24 h, 48 h and 72 h. LCMS analysis was carried out quickly after removal of the small molecules (within 10 min) to avoid deconjugation of the PD from the GFPS147C-PD conjugate during analysis.

- Timepoint 2.5 h

*Conjugate **33**, t = 2.5 h*

Expected masses: 29,342 Da (GFPS147C **32**); 29,511 Da (GFPS147C-PD **33**)

Observed mass: 29510 Da

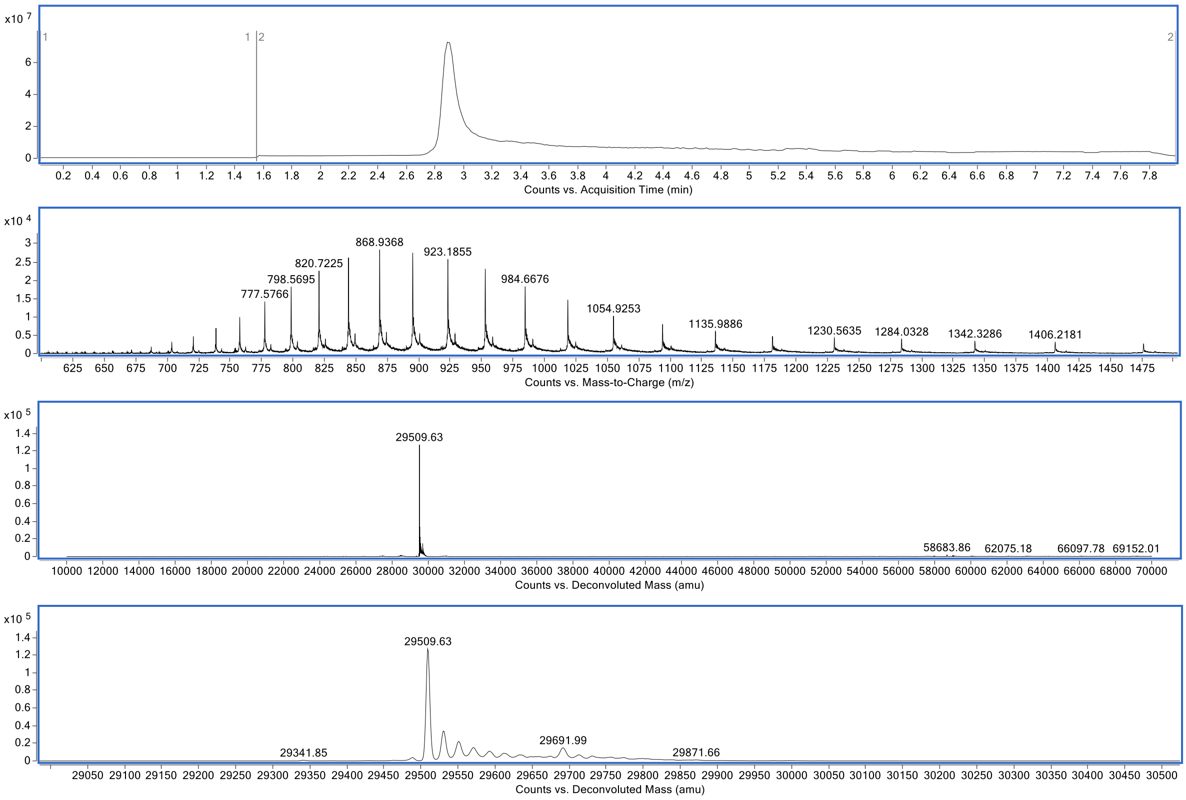

*Conjugate **34**, t = 2.5 h*

Expected masses: 29,342 Da (GFPS147C **32**); 30,108 Da (GFPS147C-PD **34**)
Observed masses: 29,343 Da; 30,108 Da

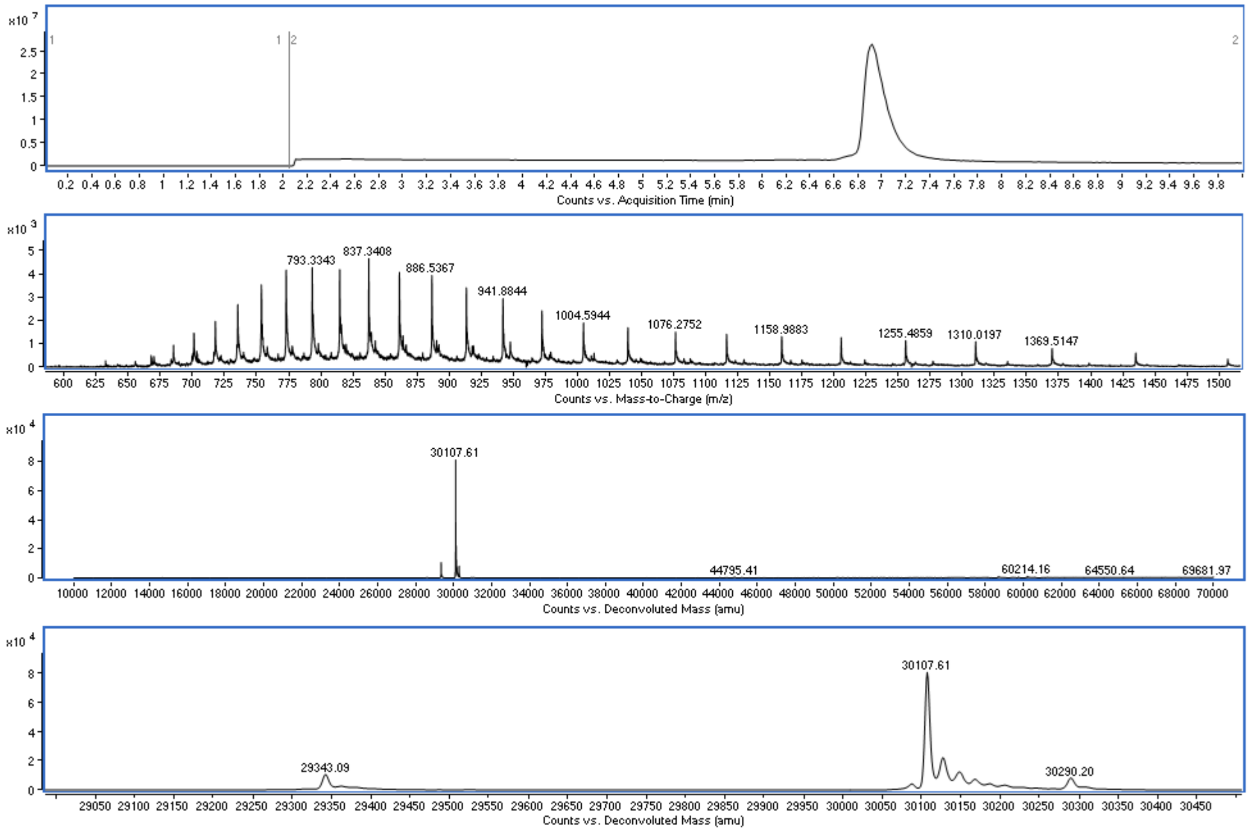

*Conjugate **35**, t = 2.5 h*

Expected masses: 29,342 Da (GFPS147C **32**); 29,574 Da (GFPS147C-PD **35**)
Observed masses: 29,342 Da; 29,574 Da

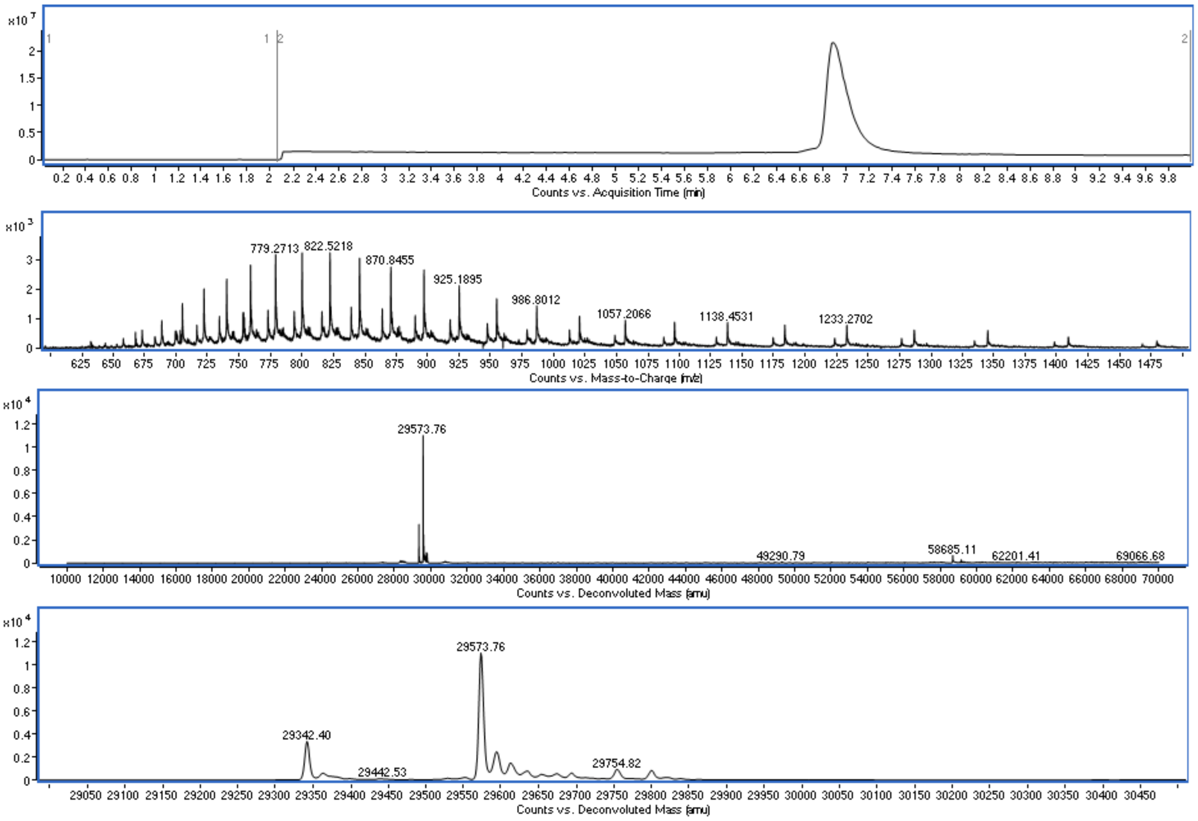

*Conjugate **36**, t = 2.5 h*

Expected masses: 29,342 Da (GFPS147C **32**); 29,559 Da (GFPS147C-PD **36**)

Observed masses: 29,343 Da; 29,559 Da

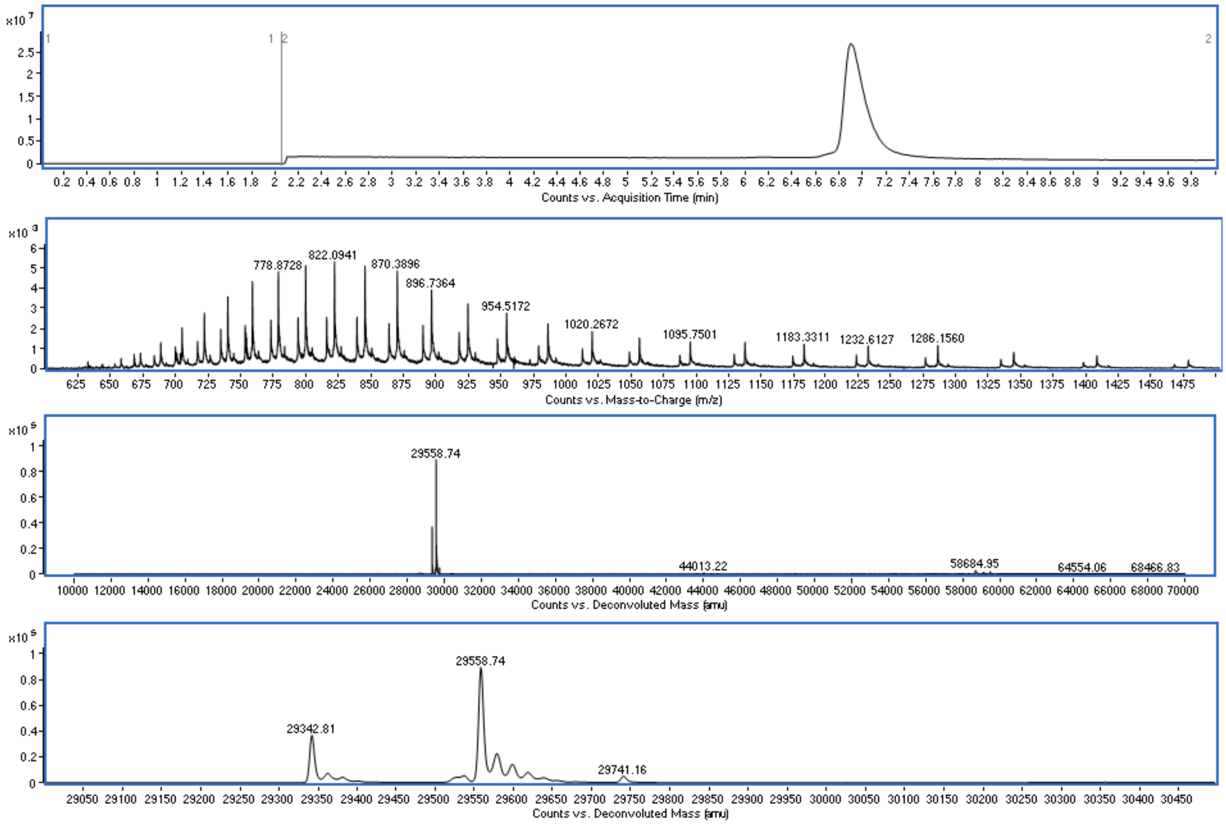

*Conjugate **S11**, t = 2.5 h*

Expected masses: 29,342 Da (GFPS147C **32**); 29,577 Da (GFPS147C-PD **S11**)
Observed masses: 29,342 Da; 29,576 Da

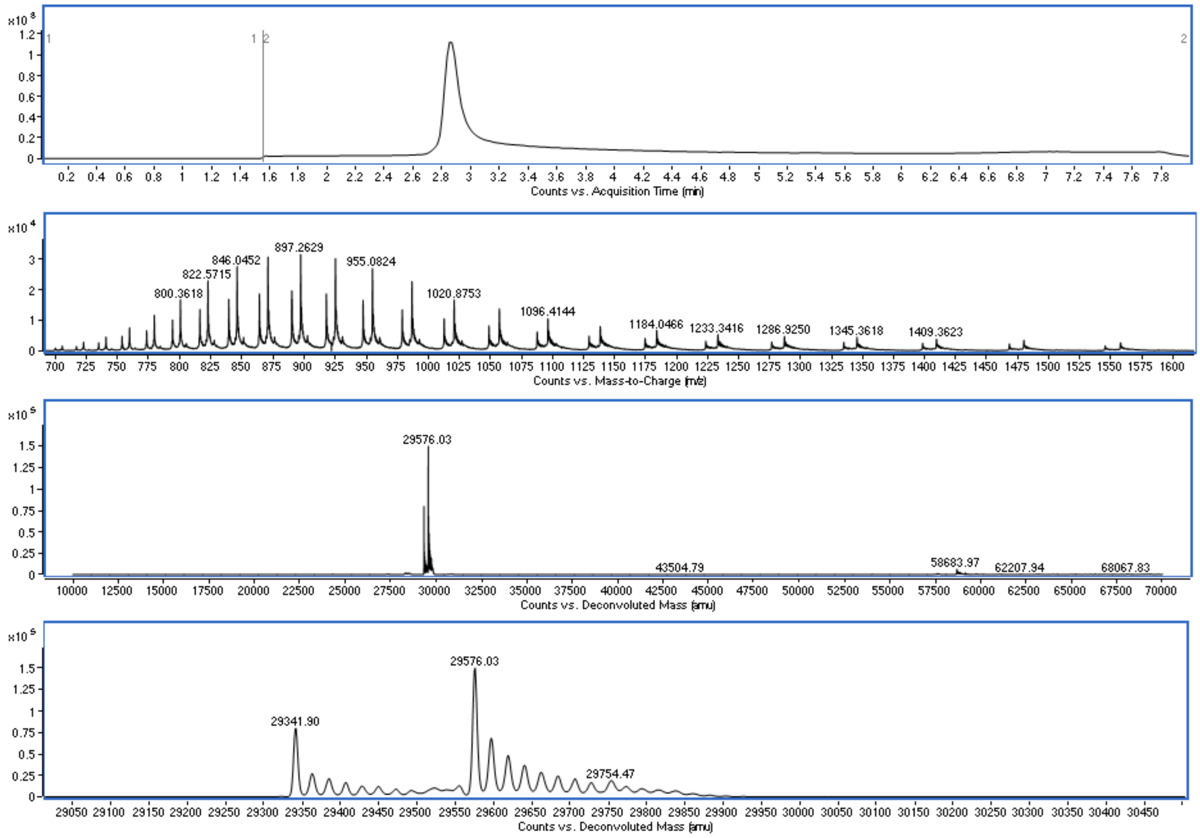

*Conjugate **37**, t = 2.5 h*

Expected masses: 29,342 Da (GFPS147C **32**); 30,170 Da (GFPS147C-PD **37**)
Observed masses: 29,343 Da; 30,169 Da

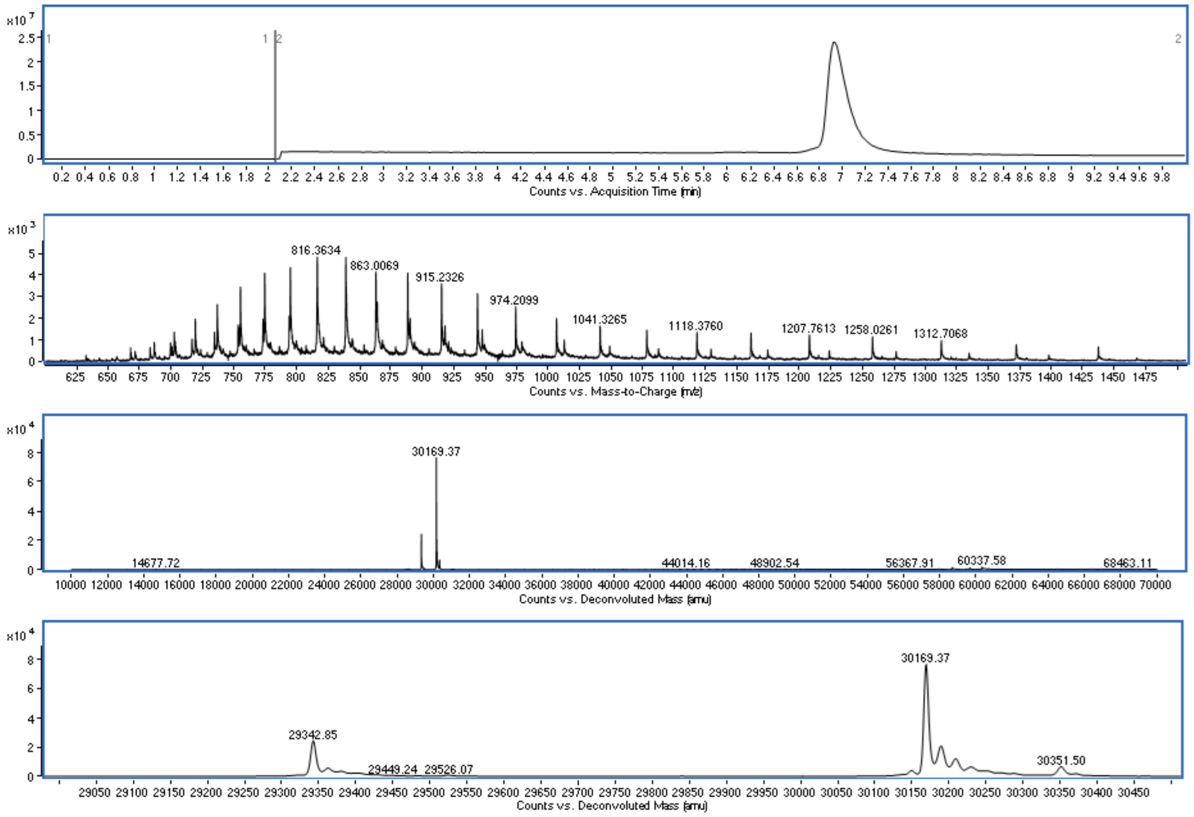

*Conjugate **38**, t = 2.5 h*

Expected masses: 29,342 Da (GFPS147C **32**); 29,604 Da (GFPS147C-PD **38**)
Observed masses: 29,342 Da; 29,603 Da; 29,621 Da

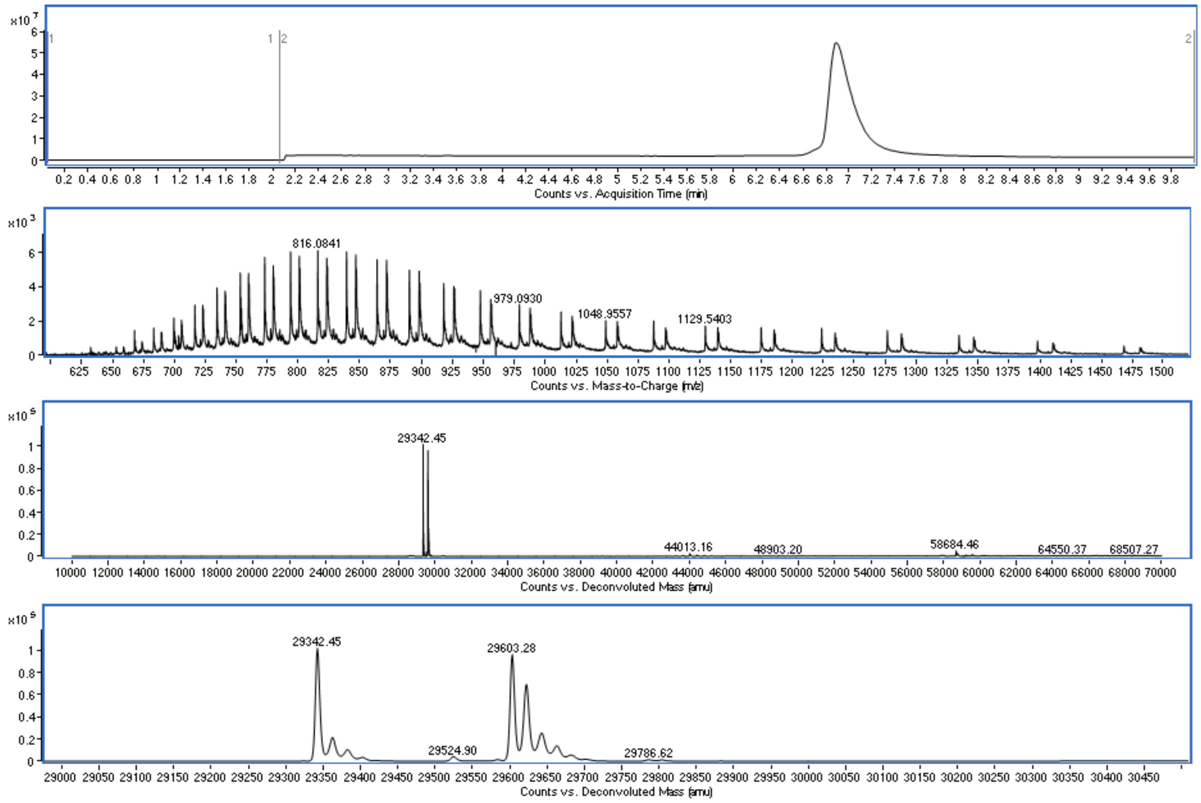

*Conjugate **39**, t = 2.5 h*

Expected masses: 29,342 Da (GFPS147C **32**); 29,649 Da (GFPS147C-PD **39**)

Observed masses: 29,342 Da; 29,438 Da; 29,648 Da

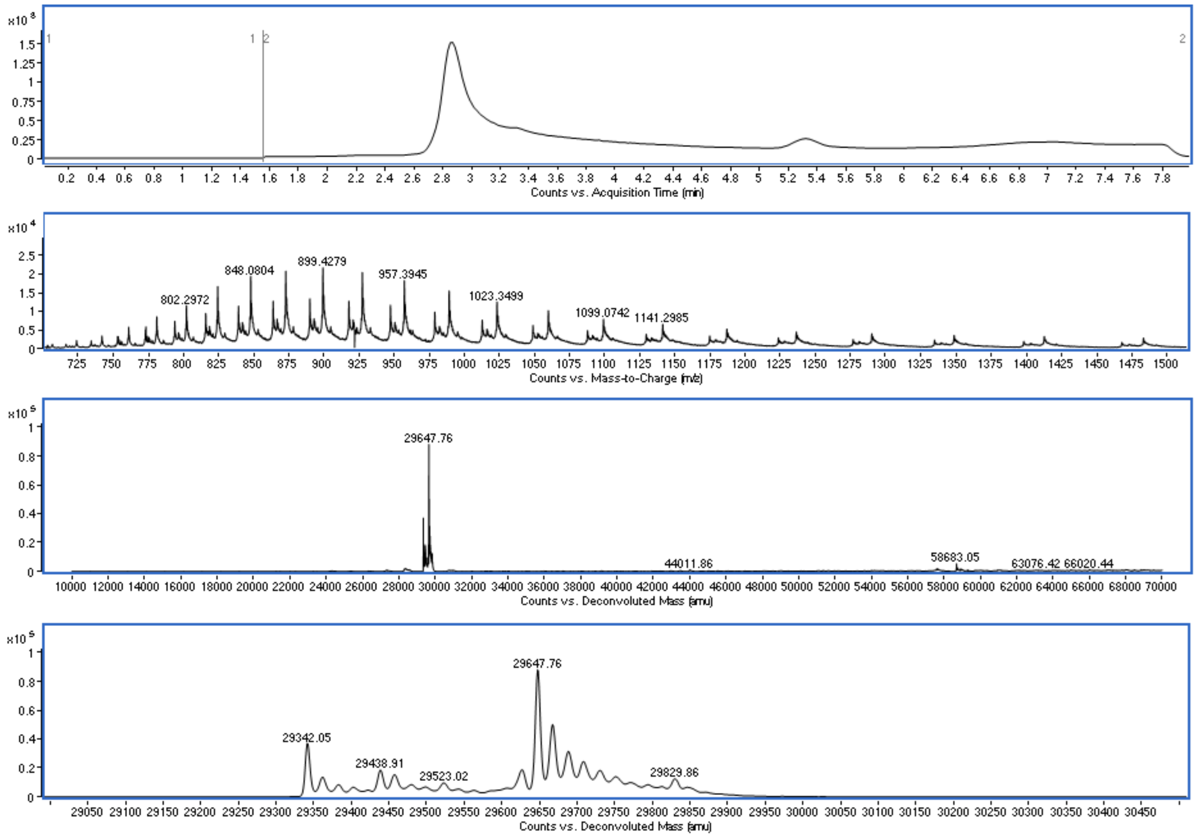

- Timepoint 6.5 h

*Conjugate **33**, t = 6.5 h*

Expected masses: 29,342 Da (GFPS147C **32**); 29,511 Da (GFPS147C-PD **33**)
Observed masses: 29,510 Da

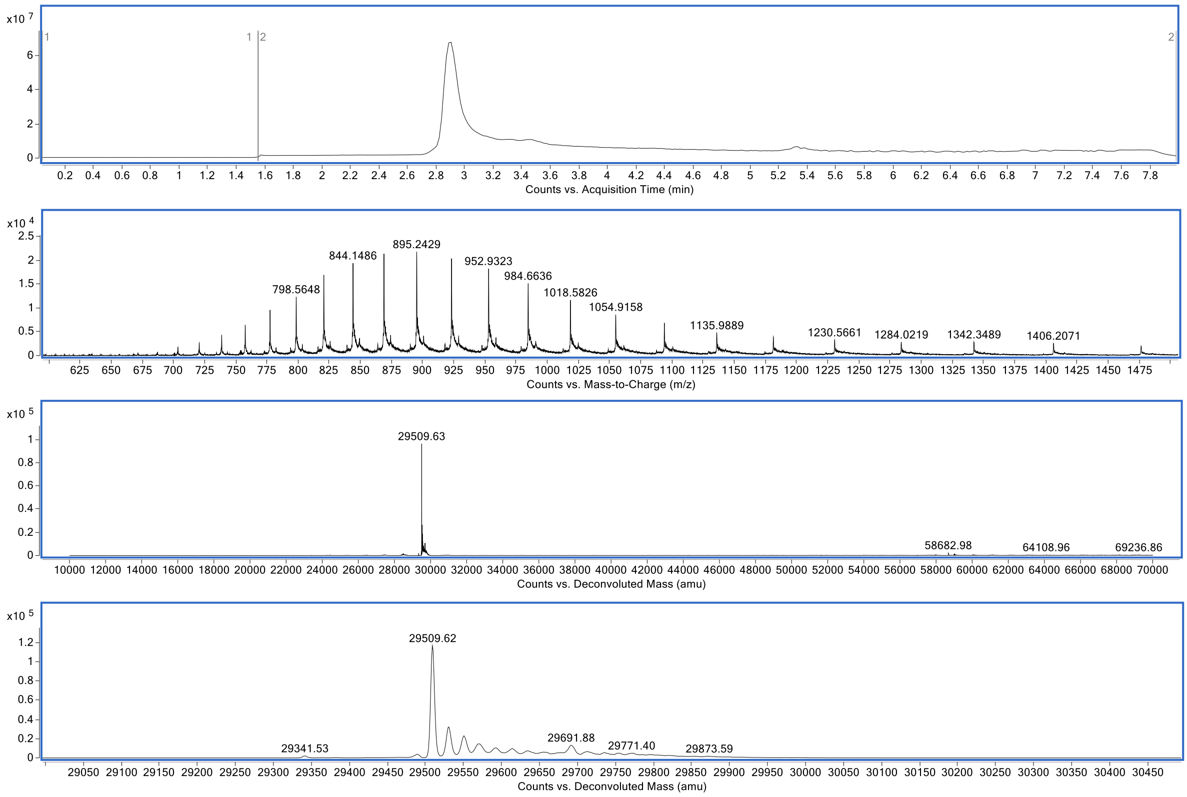

*Conjugate **34**, t = 6.5 h*

Expected masses: 29,342 Da (GFPS147C **32**); 30,108 Da (GFPS147C-PD **34**)

Observed masses: 29,342 Da; 30,107 Da

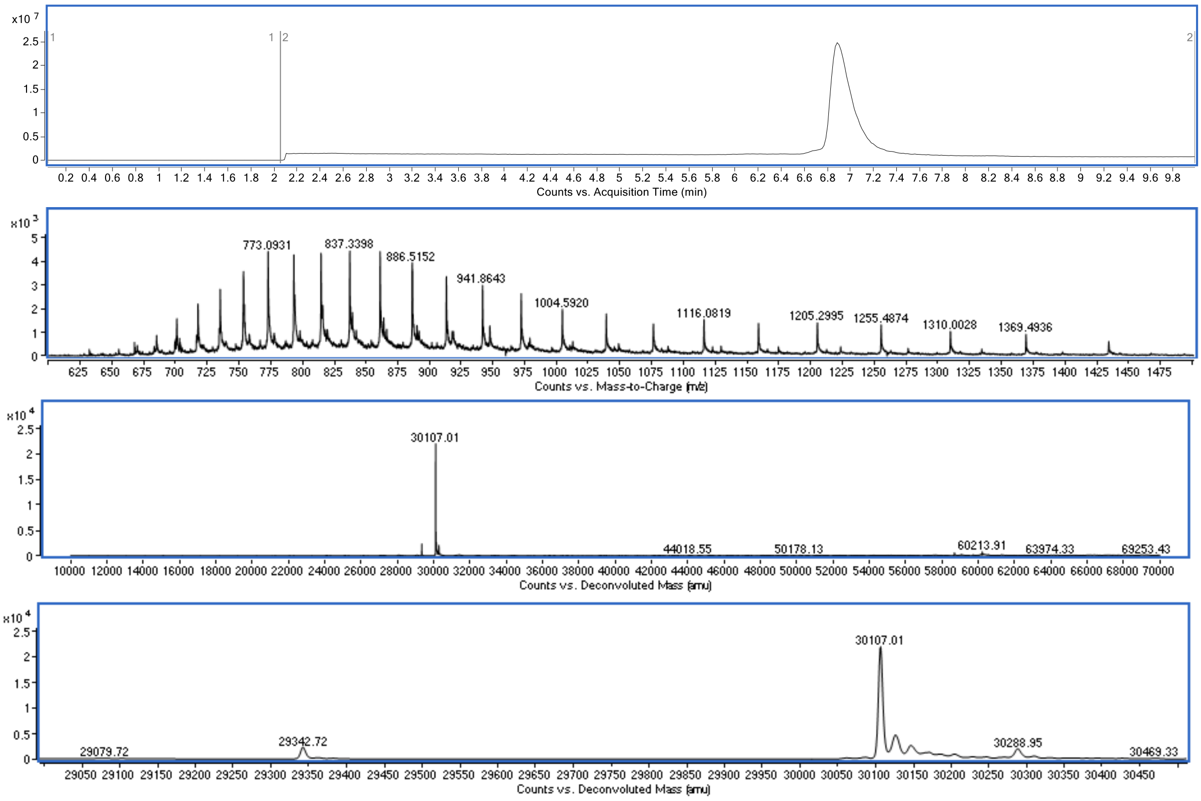

*Conjugate **35**, t = 6.5 h*

Expected masses: 29,342 Da (GFPS147C **32**); 29,574 Da (GFPS147C-PD **35**)
Observed masses: 29,342 Da; 29,574 Da

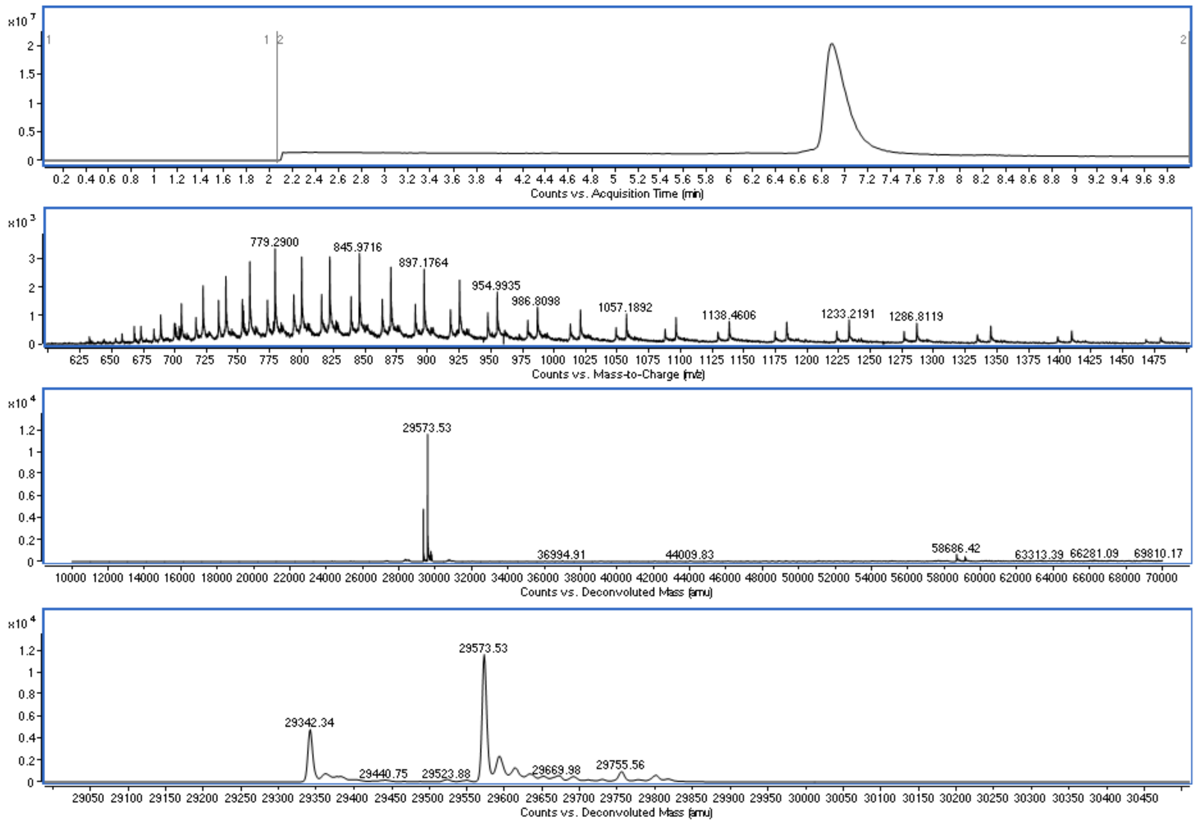

*Conjugate **36**, t = 6.5 h*

Expected masses: 29,342 Da (GFPS147C **32**); 29,559 Da (GFPS147C-PD **36**)
Observed masses: 29,343 Da; 29,559 Da

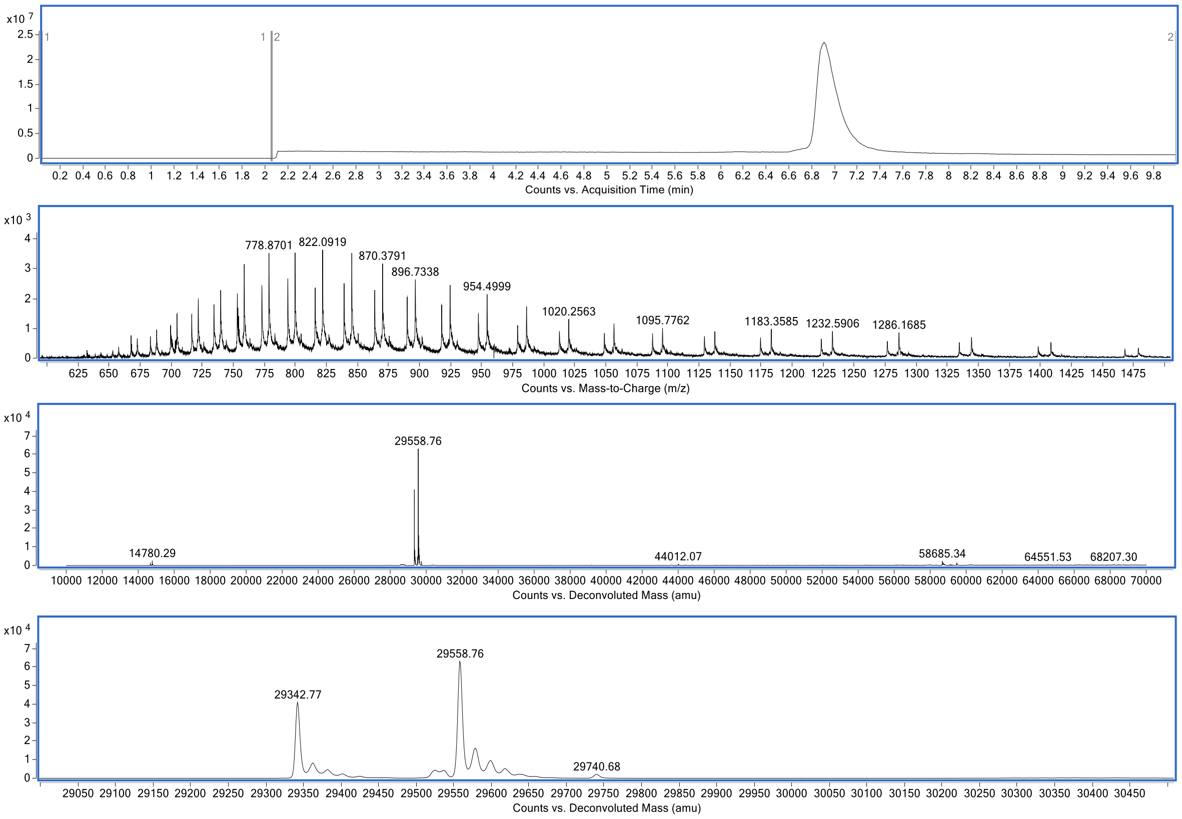

*Conjugate **S11**, t = 6.5 h*

Expected masses: 29,342 Da (GFPS147C **32**); 29,577 Da (GFPS147C-PD **S11**)
Observed masses: 29,342 Da; 29,576 Da

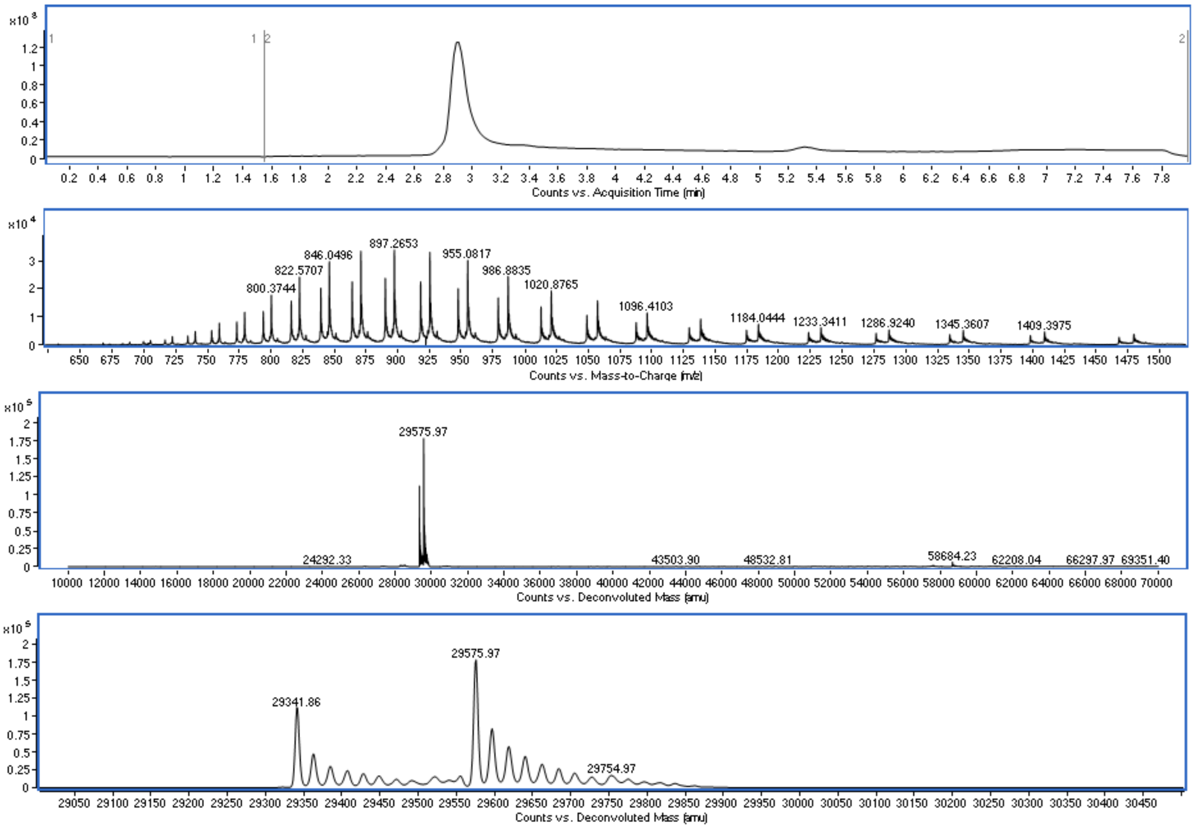

*Conjugate **37**, t = 6.5 h*

Expected masses: 29,342 Da (GFPS147C **32**); 30,170 Da (GFPS147C-PD **37**)
Observed masses: 29,343 Da; 30,169 Da

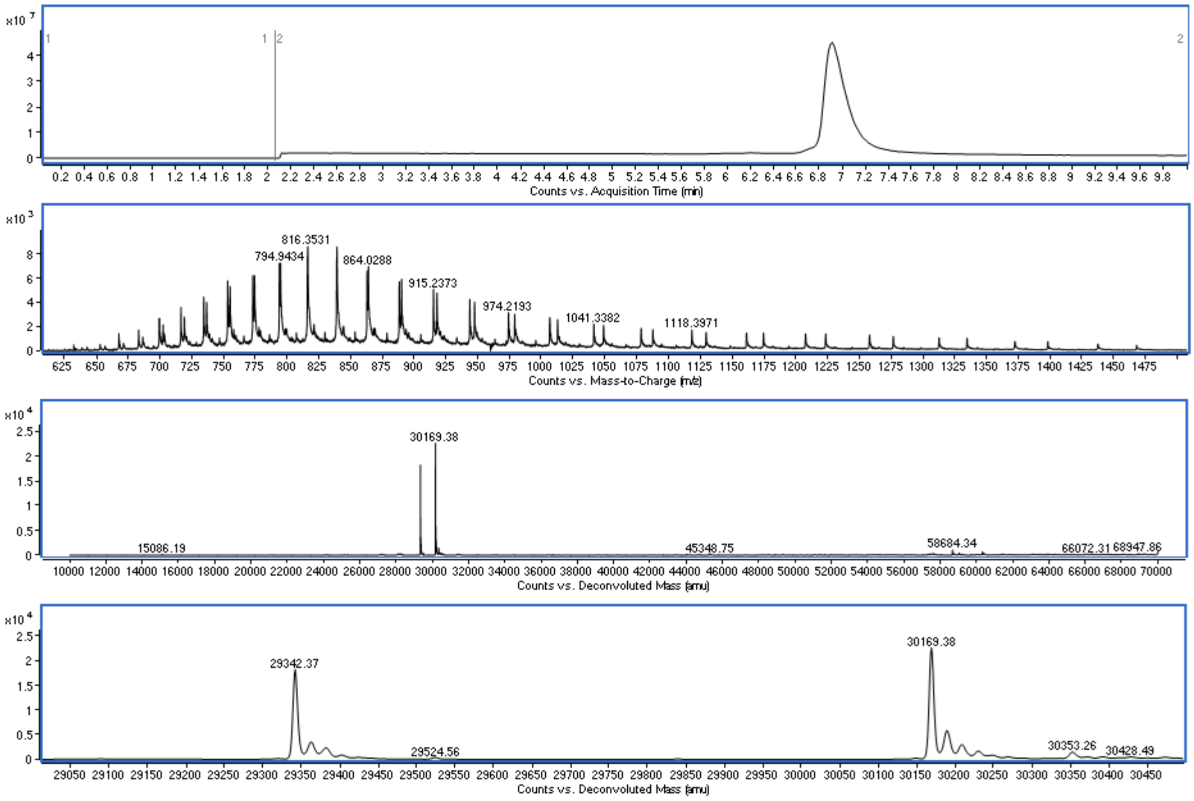

*Conjugate **38**, t = 6.5 h*

Expected masses: 29,342 Da (GFPS147C **32**); 29,604 Da (GFPS147C-PD **38**)
Observed masses: 29,343 Da; 29,603 Da; 29,621 Da

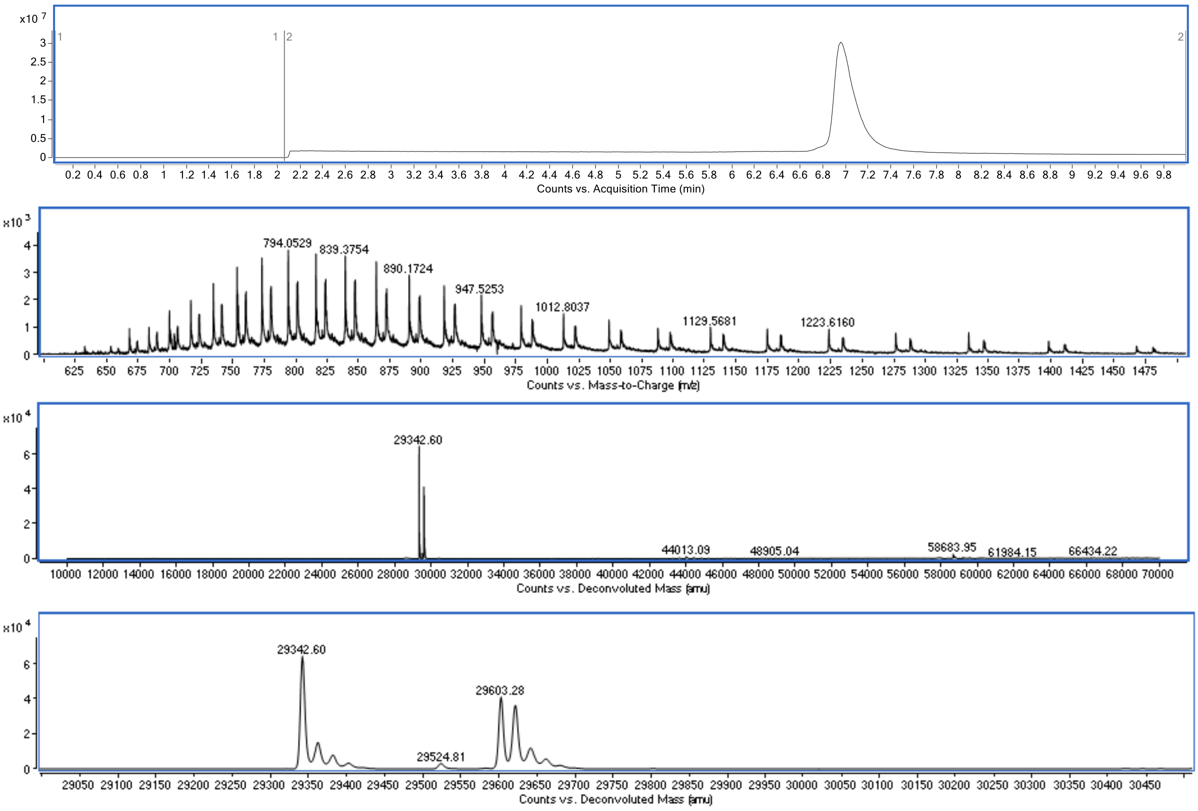

*Conjugate **39**, t = 6.5 h*

Expected masses: 29,342 Da (GFPS147C **32**); 29,649 Da (GFPS147C-PD **39**)
Observed masses: 29,342 Da; 29,455 Da; 29,648 Da

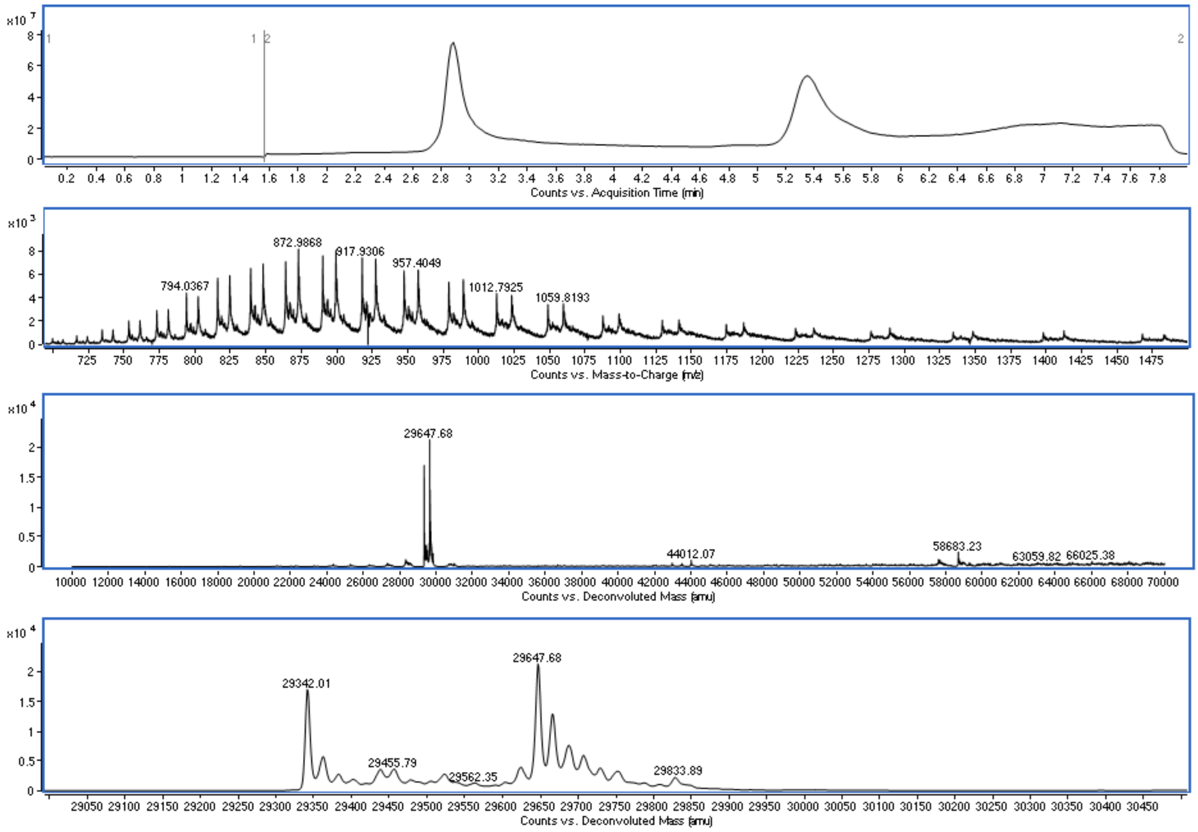

- Timepoint 24 h

*Conjugate **33**, t = 24 h*

Expected masses: 29,342 Da (GFPS147C **32**); 29,511 Da (GFPS147C-PD **33**)
Observed masses: 29,342 Da; 29,510 Da

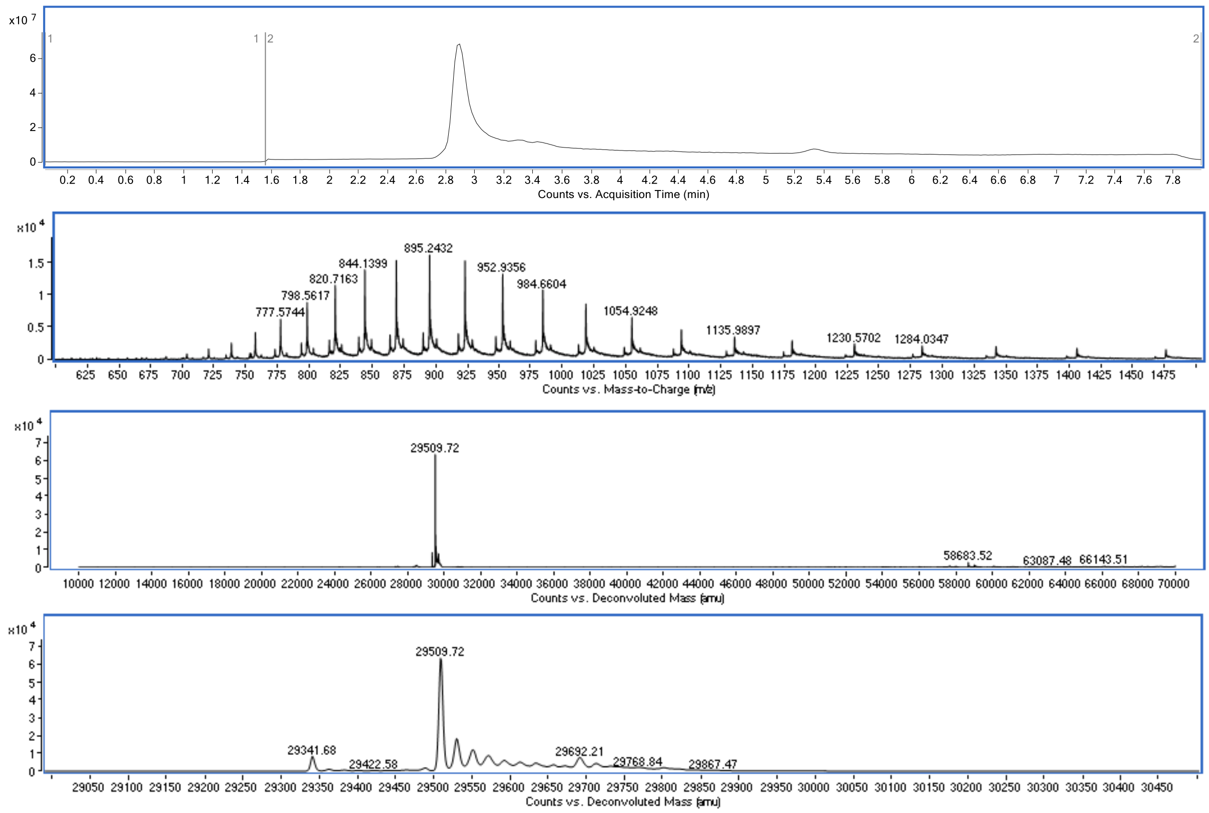

*Conjugate **34**, t = 24 h*

Expected masses: 29,342 Da (GFPS147C **32**); 30,108 Da (GFPS147C-PD **34**)
Observed masses: 29342 Da; 30,107 Da

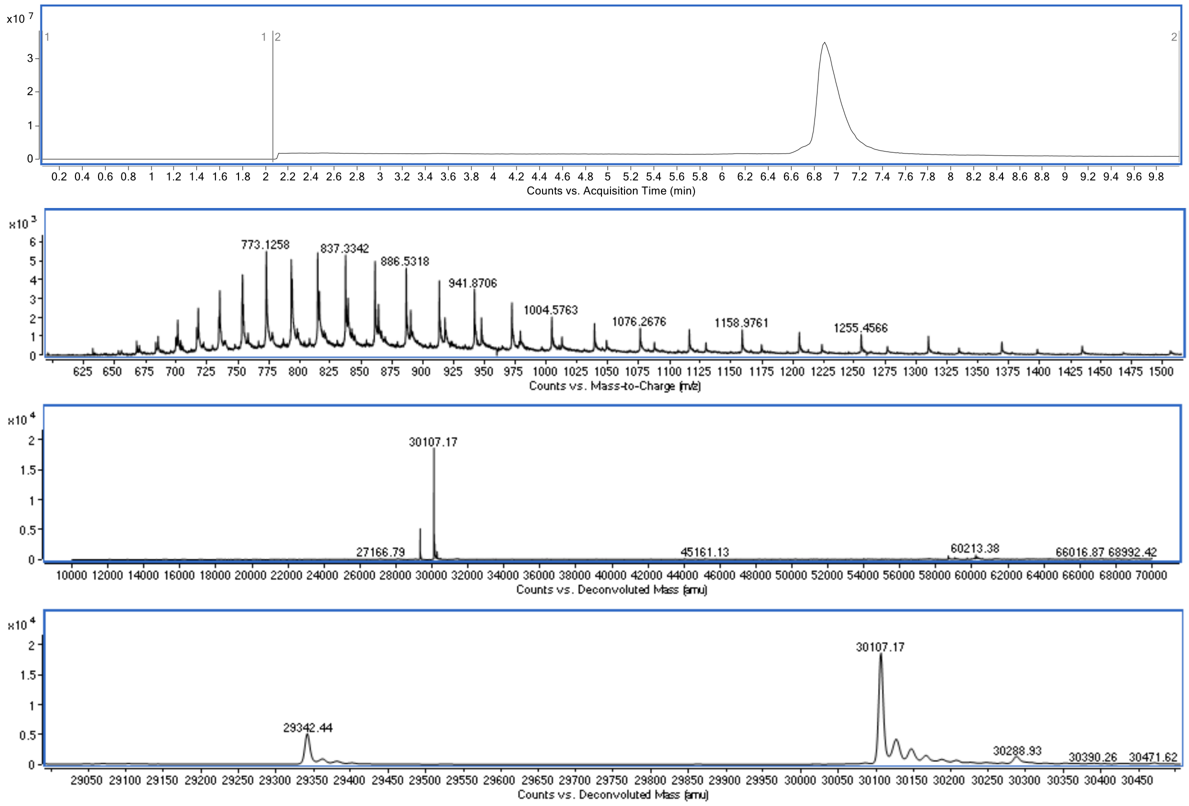

*Conjugate **35**, t = 24 h*

Expected masses: 29,342 Da (GFPS147C **32**); 29,574 Da (GFPS147C-PD **35**)
Observed masses: 29,342 Da; 29,573 Da

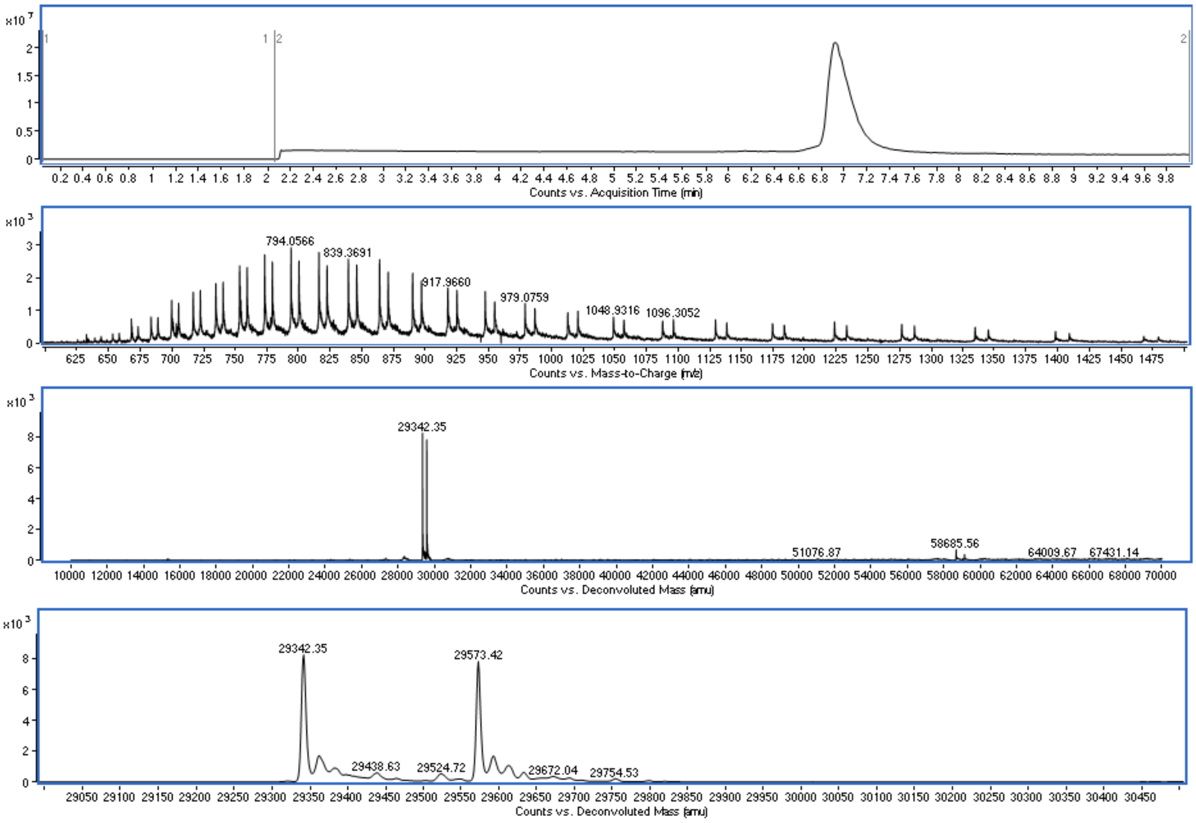

*Conjugate **36**, t = 24 h*

Expected masses: 29,342 Da (GFPS147C **32**); 29,559 Da (GFPS147C-PD **36**)
Observed masses : 29,342 Da; 29,558 Da

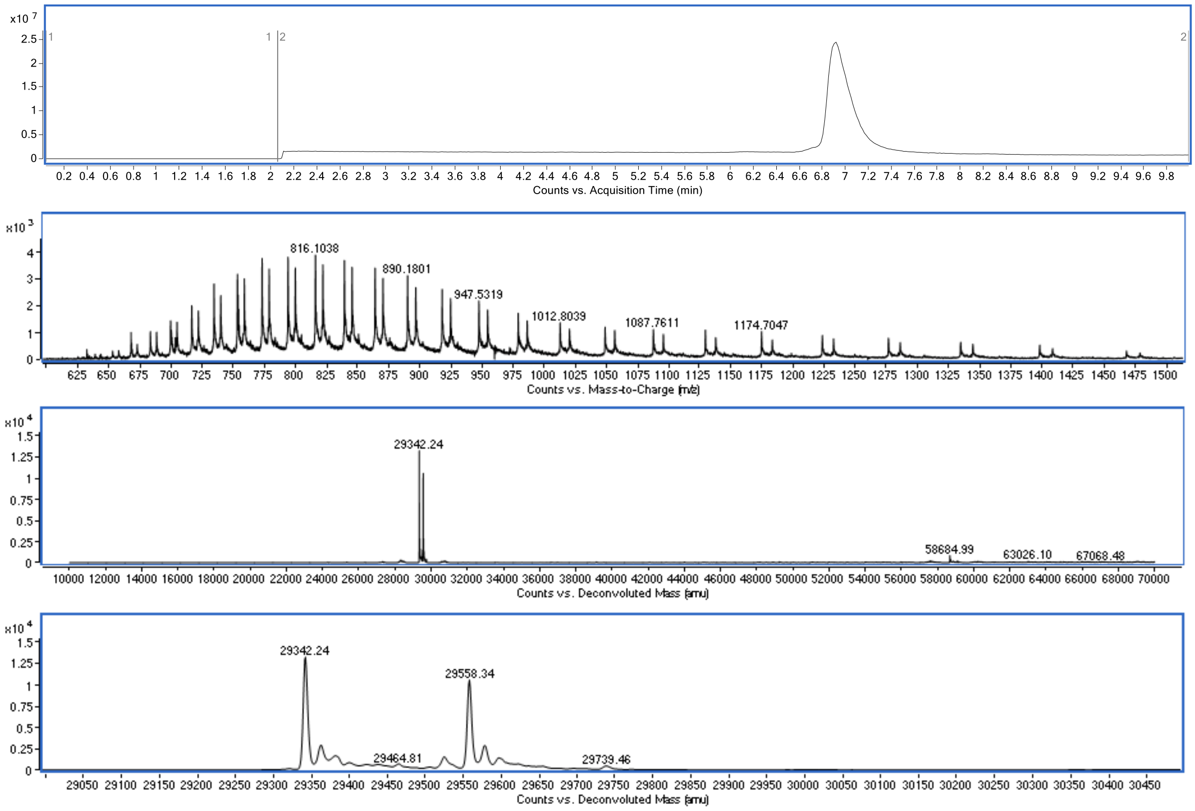

*Conjugate **S11**, t = 24 h*

Expected masses: 29,342 Da (GFPS147C **32**); 29,577 Da (GFPS147C-PD **S11**)
Observed masses: 29,342 Da; 29,576 Da

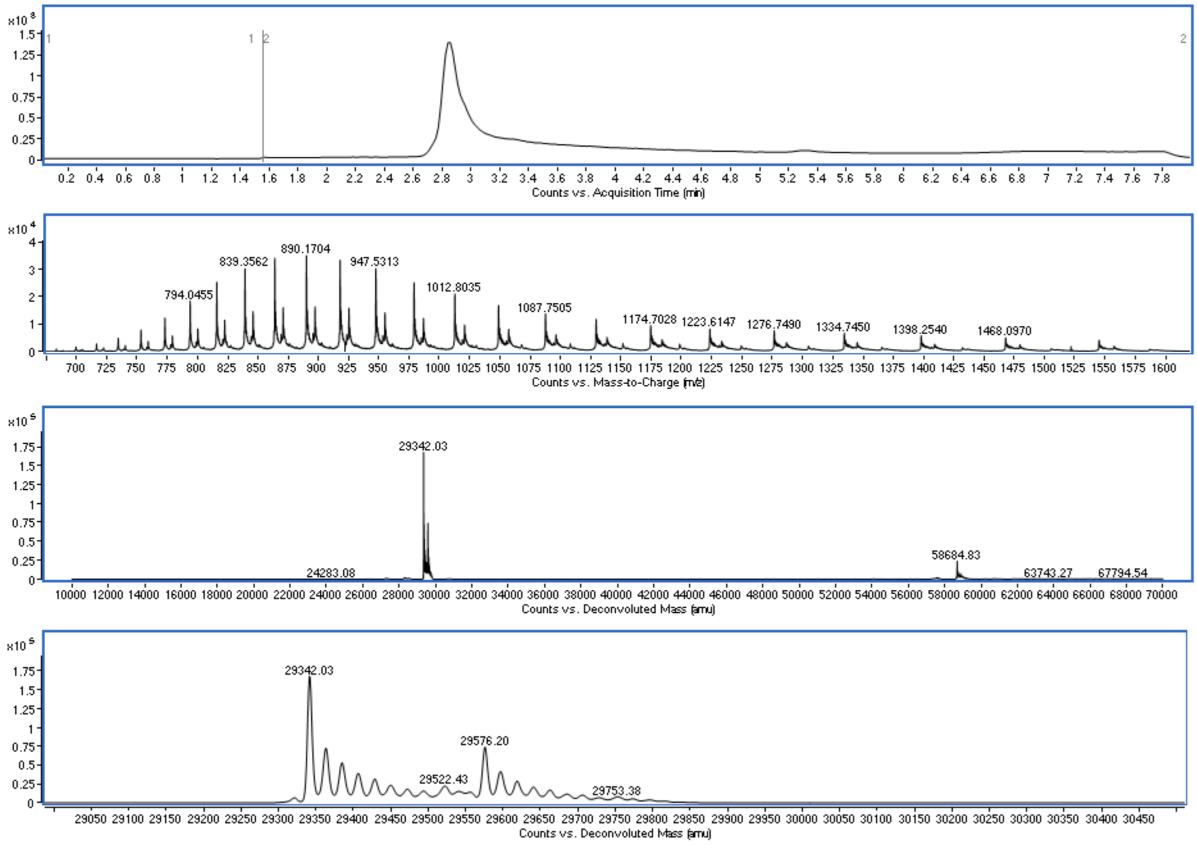

*Conjugate **37**, t = 24 h*

Expected masses: 29,342 Da (GFPS147C **32**); 30,170 Da (GFPS147C-PD **37**)
Observed masses : 29,342 Da; 30,169 Da

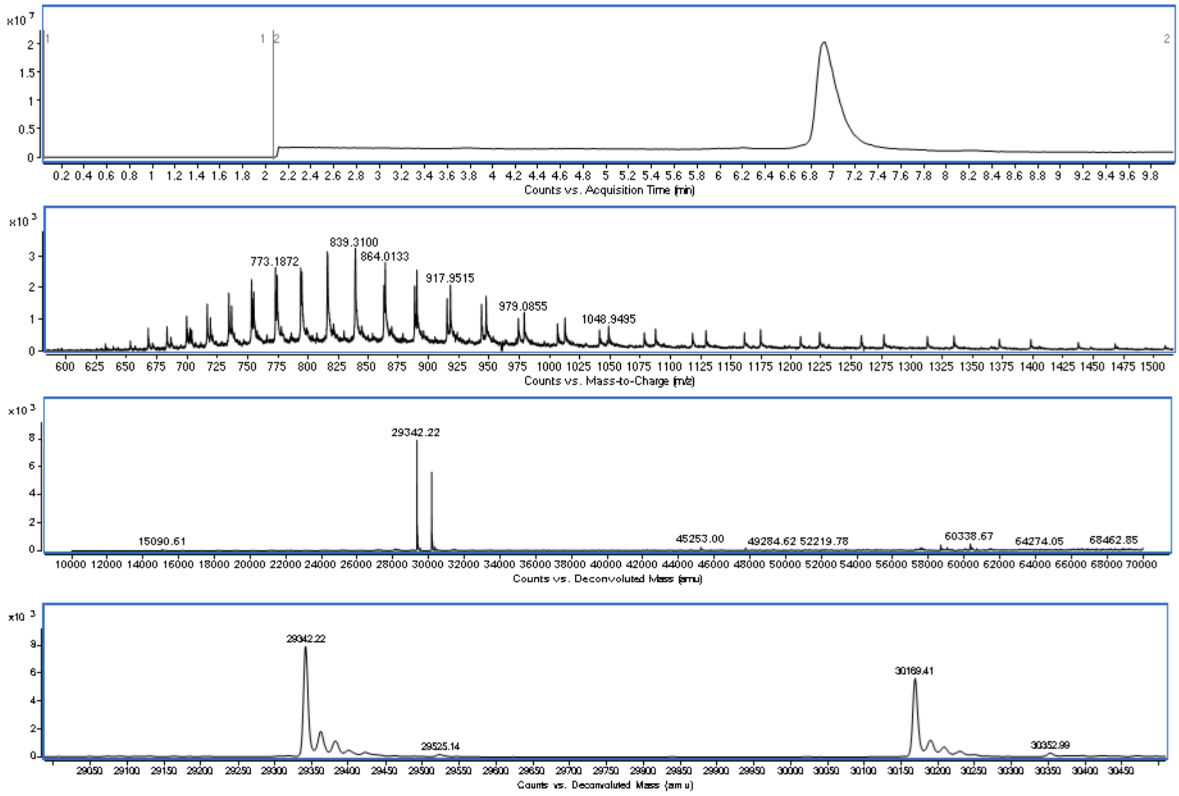

*Conjugate **38**, t = 24 h*

Expected masses: 29,342 Da (GFPS147C **32**); 29,604 Da (GFPS147C-PD **38**)
Observed masses: 29,342 Da; 29,603 Da; 29,621 Da

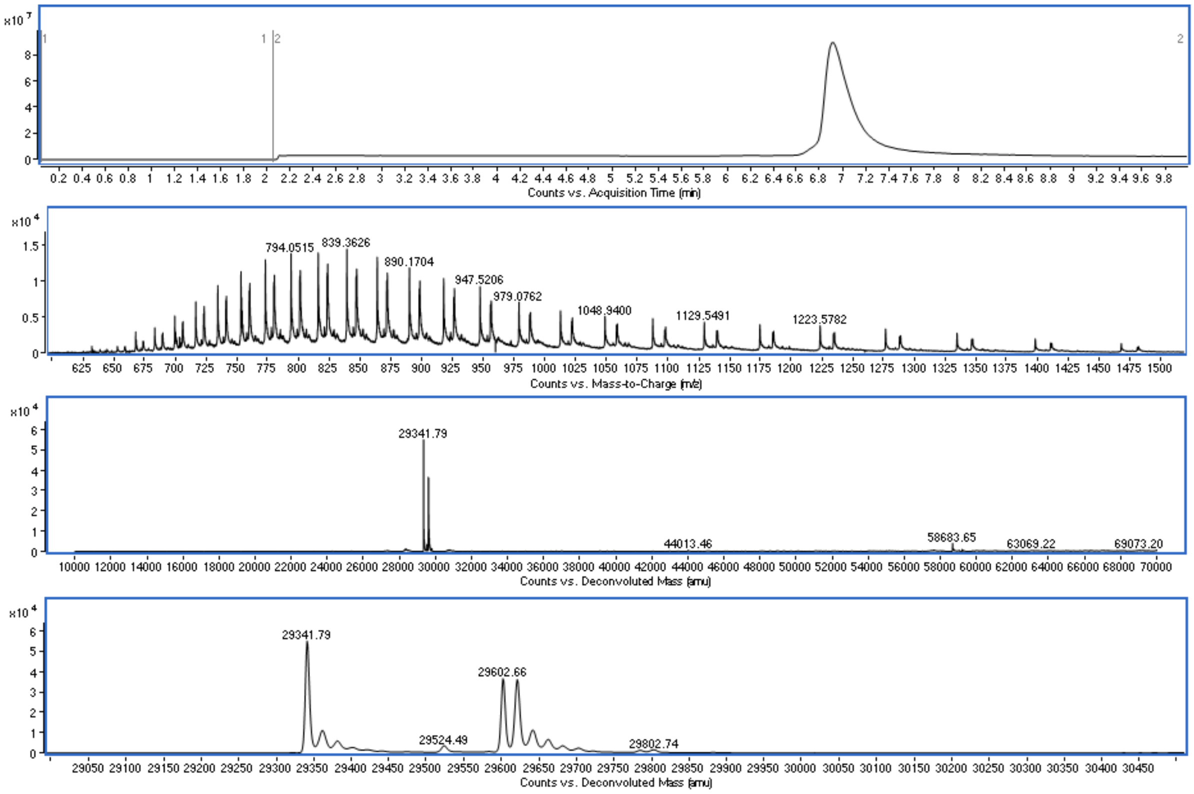

*Conjugate **39**, t = 24 h*

Expected masses: 29,342 Da (GFPS147C **32**); 29,649 Da (GFPS147C-PD **39**)
Observed masses: 29,342 Da; 29,457 Da; 29,648 Da; 26,667 Da

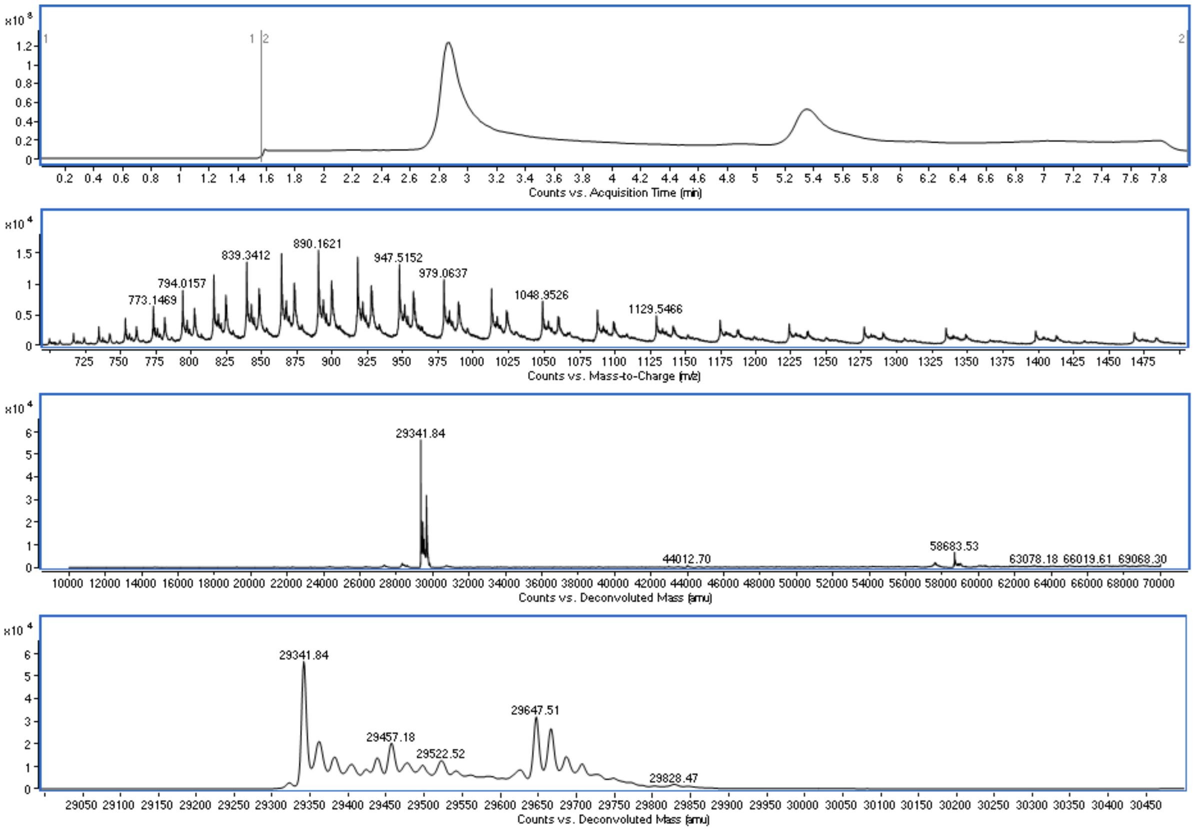

- Timepoint 48 h

*Conjugate **33**, t = 48 h*

Expected masses: 29,342 Da (GFPS147C **32**); 29,511 Da (GFPS147C-PD **33**)
Observed masses: 29,342 Da; 29,510 Da

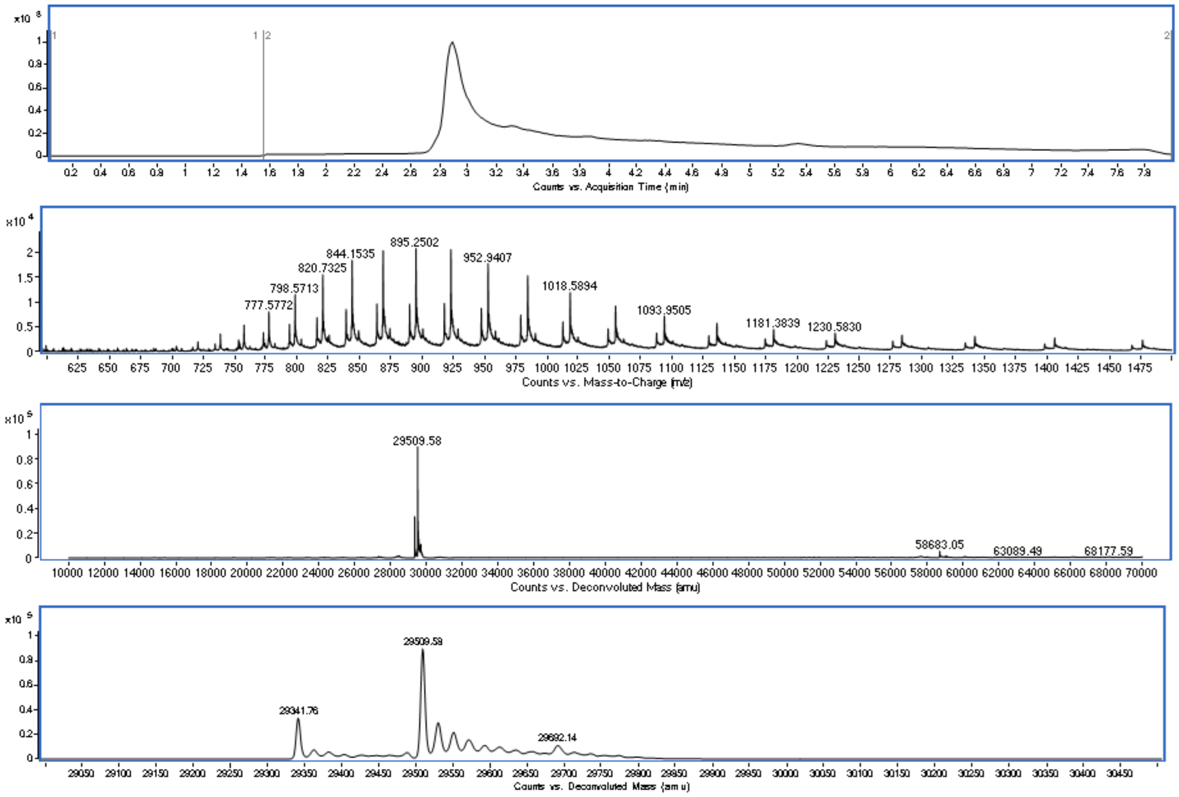

*Conjugate **34**, t = 48 h*

Expected masses: 29,342 Da (GFPS147C **32**); 30,108 Da (GFPS147C-PD **34**)

Observed masses: 29,342 Da; 30,107 Da

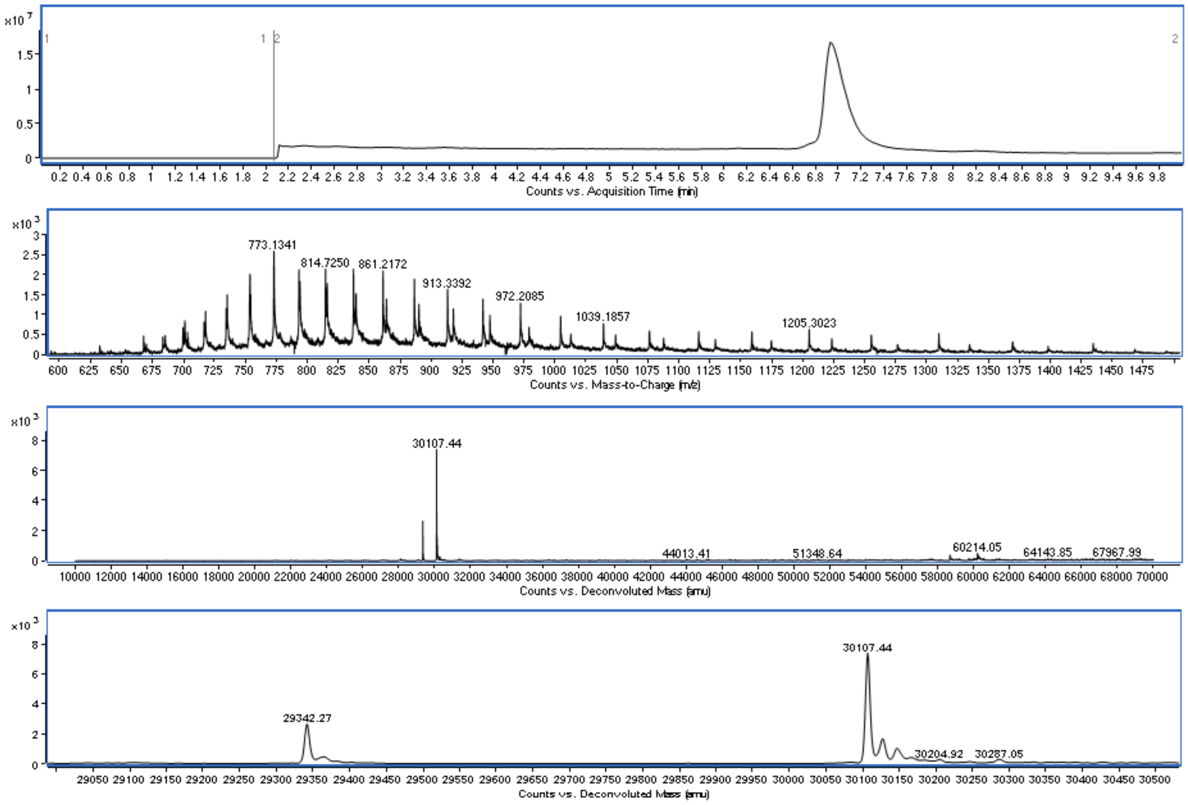

*Conjugate **35**, t = 48 h*

Expected masses: 29,342 Da (GFPS147C **32**); 29,574 Da (GFPS147C-PD **35**)
Observed masses: 29,342 Da; 29,574 Da

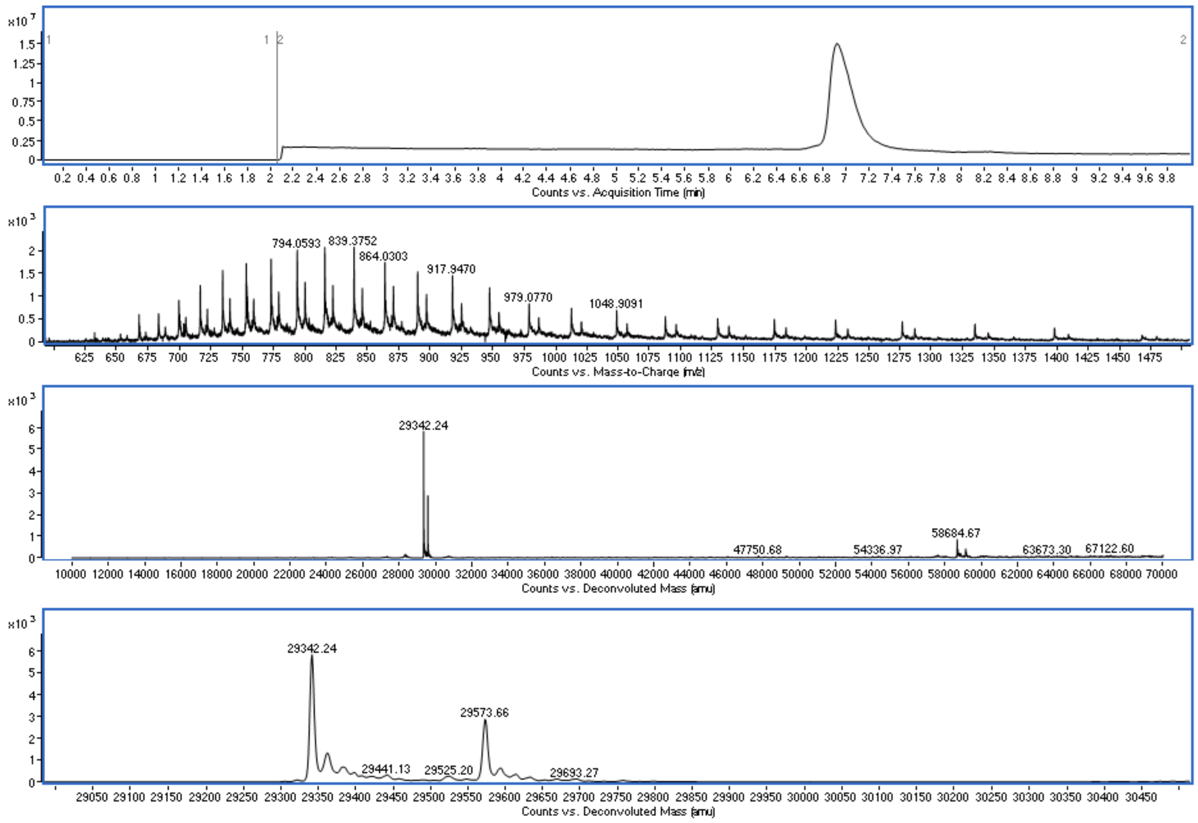

*Conjugate **36**, t = 48 h*

Expected masses: 29,342 Da (GFPS147C **32**); 29,559 Da (GFPS147C **36**)

Observed masses: 29,342 Da; 29,559 Da; 58,685 Da

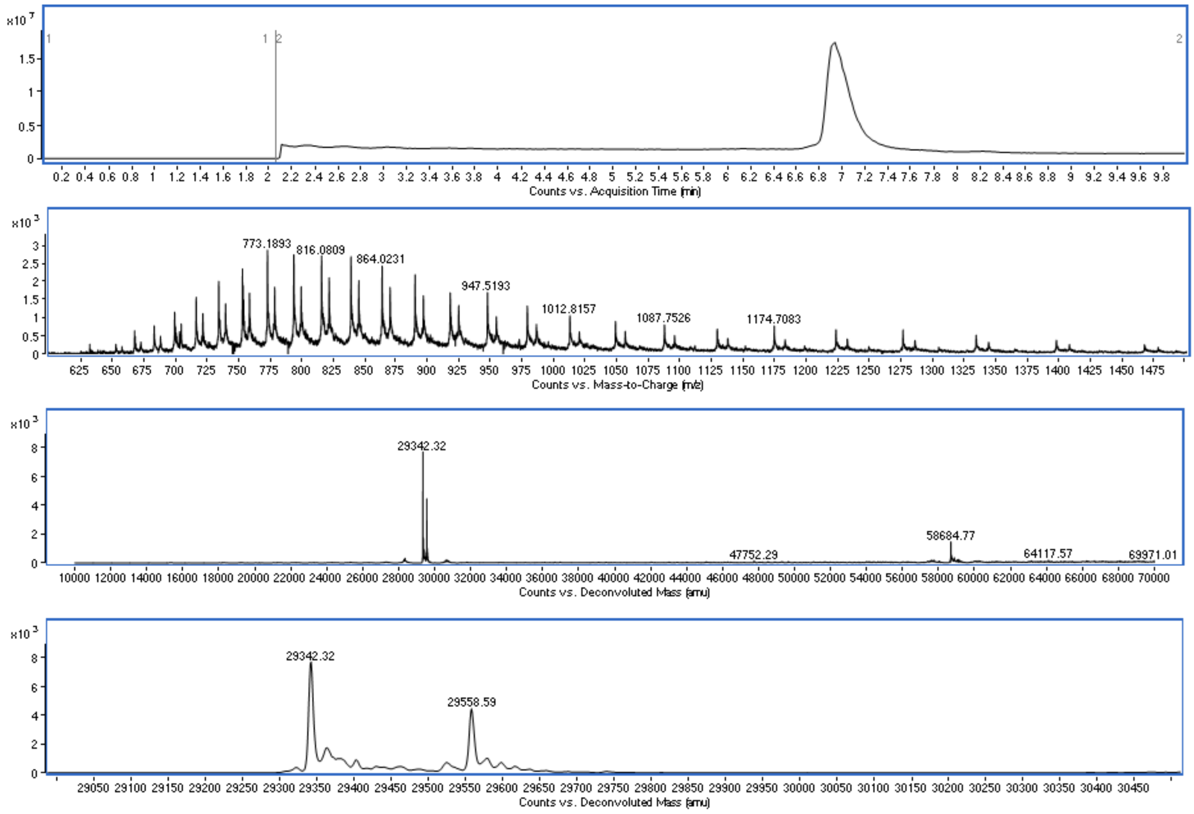

*Conjugate **S11**, t = 48 h*

Expected masses: 29,342 Da (GFPS147C **32**); 29,577 Da (GFPS147C-PD **S11**)
Observed masses: 29,342 Da; 29,576 Da

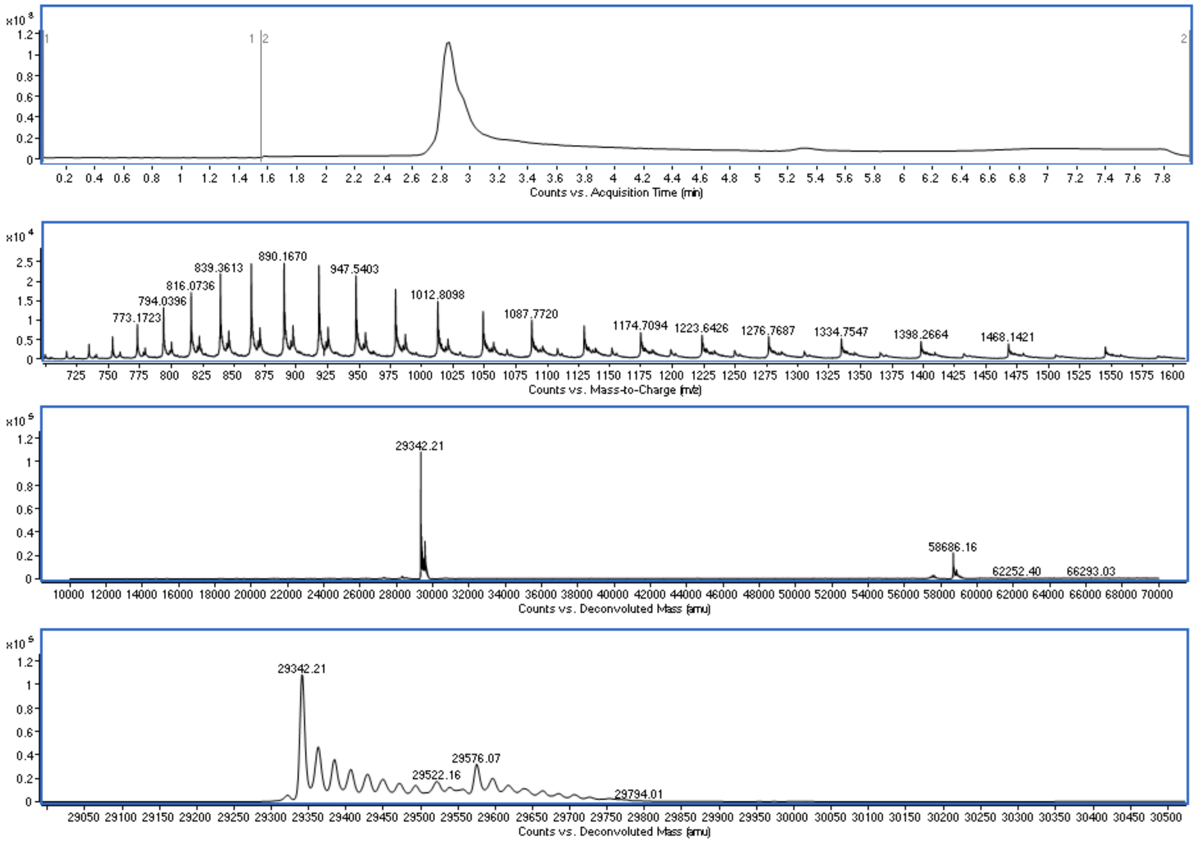

*Conjugate **37**, t = 48 h*

Expected masses: 29,342 Da (GFPS147C **32**); 30,170 Da (GFPS147C-PD **37**)
Observed masses: 29,342 Da; 30,169 Da.

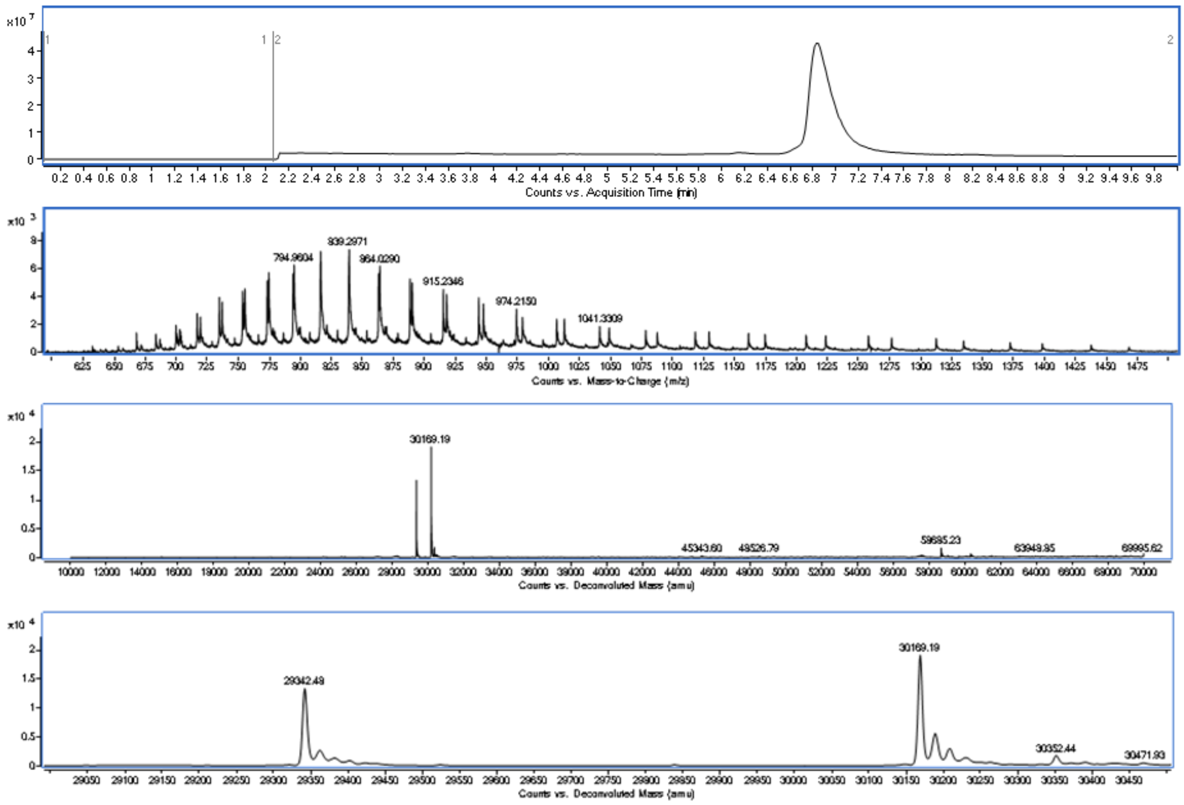

*Conjugate **38**, t = 48 h*

Expected masses: 29,342 Da (GFPS147C **32**); 29,604 Da (GFPS147C-PD **38**)
Observed masses: 29,342 Da; 29,603 Da; 29622 Da

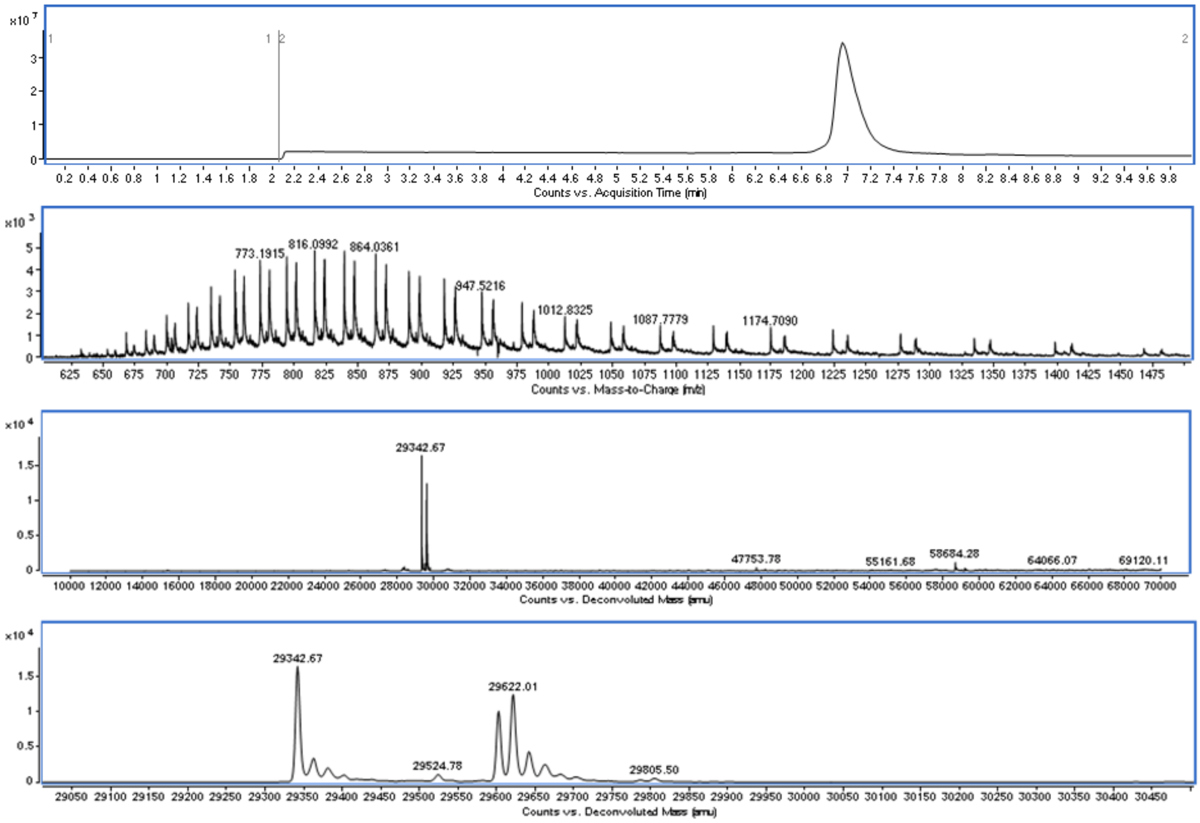

*Conjugate **39**, t = 48 h*

Expected masses: 29,342 Da (GFPS147C **32**); 29,649 Da (GFPS147C-PD **39**)
Observed masses: 29,342 Da; 29,457 Da; 29,648 Da; 29,667 Da

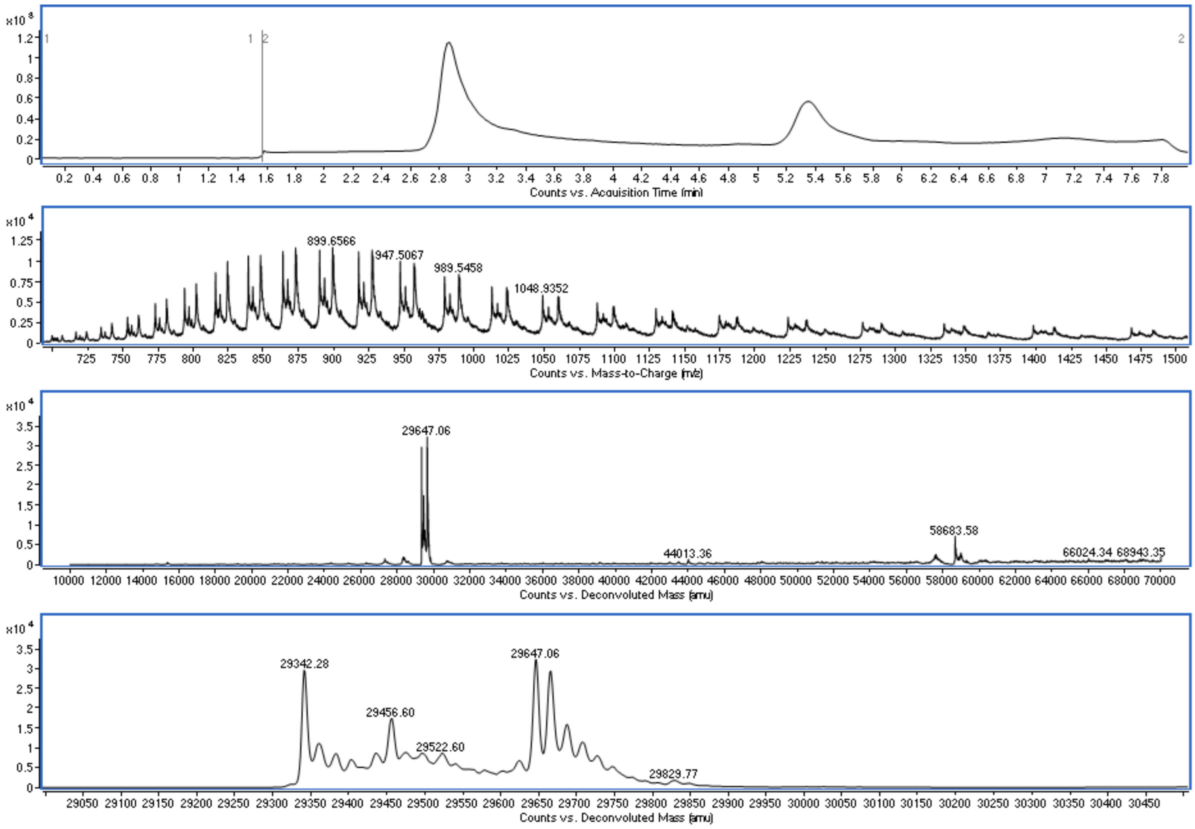

- Timepoint 72 h

*Conjugate **33**, t = 72 h*

Expected masses: 29,342 Da (GFPS147C **32**); 29,511 Da (GFPS147C-PD **33**)
Observed masses: 29,342 Da; 29,510 Da

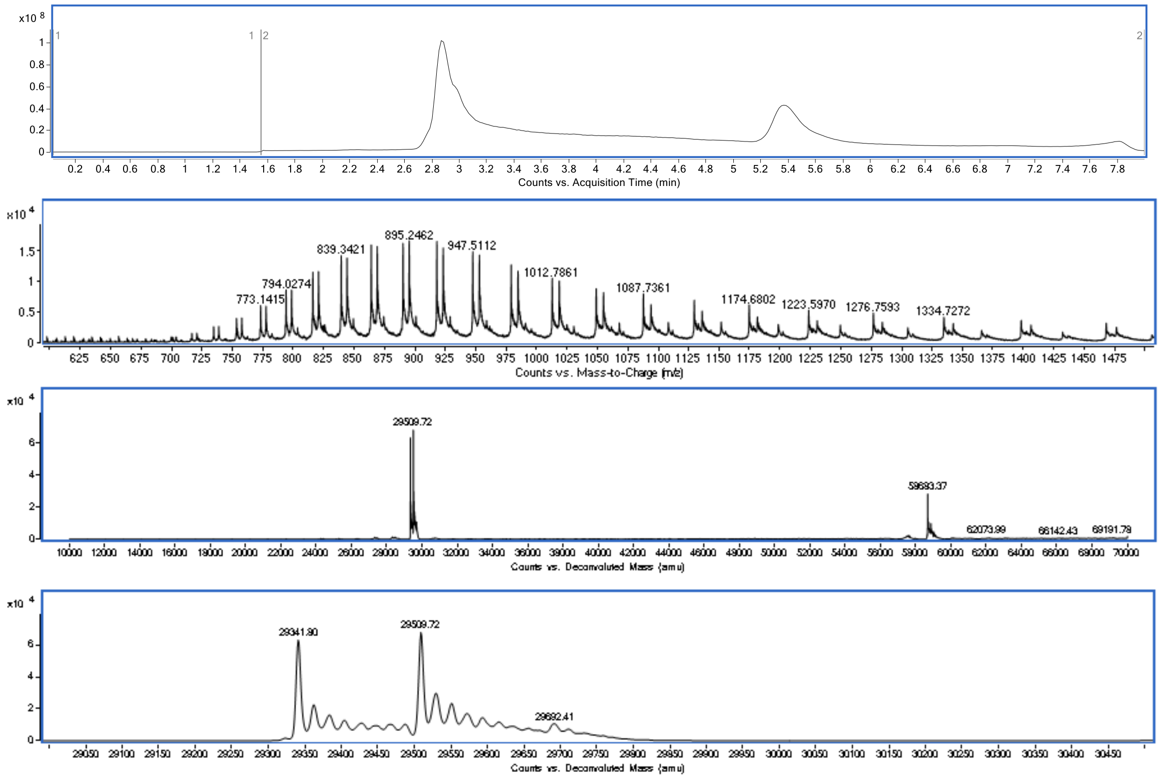

*Conjugate **34**, t = 72 h*

Expected masses: 29,342 Da (GFPS147C **32**); 30,108 Da (GFPS147C-PD **34**)
Observed masses: 29,342 Da; 30,108 Da

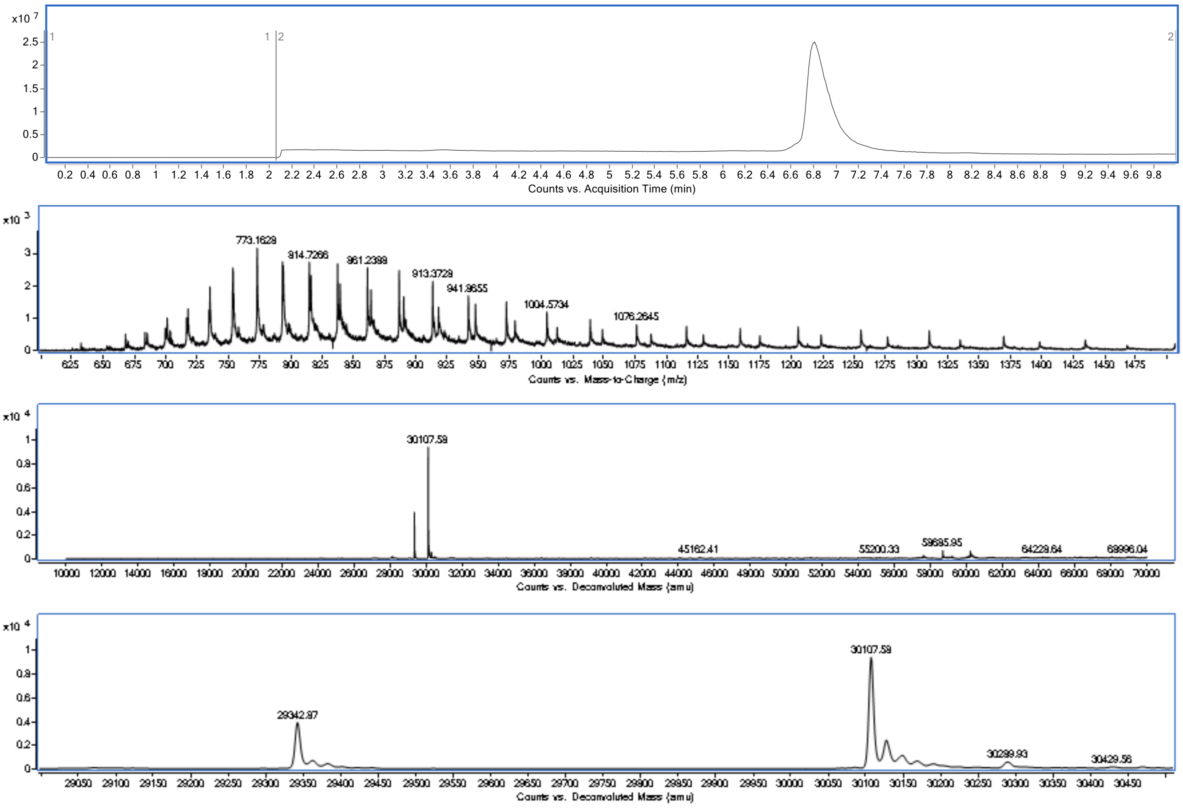

*Conjugate **35**, t = 72 h*

Expected masses: 29,342 Da (GFPS147C **32**); 29,574 Da (GFPS147C-PD **35**)
Observed masses: 29,342 Da; 29,574 Da

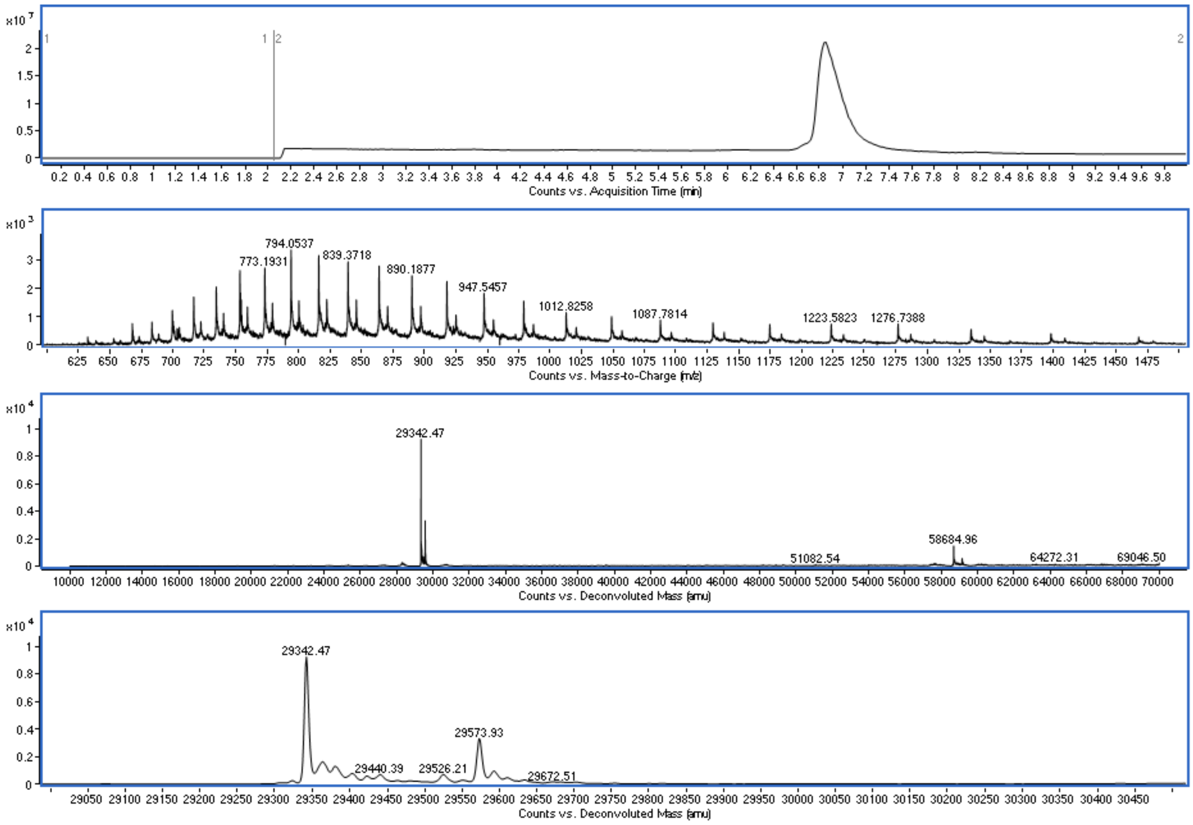

*Conjugate **36**, t = 72 h*

Expected masses: 29,342 Da (GFPS147C **32**); 29,559 Da (GFPS147C-PD **36**)
Observed masses: 29,342 Da; 29,559 Da

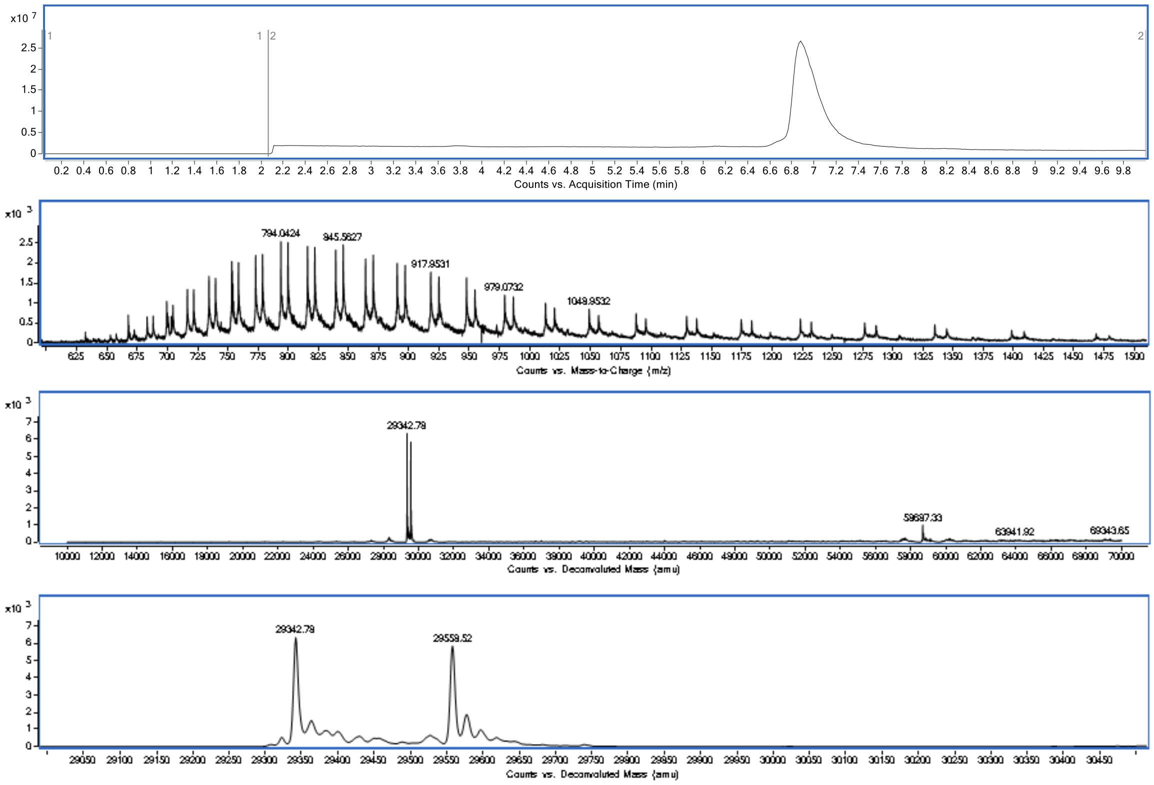

*Conjugate **S11**, t = 72 h*

Expected masses: 29,342 Da (GFPS147C **32**); 29,577 Da (GFPS147C-PD **S11**)
Observed masses: 29,342 Da; 29,576 Da

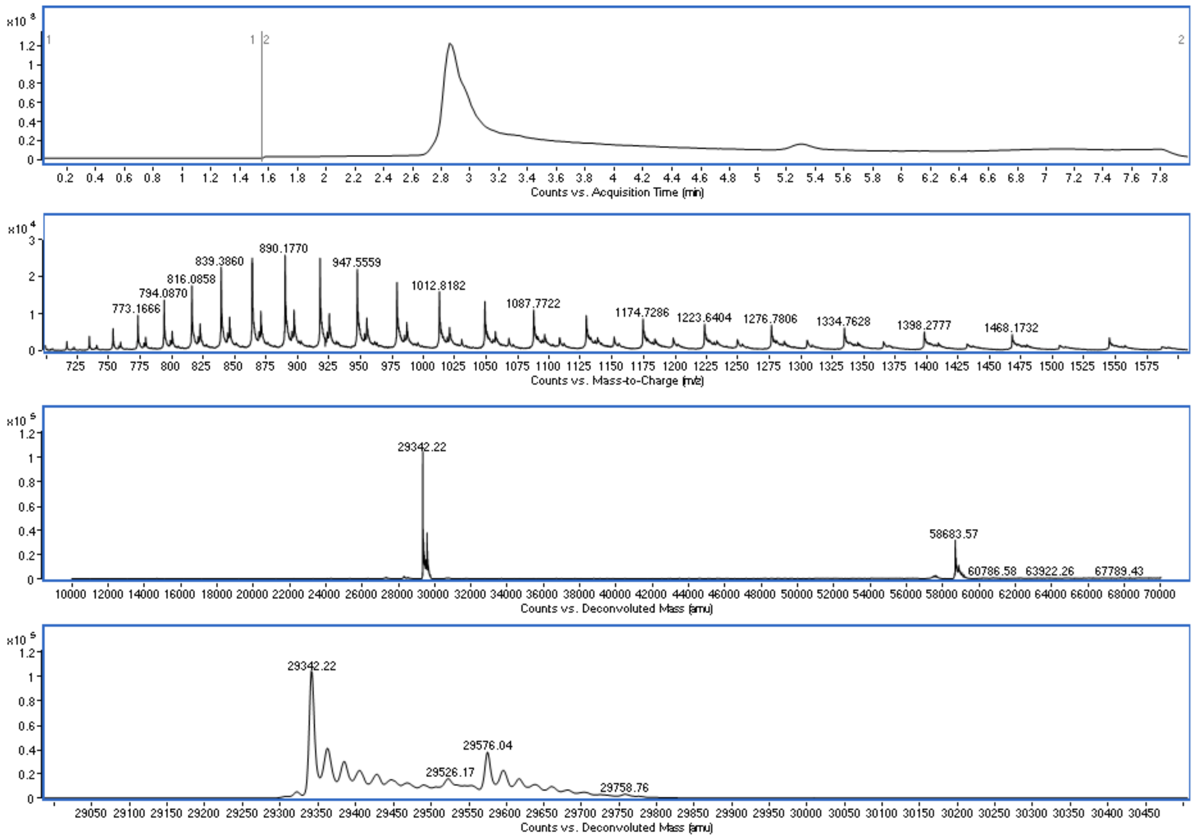

*Conjugate **37**, t = 72 h*

Expected masses: 29,342 Da (GFPS147C **32**); 30,170 Da (GFPS147C-PD **37**)
Observed masses: 29,342 Da; 30,169 Da

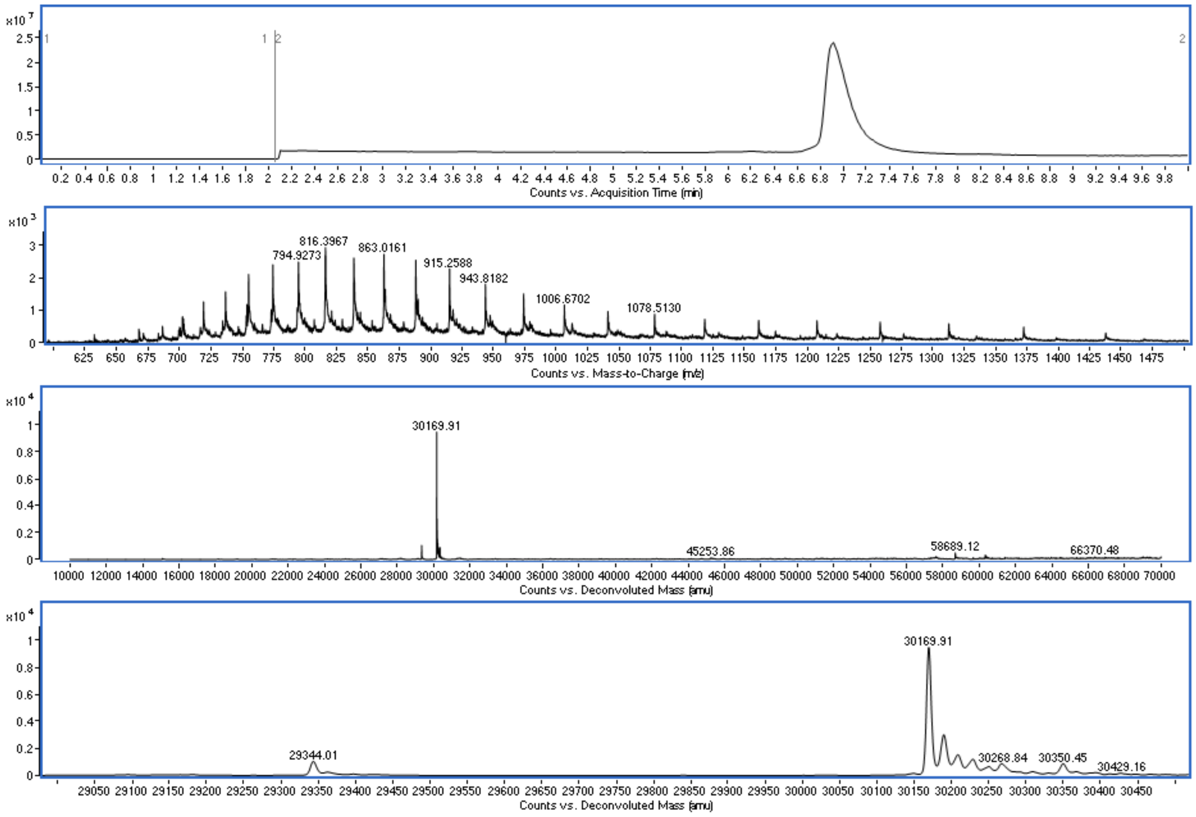

*Conjugate **38**, t = 72 h*

Expected masses: 29,342 Da (GFPS147C **32**); 29,604 Da (GFPS147C-PD **38**)
Observed masses: 29,342 Da; 29,603 Da; 29,622 Da

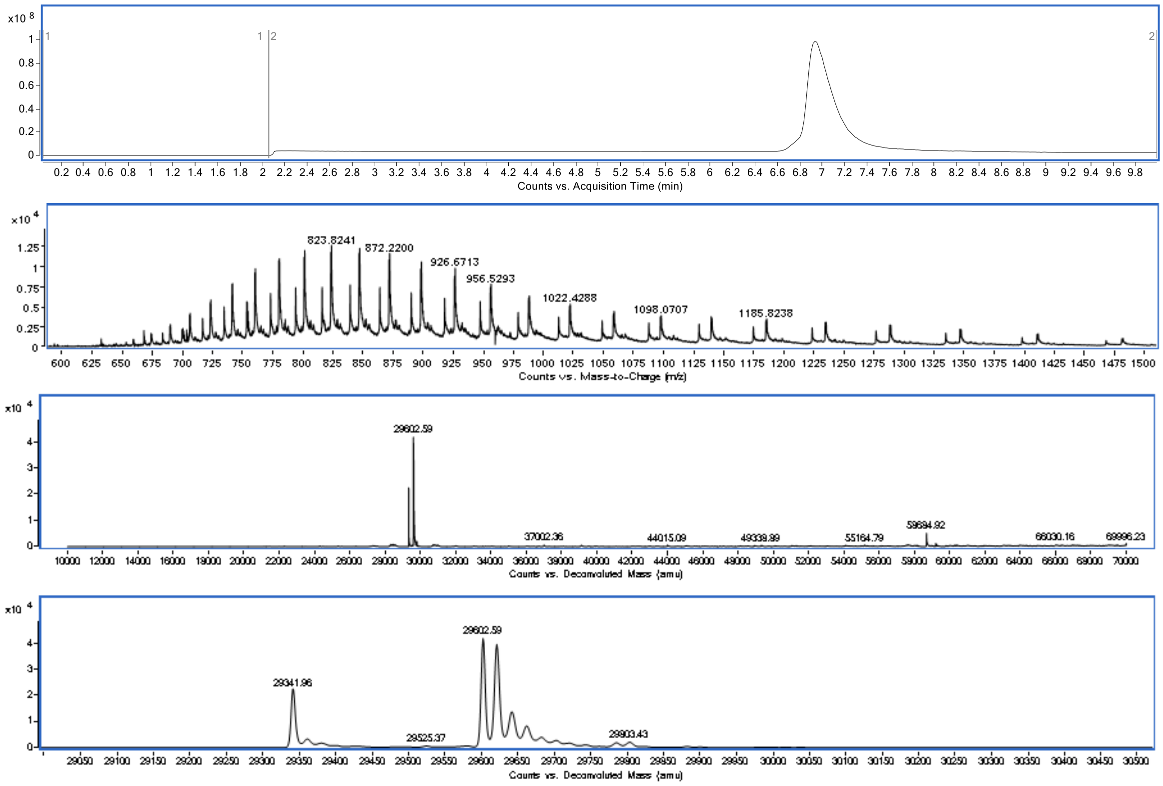

*Conjugate **39**, t = 72 h*

Expected masses: 29,342 Da (GFPS147C **32**); 29,649 Da (GFPS147C-PD **39**)

Observed masses: 29,343 Da; 29,458 Da; 29,647 Da; 29,666 Da

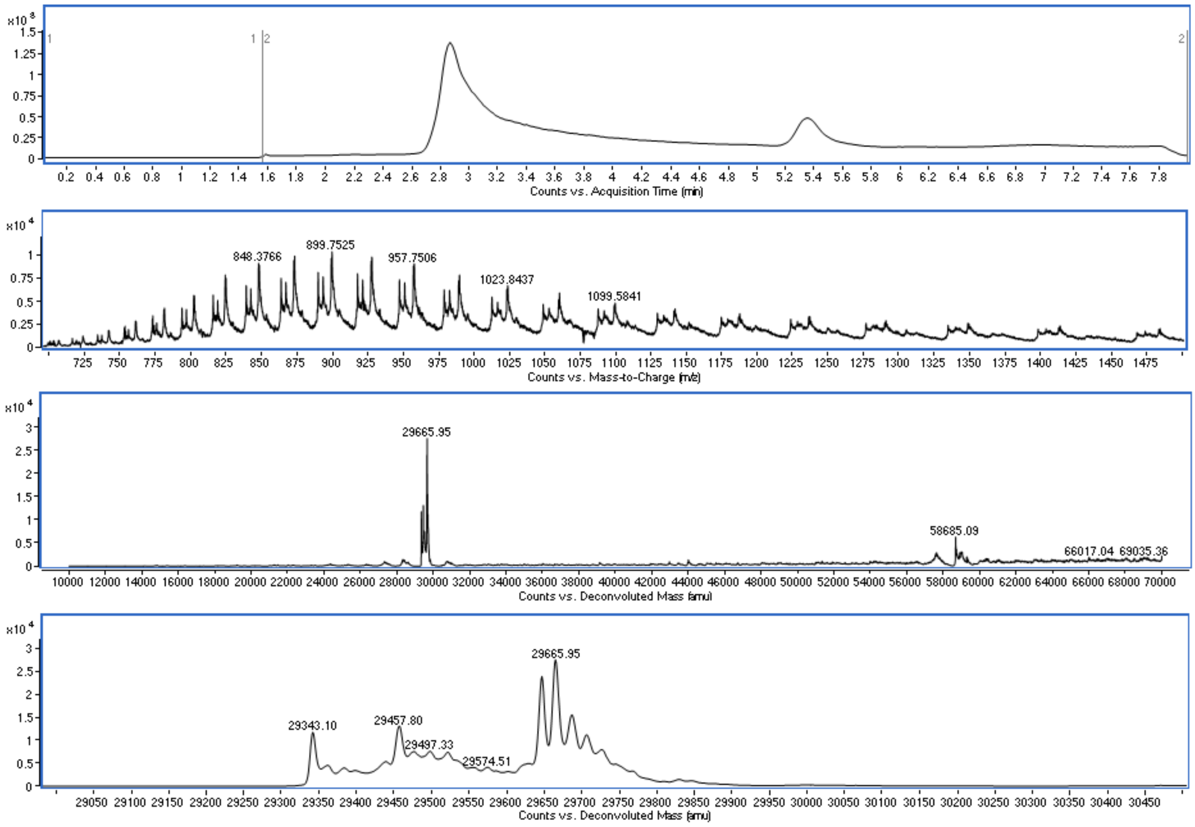

- *Summary*

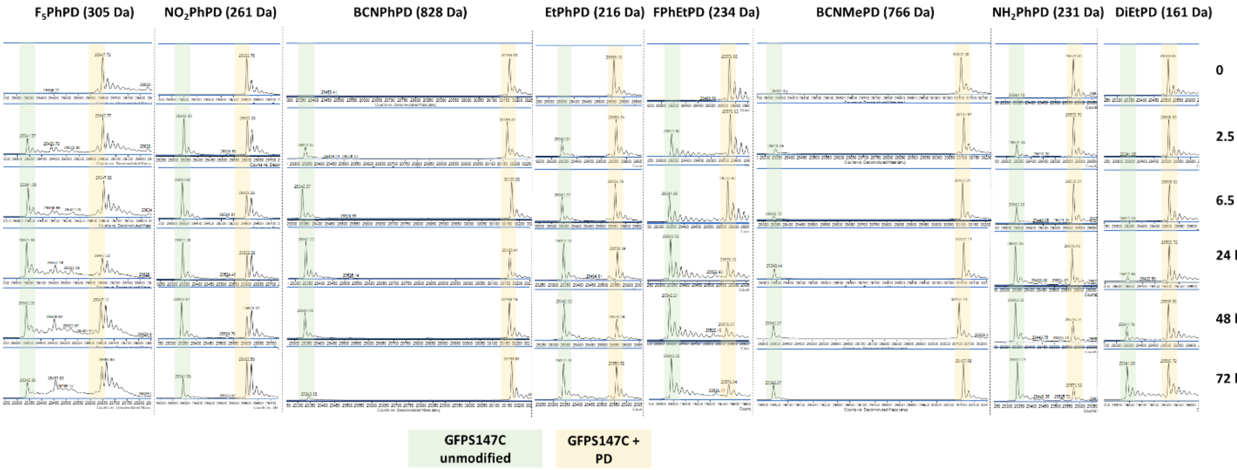

*Figure S3. Summary of deconjugation study from GFPS147C **32** at each timepoint for each PD **1-6, 30** and **31** synthesised.*

- *SDS-PAGE gel*

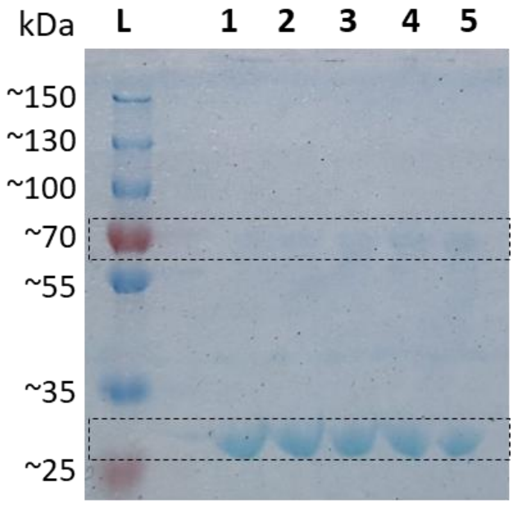

Figure S4. SDS-PAGE gel representing each timepoint for N-Et,N'-Ph PD-GFP ***36*** *conjugate, enabling to monitor the formation of GFPS147C dimer* ***S10*** *slowly overtime. Expected masses: ~29 kDa (GFPS147C/GFPS147C-conjugate) and/or ~58 kDa (GFPS147C dimer).* ***Lanes 1-6****: respectively timepoint 0, 6.5 h, 24 h, 48 h and 72 h.*

4.1.4 Hydrolysis study

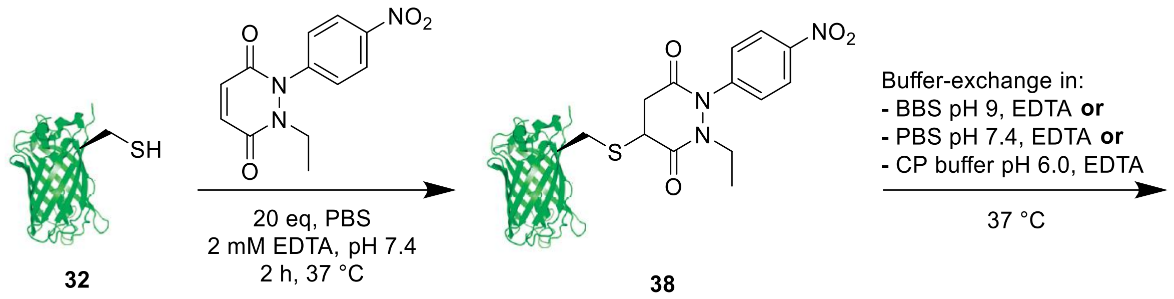

To a solution of reduced GFPS147C **32** (100 µL, 70 µM in PBS EDTA, 0.007 µmol) was added PD **5** (2.8 µL, 50 mM in MeCN, 20 equiv., 0.14 µmol) and the reaction was incubated at 37 °C for 2 h. Excess of small molecules was removed using Zeba™ Spin Desalting column in LCMS grade water. LCMS analysis had to be carried out quickly after removal of the small molecules to avoid premature deconjugation of the PD. Conjugated GFPS147C **38** was then split in 3 batches and each one was buffer exchanged respectively in BBS pH 9.0 (2 mM EDTA), PBS pH 7.4 (2 mM EDTA), CP buffer pH 6.0 (2 mM EDTA) using Zeba™ Spin Desalting column. Concentration was adjusted to 35 µM and LC-MS analyses were taken after 2.5 h, 6.5 h, 21 h, 30 h, 2 days, 3 days, 5 days and 9 days. LCMS analysis was carried out quickly after removal of the small molecules (within 10 min) to avoid deconjugation of the PD from the GFPS147C-PD conjugate **38** during analysis.

Expected masses: 29,342 Da (GFPS147C); 29,603 Da (GFPS147C-PD **38**); 29,622 Da (GFPS147C-PD hydrolysed).

- Timepoint 2.5 h

pH 9.0

Observed masses: 29,342; 29,603 Da

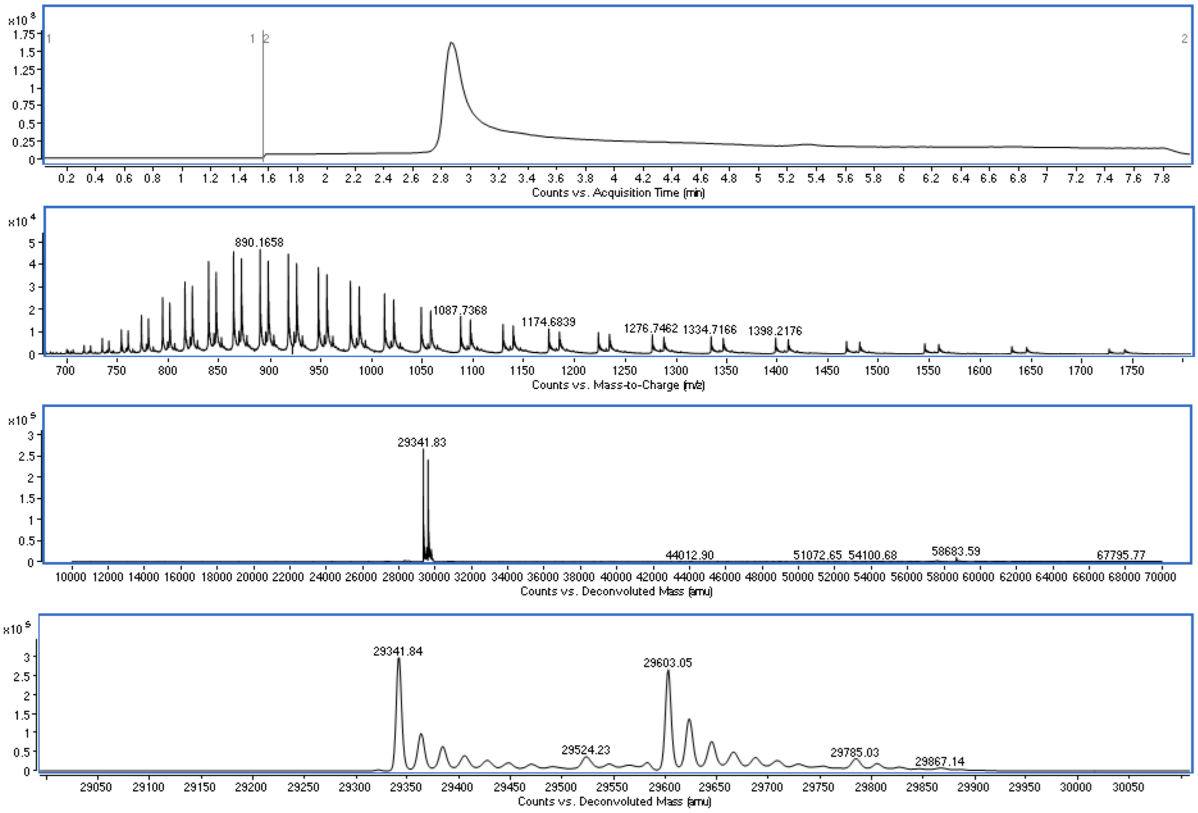

pH 7.4

Observed masses: 29,342; 29,603 Da

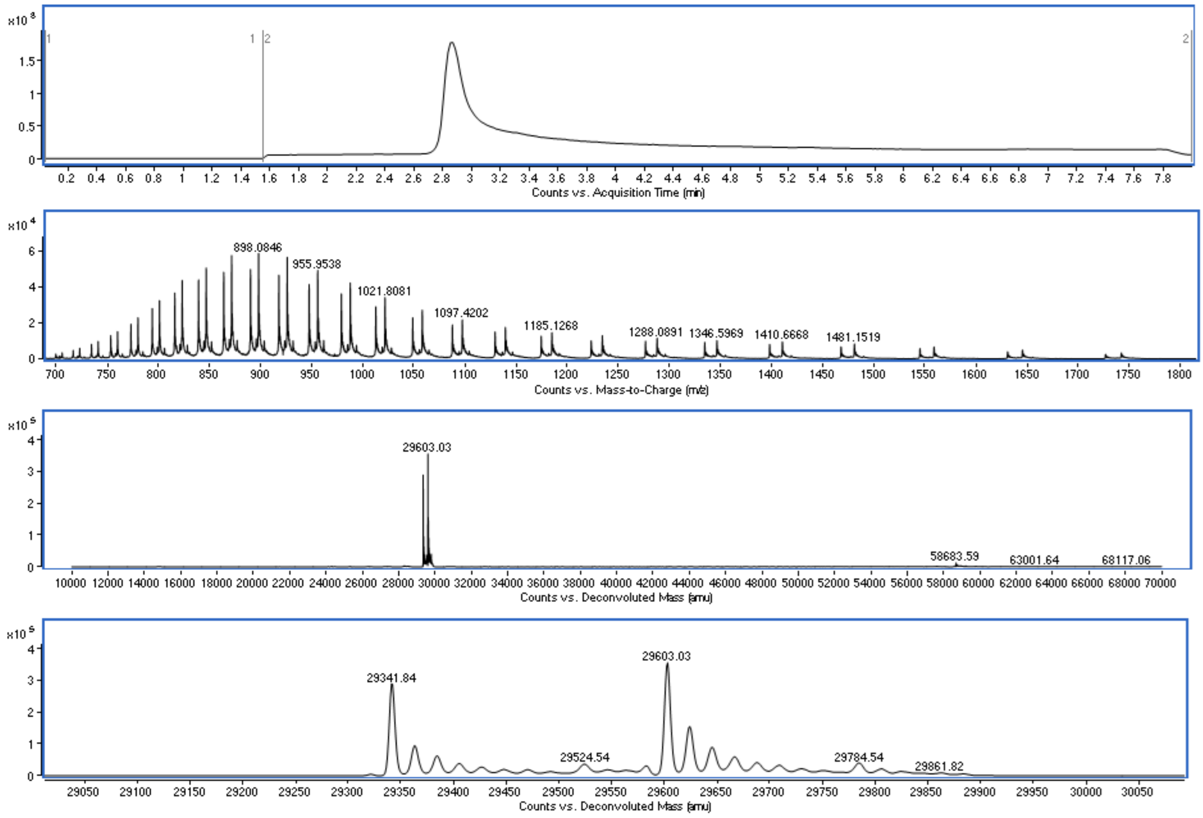

pH 6.0

Observed masses: 29,342; 29,603 Da

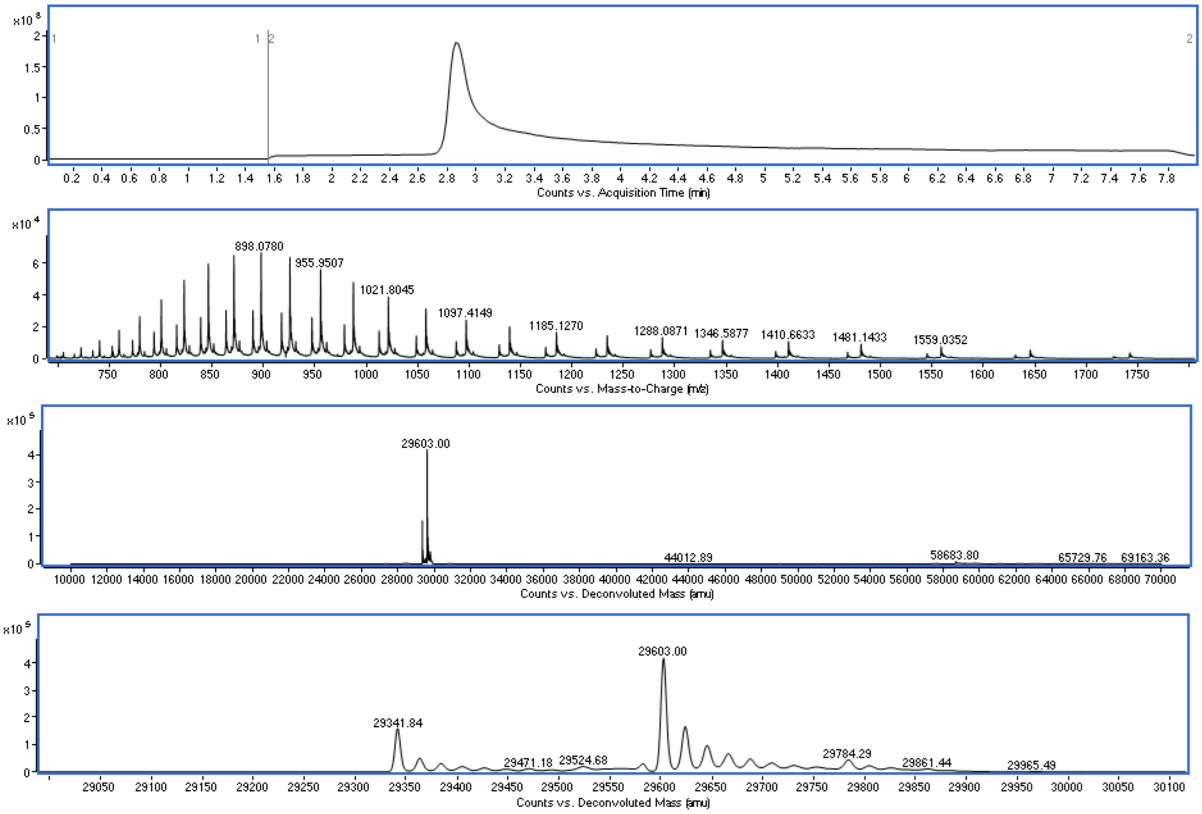

- Timepoint 6.5 h

pH 9.0

Observed masses: 29,342; 29,603; 29,622 Da

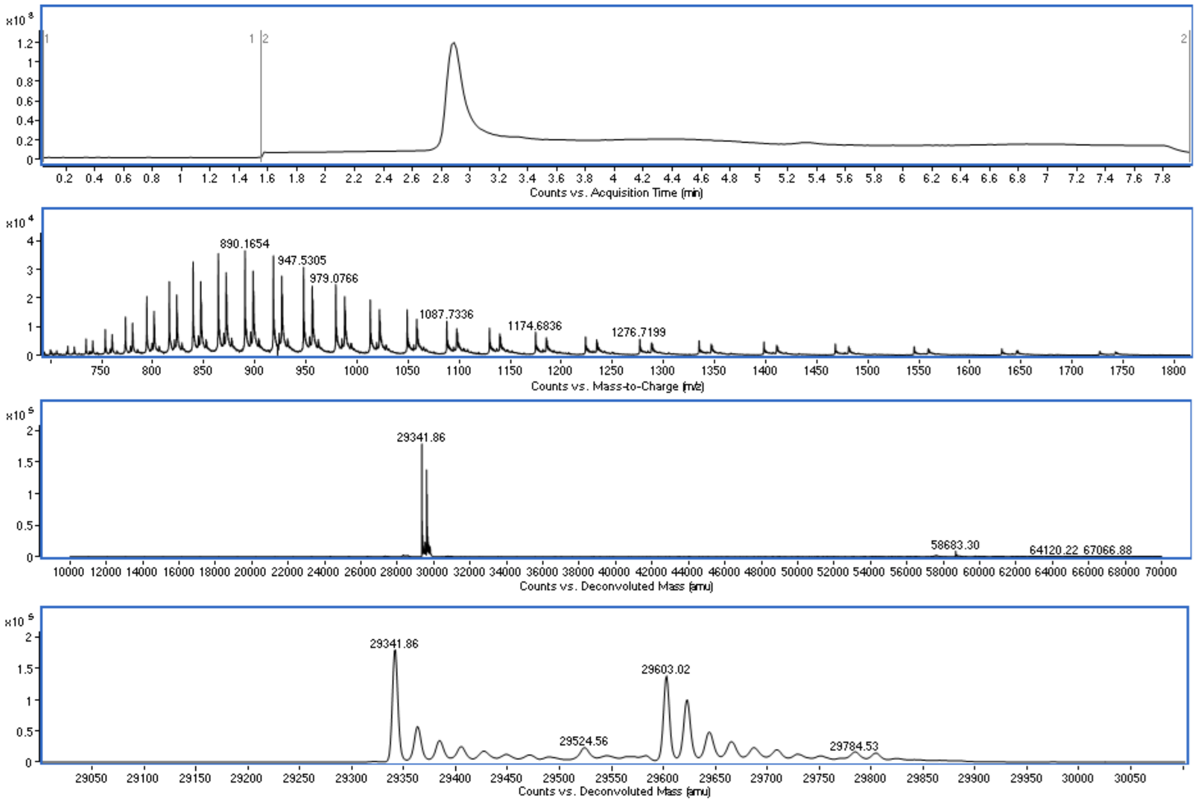

pH 7.4

Observed masses: 29,342; 29,603 Da

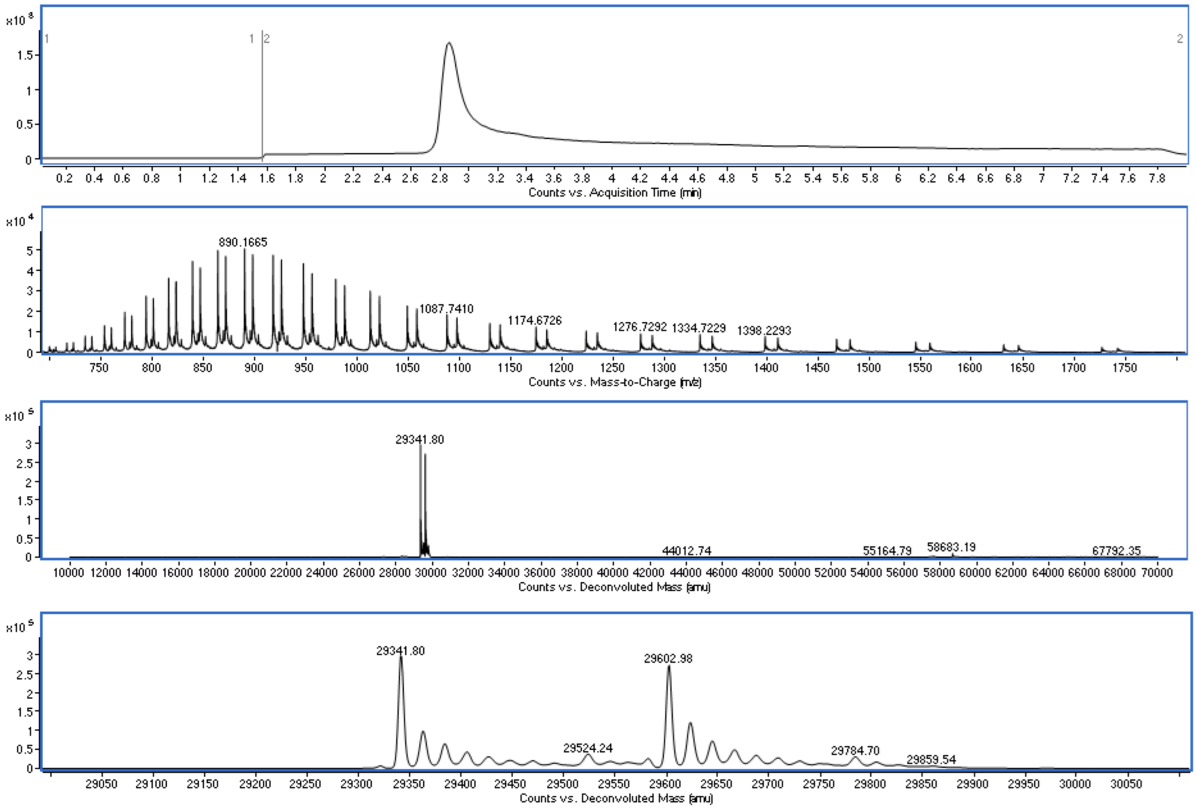

pH 6.0

Observed masses: 29,342; 29,603 Da

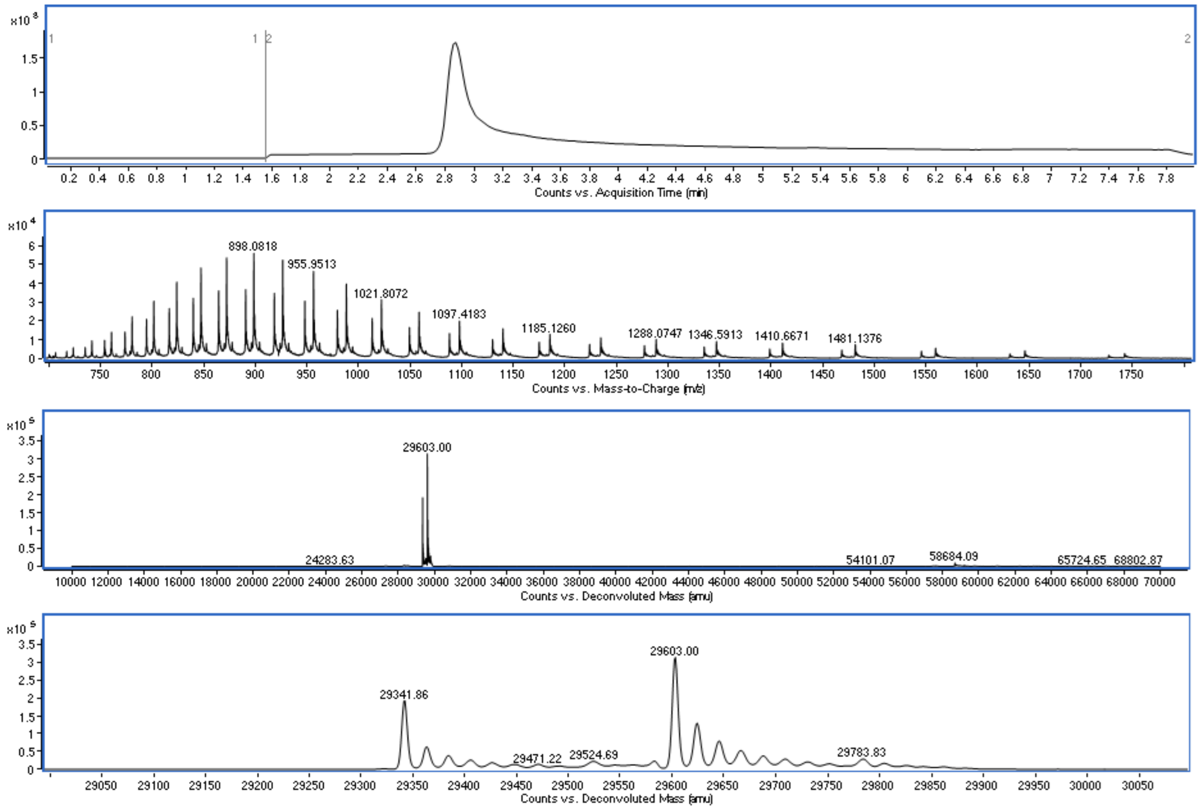

- Timepoint 21 h

pH 9.0

Observed masses: 29,342; 29,603; 29,622 Da

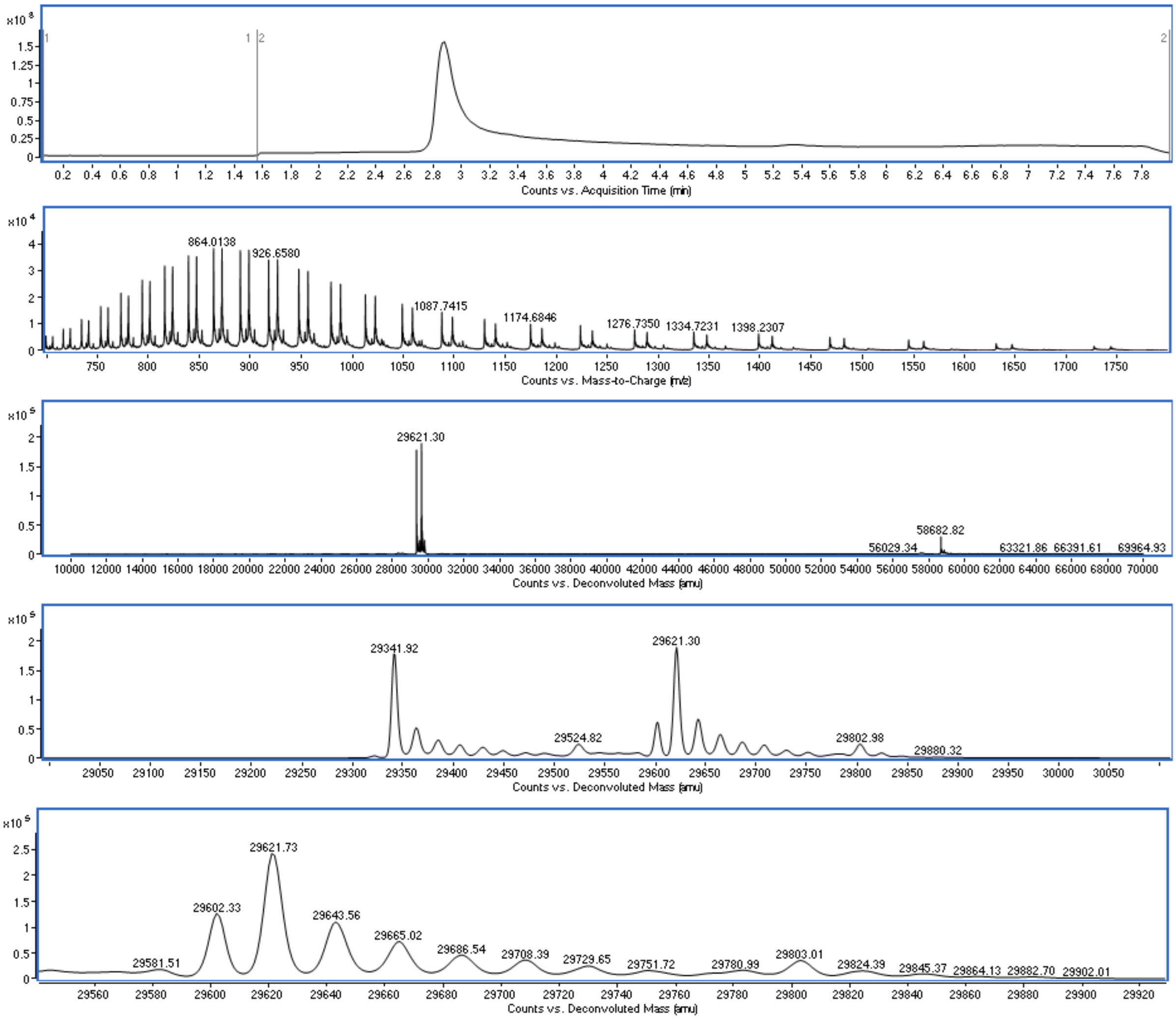

pH 7.4

Observed masses: 29,342; 29,603 Da

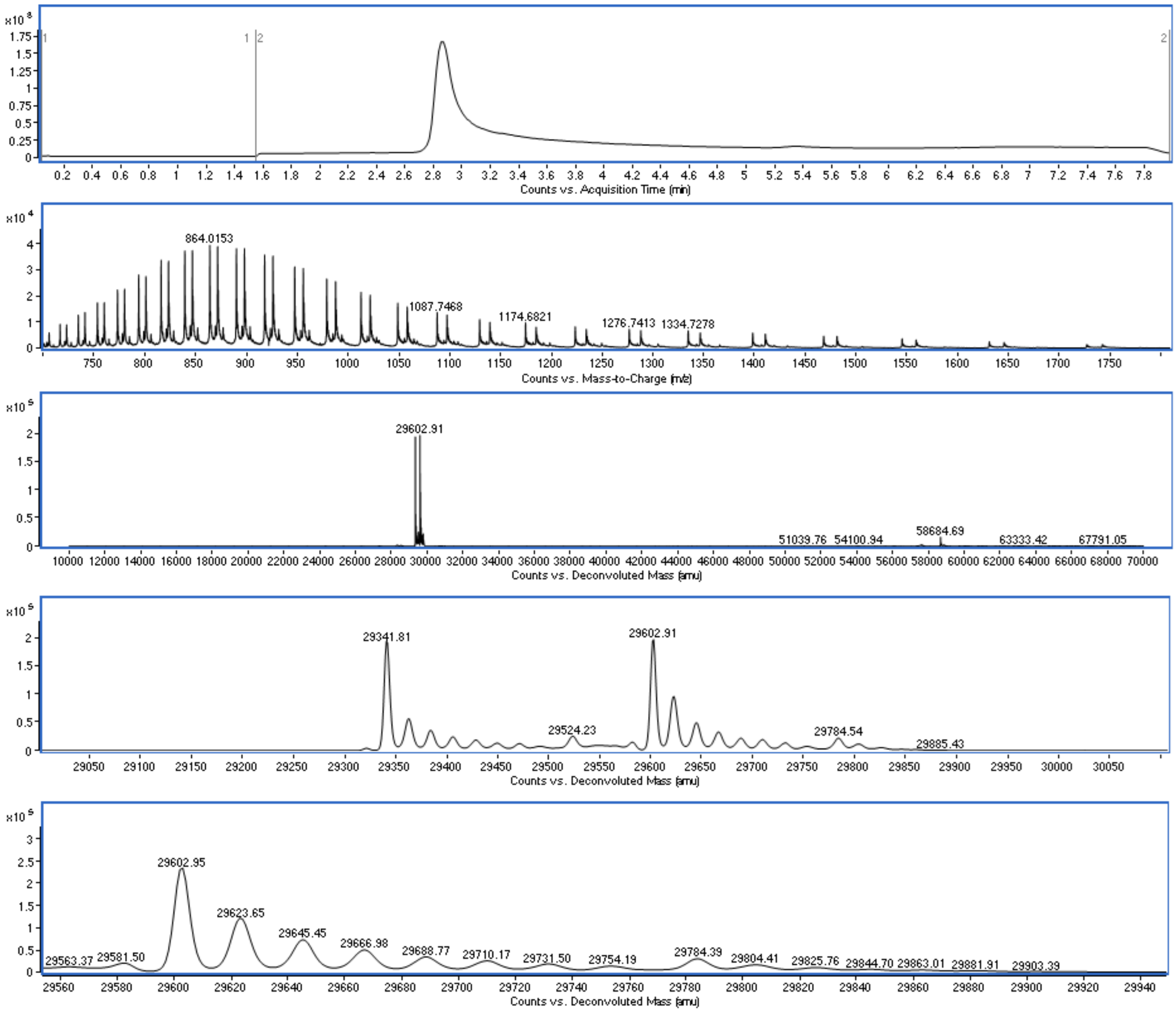

pH 6.0

Observed masses: 29,342; 29,603 Da

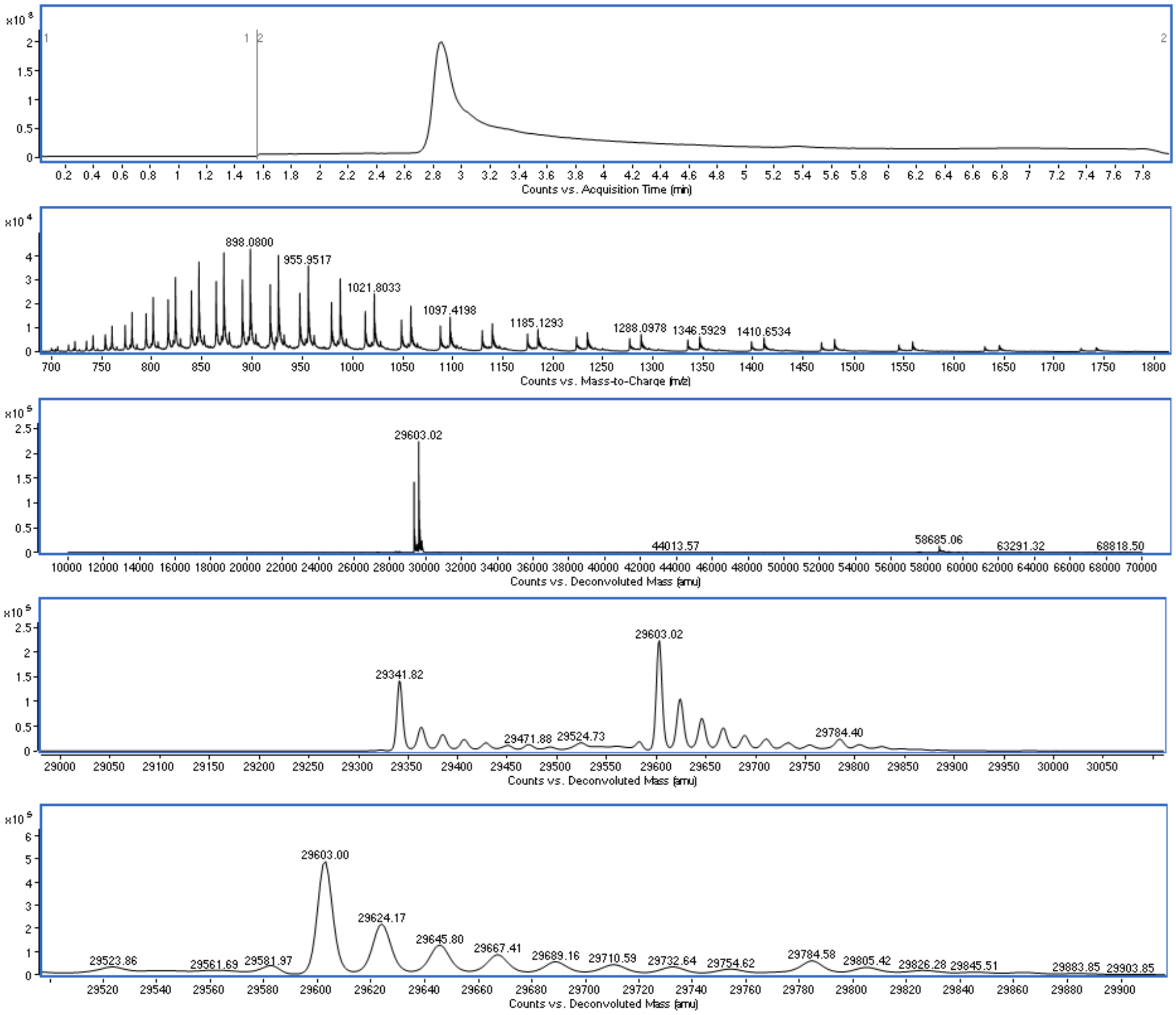

- Timepoint day 2

pH 9.0

Observed masses: 29,342; 29,603; 29,622 Da

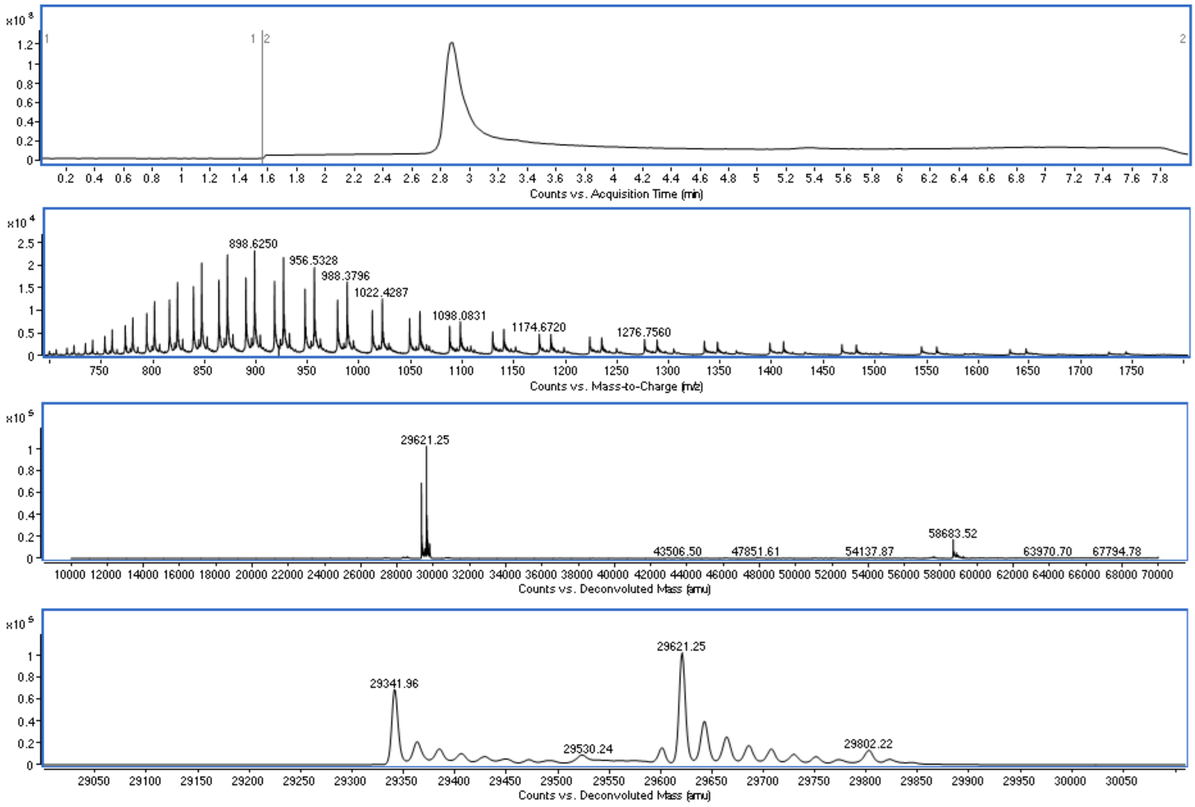

pH 7.4

Observed masses: 29,342; 29,603; 29,622 Da

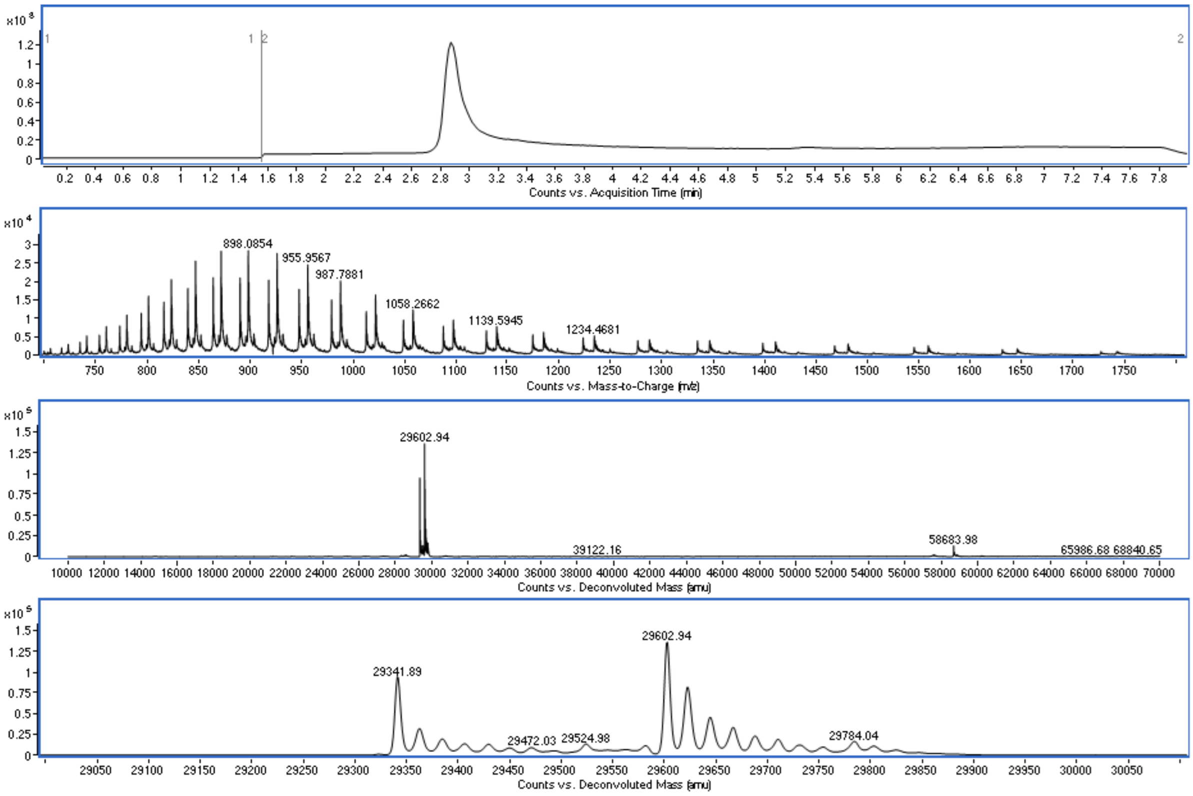

pH 6.0

Observed masses: 29,342; 29,603 Da

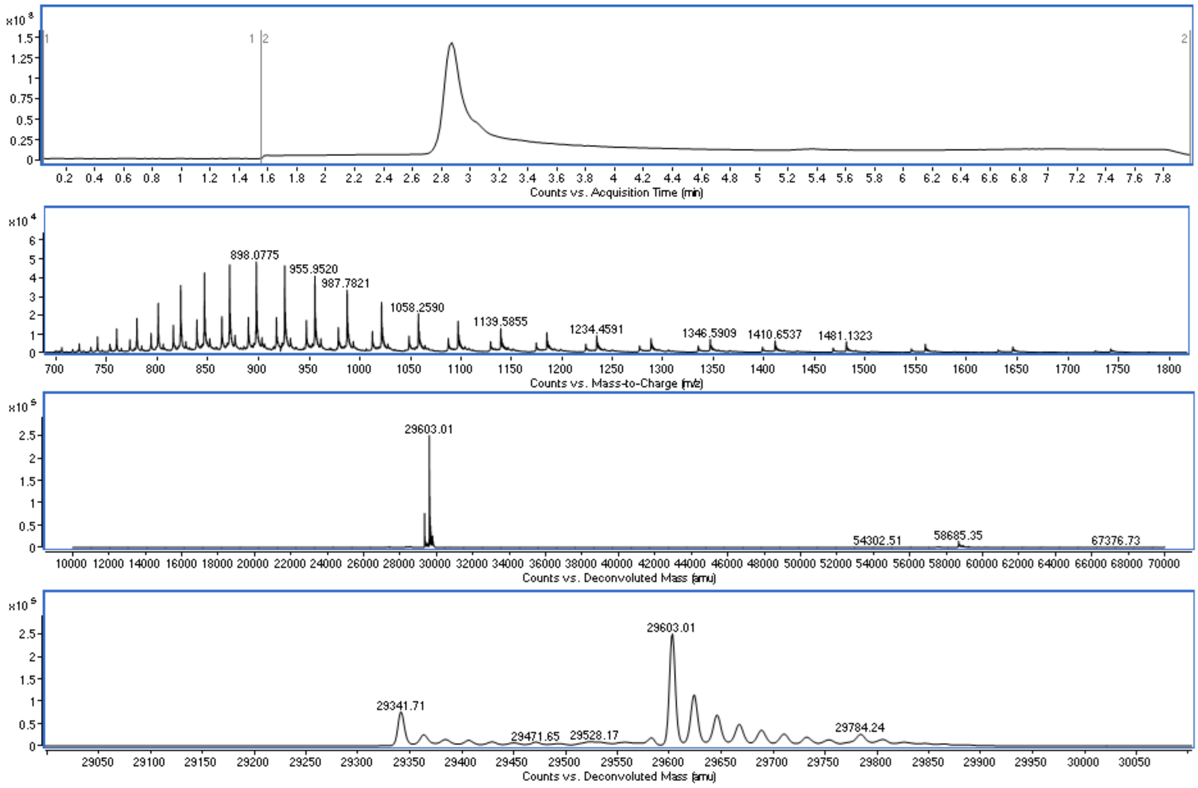

- Timepoint day 3

pH 9.0

Observed masses: 29,342; 29,622; 58,685 Da

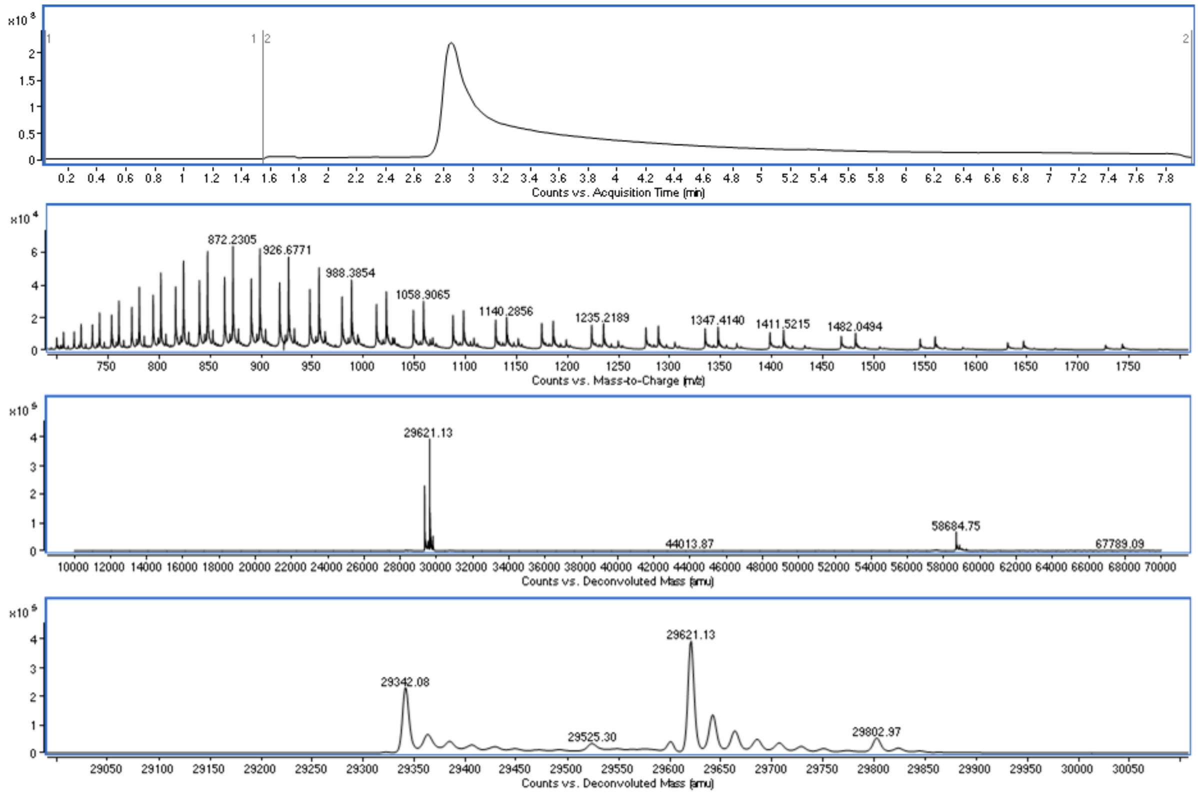

pH 7.4

Observed masses: 29,342; 29,603; 29,622; 58,684 Da

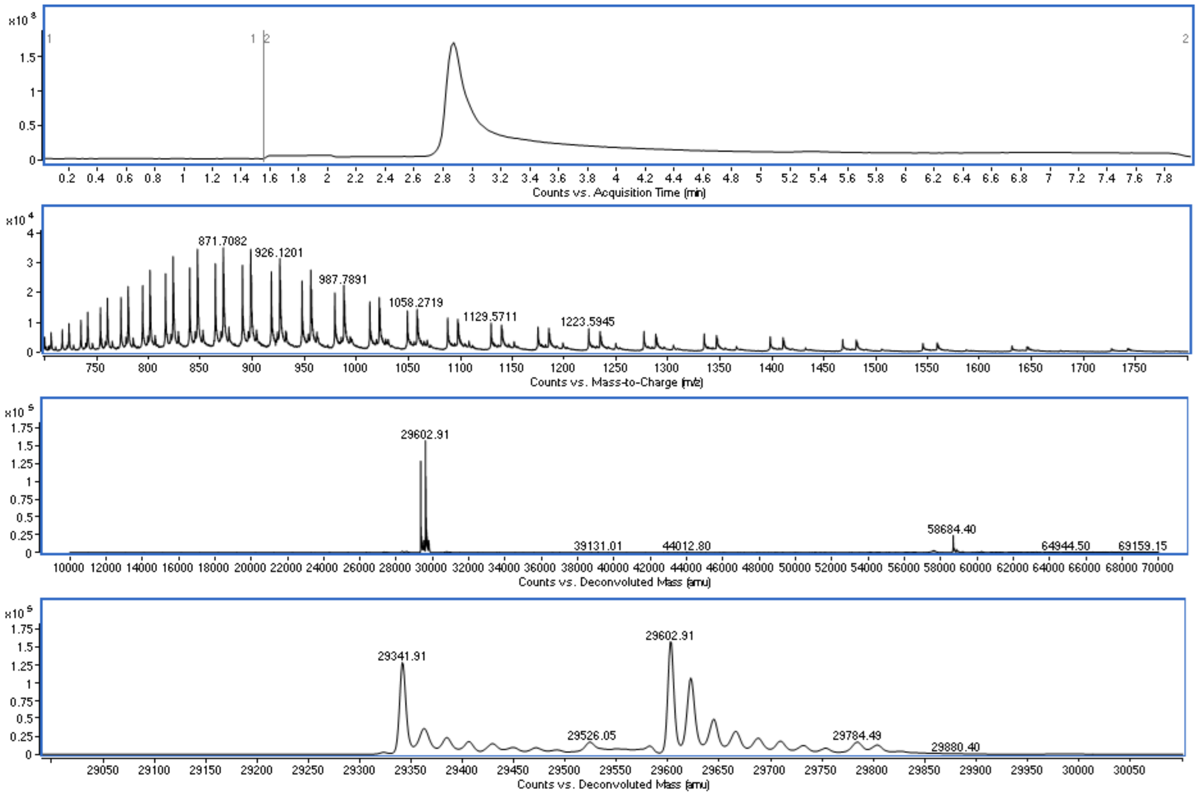

pH 6.0

Observed masses: 29,342; 29,603

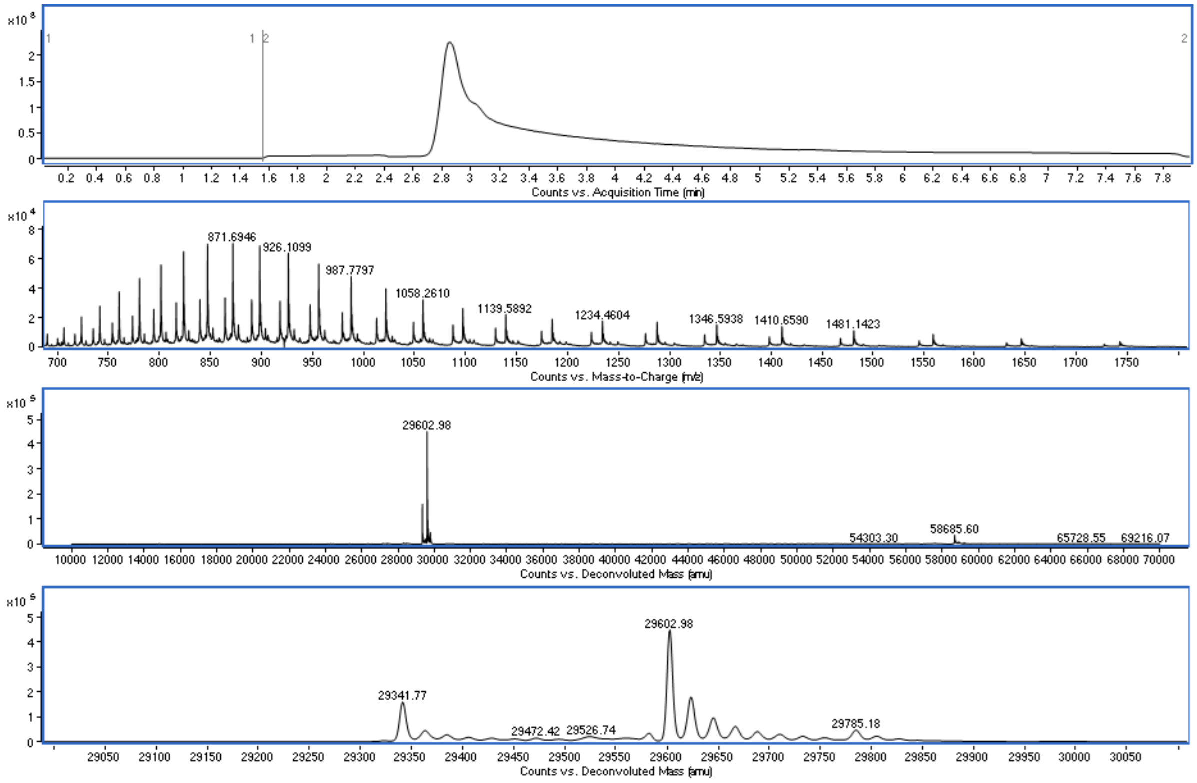

- Timepoint day 5

pH 9.0

Observed masses: 29,342; 29,622; 58,684 Da

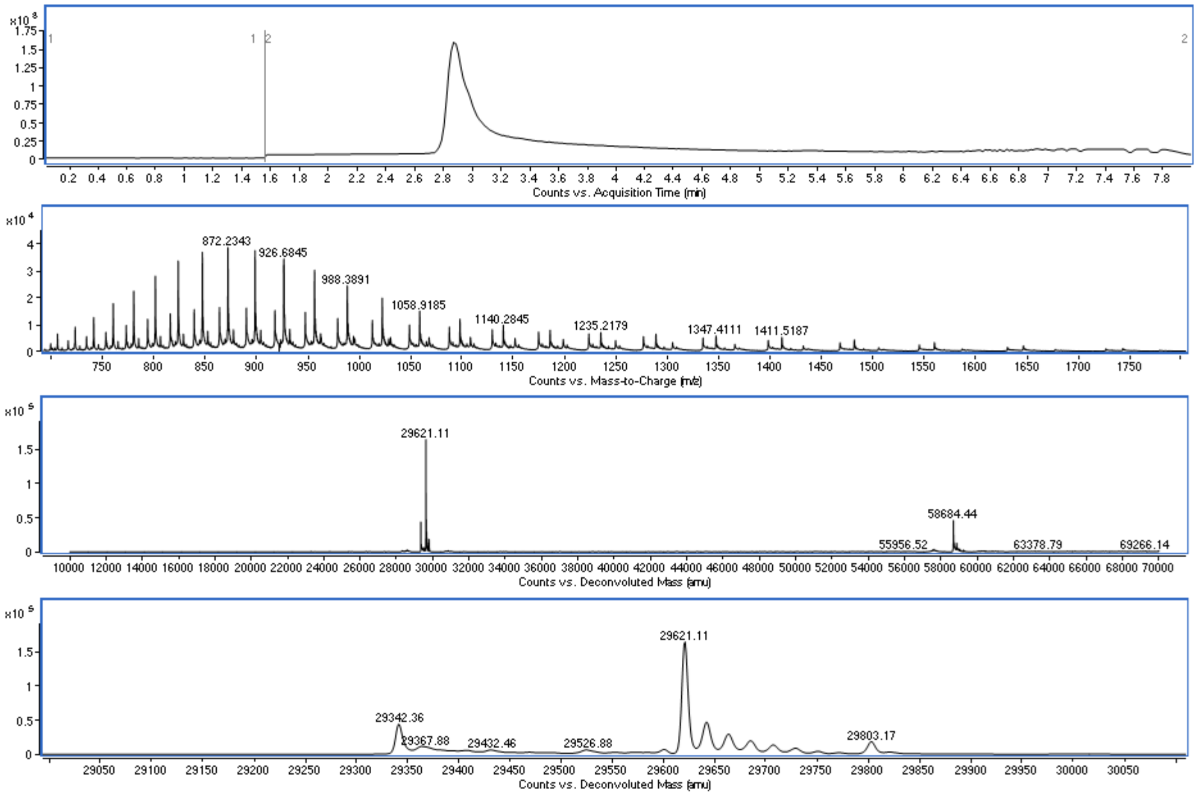

pH 7.4

Observed masses: 29,342; 29,603; 29,622; 58,685 Da

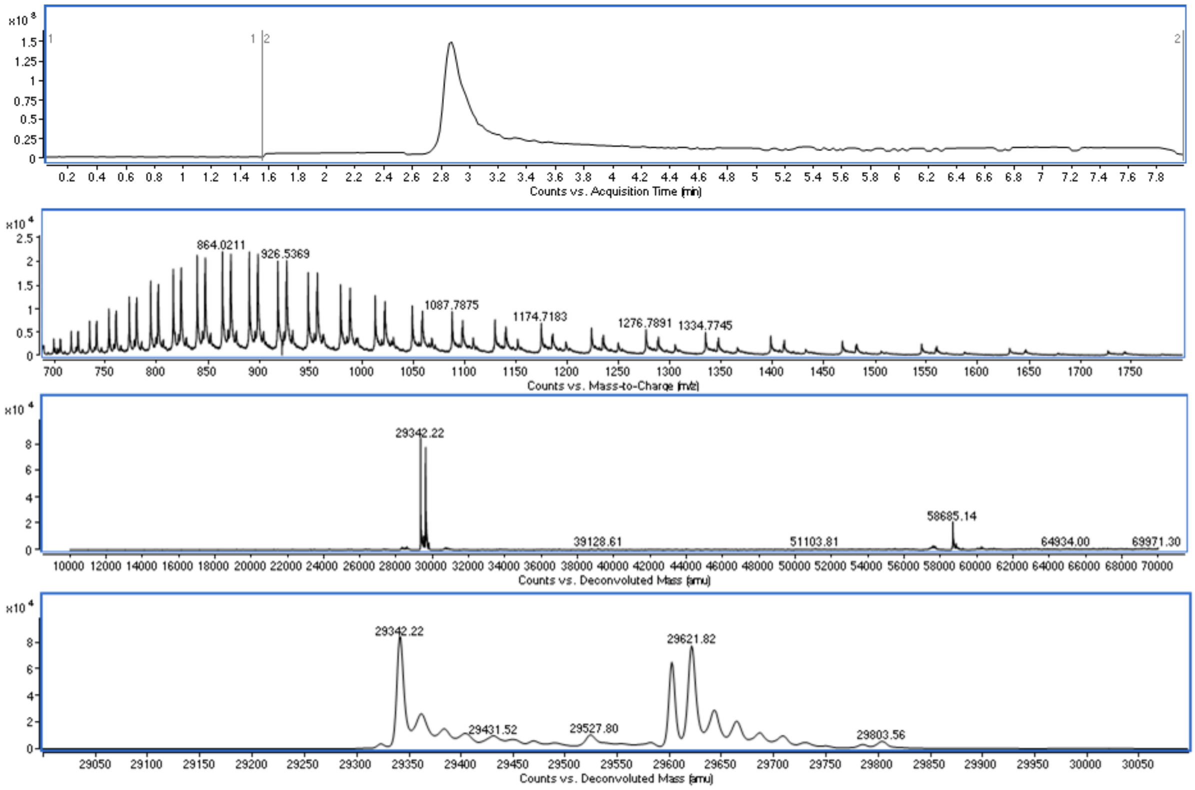

pH 6.0

Observed masses: 29,342; 29,603; 58,683 Da

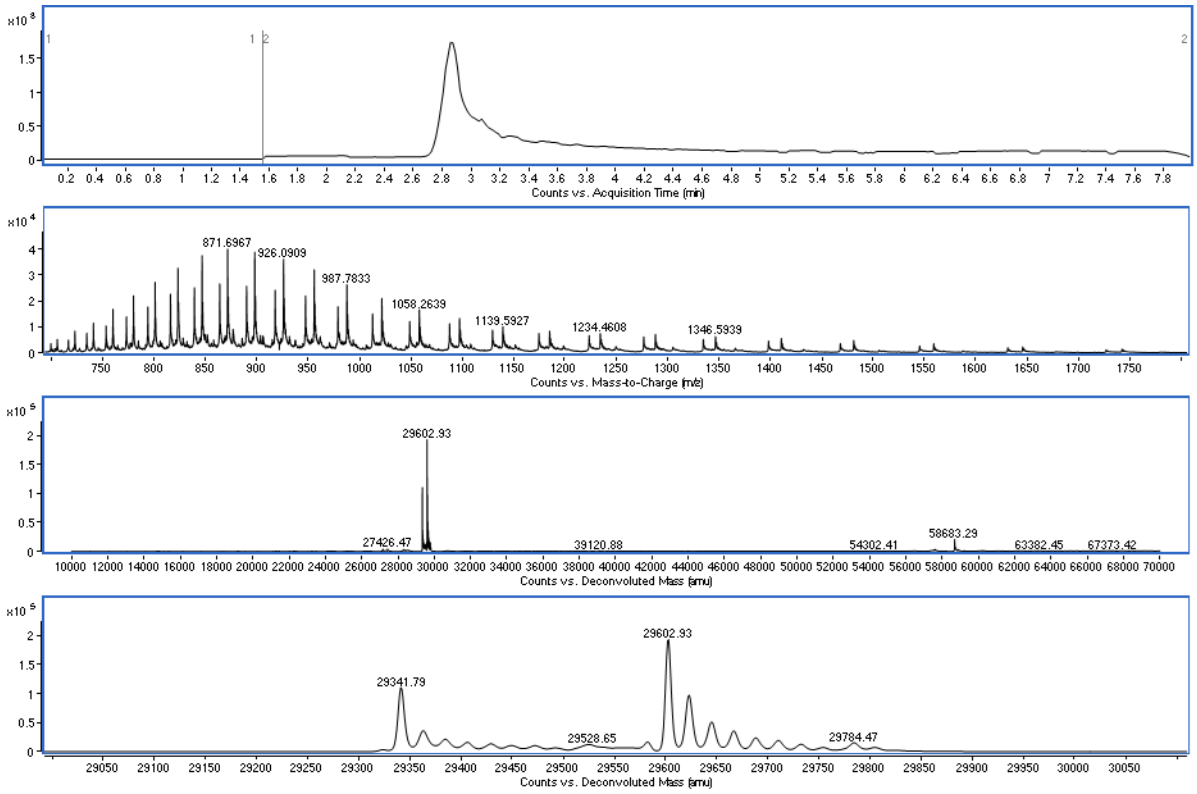

- Timepoint day 9

pH 9.0

Observed masses: 29,622; 58,683 Da

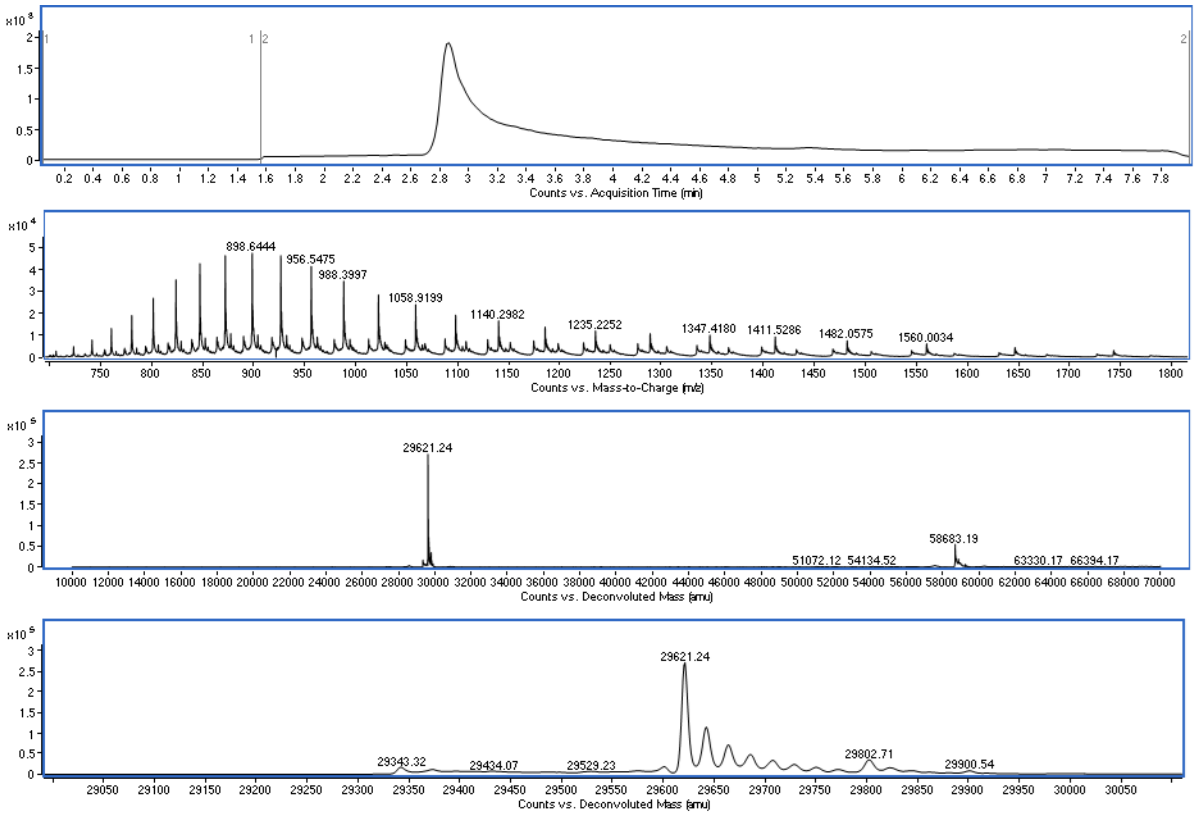

pH 7.4

Observed masses: 29,343; 29,603; 29,622; 58,691 Da

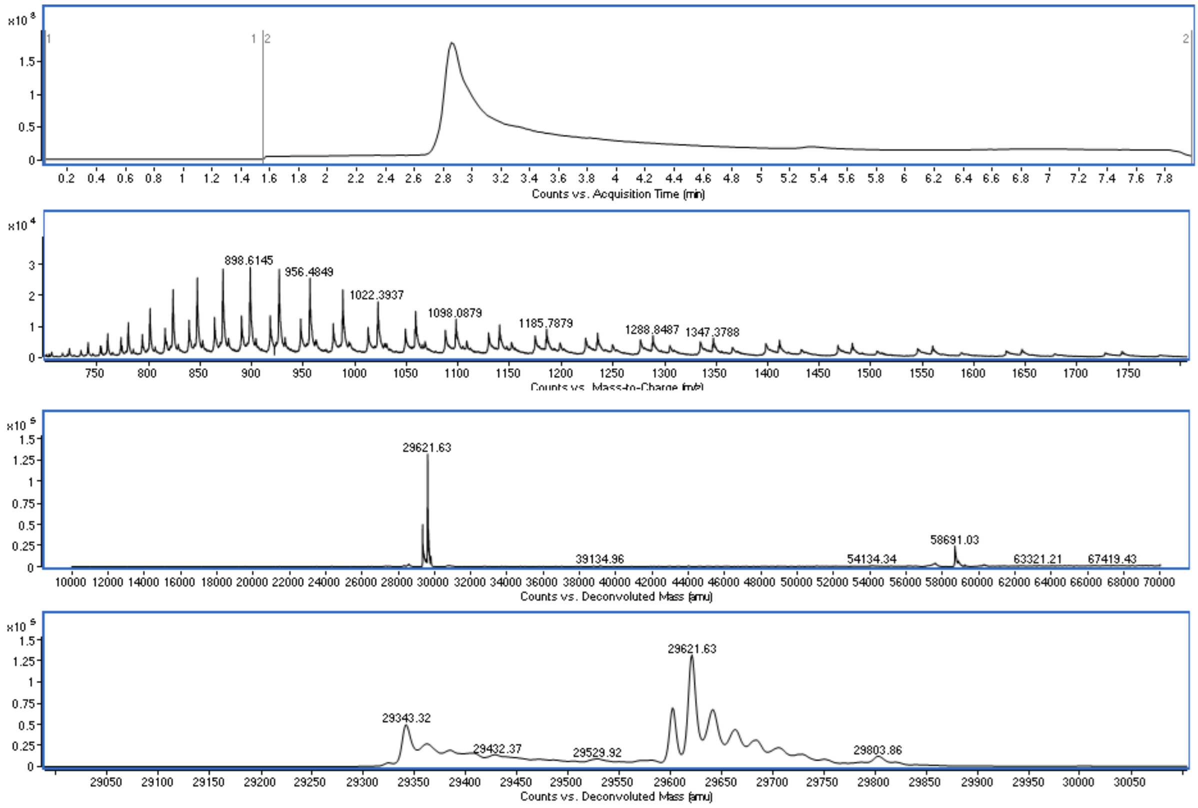

pH 6.0

Observed masses: 29,603; 58,682 Da

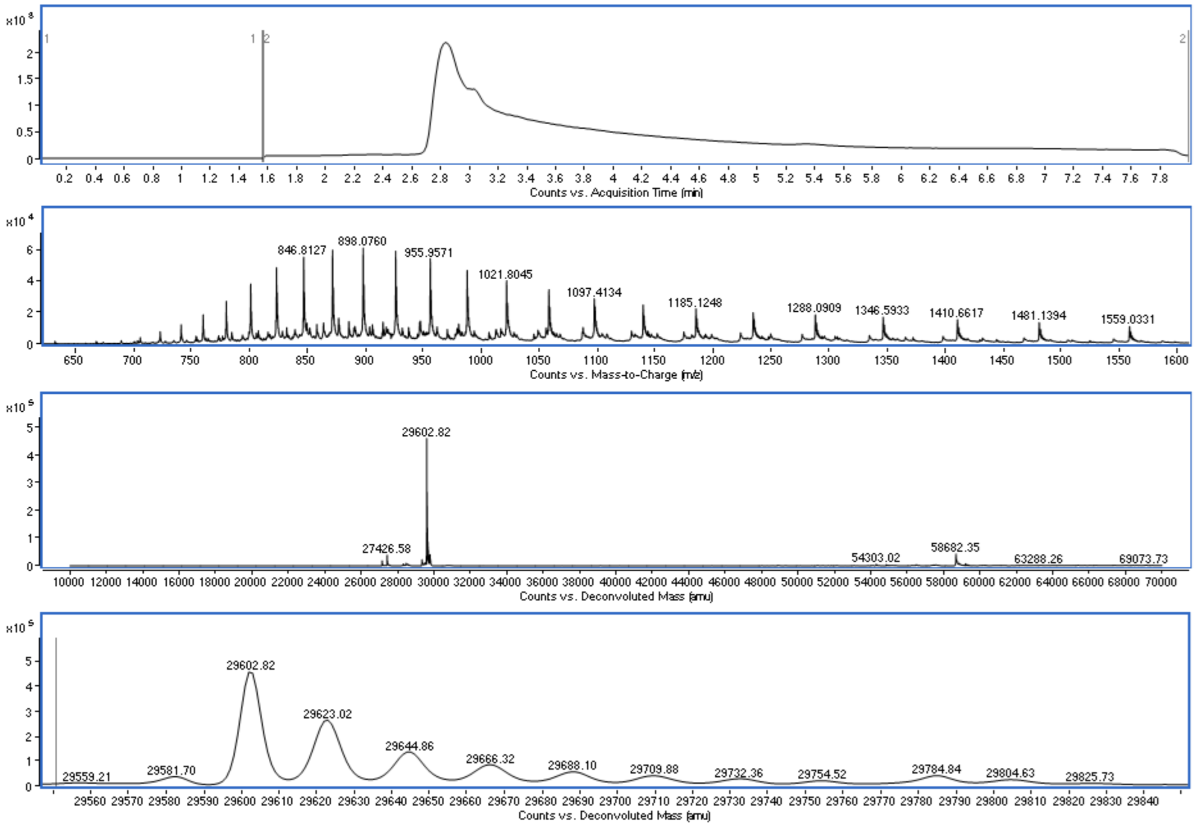

- *Summary*

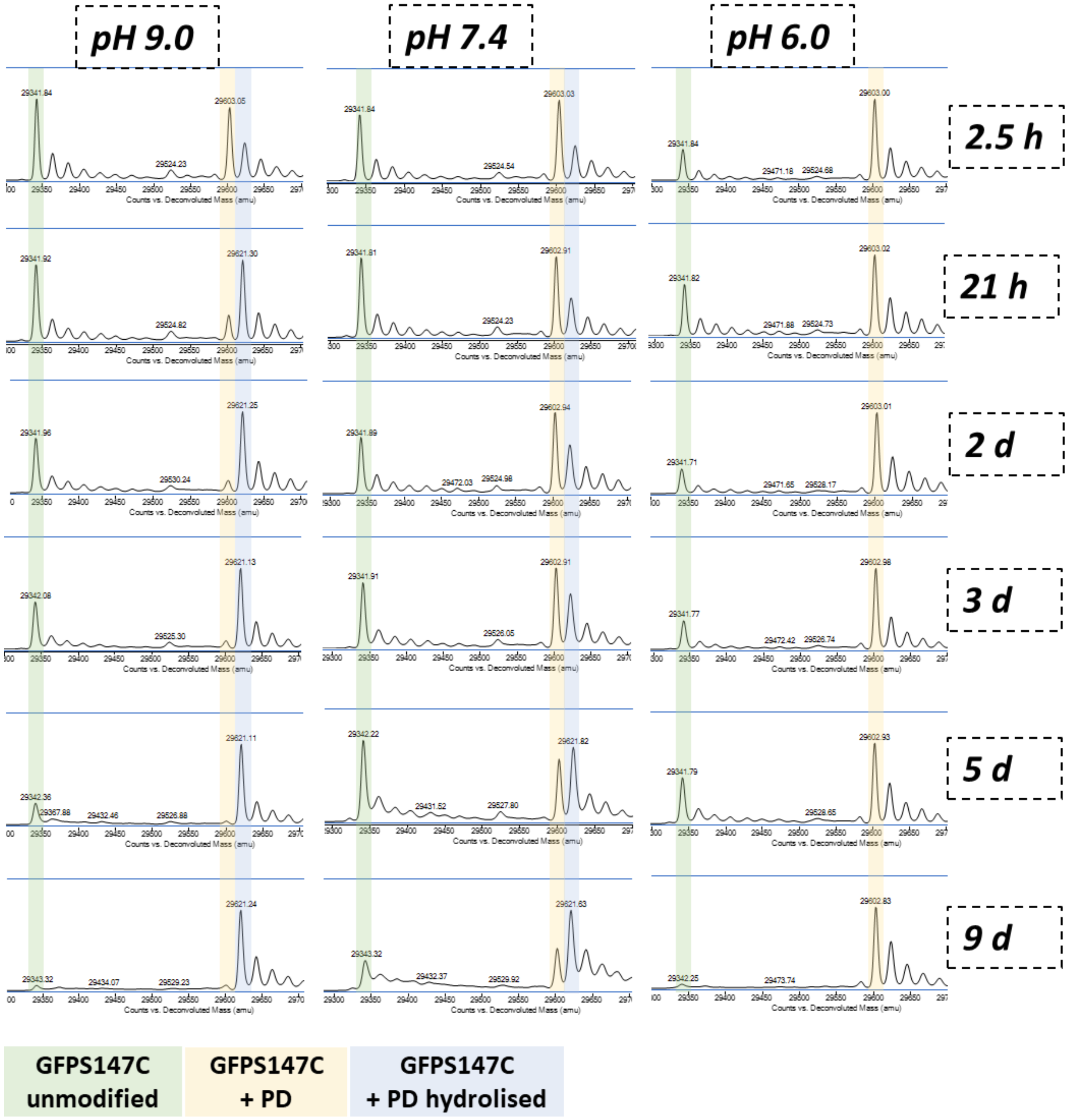

*Figure S5. Summary of stability study of GFPS147C-PD conjugate **38** overtime under different pHs tested.*

Please note that GFPS147C dimer **S10** formation was happening slowly throughout the course of the study, especially as time increased, which is why the GFPS147C peak spikes and then decreases over time.

4.1.5 *Dynamic study*

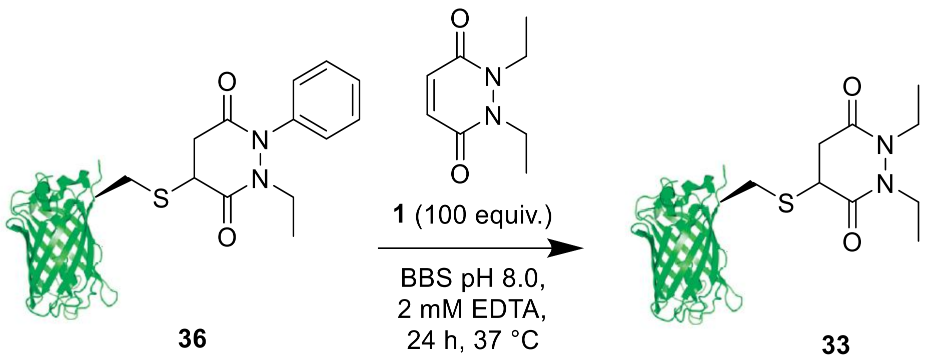

To a solution of GFPS147C-PD conjugate **36** (40 µL, 35 µM in BBS EDTA pH 8.0, 0.0014 µmol) was added PD **1** (2.8 µL, 50 mM in MeCN, 100 equiv., 0.14 µmol) and the reaction was incubated in BBS 2 mM EDTA pH 8.0, at 37 °C for 24 h. LC-MS analyses were taken at t = 0 h, 6 h and 24 h.

Expected masses: 29,557 Da (GFPS147C-PD **36**); 29,509 Da (GFPS147C + PD **33**).

- *Timepoint t = 0 h*

Observed masses: 29,558 Da.

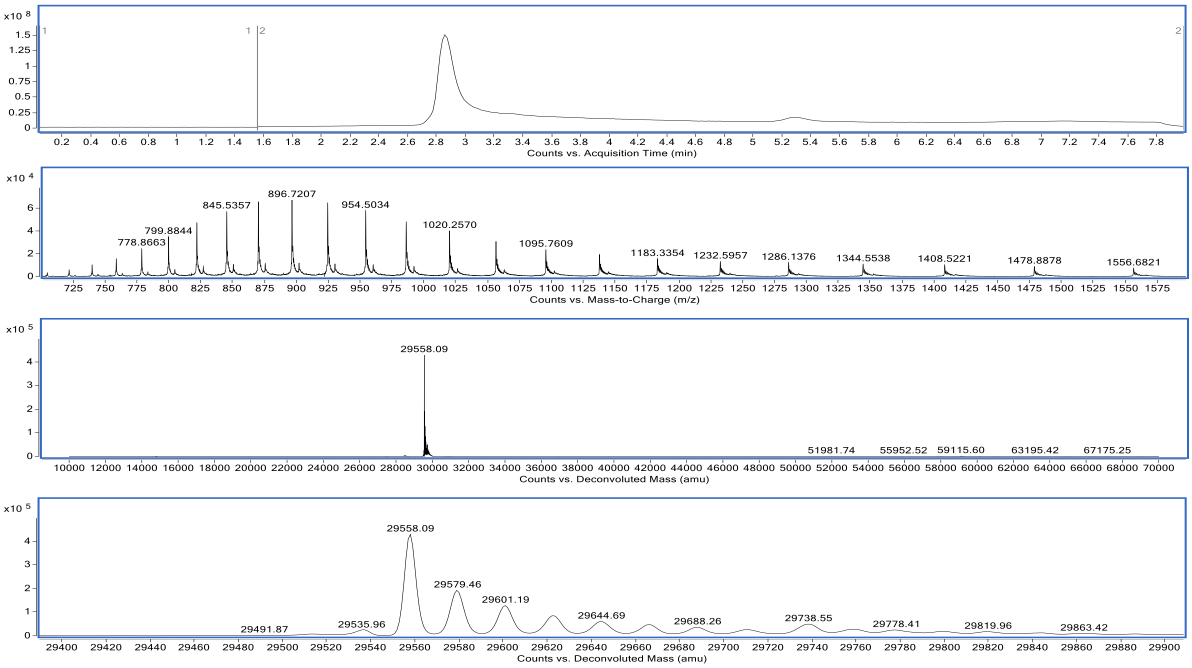

- *Timepoint t = 6 h*

Observed masses: 29,510 Da; 29,557 Da.

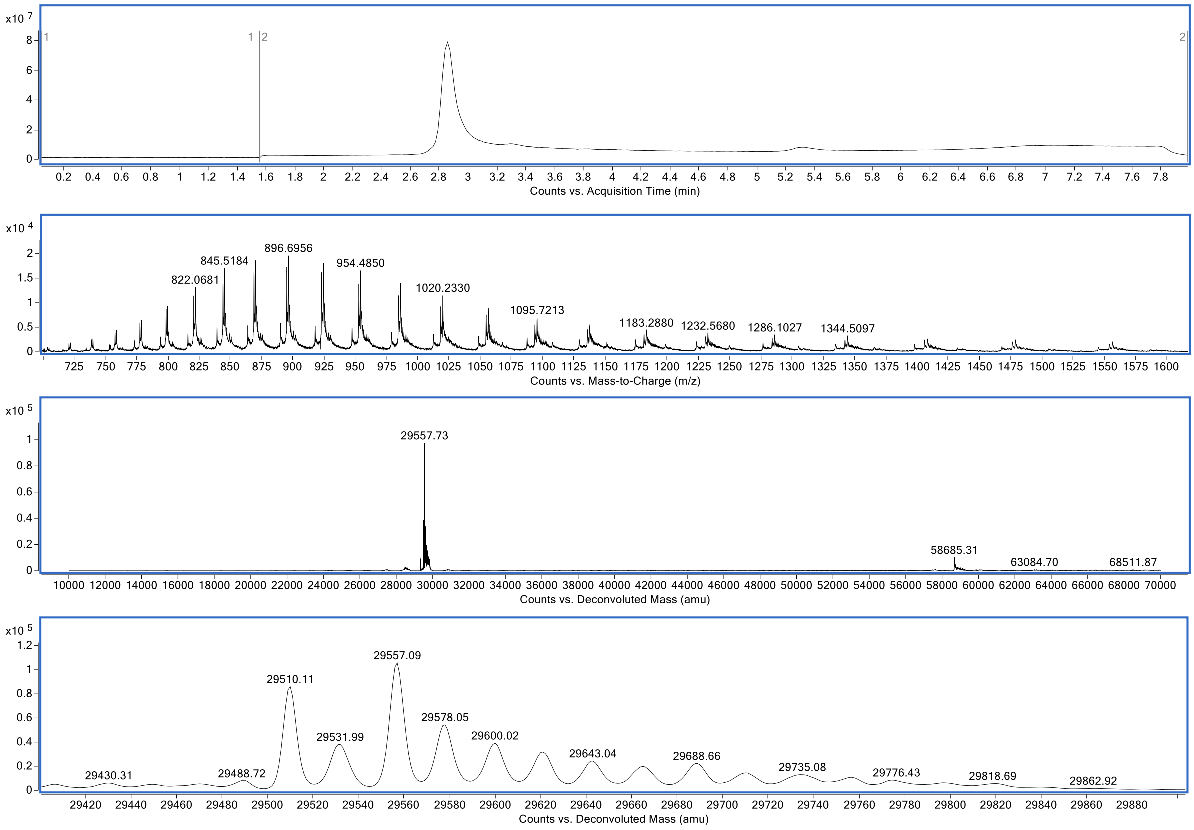

- *Timepoint t = 24 h*

Observed masses: 29,510; 58,683 Da.

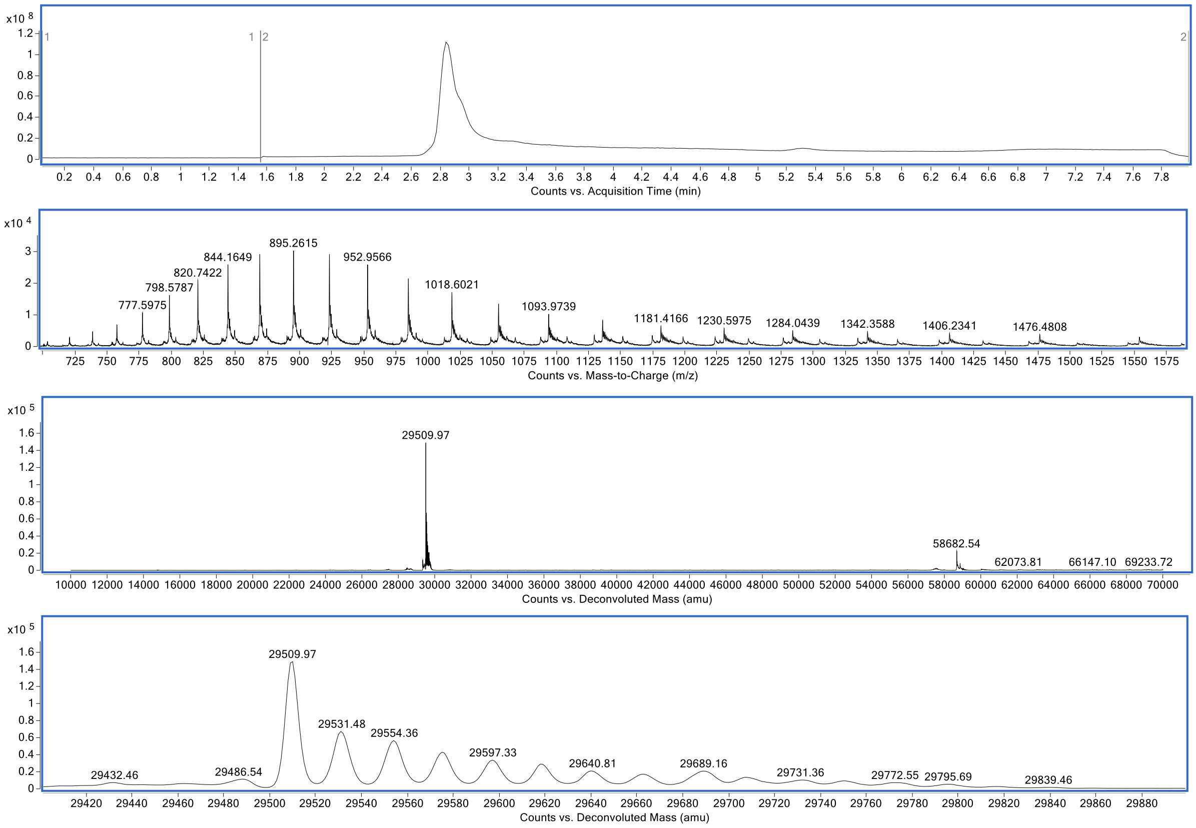

4.1.6 Control: stability study of GFPS147C-maleimide conjugates

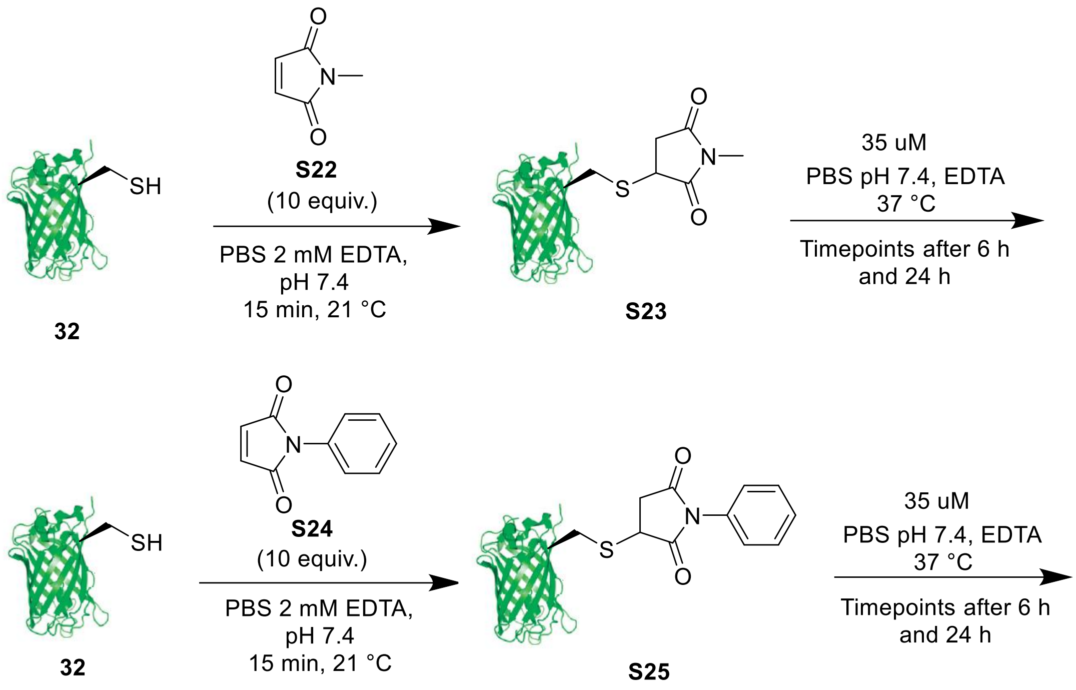

To a solution of reduced GFPS147C **32** (80 µL, 60 µM in BBS EDTA, 0.0048 µmol) was added maleimide derivative **S22** or **S24** (2.4 µL, 20 mM in DMSO, 10 equiv.) and the reaction was incubated at 21 °C for 15 min. After this time, the volume was adjusted to 100 µL and excess of small molecules was removed using Zeba™ Spin Desalting column in LCMS grade water. LCMS analysis was carried out quickly after removal of the small molecules (within 10 min) to avoid deconjugation of the maleimide from the GFPS147C-maleimide conjugate during analysis. The complete removal of the excess of small molecule was carried out by using 2 × Zeba™ Spin Desalting column in PBS pH 7.4, 2 mM EDTA. The concentration of the conjugates **S23** and **S25** was adjusted to 35 µM and the solution left to incubate under constant agitation (300 rpm) at 37 °C. LC-MS analysis was taken after 6 h and 24 h. LCMS analysis was carried out quickly after removal of the small molecules (within 10 min) to avoid deconjugation of the maleimide from the GFPS147C-maleimide conjugate during analysis.

Expected mass upon deconjugation: 29,342 Da (GFPS147C **32**).

*GFP S147C-NMM **S23**, timepoint t = 0*

Expected mass: 29,453 Da (**S23)**. Observed mass: 29,453 Da.

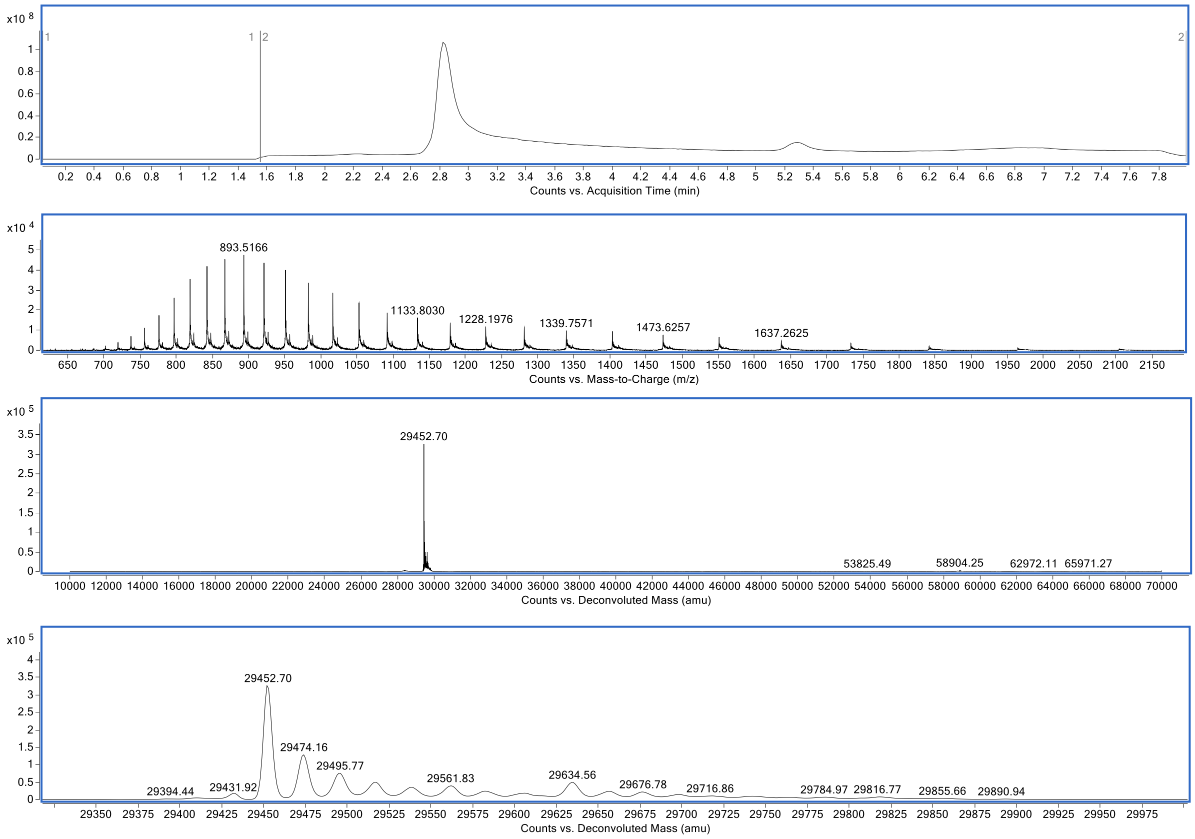

*GFP S147C-NMM **S23**, timepoint t = 6 h*

Observed mass: 29,453 Da.

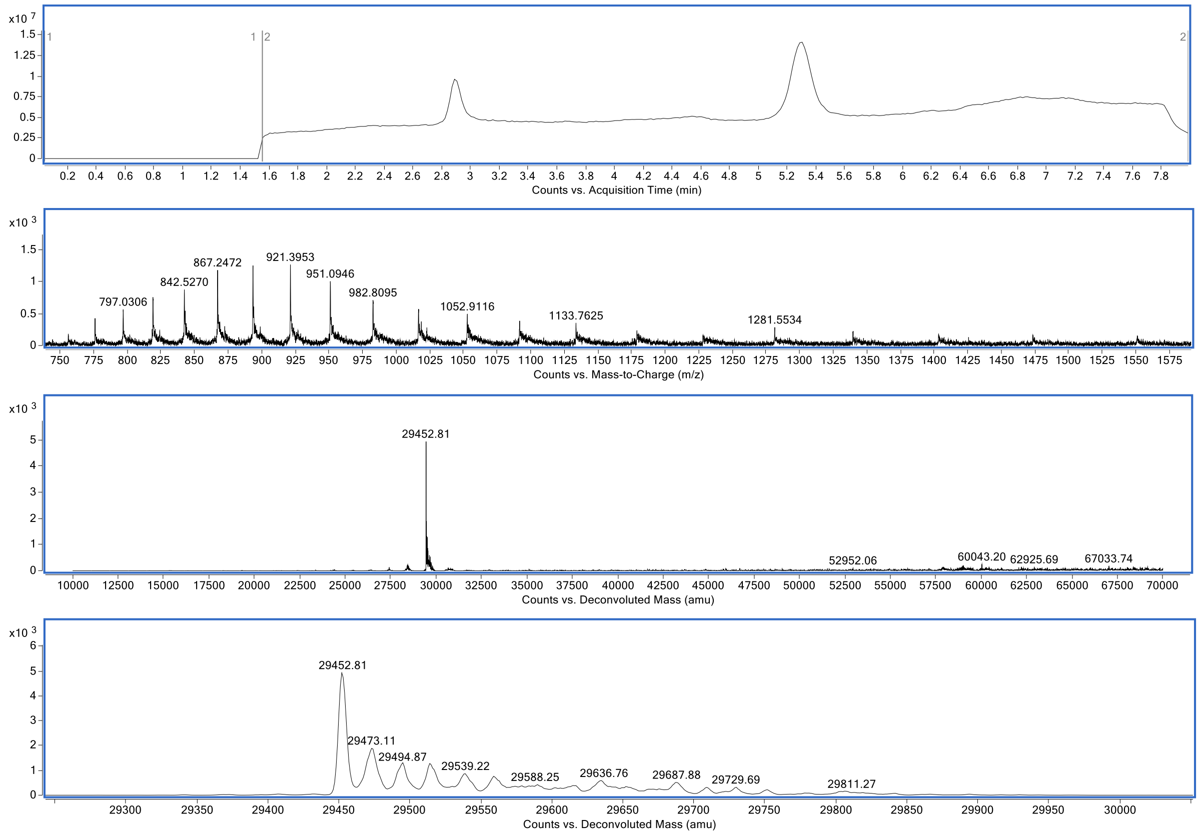

*GFP S147C-NMM **S23**, timepoint t = 24 h*

Observed masses: 29,453 Da; 29,472 Da.

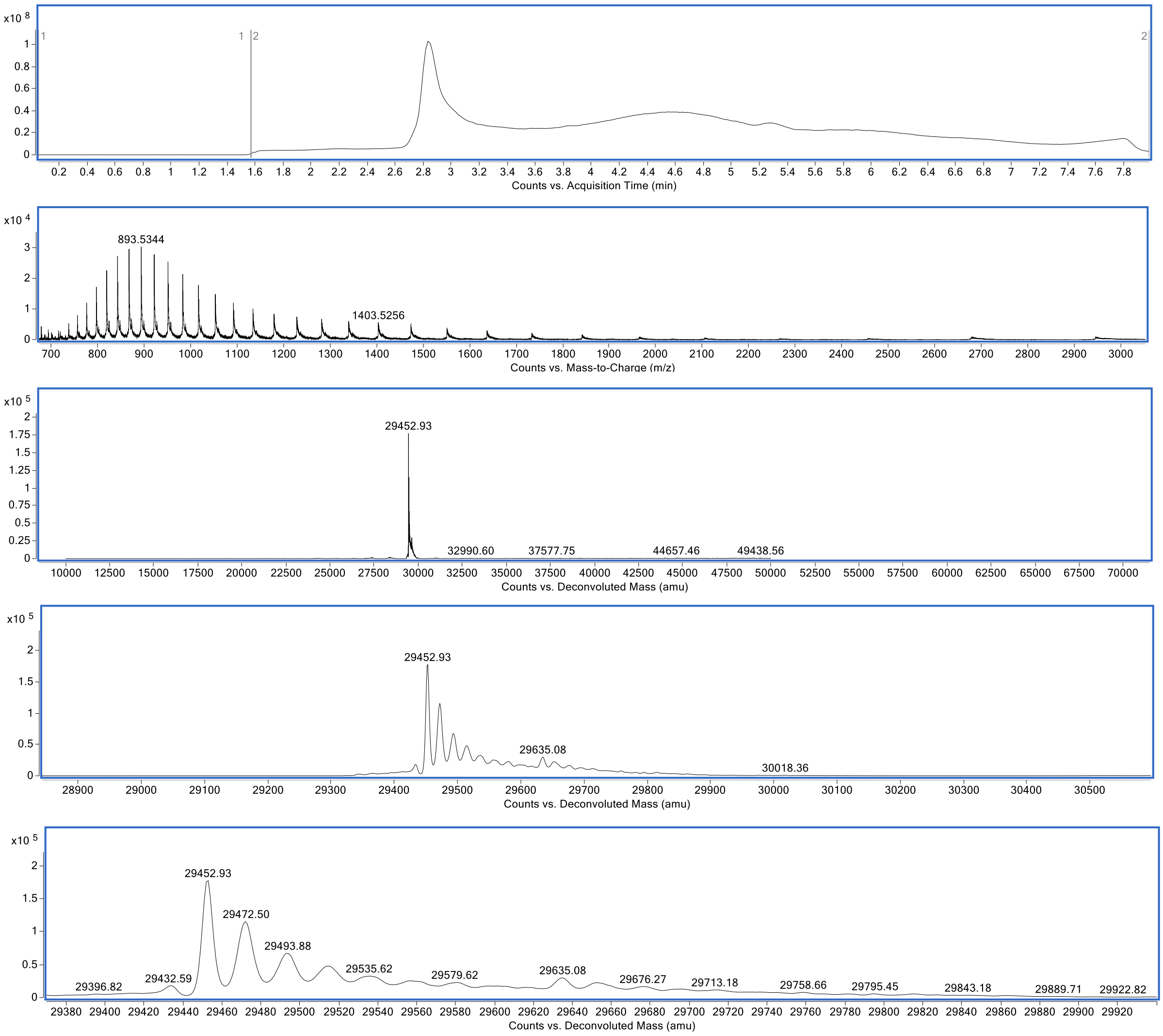

*GFPS147C-NPM **S25**, timepoint t = 0*

Expected mass: 29,515 Da (**S25**). Observed mass: 29,515 Da.

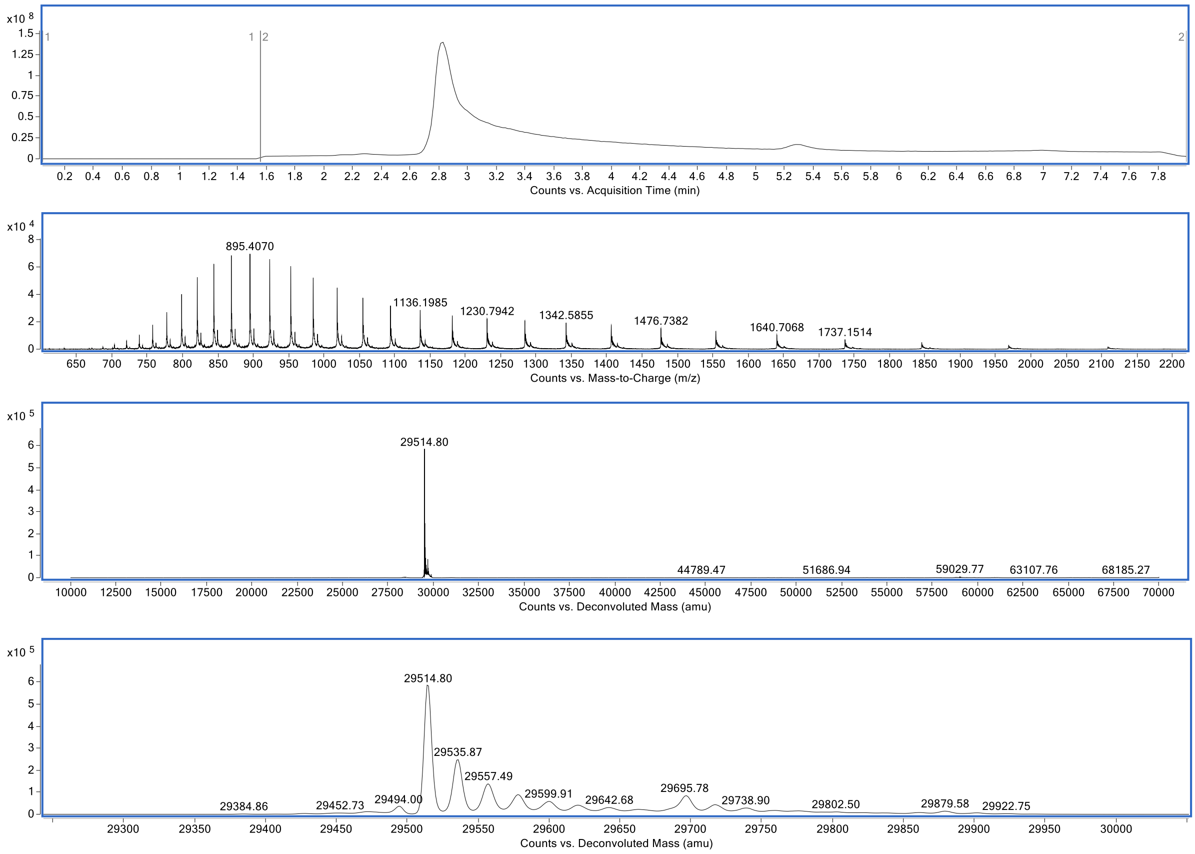

GFP S147C-NPM ***S25****, timepoint t = 6 h*

Observed masses: 29,515 Da; 29,533 Da.

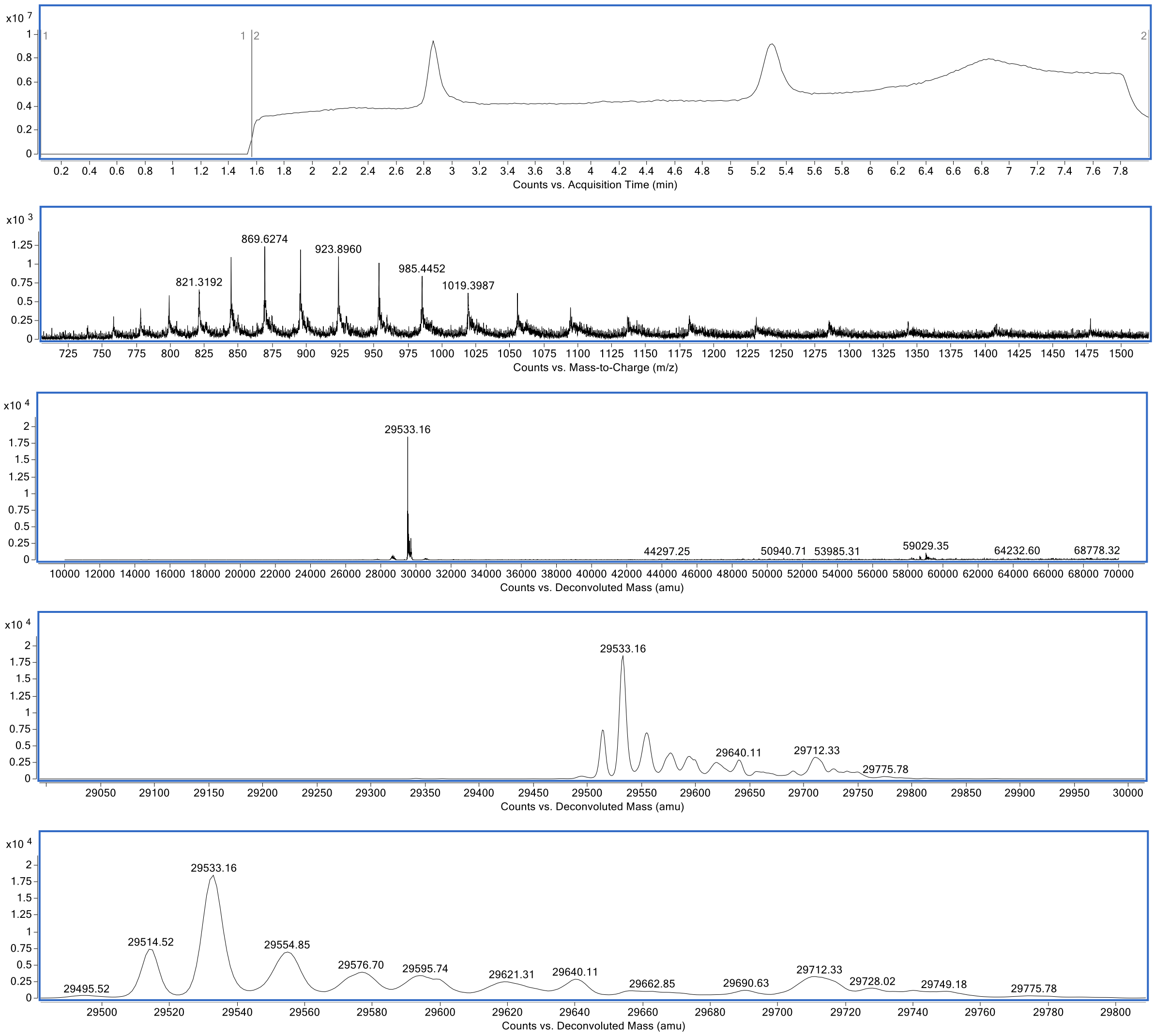

GFP S147C-NPM ***S25****, timepoint t = 24 h*

Observed mass: 29,533 Da.

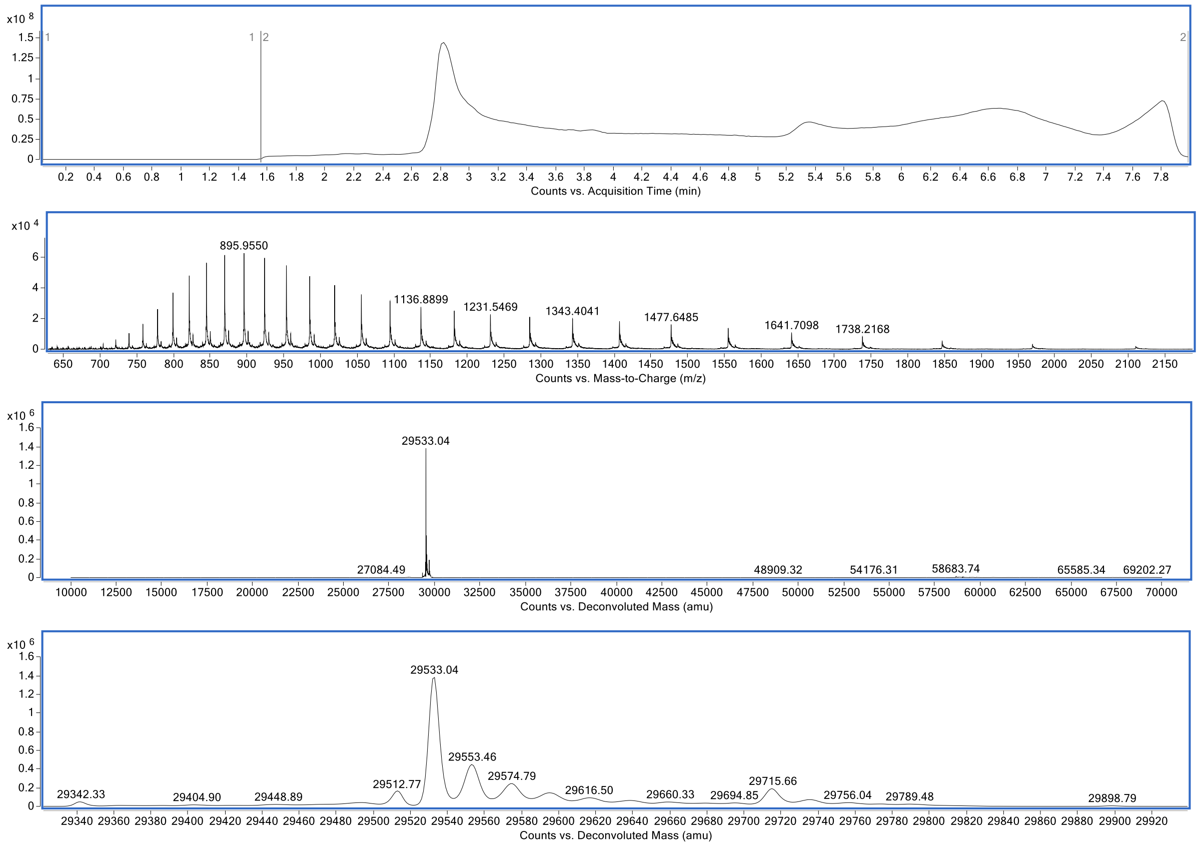

SDS-PAGE gel

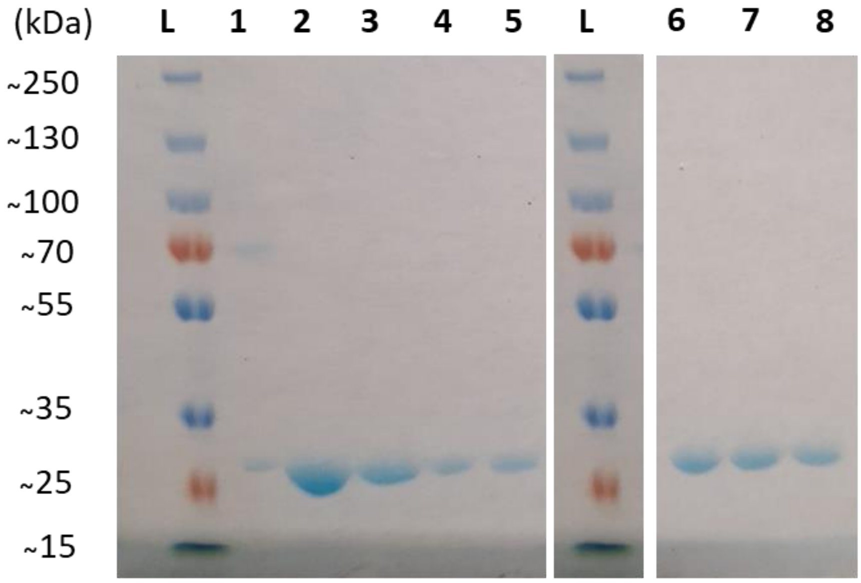

*Figure S6. SDS-PAGE gel of GFPS147C-NMM **S23** and GFPS47C-NPM **S25** after incubation in PBS pH 7.4 at 37 °C for 6 h and 24 h. **Lane L**: ladder; **Lane 1**: GFPS147C non reduced; **Lane 2**: GFPS147C reduced **32**; **Lane 3**: GFPS147C-NMM **S23** t = 0 h; **Lane 4:** GFPS147C-NMM **S23** t = 6 h; **Lane 5**: GFPS147C-NMM **S23** t = 24 h; **Lane 6**: GFPS147C-NPM **S25** t = 0 h; **Lane 7**: GFPS147C-NPM **S25** t = 6 h; **Lane 8**: GFPS147C-NPM **S25** t = 24 h*

4.2 Fab experiments

4.2.1 Trastuzumab Fab

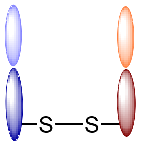

22

Trastuzumab Fab **22** was obtained through pepsin/papain digestion of Trastuzumab as described previously.[14] Expected mass: 47,639 Da. Observed mass: 47,639 Da (tailing due to Na^+ adducts).

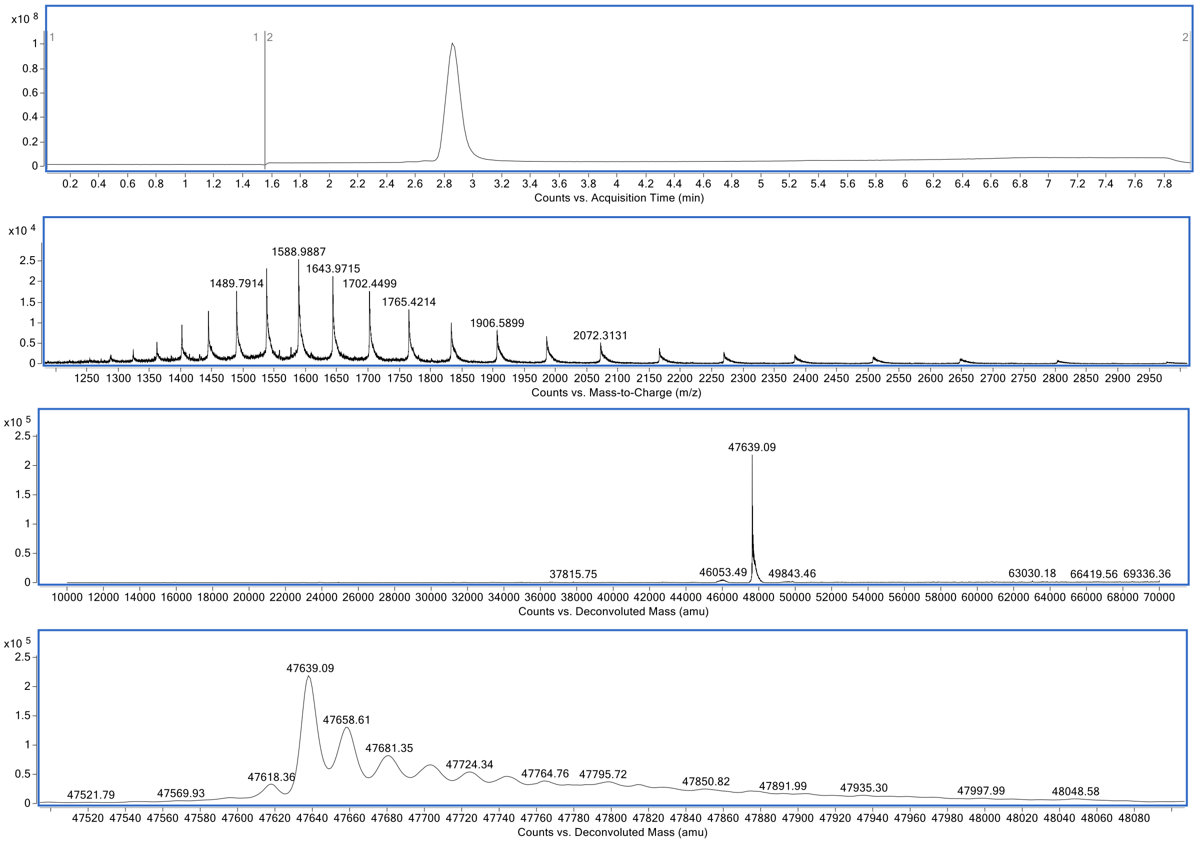

4.2.2 Release study on Fab Trastuzumab

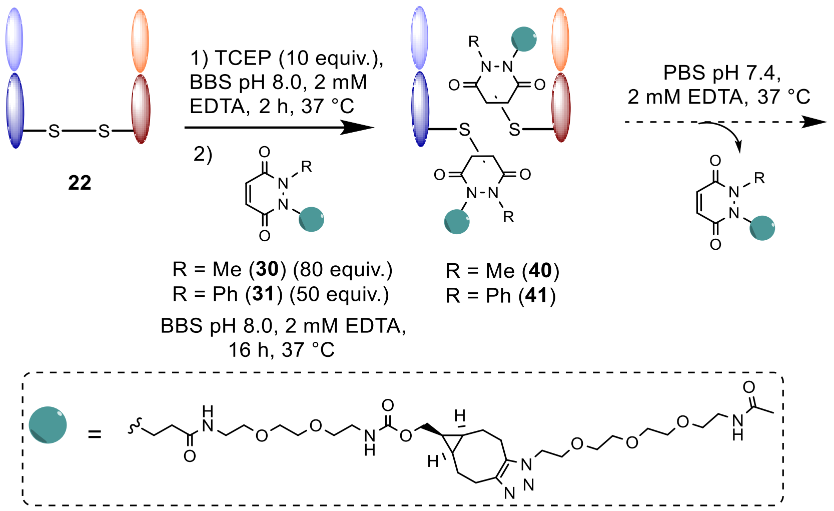

To a solution of Trastuzumab Fab **22** (60 µM, 100 µL) in BBS (25 mM sodium borate, 25 mM NaCl, 2 mM EDTA pH 8.0) was added TCEP·HCl (3 µL, 20 mM in d.d. H_2O, 10 equiv.). The mixture was incubated for 120 min at 37 °C under constant agitation (300 rpm). The excess of TCEP was removed by buffer-exchange in BBS (25 mM sodium borate, 25 mM NaCl, 2 mM EDTA, pH 8.0) using Zeba™ Spin Desalting column. PD-BCN **30** or **31** (3 µL, 20 mM in MeCN, 50 equiv. for **30** and 4.8 µL, 20 mM in MeCN, 80 equiv. for **31**) was then added and both reactions were incubated at 37 °C for 16 h. The excess of small molecules was removed by buffer-exchange in LCMS grade water using Zeba™ Spin Desalting column for LCMS analysis. 2 supplementary Zeba™ Spin Desalting columns were used to remove all the excess small molecules and buffer-swapped in PBS pH 7.4. The concentration of the Fab conjugates **40** and **41** were adjusted to 35 µM. Deconjugation of **30** and **31** was followed by LCMS by taking timepoints after 2.5 h, 6.5 h, 24 h, 48 h and 72 h. LCMS analysis was carried out quickly after removal of the small molecules (within 10 min) to avoid deconjugation of the PD from the Fab-PD conjugate during analysis.

Expected masses after deconjugation: 23,439 Da (LC), 24,202 Da (HC), 47,639 Da (Fab)

4.2.2.1. Fab conjugation

*Conjugate **40***

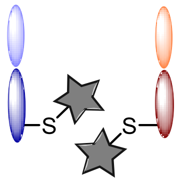

40

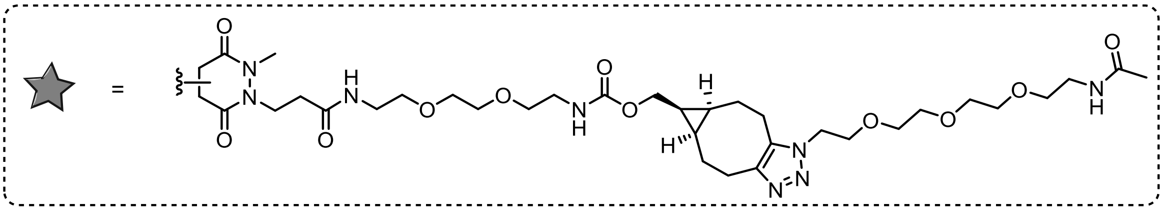

Expected masses: 24,206 Da (LC + PD); 24,967 Da (HC + PD)

Observed masses: 24,205; 24,966 Da

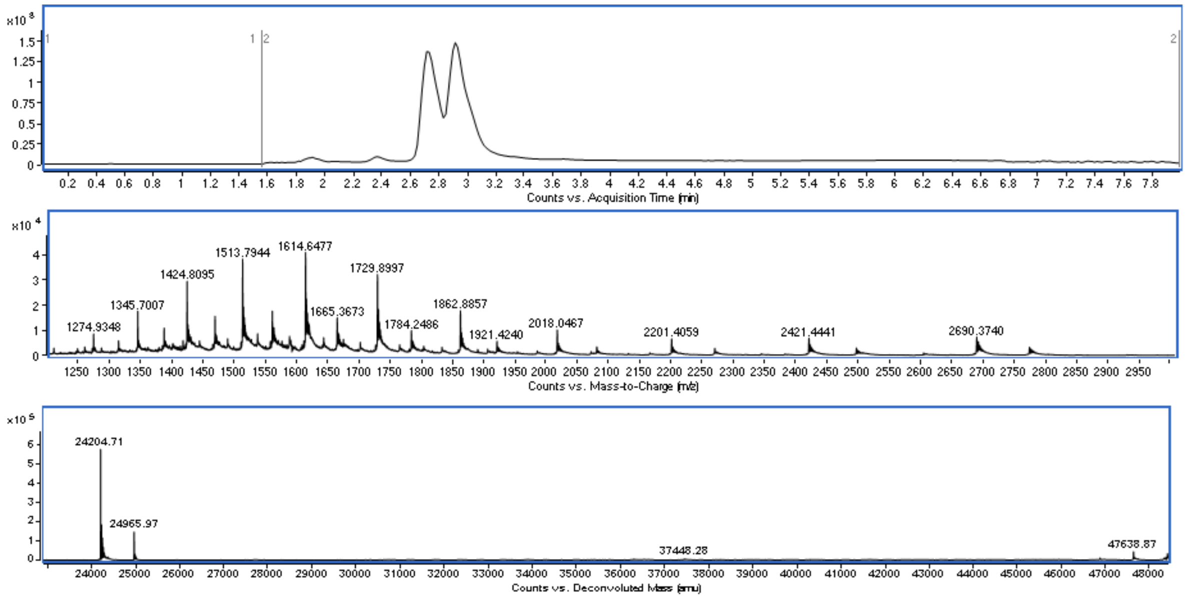

*Conjugate **41***

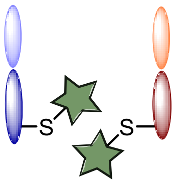

41

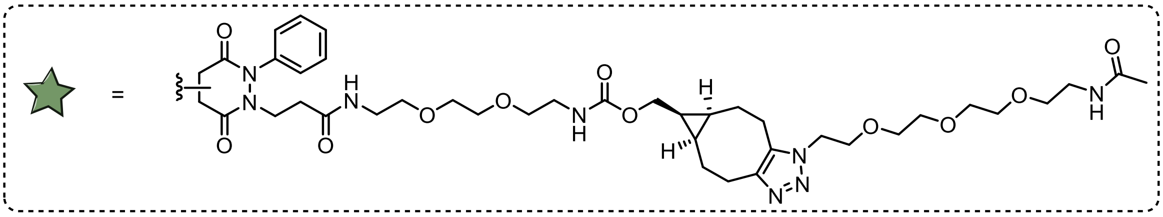

Expected masses: 24,268 Da (LC + PD), 25,029 Da (HC + PD)

Observed masses: 24,267; 25,028 Da

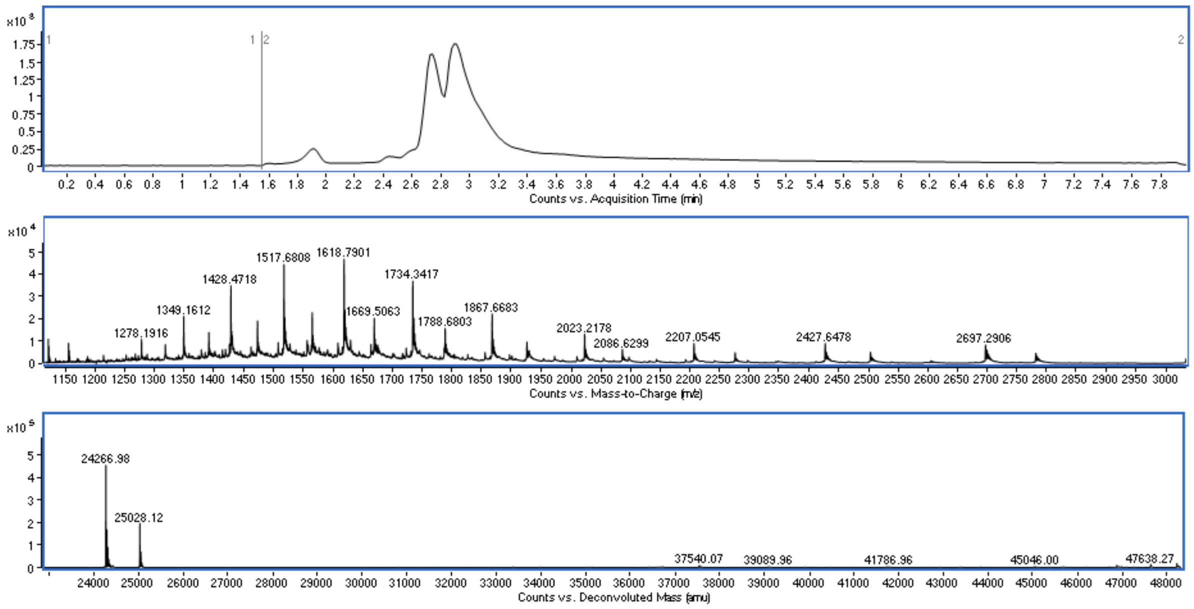

4.2.2.2. *Release study*

- *Timepoint 2.5 h*

*Conjugate **40***

Expected masses: 24,206; 24,967; 47,639 Da

Observed masses: 24,205; 24,966; 47,639 Da

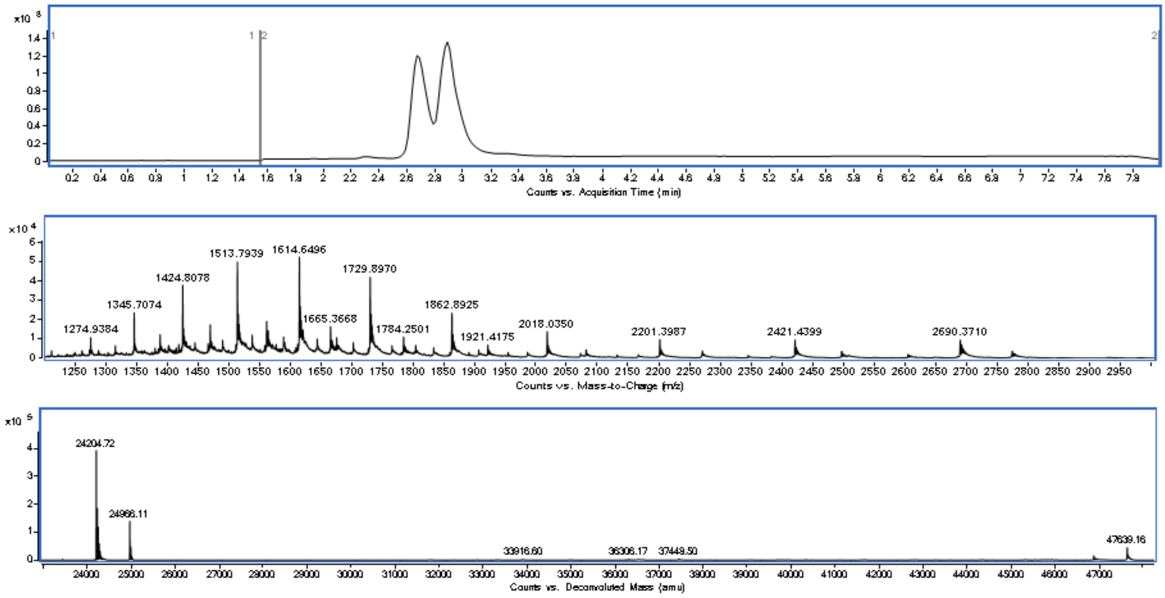

*Conjugate **41***

Expected masses: 24,268 Da (LC + PD), 25,029 Da (HC + PD), 47,639 Da (Fab)

Observed masses: 24,267; 25,029; 47,639 Da

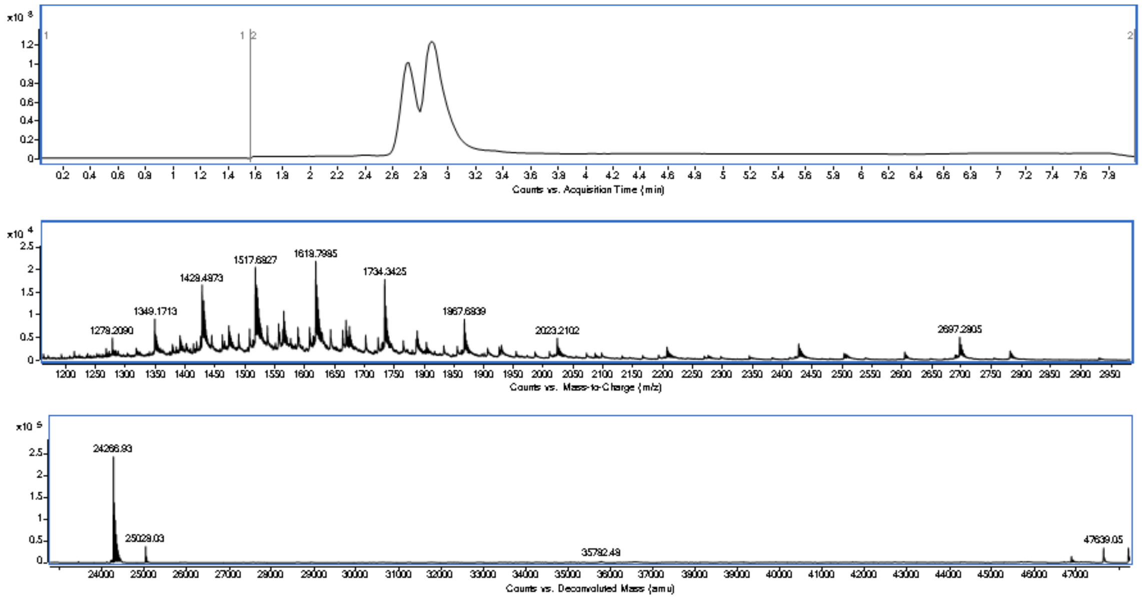

- Timepoint 6.5 h

*Conjugate **40***

Expected masses: 24,206; 24,967; 47,639 Da
Observed masses: 24,205; 24,966; 47,639 Da

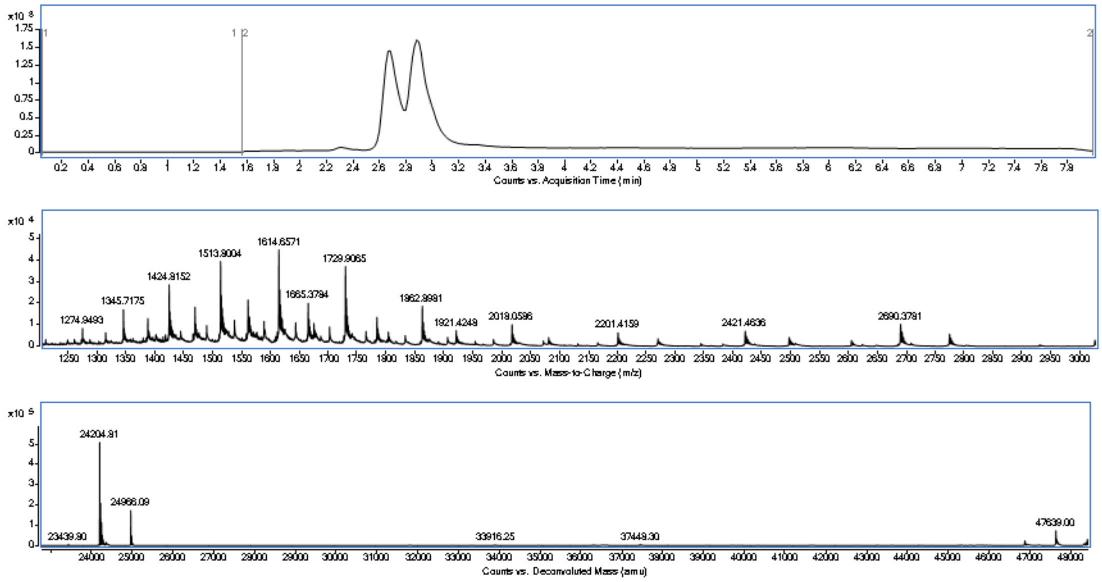

*Conjugate **41***

Expected masses: 24,268 Da (LC + PD), 25,029 Da (HC + PD), 47,639 Da (Fab)
Observed masses: 24,267; 25,029; 47,639 Da

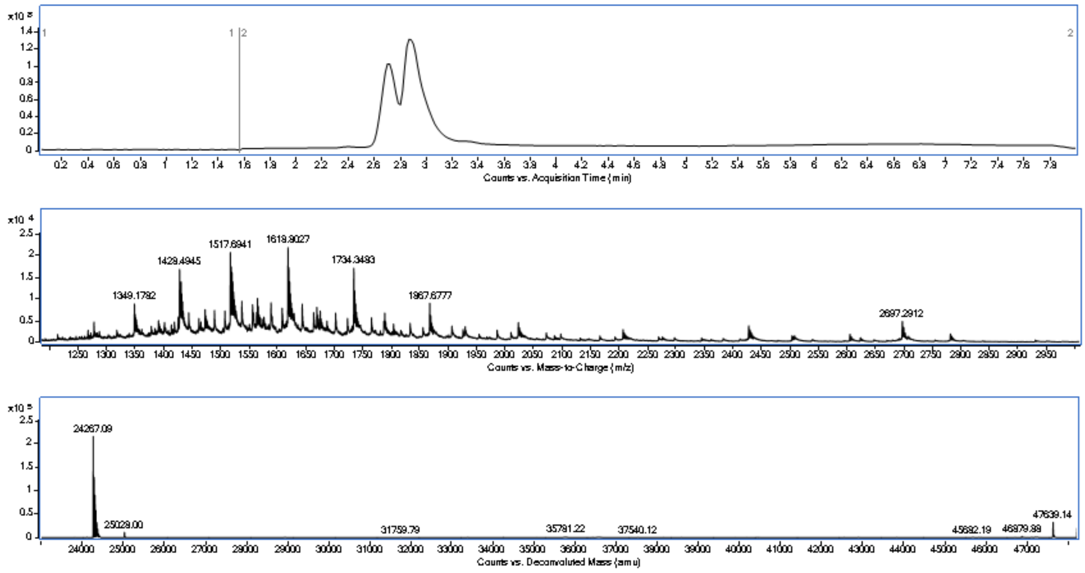

- Timepoint 24 h

*Conjugate **40***

Expected masses: 24,206; 24,967; 47,639 Da
Observed masses: 24,205; 24,966; 47,639 Da

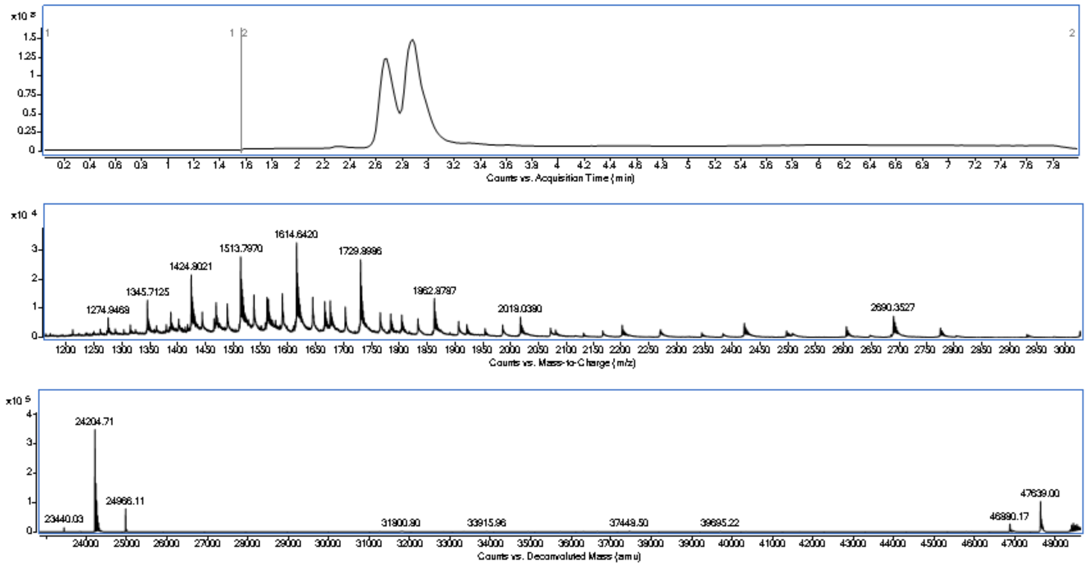

*Conjugate **41***

Expected masses: 24,268 Da (LC + PD), 25,029 Da (HC + PD), 47,639 Da (Fab)
Observed masses: 24,267; 47,639 Da

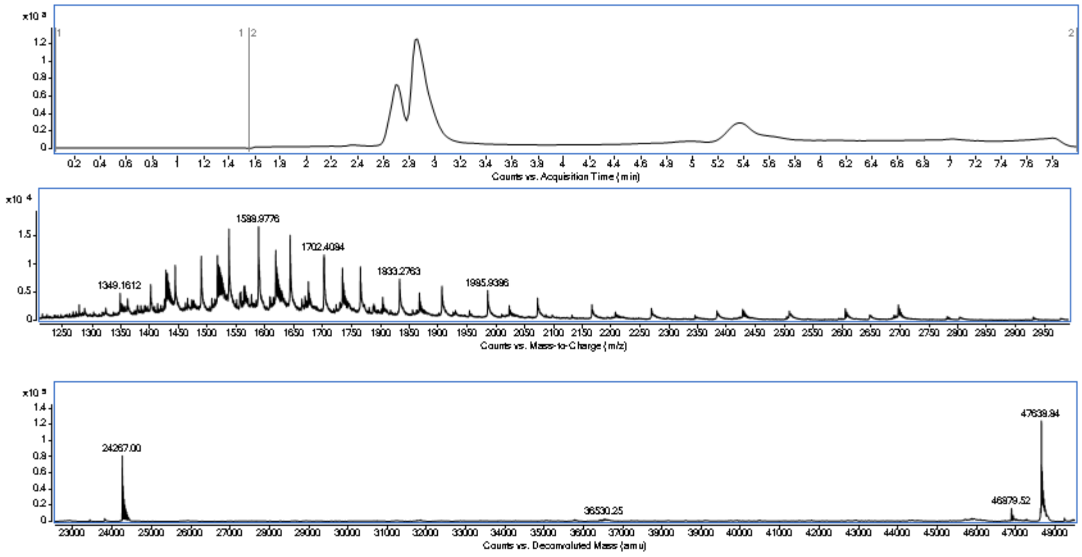

- Timepoint 48 h

*Conjugate **40***

Expected masses: 24,206; 24,967; 47,639 Da
Observed masses: 24,205; 24,966; 47,639 Da

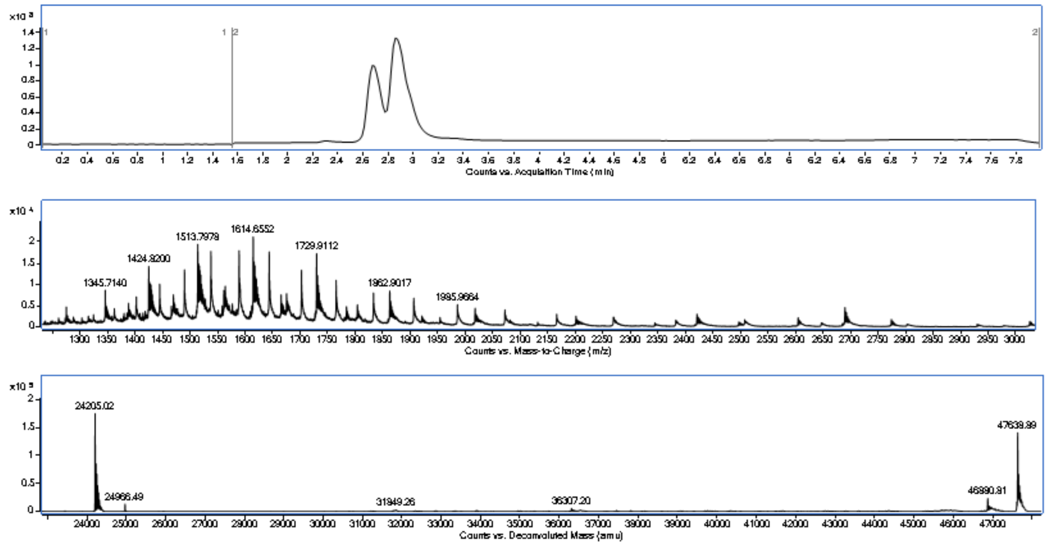

*Conjugate **41***

Expected masses: 24,268 Da (LC + PD), 25,029 Da (HC + PD), 47,639 Da (Fab)
Observed masses: 24,267; 47,639 Da

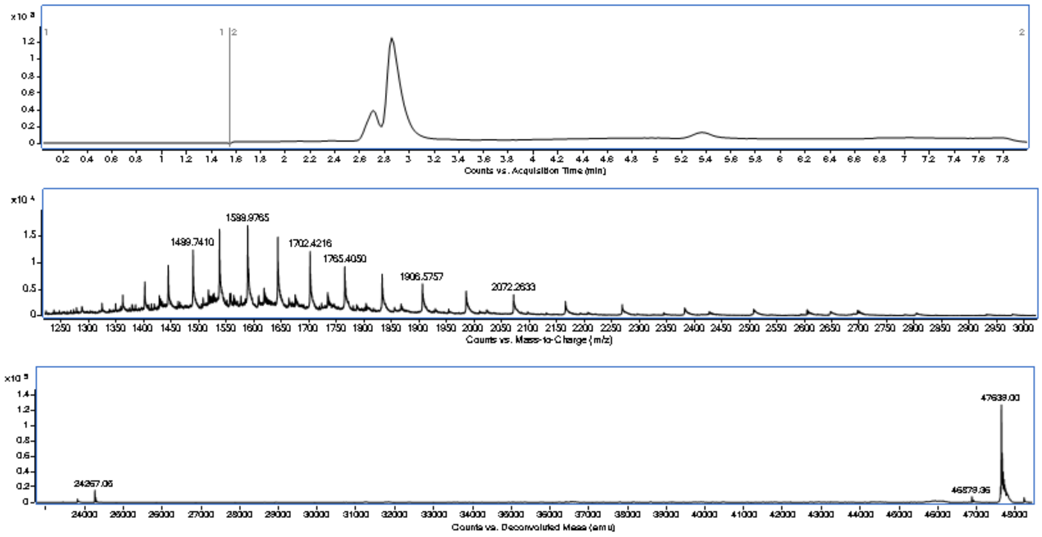

- *Timepoint 72 h*

*Conjugate **40***

Expected masses: 24,206; 24,967; 47,639 Da
Observed masses: 24,205; 24,966; 47,639 Da

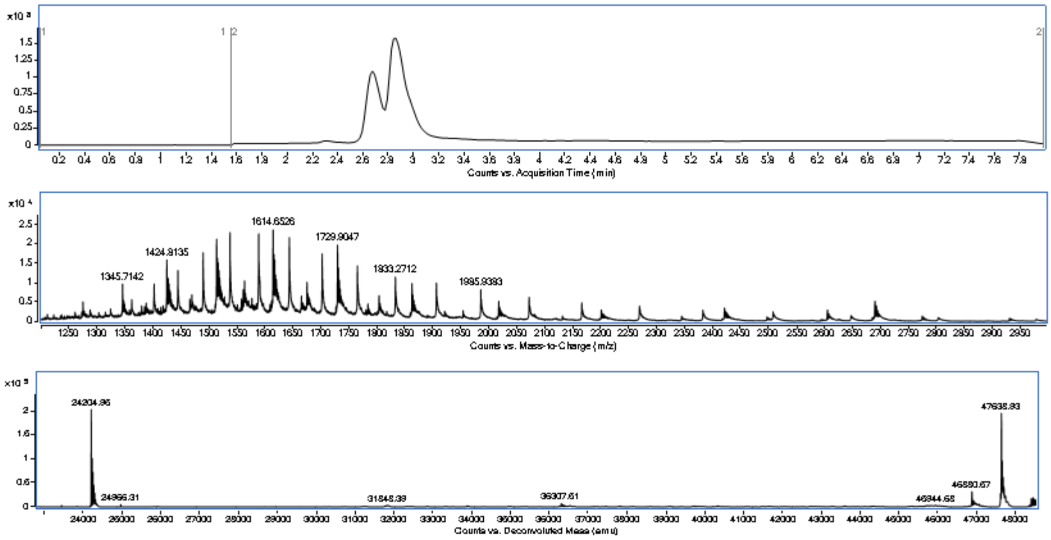

*Conjugate **41***

Expected masses: 24,268 Da (LC + PD), 25,029 Da (HC + PD), 47,639 Da (Fab)
Observed masses: 47,639 Da

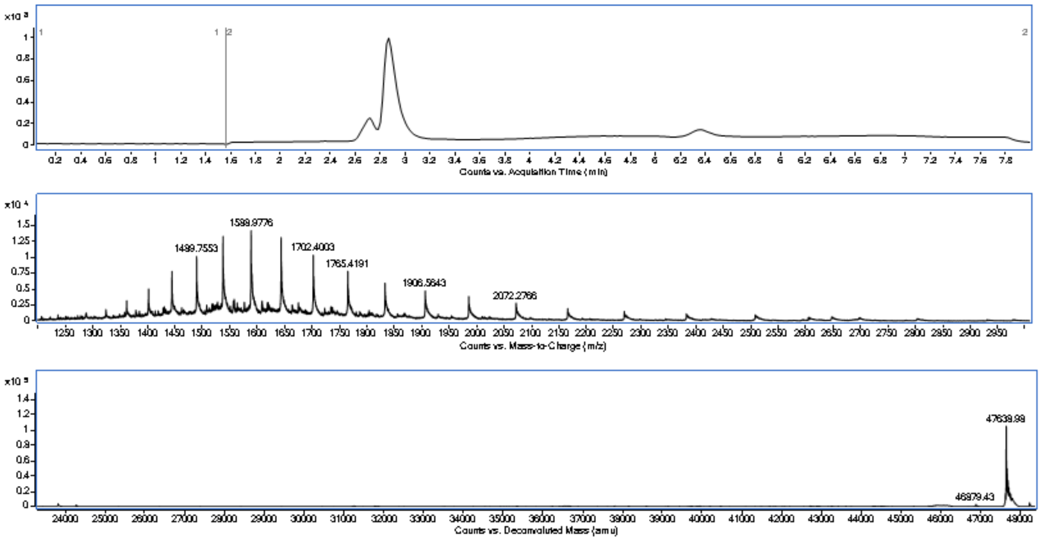

- *Summary*

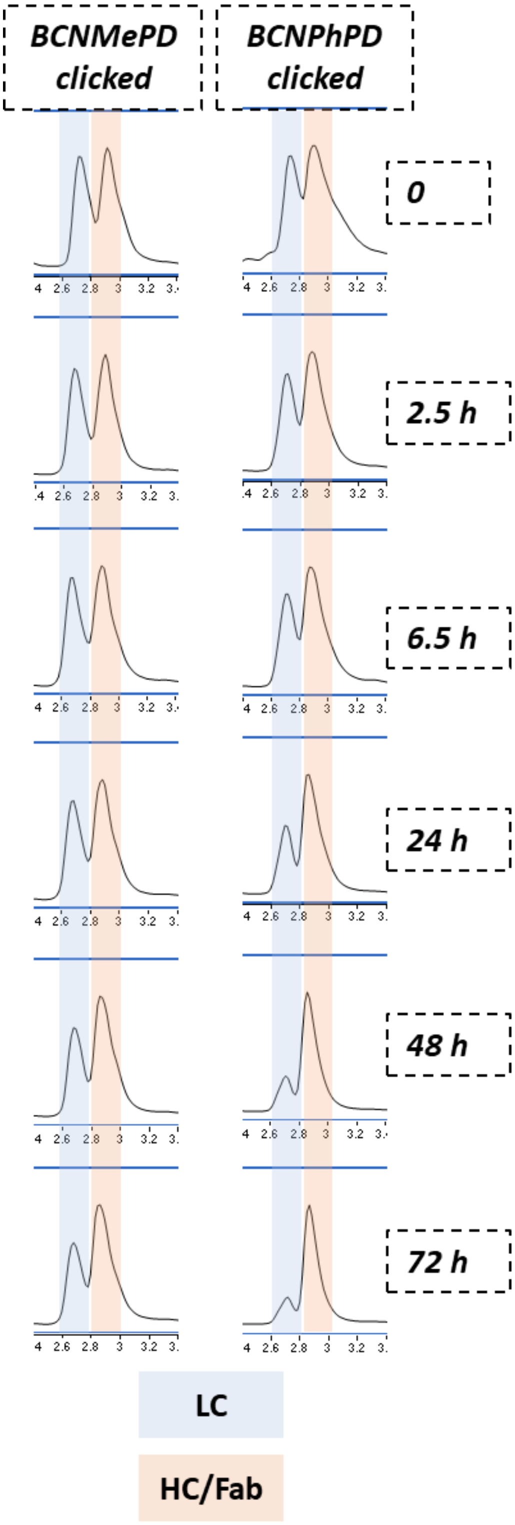

*Figure S7. Summary of deconjugation study from Fab fragment **22**, displaying HPLC spectra for both clicked PDs at each timepoint.*

- *SDS-PAGE gel*

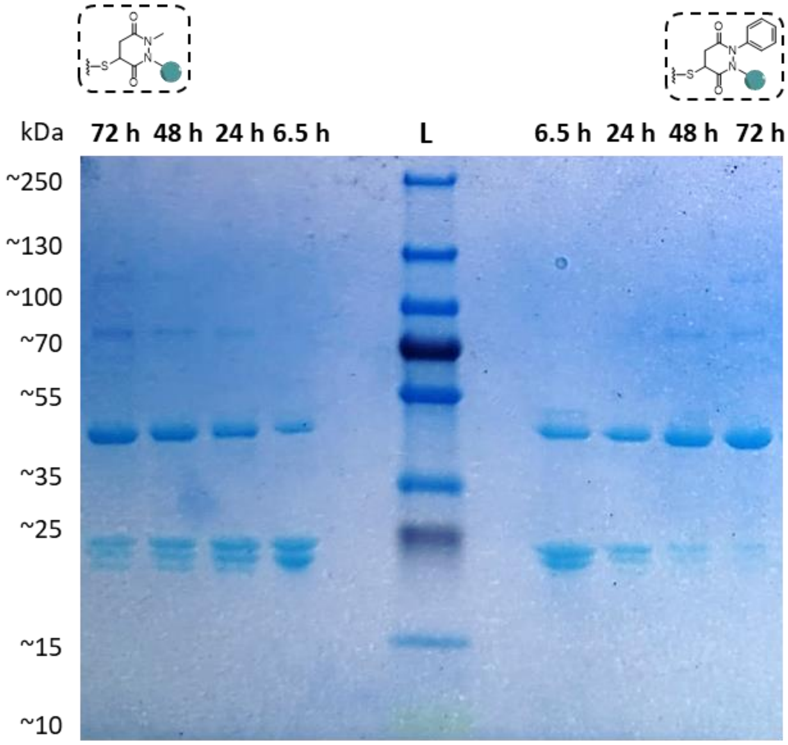

*Figure S8. SDS-PAGE gel of the release study of PDs from Fab-PD conjugates **40** and **41**, expected masses: ~29 kDa (LC/HC conjugates of Fab-PD) and/or ~50 kDa for re-oxidised Fab fragment **22**. Shows timepoints at 6.5 h, 24 h, 48 h and 72 h respectively for incubation of Fab-PD conjugates **40** and **41** at pH 7.4 at 37 °C.*

4.2.3 Competitive reaction between DiBrPD 23 and DiBrPD 24: tuning of DiBrPDs reactivity

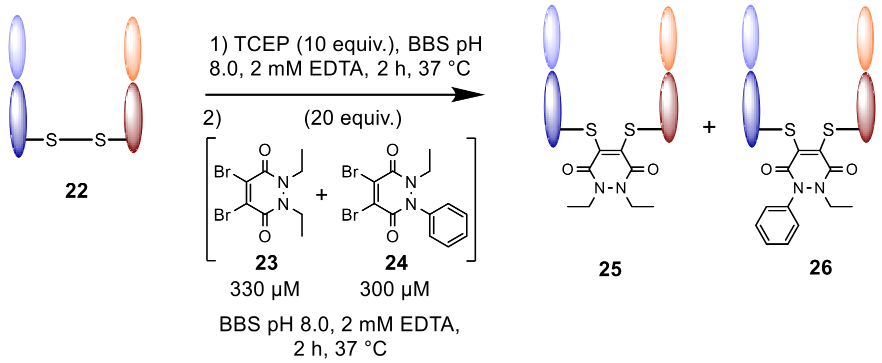

To a solution of Trastuzumab Fab **22** (20 μM, 40 μL) in BBS (25 mM sodium borate, 25 mM NaCl, 2 mM EDTA pH 8.0) was added TCEP·HCl (0.4 μL, 20 mM in H_2O, 10 equiv.). The mixture was incubated for 120 min at 37 °C under constant agitation (300 rpm). The excess of TCEP was removed by buffer-exchange in BBS (25 mM sodium borate, 25 mM NaCl, 2 mM EDTA, pH 8.0) using Zeba^{TM} Spin Desalting column. PDs **23** and **24** (0.8 μL, 1.1:1 ratio of **23**:**24** in CD_3CN, 20 equiv. of **24**) were then added and the reaction was incubated at 37 °C for 2 h. The excess of small molecules was removed by buffer-exchange in LCMS grade water using Zeba^{TM} Spin Desalting column for LCMS analysis.

LCMS analysis was carried out using Agilent 6510.

Expected masses: 47,805 Da (Fab + PD **23**); 47,853 Da (Fab + PD **24**)
Observed masses: 47,805 Da; 47,853; 23,733 (LC + PD **24** – 1 x Br); 23684 (LC + PD **23** – 1 x Br) Da

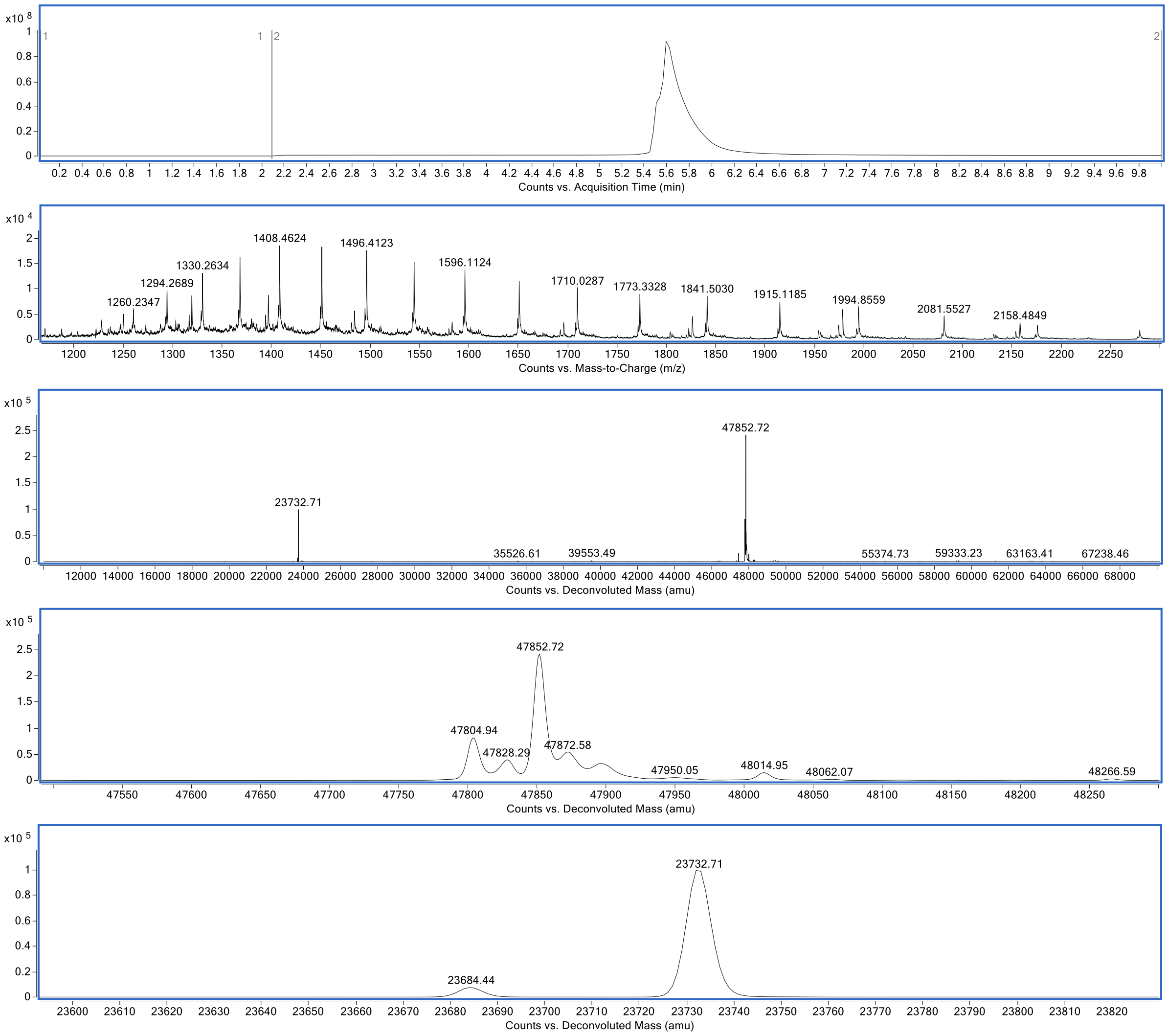
x10 8
Counts vs. Acquisition Time (min)
x10 4
1260.2347
1294.2689
1330.2634
1408.4624
1496.4123
1596.1124
1710.0287
1773.3328
1841.5030
1915.1185
1994.8559
2081.5527
2158.4849
Counts vs. Mass-to-Charge (m/z)
x10 5
23732.71
35526.61
39553.49
47852.72
55374.73
59333.23
63163.41
67238.46
Counts vs. Deconvoluted Mass (amu)
x10 5
47804.94
47828.29
47852.72
47872.58
47950.05
48014.95
48062.07
48266.59
Counts vs. Deconvoluted Mass (amu)
x10 5
23684.44
23732.71
Counts vs. Deconvoluted Mass (amu)

4.2.4 Stability of hydrolysed PD to excess thiols

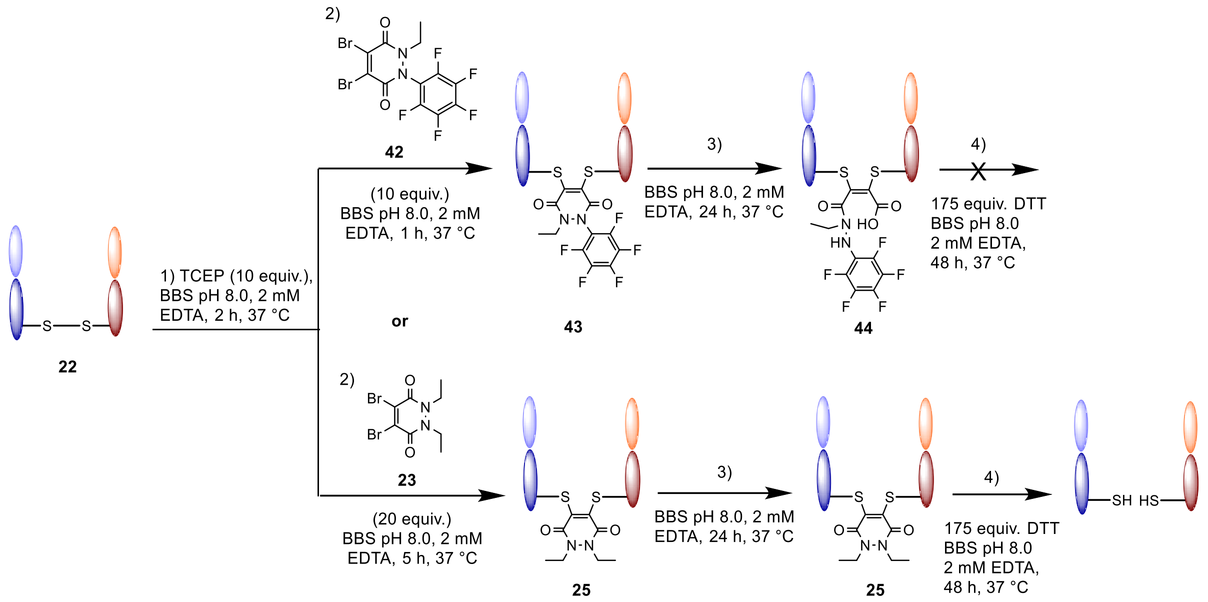

To a solution of TrastuzumabFab **22** (50 µM, 150 µL) in BBS (25 mM sodium borate, 25 mM NaCl, 2 mM EDTA pH 8.0) was added TCEP·HCl (3.8 µL, 20 mM in d.d. H_2O, 10 equiv.). The mixture was incubated for 120 min at 37 °C under constant agitation (300 rpm). The excess of TCEP was removed by buffer-exchange in BBS (25 mM sodium borate, 25 mM NaCl, 2 mM EDTA, pH 8.0) using Zeba™ Spin Desalting column. Reduced Fab was then split in two batches (~45 µM, 75 µL). To the first one, diBr *N*-Et,*N'*-F_5PhPD **42** (0.68 µL, 50 mM in MeCN, 10 equiv.) was added and to the second one, diBr N,N'- diEt **23** (0.68 µL, 50 mM in MeCN, 10 equiv.) was added. Both reactions were incubated at 37 °C for 1 h or 16 h. After this, the excess of small molecules was removed by buffer-exchange in LCMS grade water using Zeba™ Spin Desalting column and LCMS analysis were run. A supplementary Zeba™ Spin Desalting columns was used to remove all the excess small molecules and buffer-swapped in BBS pH 8.0, 2 mM EDTA. Both reactions were incubated at 37 °C for 24 h and LCMS analyses were run in LCMS grade water after this. DTT (9.8 µL, 50 mM in MeCN, 175 equiv.) was then added to both reactions and they were incubated at 37 °C. LCMS analysis were taken after 2 h, and additional 48 h for Fab-PD conjugate **43**.

LCMS analyses were carried out on Agilent 6510.

Conjugate **43**

Expected mass: 47,943 Da (Conjugate **43**)

Observed masses: 47,942 Da, 47,962 Da (small hydrolysis observed due to LCMS analysis running after a couple of hours)

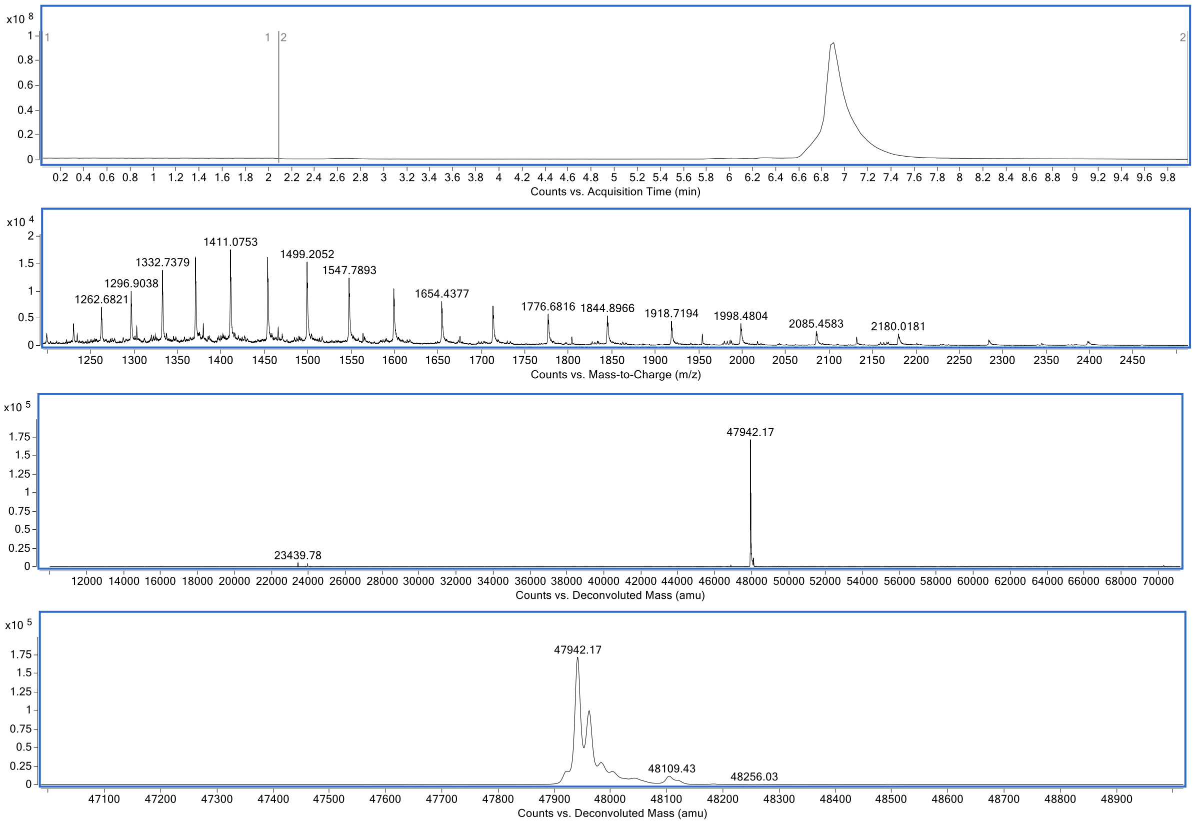

Conjugate **44**

Expected mass: 47,961 Da (Conjugate **44**)
Observed masses: 47,962 Da

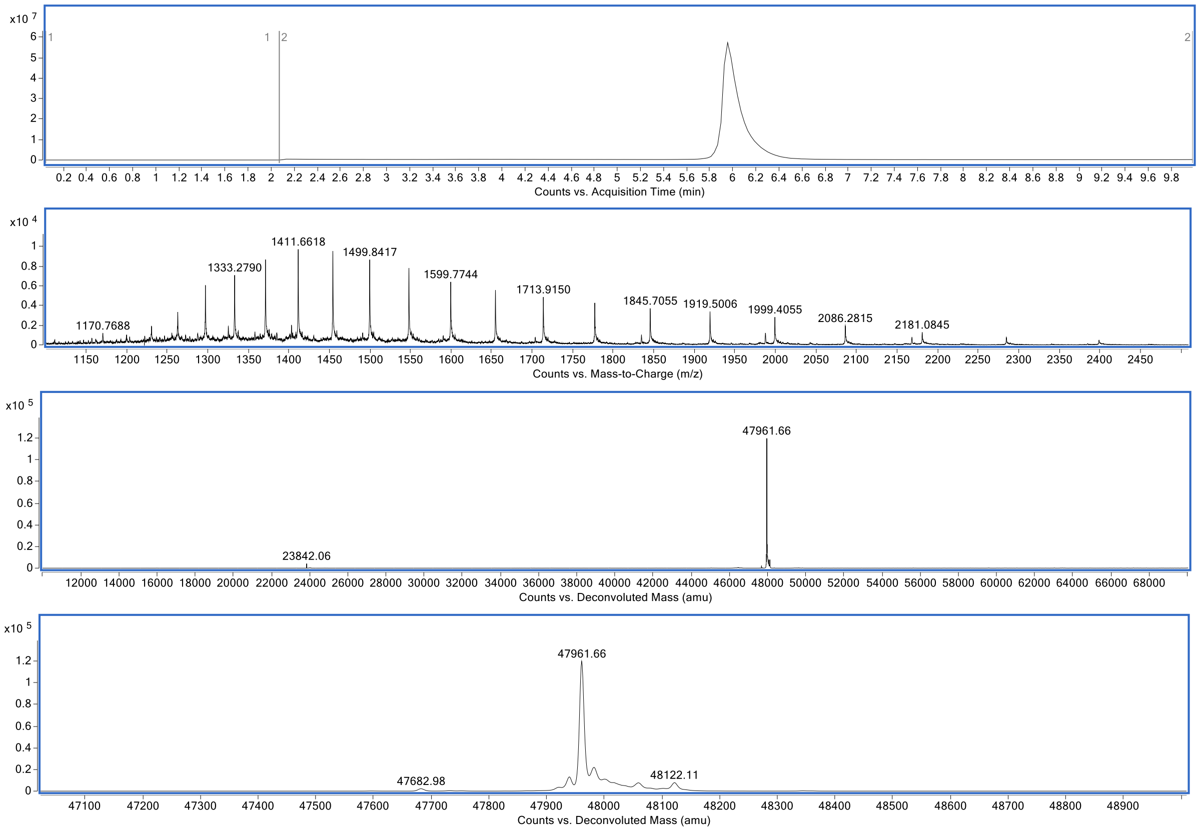

Conjugate **44** after 4)

t = 2 h

Expected mass: 47,961 Da (Conjugate **44**)
Observed masses: 47,961 Da

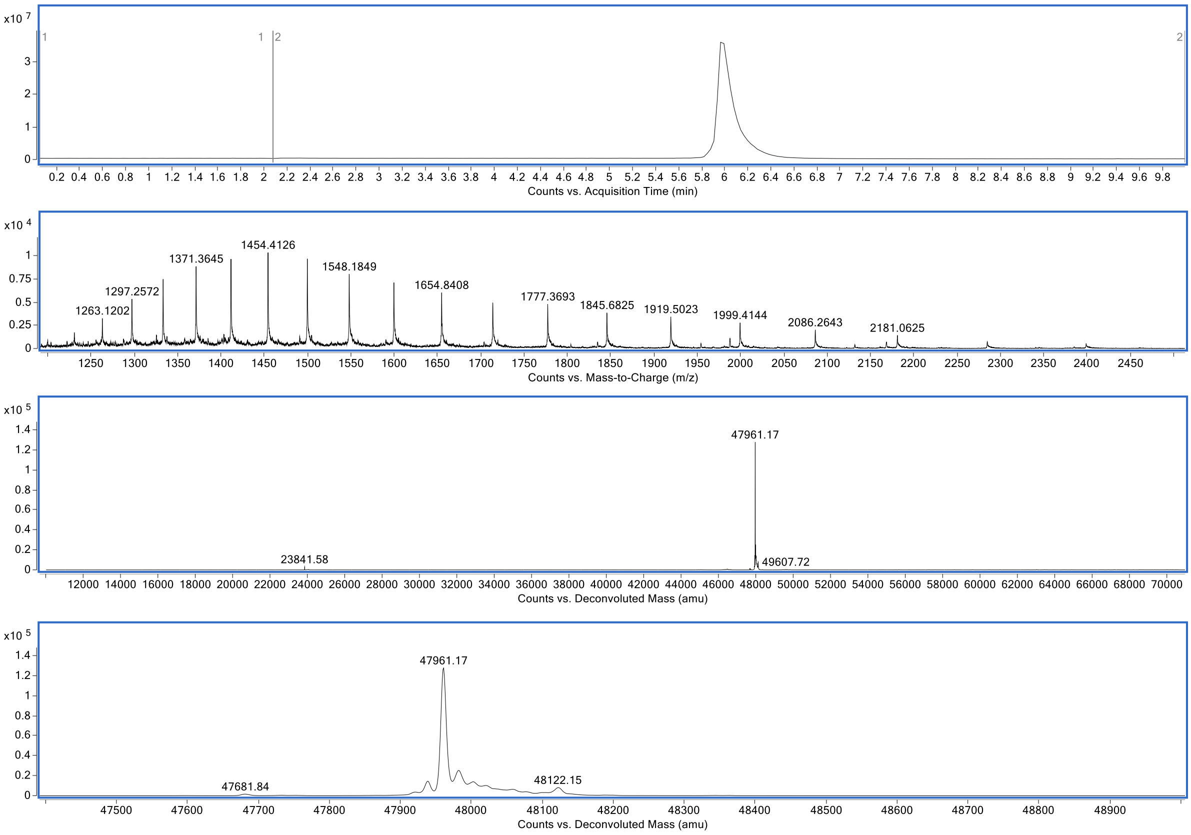

t = 48 h

Expected mass: 47,961 Da (Conjugate **44**)
Observed masses: 47,961 Da

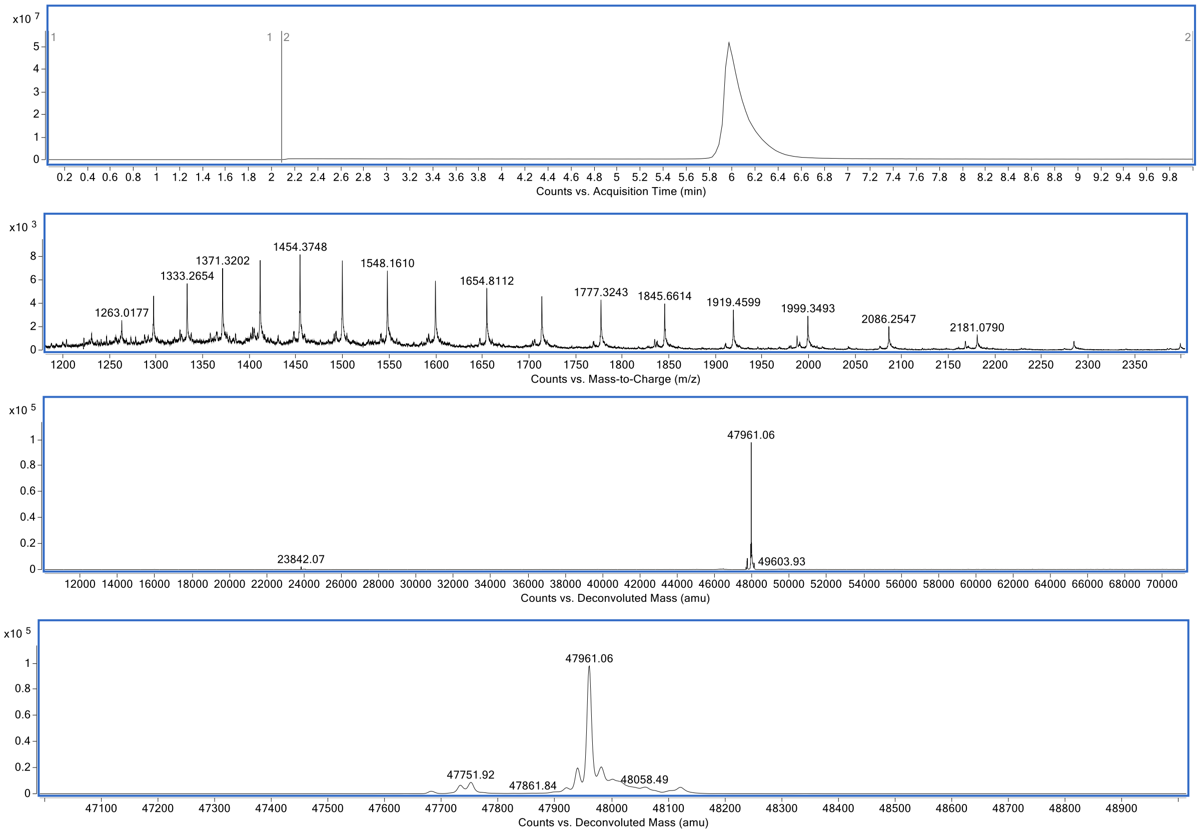

Conjugate **25**

Expected mass: 47,806 Da (Conjugate **25**)
Observed mass: 47,805 Da

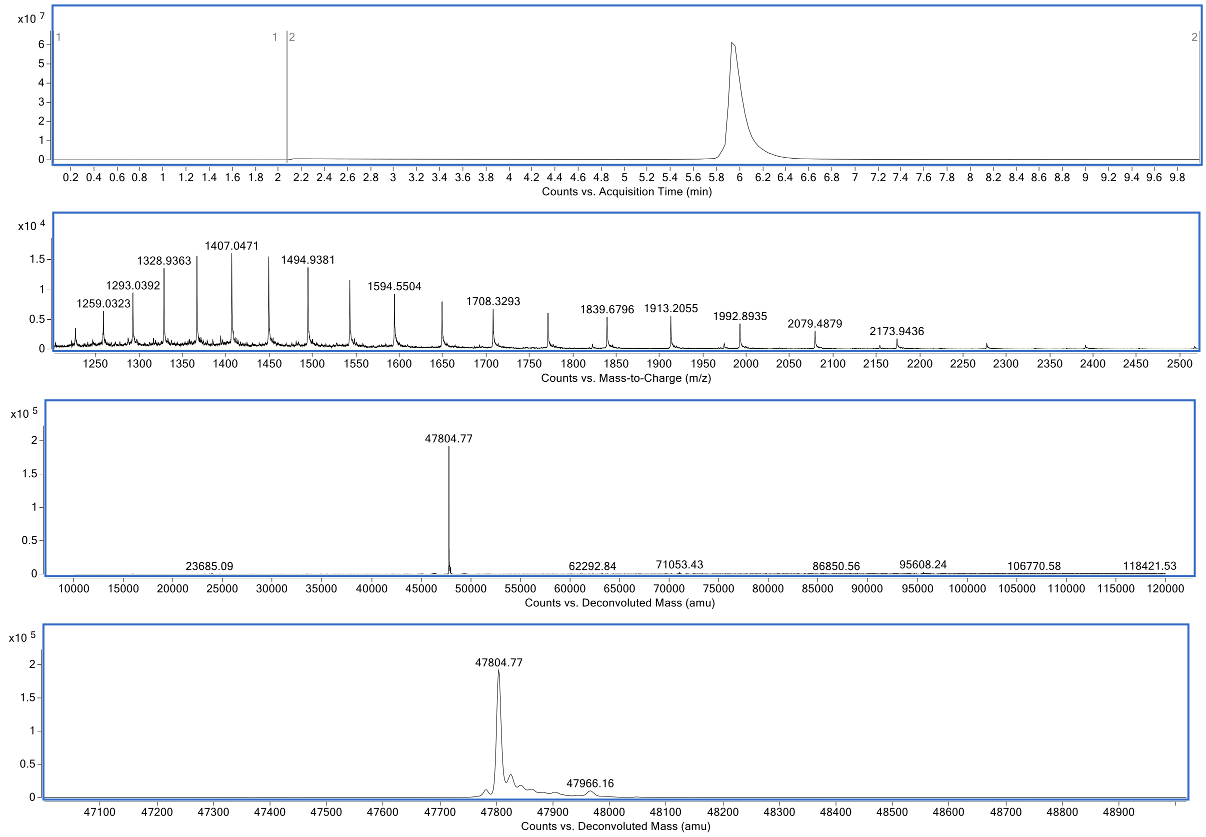

Conjugate **25** after 3)

Expected mass: 47,806 Da (Conjugate **25**)
Observed mass: 47,806 Da

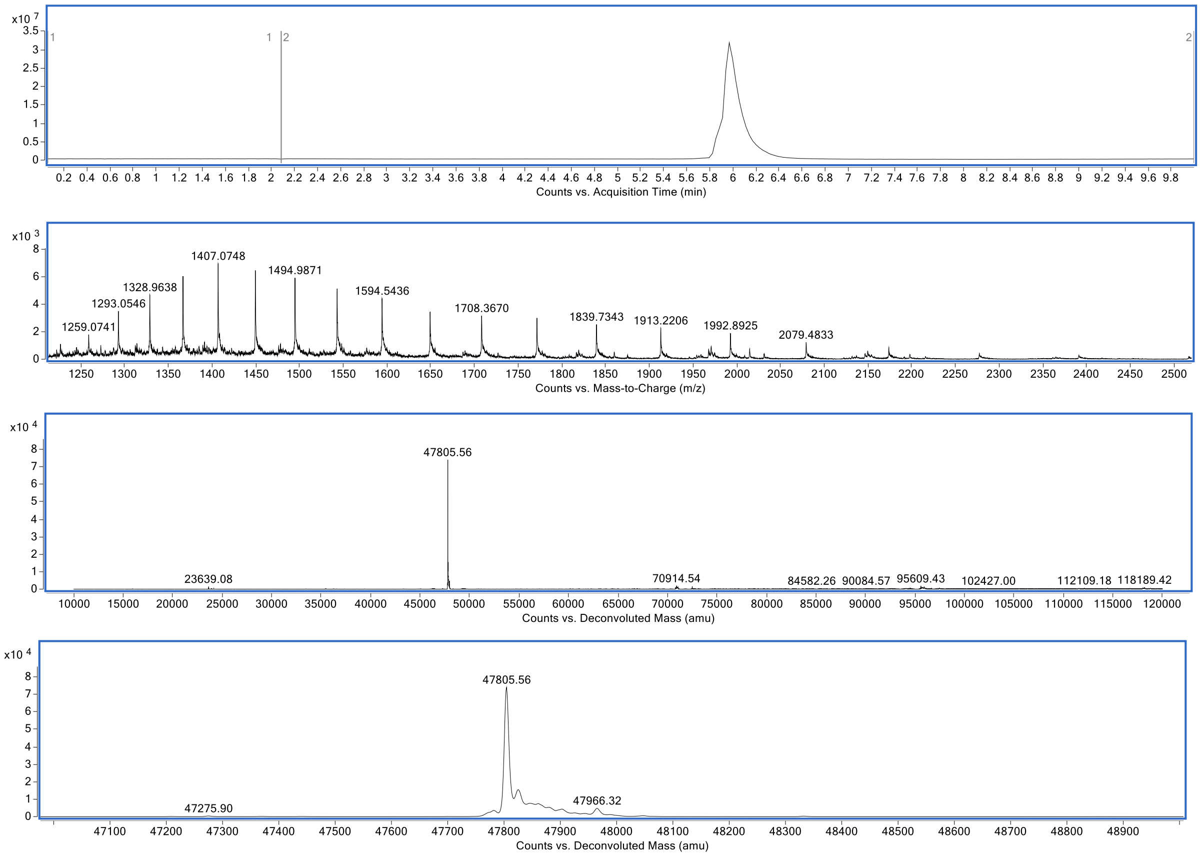

Conjugate **25** after 4)

t = 2 h

Expected masses: 23,439 Da (LC of **22**); 24,201 Da (HC of **22**); 47,639 Da (**22**)
Observed masses: 23,440 Da; 24,201 Da

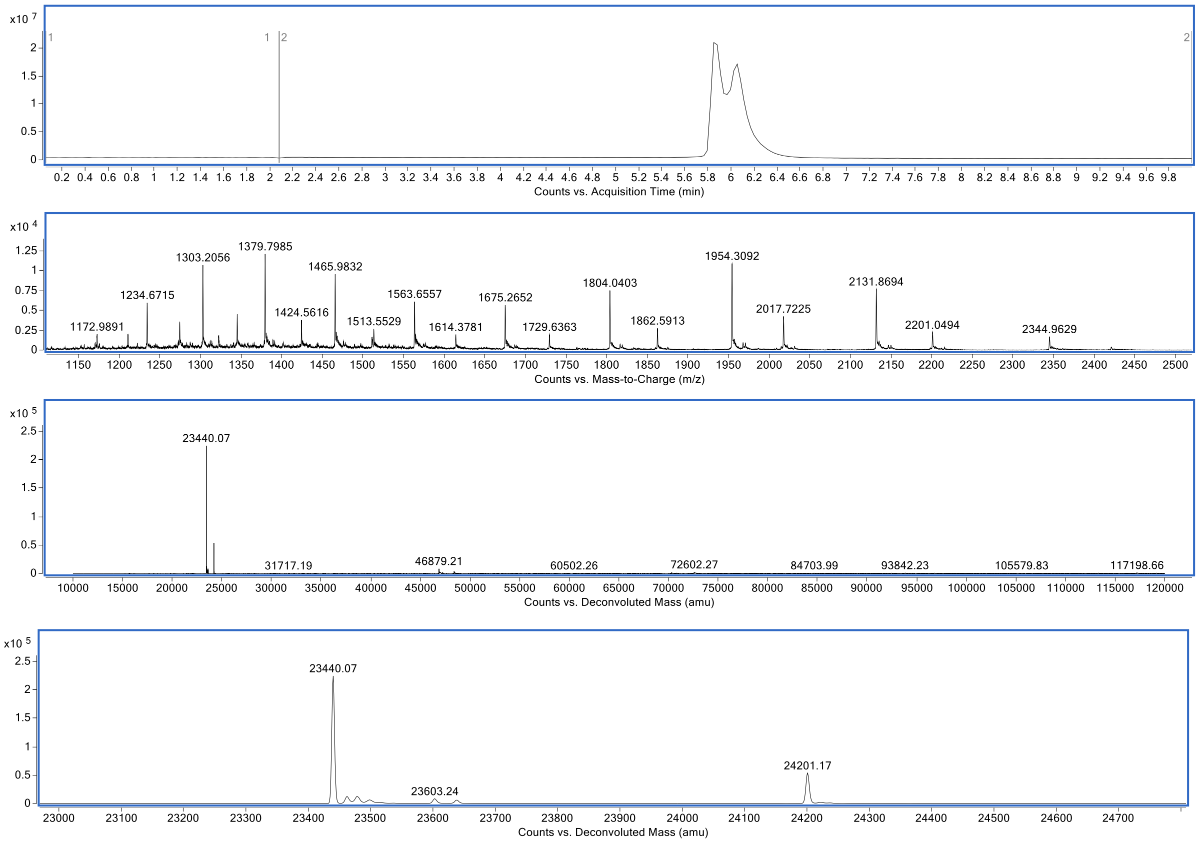

4.3 Hydrogel experiments

4.3.1 Synthesis of 4-Arm PEG 51 and 46

Synthesis of 4-Arm PEG 51

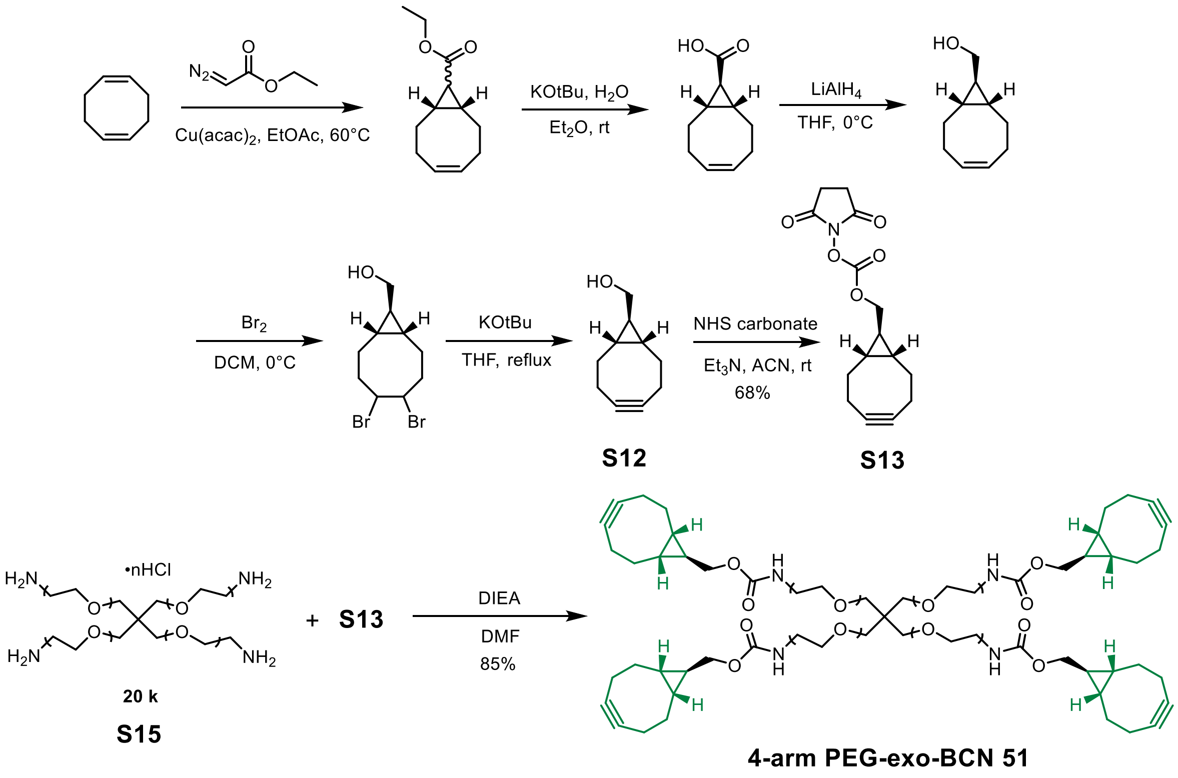

Scheme S5. Synthesis of 4-arm PEG-exo-BCN 51

S12: This compound was synthesized following reported procedures.[15, 16]

^{1}H NMR (400 MHz, $CDCl_3$) δ 3.55 (dd, J = 6.3, 0.6 Hz, 2H), 2.42 (ddd, J = 13.7, 3.5, 1.8 Hz, 2H), 2.36 – 2.23 (m, 2H), 2.22 – 2.10 (m, 2H), 1.55 (s, 1H), 1.46 – 1.29 (m, 2H), 0.75 – 0.60 (m, 3H).

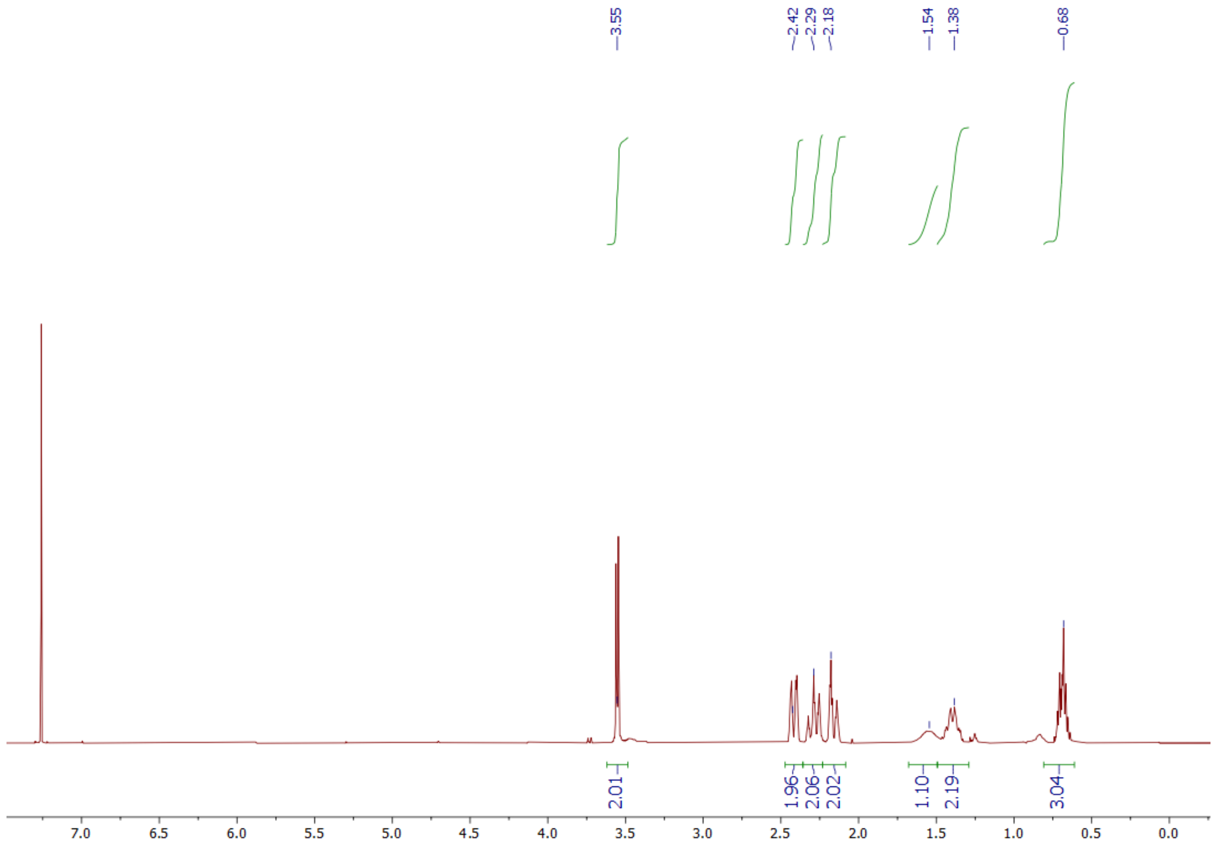

S13: To a stirred solution of **S12** (170 mg, 1.1 mmol, 1.0 equiv.) in acetonitrile (5 ml) at 21 °C, triethylamine (470 µl, 3.3 mmol, 3.0 equiv.) and NHS carbonate (570 mg, 1.6 mmol, 1.5 equiv.) were added sequentially. The reaction mixture was stirred at 21 °C under nitrogen for 16 h. Upon completion, solvent was removed, and the crude product was subject to column chromatography (30% to 50% EtOAc/cHex) to yield **S13** as a pale white solid (224 mg, 68%).

m.p. 127.1 – 129.1 °C; **^{1}H NMR** (400 MHz, $CDCl_3$) δ 4.26 (d, J = 6.9 Hz, 2H), 2.84 (s, 4H), 2.44 (dq, J = 13.5, 2.8 Hz, 2H), 2.37 – 2.25 (m, 2H), 2.24 – 2.10 (m, 2H), 1.47 – 1.35 (m, 2H), 0.91 – 0.75 (m, 3H); **^{13}C NMR** (101 MHz, $CDCl_3$) δ 168.81, 151.81, 98.78, 77.46, 77.34, 77.14, 76.82, 76.12, 33.15, 25.61, 23.56, 22.99, 21.36; **HRMS** (ESI) calcd for $C_{15}H_{18}NO_5$ $[M+H]^+$ 292.1179, observed 292.1180, calcd for $[2M+H]^+$ 583.2286, observed 583.2289.

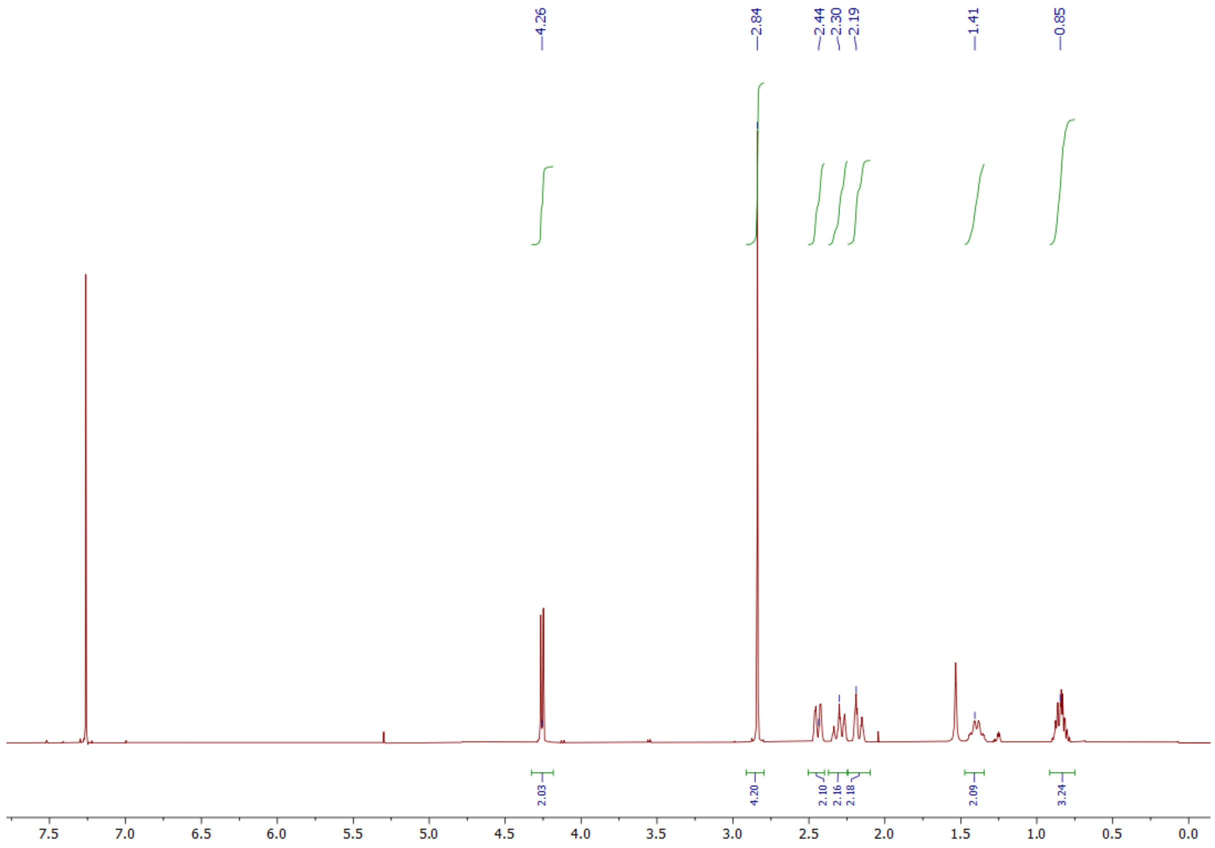

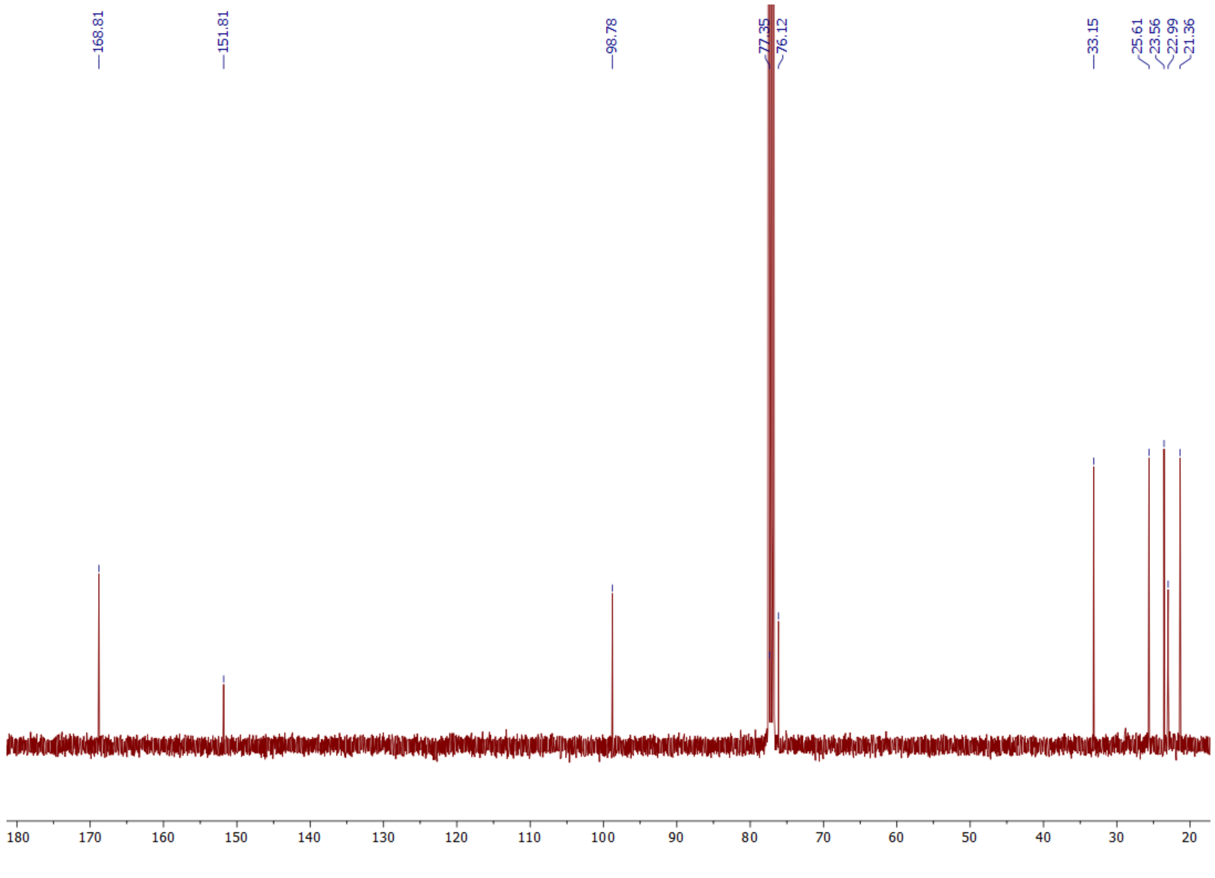

4-Arm PEG-*exo*-BCN 51: 20 k 4-arm PEG-NH_2 hydrochloride **S15** (400 mg, 0.08 mmol amine, 1.0 equiv.) and **S13** (42 mg, 0.14 mmol, 7.0 equiv.) were dissolved in dimethylformamide (2.2

mL). *N,N*-Diisopropylethylamine (137.5 µL, 0.8 mmol, 10.0 equiv.) was added to the mixture, and the reaction was stirred for 48 h. The crude mixture was diluted with water and dialyzed using 2K MWCO dialysis tube (Spectra/Por® 6) against water for 48 h, followed by lyophilization to yield **51** a white powder (360 mg, 85%).

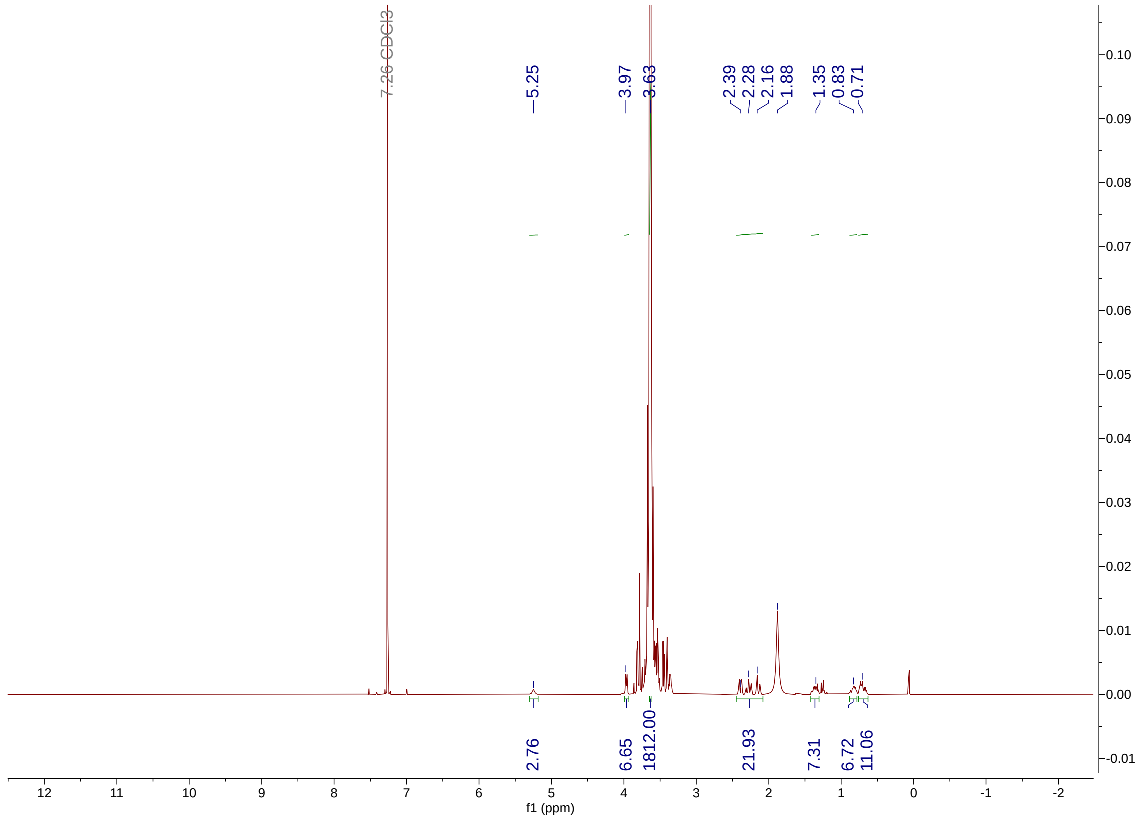

Functionalization of the 4-Arm PEG amine with NHS ester BCN was confirmed to be >90% converted by ^1H-NMR ($CDCl_3$) by comparing integral values for characteristic BCN peaks (δ 2.39, 2.27, 2.14) with those from the PEG backbone (δ 3.63).

Synthesis of 4-Arm PEG-azide 46

10 kDa 4-arm PEG-NH_2 hydrochloride **S15** (201.6 mg, 0.020 mmol amine, 1.0 equiv.) and azido-acetic acid (6.2 mg, 0.103 mmol, 5.2 equiv.) were dissolved in dimethylformamide (1 mL). *N*,*N*-Diisopropylethylamine (50 µL) was added to the mixture, and the reaction was stirred for 24 h at 21 °C. The crude mixture was precipitated into cold diethyl ether (10 mL) then centrifuged (5000 rcf, 5 min, 4 °C). The supernatant was removed, the precipitate dissolved with water (2 mL) and dialyzed using 2 kDa MWCO dialysis tube (Spectra/Por® 6) against water for two days, followed by lyophilization to yield a white powder (199.9 mg, 97%).

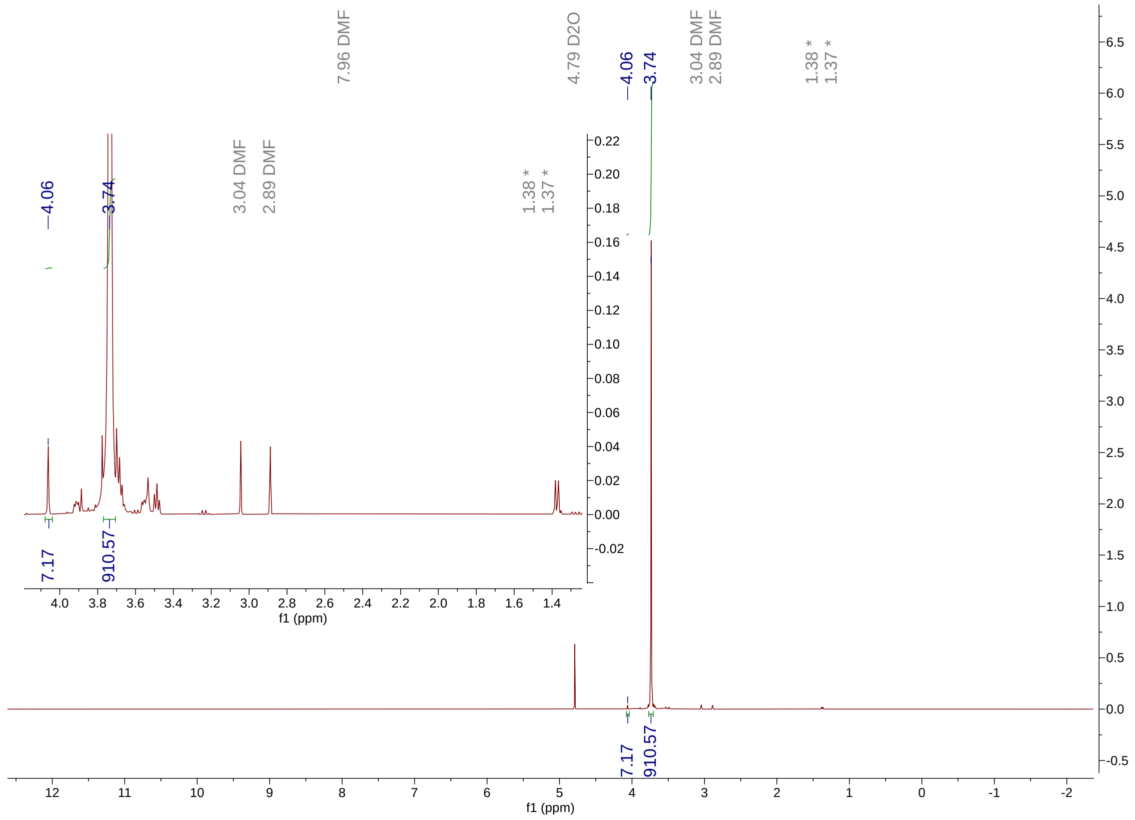

Functionalization of the 4-Arm PEG amine with azido acetic acid was confirmed to be >90% converted by ^{1}H-NMR (D_2O) by comparing integral values for characteristic alpha-CH_2 protons to the azide group (δ 4.06) with those from the PEG backbone (δ 3.74). Trace amounts of DMF (δ 7.96, 3.04 and 2.89) and suspected *N*,*N*′-diisopropylurea (*, CH_3) are found in the ^{1}H NMR spectra.

4.3.2 *Synthesis of 4-Arm PEG-N_3*

Synthesis of 4-Arm PEG MePD-CGY 49

To a solution of BCN-MePD-CGY **47** (0.97 mg, 0.0011 mmol, 172.4 µL) in 30% acetonitrile in acetate buffer (100 mM, pH = 5, 1 mM EDTA) was added a solution of 10 kDa 4-Arm PEG-azide (12 mg, 0.0011 mmol, 324 µL) dissolved in 30% acetonitrile in acetate buffer (100 mM, pH = 5, 1 mM EDTA). The reaction was allowed to occur at 20 °C for 2 h. The reaction was then frozen at -80 °C and lyophilised to give compound **49** as a white solid which was used for hydrogel formation.

Synthesis of 4-Arm PEG-PhPD-CGY 50

To a solution of BCN-PhPD-CGY **48** (1.04 mg, 0.0011 mmol, 124.2 µL) in 30% acetonitrile in acetate buffer (100 mM, pH = 5, 1 mM EDTA) was added a solution of 10 kDa 4-Arm PEG-azide (12 mg, 0.0011 mmol, 324 µL) dissolved in 30% acetonitrile in acetate buffer (100 mM, pH = 5, 1 mM EDTA). The reaction was allowed to occur at 20 °C for 2 hours. The reaction was then frozen at -80 °C and lyophilised to give the title compound **50** as a white solid which was used for hydrogel formation.

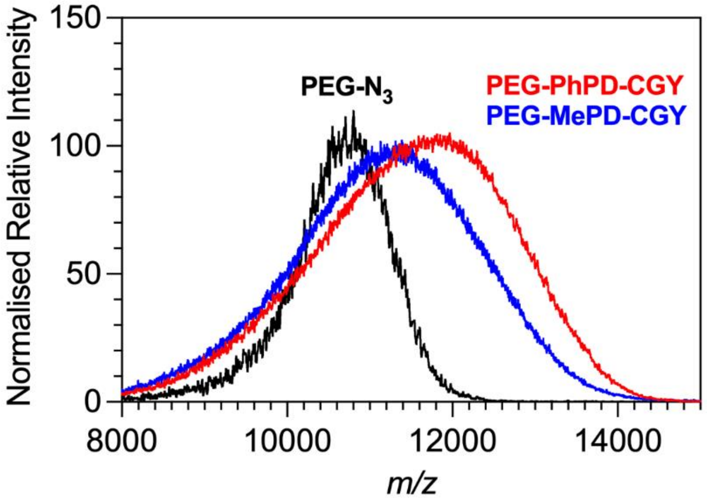

*Figure S9. MALDI-ToF spectra of the 4-Arm PEG-PD-peptides 49 and 50 showing an increase in molecular weight after reaction of 4-Arm-PEG-azide **46**.*

4.3.3 Hydrogels 52 and 53 formation

A stock solution of 4-Arm PEG-*exo*-BCN (53.84 mg) was prepared at a concentration of 180 mg/mL by dissolving in 299 µL of acetate buffer (100 mM, pH = 5, 1 mM EDTA). A stock solution of 4-Arm-PEG-PD-peptide was prepared at a concentration of 120 mg/mL by reconstituting the 4-Arm-PEG-PD peptides in acetate buffer (100 mM, pH = 5, 1 mM EDTA). To make the hydrogels, 28 µL of PEG-N3-PD-Peptide and 28 µL of PEG-BCN was mixed briefly and then transferred into the PDMS mould. A coverslip was placed on top of the mould and the samples were allowed to cure for 2.5 hours at room temperature. After curing, the hydrogels were transferred to a 12 well plate and washed with 100 mM acetate buffer (pH = 5) with 1 mM EDTA five times, then the hydrogels **52** and **53** were stored at 4 ºC in 100 mM acetate buffer (pH = 5) with 1 mM EDTA overnight.

n = 3 hydrogels were prepared for each PD-peptide combination for a total of 9 hydrogels.

4.3.4 Peptide Release from the Hydrogels

The gels were transferred to 7 mL glass vials. Each hydrogel was blotted with a Kimwipe® to remove excess buffer. Timepoint zero was defined to be the time at which 500 µL of 1x PBS was added to the hydrogels. Hydrogels were sampled at 30 m, 1 h, 2 h, 4 h, 8 h and 24 h. Each hydrogel was sampled by removing 400 µL of supernatant and transferring to a HPLC vial, followed by the addition of 400 µL of 1xPBS to the hydrogel sample. A 5 µL aliquot of a 20

mM TCEP solution was added to the HPLC vials to prevent oxidation of the peptides during the HPLC measurement. The concentration of peptide released was determined using LC-MS operating in selective ion monitoring mass spectrometry (SIMS) based on the area under the curve, with the target mass being 342 m/z, corresponding to the $[M+H]^+$ for the released CGY peptide. Standards of CGY were prepared to generate a calibration curve to determine the concentration of the CGY peptide released.

5. DFT calculations

Summary of Calculated Gibbs Free Energies for Pyridazinediones and Adducts[a]

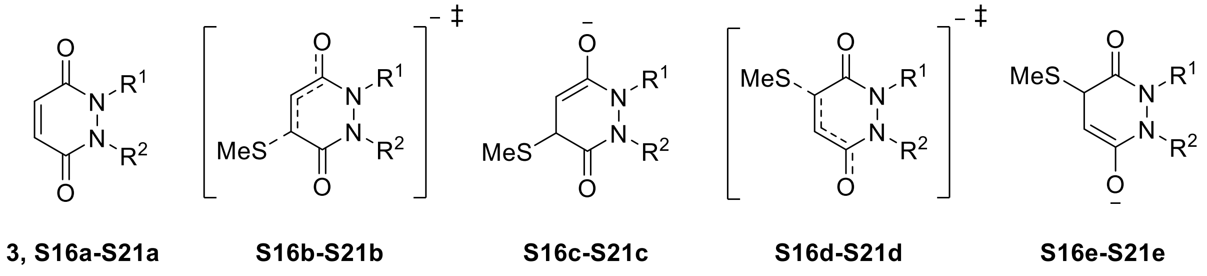

	R^1	R^2	*G*(a)/Hartree	*G*(b)/Hartree	*G*(c)/Hartree	*G*(d)/Hartree	*G*(e)/Hartree
**3**	Et	Ph	–724.156855	n.c.	n.c.	n.c.	n.c.
**S16**	Me	Me	-493.215354	-931.393746	-931.398275	n.a.	n.a.
**S17**	Me	Ph	-684.877029	-1123.057143	-1123.061005	-1123.058371	-1123.065669
**S18**	Me	4-$H_2NC_6H_4$	-740.223096	-1178.400572	-1178.406836	-1178.402938	-1178.404563
**S19**	Me	4-FC_6H_4	-784.12805	-1222.308609	-1222.316856	-1222.309796	-1222.312406
**S20**	Me	4-$O_2NC_6H_4$	-889.370319	-1327.553827	-1327.569029	-1327.555698	-1327.558824
**S21**	Me	C_6F_5	-1181.085565	-1619.268578	-1222.316856	-1619.268115	-1222.312406

Table S7. Calculated Gibbs free energies for pyridazinediones and adducts. All energies calculated using M06-2X/6-311+G(d,p)/CPCM(H_2O). n.c. = not calculated. n.a. = not applicable.

Calculated Activation Energies, Predicted Regioselectivities and Predicted Rates for Michael Addition

PD	$\Delta G^{\ddagger}$(beta to Ar)/kJ mol^{-1}	$\Delta G^{\ddagger}$(gamma to Ar)/kJ mol^{-1}	$\Delta\Delta G^{\ddagger}$/kJ mol^{-1}	Predicted Ratio (beta-to-Ar:gamma-to-Ar)^{a}	Predicted Relative Rate of Addition^{b}
**S16a**	n.a.	44.3	n.a.	n.a.	1
**S17a**	39.7	36.5	3.2	1:3.7	14.5
**S18a**	46.7	40.5	6.2	1:12.3	2.5
**S19a**	38.6	35.5	3.1	1:3.5	22.4
**S20a**	30.8	25.9	4.9	1:7.3	931
**S21a**	32.1	33.3	−1.2	1.6:1	107

*Table S8. Calculated activation energies, predicted regioselectivities and predicted rates for Michael Addition for PD **S16a-S21a.** ^{a}Ratio predicted as exp(−ΔΔG/RT) with T = 298.15 K. ^{b}Rate predicted as k = A[exp(−ΔG‡$_{syn\text{-}to\text{-}Ar}$/RT) + exp(−ΔG‡$_{syn\text{-}to\text{-}Me}$/RT)] at T = 298.15 K, normalised to k_{S16a} = 1.*

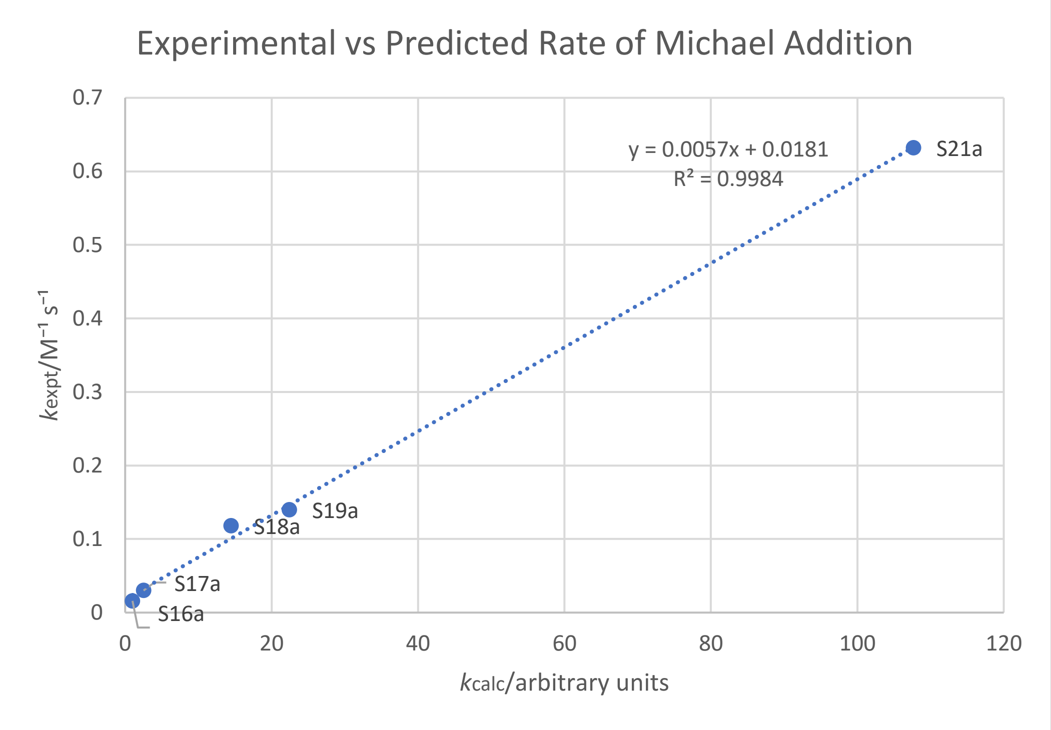

*Graph S5. Experimental k_{MA} found in kinetic assays in section 3.3 for PD **1-5** vs predicted rate of Michael Addition found by DFT calculations for **S16a-S19a** and **S21a**.*

Coordinates and Energies (M06-2X/6-311+G(d,p)/CPCM(H_2O))

Methanethiol

E = -438.678993

G = -438.656895

C	-1.15754	0.01928	0.00000
H	-1.52461	-1.00516	-0.00000
H	-1.51512	0.52622	-0.89303
H	-1.51512	0.52622	0.89303
S	0.66195	-0.08712	0.00000
H	0.90884	1.23092	0.00000

Methanethiolate

E = -438.209580
G = -438.195249

S	-0.00000	0.00000	0.71011
C	0.00000	-0.00000	-1.13071
H	-0.00000	1.01785	-1.52582
H	0.88148	-0.50892	-1.52582
H	-0.88148	-0.50892	-1.52582

Ethyl phenyl PD (3)
E = -724.339266
G = -724.156855

C	-0.70406	1.70936	0.32781
C	-2.12968	1.95428	0.06393
C	-2.92017	0.99473	-0.41351
C	-2.45576	-0.39679	-0.52838
H	-2.46642	2.97214	0.21459
H	-3.95955	1.16369	-0.66518
O	0.07167	2.60658	0.62053
O	-3.22683	-1.31456	-0.78248
N	-1.12923	-0.62113	-0.27184
N	-0.27739	0.41379	0.14933
C	-0.71901	-1.97650	0.13696
H	-1.37407	-2.65298	-0.40450
H	0.29980	-2.14870	-0.20492
C	1.12883	0.17715	-0.04813
C	1.98956	0.23591	1.03936
C	1.59773	-0.10132	-1.32773
C	3.34904	0.01502	0.83985
H	1.59557	0.45966	2.02318
C	2.95525	-0.33421	-1.51581
H	0.90049	-0.14390	-2.15692
C	3.83080	-0.27400	-0.43373
H	4.02891	0.06266	1.68154
H	3.32901	-0.55729	-2.50756
H	4.88851	-0.45298	-0.58403
C	-0.84439	-2.16147	1.64305
H	-0.54200	-3.17382	1.91447
H	-0.20442	-1.45692	2.17773
H	-1.87711	-2.00929	1.96275

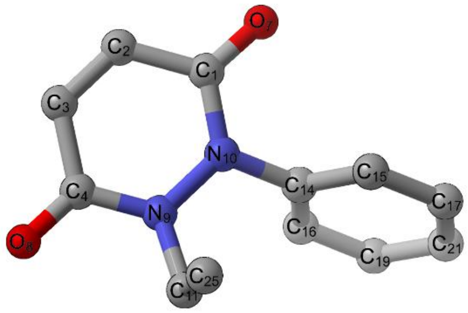

*Figure S10. Structure depiction of **3** generated using CYLview20.[17] Hydrogen atoms are omitted for clarity. The angle between the planes is defined by C_1-N_9-N_{10} and C_{14}-C_{15}-C_{16} was calculated to be 67.4° using their 3D coordinates.*

Dimethyl PD (S16a)
E = -493.324861
G = -493.215354

C	-0.02674	1.43492	-0.57002
C	-0.04177	0.66479	-1.82085
C	0.04177	-0.66479	-1.82085
C	0.02674	-1.43492	-0.57002
H	-0.07222	1.24957	-2.73136
H	0.07222	-1.24957	-2.73136
O	0.00576	2.66186	-0.56028
O	-0.00576	-2.66186	-0.56028
N	0.00576	-0.69930	0.58083
N	-0.00576	0.69930	0.58083
C	-0.34820	-1.34433	1.84365
H	-0.41876	-2.40744	1.64058
H	0.41517	-1.17098	2.59983
H	-1.31222	-0.96919	2.19072
C	0.34820	1.34433	1.84365
H	1.31222	0.96919	2.19072
H	0.41876	2.40744	1.64058
H	-0.41517	1.17098	2.59983

TS for addition to dimethyl PD (S16b)
E = -931.533727
G = -931.393746

C	0.38401	-1.40734	0.46378
C	-0.74696	-0.71795	1.13281
C	-0.51254	0.58325	1.58630
C	0.35119	1.43573	0.84392
H	-1.13434	1.04278	2.34287
O	0.52886	-2.63012	0.49346
O	0.40503	2.67450	0.92818
N	1.17989	0.80563	-0.09882

N	1.29582	-0.58892	-0.11827
C	1.39261	1.43532	-1.39794
H	1.10199	2.47825	-1.30449
H	2.44212	1.38720	-1.69271
H	0.78119	0.94155	-2.16071
C	2.57631	-1.09195	-0.59149
H	3.37573	-0.49099	-0.15501
H	2.66872	-2.12573	-0.27086
H	2.64268	-1.04725	-1.68059
H	-1.32291	-1.39332	1.75067
S	-2.17983	-0.84932	-0.64727
C	-2.33837	0.93078	-0.93585
H	-2.02132	1.45955	-0.02861
H	-1.71817	1.26635	-1.76820
H	-3.37704	1.19164	-1.13868

Enolate from addition to dimethyl PD (S16c)
E = -931.538986
G = -931.398275

C	-0.43880	0.96578	-0.61387
C	-0.83255	-0.46691	-0.87311
C	0.33279	-1.33465	-1.15182
C	1.53015	-1.13935	-0.50177
H	0.19092	-2.24133	-1.72609
O	-1.18085	1.91043	-0.89303
O	2.52400	-1.91413	-0.43463
N	1.69641	0.11764	0.21250
N	0.79331	1.15418	-0.09700
C	1.84295	-0.04455	1.66227
H	2.55923	-0.84532	1.83199
H	2.23590	0.87484	2.10001
H	0.88590	-0.29292	2.13779
C	1.30490	2.49925	0.11796
H	2.38697	2.46807	0.00153
H	0.86763	3.16812	-0.62062
H	1.05768	2.86860	1.11693
H	-1.56629	-0.45444	-1.68031
S	-1.78584	-1.07411	0.62625
C	-3.30273	-0.07487	0.53584
H	-3.08162	0.98066	0.68349
H	-3.78843	-0.21759	-0.43013
H	-3.96801	-0.42426	1.32505

Methyl phenyl PD (S17a)
E = -685.032397
G = -684.877029

C	-0.83578	1.59724	-0.02404
C	-2.28410	1.68735	-0.25687

C	-3.04199	0.59540	-0.34942
C	-2.50129	-0.74585	-0.07862
H	-2.67254	2.68553	-0.41438
H	-4.10015	0.63656	-0.57463
O	-0.09548	2.56749	-0.08896
O	-3.21089	-1.74609	-0.05650
N	-1.17141	-0.80296	0.21846
N	-0.34966	0.33175	0.20580
C	-0.62001	-1.99196	0.86905
H	-1.46171	-2.59327	1.20013
H	-0.00402	-2.57163	0.18186
H	-0.02208	-1.67935	1.72423
C	1.05817	0.08383	0.04612
C	1.94649	0.55258	1.00520
C	1.50190	-0.61587	-1.07077
C	3.30740	0.31922	0.83830
H	1.56928	1.09284	1.86464
C	2.86300	-0.85717	-1.22219
H	0.78514	-0.96812	-1.80440
C	3.76512	-0.38788	-0.27069
H	4.00842	0.68337	1.57919
H	3.21747	-1.40574	-2.08610
H	4.82514	-0.57428	-0.39313

TS for addition to methyl phenyl PD, beta to phenyl (S17b)
E = -1123.244146
G = -1123.057143

C	0.39083	-1.03828	-0.88142
C	1.78049	-0.68850	-1.25446
C	2.07445	0.66240	-1.42319
C	1.43439	1.62180	-0.58492
H	2.92850	0.99386	-1.99802
O	-0.15039	-2.07747	-1.23631
O	1.81277	2.78601	-0.38932
N	0.31554	1.16365	0.12888
N	-0.28503	-0.05942	-0.19368
C	0.14194	1.55601	1.52427
H	0.78875	2.41045	1.70513
H	-0.89220	1.84287	1.72370
H	0.41955	0.72573	2.18125
H	2.21347	-1.41432	-1.92884
S	2.70935	-1.63383	0.65247
C	3.46250	-0.11539	1.28773
H	3.49455	0.62344	0.47693
H	2.89306	0.30561	2.11740
H	4.48433	-0.30410	1.61705
C	-1.70459	-0.09214	-0.04479
C	-2.45844	1.02524	-0.40137
C	-2.32664	-1.22092	0.48507
C	-3.83916	1.00443	-0.23961

H	-1.95590	1.90196	-0.79238
C	-3.70989	-1.23578	0.63181
H	-1.72780	-2.07392	0.77427
C	-4.47059	-0.12666	0.27149
H	-4.42172	1.87484	-0.51704
H	-4.19200	-2.11564	1.04099
H	-5.54668	-0.14067	0.39508

Enolate from addition to methyl phenyl PD, beta to phenyl (S17c)
E = -1123.249825
G = -1123.061005

C	0.60346	-0.69501	-0.80251
C	2.03348	-0.22618	-0.87754
C	2.12364	1.24246	-1.02939
C	1.27153	2.06462	-0.32695
H	2.97134	1.67378	-1.54561
O	0.22995	-1.73753	-1.32790
O	1.35349	3.30806	-0.13572
N	0.12100	1.43313	0.29577
N	-0.25073	0.14736	-0.14611
C	0.08253	1.53172	1.75594
H	0.34287	2.55427	2.02007
H	-0.92905	1.32279	2.11028
H	0.78514	0.83314	2.22548
H	2.49863	-0.77240	-1.69836
S	2.91499	-0.82342	0.66633
C	2.83719	-2.62694	0.44454
H	1.80718	-2.97927	0.48033
H	3.28941	-2.90812	-0.50712
H	3.40433	-3.07947	1.25739
C	-1.65288	-0.11352	-0.08052
C	-2.55847	0.90087	-0.38835
C	-2.11546	-1.36267	0.33232
C	-3.92485	0.65656	-0.29989
H	-2.18160	1.87221	-0.68225
C	-3.48361	-1.59943	0.41084
H	-1.40465	-2.13744	0.58758
C	-4.39358	-0.59376	0.09473
H	-4.62447	1.44786	-0.54204
H	-3.83791	-2.57192	0.73174
H	-5.45845	-0.78065	0.16323

TS for addition to methyl phenyl PD, gamma to phenyl (S17d)
E = -1123.246588
G = -1123.058371

C	-1.97212	-1.11135	-0.35749
C	-2.49439	-0.04360	0.52314
C	-1.83405	0.19705	1.71302
C	-0.42058	-0.01195	1.80199

H	-2.29812	0.73563	2.52841
O	-2.68468	-1.75564	-1.12474
O	0.31719	0.42146	2.68868
N	0.15524	-0.71377	0.71658
N	-0.66007	-1.41648	-0.17697
H	-3.56718	0.07156	0.46259
S	-2.10120	1.65803	-1.14110
C	-0.91479	2.59465	-0.14345
H	-1.02158	2.29483	0.90719
H	0.11428	2.40110	-0.45187
H	-1.11232	3.66491	-0.21028
C	1.41222	-0.29862	0.17897
C	2.55778	-0.32186	0.97313
C	1.48921	0.11316	-1.15308
C	3.77326	0.09236	0.43562
H	2.48613	-0.65830	1.99767
C	2.71144	0.50765	-1.68400
H	0.58422	0.13727	-1.75145
C	3.85736	0.50386	-0.89143
H	4.66113	0.07814	1.05673
H	2.76549	0.82831	-2.71775
H	4.80837	0.81453	-1.30692
C	-0.08168	-2.63544	-0.72606
H	0.53605	-3.10046	0.04237
H	-0.89694	-3.29842	-1.00413
H	0.53133	-2.42777	-1.60575

Enolate from addition to methyl phenyl PD, gamma to phenyl (S17e)
E = -1123.254754
G = -1123.065669

C	1.91780	-0.24072	0.74654
C	2.03270	-0.15159	-0.76094
C	1.40602	-1.34510	-1.39066
C	0.22424	-1.84248	-0.89971
H	1.88080	-1.81754	-2.24007
O	2.85213	0.00798	1.50148
O	-0.49510	-2.78440	-1.31243
N	-0.25726	-1.12831	0.30623
N	0.72031	-0.69194	1.20217
H	3.09664	-0.07803	-0.98488
S	1.27882	1.43820	-1.36571
C	2.28564	2.65163	-0.46080
H	2.10746	2.58208	0.61203
H	3.34467	2.49616	-0.66944
H	1.99287	3.64099	-0.81057
C	-1.40507	-0.31301	0.22612
C	-2.40152	-0.57547	-0.72849
C	-1.59101	0.76119	1.10944
C	-3.53654	0.22362	-0.79236
H	-2.27439	-1.40510	-1.40975

C	-2.73749	1.54540	1.03545
H	-0.82672	1.00349	1.83600
C	-3.72228	1.28763	0.08756
H	-4.28793	0.00505	-1.54311
H	-2.85160	2.37278	1.72693
H	-4.61257	1.90192	0.03347
C	0.57315	-1.15915	2.57436
H	0.90869	-2.19582	2.66732
H	1.16623	-0.52298	3.22753
H	-0.47693	-1.09272	2.85574

Methyl 4-aminophenyl PD (S18a)
E = -740.393229
G = -740.223096

C	-1.29180	1.57475	0.03213
C	-2.74621	1.61624	-0.17075
C	-3.46279	0.49935	-0.29087
C	-2.86092	-0.83072	-0.11503
H	-3.17996	2.60345	-0.26772
H	-4.52852	0.50771	-0.48142
O	-0.59666	2.58254	0.02847
O	-3.53291	-1.85857	-0.14272
N	-1.51983	-0.84818	0.12768
N	-0.74892	0.32481	0.17977
C	-0.87400	-2.06625	0.61162
H	-1.65691	-2.80962	0.72255
H	-0.12575	-2.42074	-0.09627
H	-0.40406	-1.86871	1.57518
C	0.67567	0.16158	0.07877
C	1.47805	0.34383	1.19786
C	1.24480	-0.17264	-1.14555
C	2.85203	0.19054	1.09736
H	1.02195	0.60453	2.14594
C	2.61671	-0.33754	-1.24878
H	0.60879	-0.31215	-2.01299
C	3.44189	-0.15704	-0.12758
H	3.47828	0.33512	1.97034
H	3.06096	-0.60323	-2.20127
N	4.81823	-0.26167	-0.24338
H	5.32027	-0.44808	0.61284
H	5.15455	-0.81740	-1.01667

TS for addition to methyl 4-aminophenyl PD, beta to aryl (S18b)
E = -1178.603091
G = -1178.400572

C	0.74877	-0.96931	-0.95295
C	2.16408	-0.64355	-1.24826

C	2.49647	0.70938	-1.33630
C	1.83855	1.64705	-0.49062
H	3.38442	1.04526	-1.85476
O	0.19740	-1.98144	-1.37356
O	2.23149	2.79589	-0.23102
N	0.67089	1.19301	0.14345
N	0.07146	-0.01206	-0.24993
C	0.43582	1.53604	1.54274
H	1.07307	2.38355	1.78068
H	-0.60684	1.81442	1.70483
H	0.68446	0.68420	2.18417
H	2.60012	-1.33950	-1.95177
S	3.01106	-1.71226	0.60641
C	3.74016	-0.25022	1.38587
H	3.80922	0.54582	0.63378
H	3.13659	0.11111	2.21941
H	4.74604	-0.46985	1.74372
C	-1.35199	-0.05106	-0.11613
C	-2.12516	1.00619	-0.58714
C	-1.96872	-1.12873	0.51075
C	-3.50456	0.98389	-0.43919
H	-1.63858	1.85182	-1.05993
C	-3.34975	-1.16244	0.64416
H	-1.36234	-1.94406	0.88590
C	-4.13931	-0.10591	0.17165
H	-4.10001	1.81384	-0.80335
H	-3.82439	-2.00886	1.12831
N	-5.51955	-0.10621	0.36784
H	-5.93388	-1.02239	0.46932
H	-6.04176	0.46401	-0.28306

Enolate from addition to methyl 4-aminophenyl PD, beta to aryl (S18c)
E = -1178.610452
G = -1178.406836

C	2.22552	0.00977	0.78587
C	2.38848	0.02815	-0.71808
C	1.94548	-1.26435	-1.30116
C	0.81418	-1.88578	-0.82946
H	2.48691	-1.69425	-2.13327
O	3.07777	0.45437	1.55119
O	0.22216	-2.90768	-1.25811
N	0.21781	-1.24118	0.35724
N	1.09892	-0.59820	1.23064
H	3.44080	0.23419	-0.91269
S	1.45244	1.47112	-1.43537
C	2.23708	2.86054	-0.56375
H	2.01475	2.82545	0.50249
H	3.31688	2.84272	-0.71542
H	1.83101	3.77914	-0.98606
C	-1.05273	-0.60777	0.23428

C	-2.06750	-1.18662	-0.53858
C	-1.34201	0.58316	0.90794
C	-3.31216	-0.57869	-0.64694
H	-1.86938	-2.11424	-1.05654
C	-2.59614	1.17684	0.80903
H	-0.57788	1.06750	1.50278
C	-3.60407	0.60935	0.02866
H	-4.07688	-1.04431	-1.26062
H	-2.78849	2.10226	1.34235
C	0.92793	-0.93408	2.63634
H	1.32746	-1.93036	2.84388
H	1.45069	-0.19557	3.23931
H	-0.13503	-0.91413	2.87646
N	-4.84994	1.24464	-0.12570
H	-5.10748	1.82421	0.66248
H	-5.60363	0.60600	-0.34397

TS for addition to methyl 4-aminophenyl PD, gamma to phenyl (S18d)
E = -1178.605151
G = -1178.402938

C	-2.28611	-0.89104	-0.74052
C	-2.82881	-0.00852	0.31875
C	-2.27764	-0.11894	1.59065
C	-0.89864	-0.45021	1.74006
H	-2.79400	0.24277	2.46986
O	-2.95816	-1.27511	-1.69770
O	-0.21988	-0.29770	2.76169
N	-0.25890	-0.93850	0.57724
N	-1.01897	-1.32980	-0.53250
H	-3.89070	0.16257	0.21097
S	-2.25271	1.97012	-0.79652
C	-1.13348	2.60426	0.47645
H	-1.29428	2.03075	1.39884
H	-0.08737	2.49807	0.18471
H	-1.34298	3.65318	0.68705
C	1.06895	-0.50067	0.26149
C	2.15064	-0.91190	1.03414
C	1.28850	0.32710	-0.83869
C	3.43441	-0.47678	0.72901
H	1.97972	-1.56766	1.87766
C	2.57250	0.74360	-1.15889
H	0.44013	0.64464	-1.43816
C	3.66601	0.35279	-0.37456
H	4.27065	-0.79479	1.34231
H	2.73347	1.38535	-2.01857
C	-0.44370	-2.40571	-1.32626
H	0.00189	-3.13700	-0.65094
H	-1.24478	-2.86311	-1.90052
H	0.32287	-2.03035	-2.00762
N	4.94875	0.82839	-0.65402

H	5.10201	1.08553	-1.61945
H	5.69910	0.24592	-0.30842

Enolate from addition to methyl 4-aminophenyl PD, gamma to phenyl (S18e)
E = -1178.608554
G = -1178.404563

C	0.91152	-0.66458	-0.82022
C	2.36963	-0.28560	-0.87025
C	2.55892	1.17859	-0.96345
C	1.74552	2.03411	-0.25490
H	3.44638	1.57055	-1.44338
O	0.48198	-1.67932	-1.36286
O	1.90674	3.26400	-0.02458
N	0.53877	1.46612	0.32073
N	0.10363	0.21508	-0.16675
C	0.48543	1.51540	1.78345
H	0.79027	2.51400	2.08831
H	-0.53952	1.34050	2.11645
H	1.14798	0.76762	2.23586
H	2.80734	-0.82977	-1.70756
S	3.20054	-0.99627	0.65449
C	3.02119	-2.78251	0.36255
H	1.97240	-3.07536	0.37940
H	3.46285	-3.05217	-0.59740
H	3.55627	-3.29777	1.15971
C	-1.31242	0.02339	-0.11207
C	-2.16972	1.01825	-0.57311
C	-1.84580	-1.13896	0.43697
C	-3.54529	0.84922	-0.49698
H	-1.75042	1.93073	-0.97963
C	-3.22075	-1.31727	0.50294
H	-1.17782	-1.90657	0.80911
C	-4.09222	-0.32383	0.03887
H	-4.20603	1.62986	-0.85815
H	-3.62723	-2.22856	0.92801
N	-5.47336	-0.52250	0.05644
H	-5.79786	-1.17460	0.75723
H	-6.02228	0.32613	0.06400

4-Fluorophenyl methyl PD (S19a)
E = -784.273709
G = -784.128050

C	-1.25467	1.58700	-0.03104
C	-2.70052	1.62088	-0.29059
C	-3.41371	0.49998	-0.39125
C	-2.82732	-0.81945	-0.10755
H	-3.12453	2.60273	-0.45858
H	-4.46873	0.49957	-0.63436

O	-0.54946	2.58350	-0.08223
O	-3.49945	-1.84516	-0.09629
N	-1.50066	-0.82615	0.21003
N	-0.72487	0.34134	0.21213
C	-0.91064	-1.99309	0.86592
H	-1.73082	-2.64581	1.14970
H	-0.23457	-2.52529	0.19702
H	-0.36875	-1.66413	1.75200
C	0.69386	0.15295	0.08941
C	1.53419	0.62248	1.09095
C	1.20100	-0.48744	-1.03615
C	2.90770	0.45465	0.96810
H	1.11273	1.11629	1.95736
C	2.57183	-0.67402	-1.16037
H	0.52506	-0.84226	-1.80572
C	3.39251	-0.19409	-0.15430
H	3.59340	0.80932	1.72653
H	3.00205	-1.17298	-2.01896
F	4.72208	-0.36774	-0.27232

TS for addition to 4-fluorophenyl methyl PD, betato aryl (S19b)
E = -1222.485775
G = -1222.308609

C	0.72886	-1.01985	-0.90512
C	2.12792	-0.68102	-1.24885
C	2.44264	0.66695	-1.39177
C	1.79465	1.62559	-0.55782
H	3.31332	0.99502	-1.94313
O	0.18242	-2.05040	-1.27673
O	2.18293	2.78206	-0.34174
N	0.65225	1.17576	0.12553
N	0.05008	-0.04012	-0.22190
C	0.46848	1.54129	1.52778
H	1.10281	2.40117	1.72581
H	-0.57031	1.81126	1.72606
H	0.75384	0.70355	2.17190
H	2.56914	-1.40505	-1.91965
S	3.00296	-1.66483	0.67573
C	3.78491	-0.16927	1.32995
H	3.82161	0.58285	0.53130
H	3.22852	0.24657	2.17096
H	4.80652	-0.37768	1.64791
C	-1.37086	-0.06527	-0.09794
C	-2.11358	1.05400	-0.46973
C	-2.00719	-1.19040	0.42232
C	-3.49663	1.04813	-0.33618
H	-1.60321	1.92906	-0.85305
C	-3.39161	-1.20958	0.54509
H	-1.41968	-2.04625	0.72486
C	-4.10601	-0.08873	0.16297

H	-4.09529	1.90543	-0.61635
H	-3.91091	-2.07232	0.94247
F	-5.45025	-0.10112	0.29082

Enolate from addition to 4-fluorophenyl methyl PD, beta to aryl (S19c)
E = -1222.496209
G = -1222.316856

C	2.22858	0.01898	0.75208
C	2.33896	0.08363	-0.75614
C	1.91216	-1.20974	-1.35416
C	0.81335	-1.86773	-0.85983
H	2.45646	-1.62211	-2.19298
O	3.10520	0.43360	1.50338
O	0.24167	-2.91010	-1.26235
N	0.22307	-1.21863	0.33380
N	1.11190	-0.60046	1.21490
H	3.37852	0.32081	-0.98069
S	1.34003	1.51483	-1.40302
C	2.11900	2.89925	-0.51870
H	1.93806	2.82678	0.55352
H	3.19217	2.91704	-0.71139
H	1.67157	3.81774	-0.89699
C	-1.04460	-0.60219	0.23542
C	-2.01246	-1.10575	-0.64794
C	-1.37421	0.50252	1.03271
C	-3.26503	-0.51097	-0.73749
H	-1.77276	-1.96346	-1.26008
C	-2.63243	1.09065	0.95172
H	-0.63914	0.92685	1.70327
C	-3.55691	0.57481	0.06658
H	-4.01299	-0.89192	-1.42223
H	-2.88667	1.94733	1.56374
C	1.01174	-1.02531	2.60457
H	1.47008	-2.00858	2.74070
H	1.51746	-0.29482	3.23170
H	-0.04091	-1.07645	2.88118
F	-4.78454	1.14670	-0.01593

TS for addition to 4-fluorophenyl methyl PD, gamma to phenyl (S19d)
E = -1222.488450
G = -1222.309796

C	-2.29886	-0.87478	-0.70427
C	-2.82268	0.01928	0.35165
C	-2.26366	-0.06558	1.61277
C	-0.88434	-0.41729	1.75950
H	-2.76337	0.32328	2.48988
O	-2.98016	-1.26647	-1.64949
O	-0.20092	-0.25591	2.77172
N	-0.26107	-0.91906	0.59175

N	-1.03549	-1.33168	-0.49764
H	-3.87315	0.24483	0.23598
S	-2.14177	1.99499	-0.85556
C	-1.01423	2.59749	0.42723
H	-1.23178	2.07008	1.36539
H	0.02996	2.41497	0.16649
H	-1.15701	3.66466	0.59932
C	1.06303	-0.50015	0.25771
C	2.12907	-0.81085	1.10004
C	1.28500	0.20910	-0.92379
C	3.41480	-0.38969	0.77625
H	1.94655	-1.37390	2.00402
C	2.56945	0.61444	-1.26366
H	0.44165	0.45466	-1.56099
C	3.60701	0.30996	-0.40033
H	4.25916	-0.61328	1.41591
H	2.76618	1.16680	-2.17385
C	-0.49320	-2.44508	-1.26420
H	0.00650	-3.12709	-0.57604
H	-1.32114	-2.94883	-1.75629
H	0.22071	-2.10375	-2.01713
F	4.85780	0.70676	-0.72375

Enolate from addition to 4-fluorophenyl methyl PD, gamma to phenyl (S19e)
E = -1222.491666
G = -1222.312406

C	0.89827	-0.69253	-0.79934
C	2.35052	-0.29821	-0.87043
C	2.51886	1.16463	-1.01216
C	1.70675	2.02916	-0.31372
H	3.39186	1.55278	-1.52041
O	0.47224	-1.71711	-1.32002
O	1.85255	3.26592	-0.11860
N	0.51878	1.45857	0.30075
N	0.08597	0.19423	-0.14875
C	0.48704	1.54619	1.76249
H	0.79623	2.55275	2.03512
H	-0.53291	1.38224	2.11640
H	1.15627	0.81098	2.22457
H	2.78738	-0.86273	-1.69447
S	3.19811	-0.95170	0.66939
C	3.03522	-2.74735	0.43061
H	1.98961	-3.05054	0.46144
H	3.47562	-3.04076	-0.52289
H	3.57868	-3.23355	1.24020
C	-1.32736	0.00634	-0.09676
C	-2.17601	1.06131	-0.42753
C	-1.85826	-1.21285	0.32239
C	-3.55445	0.89742	-0.35682
H	-1.74901	2.00928	-0.72747

C	-3.23543	-1.38854	0.38616
H	-1.19366	-2.02133	0.59535
C	-4.05432	-0.32801	0.04373
H	-4.23288	1.70249	-0.60927
H	-3.67059	-2.32618	0.70795
F	-5.39367	-0.49263	0.11294

Methyl 4-nitrophenyl PD (S20a)
E = -889.524556
G = -889.370319

C	-1.92224	1.58205	-0.15270
C	-3.33890	1.50554	-0.53904
C	-3.97955	0.33996	-0.60810
C	-3.36394	-0.90608	-0.12126
H	-3.79137	2.44311	-0.83584
H	-5.00242	0.25469	-0.95223
O	-1.26978	2.60616	-0.24211
O	-3.98893	-1.95374	-0.02801
N	-2.07285	-0.80796	0.31526
N	-1.34360	0.38363	0.23310
C	-1.54700	-1.82452	1.23149
H	-2.39749	-2.34186	1.66807
H	-0.91403	-2.54355	0.71161
H	-0.97343	-1.32450	2.01040
C	0.07280	0.22918	0.13460
C	0.90511	1.05809	0.88318
C	0.59485	-0.74797	-0.71189
C	2.27975	0.91562	0.77850
H	0.47630	1.80137	1.54016
C	1.96794	-0.90368	-0.81162
H	-0.07159	-1.37422	-1.29209
C	2.78284	-0.06529	-0.06433
H	2.95122	1.54208	1.34857
H	2.40061	-1.65028	-1.46248
N	4.24224	-0.22473	-0.16867
O	4.66830	-1.11142	-0.87992
O	4.94438	0.53890	0.46166

TS for addition to methyl 4-nitrophenyl PD, beta to aryl (S20b)
E = -1327.739889
G = -1327.553827

C	1.32446	-1.07407	-0.87840
C	2.70882	-0.71228	-1.24456
C	3.00807	0.62874	-1.42516
C	2.35517	1.59862	-0.60286
H	3.87160	0.95280	-1.98952
O	0.79856	-2.12204	-1.21362
O	2.73346	2.75800	-0.40860

N	1.23026	1.14248	0.10971
N	0.61971	-0.06945	-0.23087
C	1.09824	1.48737	1.52495
H	1.73909	2.34499	1.71236
H	0.06791	1.75567	1.76577
H	1.40877	0.63870	2.14201
H	3.17769	-1.46042	-1.86820
S	3.54216	-1.60736	0.79016
C	4.38047	-0.09413	1.32236
H	4.41396	0.60470	0.47611
H	3.85867	0.39089	2.14844
H	5.40550	-0.30743	1.62601
C	-0.79030	-0.07552	-0.13875
C	-1.49366	1.11799	-0.34472
C	-1.47957	-1.24840	0.19347
C	-2.87341	1.13757	-0.24968
H	-0.94947	2.02222	-0.58290
C	-2.86109	-1.23319	0.28412
H	-0.93418	-2.15944	0.38388
C	-3.53858	-0.04242	0.05758
H	-3.42940	2.04934	-0.41798
H	-3.40826	-2.12895	0.54322
N	-4.99902	-0.02699	0.15805
O	-5.57341	1.03386	0.00437
O	-5.56921	-1.07598	0.38908

Enolate from addition to methyl 4-nitrophenyl PD, beta to aryl (S20c)
E = -1327.756608
G = -1327.569029

C	2.75950	0.29207	0.70667
C	2.82638	0.25622	-0.80621
C	2.56817	-1.13339	-1.28115
C	1.56454	-1.87237	-0.71057
H	3.17790	-1.55885	-2.06616
O	3.61209	0.82142	1.40494
O	1.13062	-3.01948	-0.95820
N	0.88679	-1.12746	0.39166
N	1.69910	-0.38028	1.24320
H	3.82050	0.60622	-1.08142
S	1.62580	1.47642	-1.52731
C	2.21009	3.01203	-0.74927
H	2.05465	2.98563	0.32921
H	3.26670	3.16964	-0.96760
H	1.63013	3.83029	-1.17445
C	-0.43043	-0.72999	0.28979
C	-1.29368	-1.34716	-0.64432
C	-0.94679	0.29046	1.11827
C	-2.61119	-0.95463	-0.74239
H	-0.91129	-2.13330	-1.27866
C	-2.26815	0.67548	1.02002

H	-0.29857	0.79788	1.81865
C	-3.09709	0.05034	0.09294
H	-3.26966	-1.42176	-1.46215
H	-2.65854	1.46326	1.64986
C	1.72001	-0.80530	2.63908
H	2.30416	-1.72234	2.75382
H	2.16141	-0.01094	3.23623
H	0.69738	-0.98355	2.96882
N	-4.48331	0.45580	-0.01034
O	-4.88992	1.33598	0.73139
O	-5.19201	-0.09837	-0.83537

TS for addition to methyl 4-nitrophenyl PD, gamma to phenyl (S20d)
E = -1327.743139
G = -1327.555698

C	2.86127	0.03768	1.06304
C	3.39075	0.00962	-0.31422
C	2.93329	-0.93689	-1.18405
C	1.60224	-1.47563	-1.03655
H	3.44421	-1.17393	-2.10760
O	3.49485	0.47049	2.01943
O	1.00623	-2.12592	-1.88457
N	0.93461	-1.08799	0.15234
N	1.66864	-0.60088	1.23472
H	4.36734	0.45761	-0.42792
S	2.18952	2.27435	-0.69398
C	1.19416	1.61639	-2.06177
H	1.63959	0.67817	-2.41812
H	0.16733	1.40864	-1.75170
H	1.17154	2.31183	-2.90179
C	-0.43008	-0.71972	0.13250
C	-1.38705	-1.55759	-0.45298
C	-0.81773	0.49262	0.71999
C	-2.71732	-1.17162	-0.47256
H	-1.08537	-2.50019	-0.88179
C	-2.14658	0.87399	0.71098
H	-0.06153	1.14569	1.13749
C	-3.07857	0.03551	0.11067
H	-3.47074	-1.80675	-0.91743
H	-2.45844	1.81414	1.14446
C	1.21405	-1.01201	2.55814
H	0.73125	-1.98434	2.46662
H	2.08468	-1.08791	3.20558
H	0.50987	-0.29597	2.98705
N	-4.48610	0.43623	0.09705
O	-5.28242	-0.28219	-0.47669
O	-4.79399	1.46985	0.65943

Enolate from addition to methyl 4-nitrophenyl PD, gamma to phenyl (S20e)
E = -1327.746368

G = -1327.558824

C	1.46285	-0.73356	-0.79075
C	2.92178	-0.36981	-0.85466
C	3.11562	1.08333	-1.05334
C	2.32775	1.98220	-0.37164
H	3.98521	1.43640	-1.59127
O	1.01550	-1.74486	-1.30776
O	2.49049	3.22023	-0.21246
N	1.14131	1.44173	0.27605
N	0.66011	0.18947	-0.15011
C	1.11369	1.56906	1.73445
H	1.49219	2.55940	1.97803
H	0.08525	1.48931	2.09386
H	1.73041	0.80293	2.21715
H	3.35656	-0.97254	-1.65192
S	3.73061	-0.98000	0.72112
C	3.53705	-2.77930	0.54042
H	2.48521	-3.06186	0.55671
H	3.99511	-3.11477	-0.39051
H	4.04996	-3.24653	1.38044
C	-0.74743	0.06193	-0.11431
C	-1.54236	1.20850	-0.23811
C	-1.35016	-1.18556	0.09787
C	-2.92144	1.11011	-0.18556
H	-1.06364	2.16823	-0.37341
C	-2.72923	-1.28795	0.15118
H	-0.74013	-2.06613	0.22754
C	-3.49696	-0.13973	0.00277
H	-3.54525	1.98659	-0.29323
H	-3.20678	-2.24304	0.32047
N	-4.95474	-0.24832	0.06098
O	-5.61073	0.77241	-0.02571
O	-5.44371	-1.35446	0.19217

Methyl pentafluorophenyl PD (S21a)
E = -1181.195543
G = -1181.085565

C	-1.67877	1.28118	-0.96981
C	-3.12884	1.20811	-1.18106
C	-3.86959	0.28260	-0.57283
C	-3.31199	-0.62798	0.44311
H	-3.53555	1.90791	-1.89966
H	-4.93220	0.17985	-0.75253
O	-0.93590	2.01329	-1.59616
O	-4.01175	-1.40961	1.07331
N	-1.97574	-0.50087	0.69149
N	-1.17555	0.39586	-0.03117
C	-1.37742	-1.09294	1.88482

H	-2.19658	-1.44379	2.50543
H	-0.73295	-1.93544	1.63015
H	-0.80814	-0.33107	2.41720
C	0.21946	0.15957	-0.01642
C	1.09364	1.13651	0.44346
C	0.74657	-1.03615	-0.49055
C	2.46148	0.92582	0.43296
C	2.11034	-1.26755	-0.48411
C	2.96761	-0.27933	-0.02707
F	4.27454	-0.48821	-0.02809
F	0.62271	2.28364	0.90826
F	3.28887	1.86548	0.87157
F	2.60032	-2.41754	-0.92754
F	-0.06970	-1.97790	-0.94808

TS for addition to methyl pentafluorophenyl PD, beta to aryl (S21b)
E = -1619.410233
G = -1619.268578

C	1.23329	-0.45185	-1.27630
C	2.62533	0.00467	-1.44928
C	2.96305	1.25333	-0.95095
C	2.32998	1.74378	0.23596
H	3.84486	1.78020	-1.28928
O	0.65148	-1.21223	-2.03261
O	2.75058	2.65753	0.95153
N	1.16664	1.06604	0.64929
N	0.56916	0.15165	-0.22904
C	1.03040	0.69010	2.05705
H	1.61377	1.39770	2.64009
H	-0.01403	0.75226	2.36739
H	1.40010	-0.32857	2.21300
H	3.07840	-0.35637	-2.36166
S	3.44209	-1.80145	-0.12539
C	4.33130	-0.76468	1.06201
H	4.34228	0.26752	0.68686
H	3.85358	-0.76841	2.04256
H	5.36433	-1.09582	1.16982
C	-0.83134	0.04035	-0.12384
C	-1.65775	1.14200	-0.32243
C	-1.42460	-1.17455	0.19918
C	-3.03148	1.04091	-0.18807
C	-2.79928	-1.29438	0.31135
C	-3.60263	-0.18227	0.12324
F	-4.91934	-0.28742	0.24154
F	-3.80637	2.10337	-0.37911
F	-3.35002	-2.46418	0.61796
F	-0.67034	-2.24352	0.42216
F	-1.13409	2.31440	-0.66068

Enolate from addition to methyl pentafluorophenyl PD, beta to aryl (S21c)

E = -1619.417395
G = -1619.276037

C	-2.51273	0.41190	1.03583
C	-3.06034	0.69366	-0.34240
C	-2.24604	1.65337	-1.11976
C	-0.88080	1.72832	-0.98431
H	-2.69823	2.15867	-1.96328
O	-3.24404	0.06198	1.96064
O	-0.02249	2.26549	-1.72366
N	-0.33348	1.09710	0.21721
N	-1.18503	0.61118	1.22256
H	-4.08844	1.02913	-0.19339
S	-3.23633	-0.93425	-1.25480
C	-4.55400	-1.75096	-0.30561
H	-4.21511	-1.99667	0.69894
H	-5.43101	-1.10482	-0.24984
H	-4.81653	-2.66409	-0.83935
C	0.88002	0.39360	0.08596
C	2.08533	1.09469	0.05335
C	0.95503	-0.99715	0.02195
C	3.30006	0.44361	-0.08048
C	2.16598	-1.66157	-0.07812
C	3.34333	-0.93848	-0.14369
C	-0.59285	0.56932	2.55195
H	0.02395	1.45911	2.68520
H	-1.39717	0.55493	3.28201
H	0.02526	-0.32303	2.68205
F	4.50892	-1.56634	-0.25566
F	-0.15631	-1.72793	0.06141
F	2.10688	2.41361	0.20201
F	4.43437	1.14017	-0.11335
F	2.19973	-2.99114	-0.13530

TS for addition to methyl pentafluorophenyl PD, gamma to aryl (S21d)
E = -1619.411035
G = -1619.268115

C	2.66947	-0.56507	1.20880
C	3.28590	-0.82852	-0.10780
C	2.55898	-1.46596	-1.08214
C	1.12702	-1.38635	-1.08835
H	3.02257	-1.89027	-1.96230
O	3.32803	-0.41667	2.23377
O	0.38178	-1.67030	-2.02349
N	0.55893	-0.86366	0.08618
N	1.30509	-0.60631	1.24555
H	4.35972	-0.93322	-0.06394
S	3.54529	1.61233	-0.57214
C	2.41044	1.58811	-1.98541
H	2.27530	0.55079	-2.32000

H	1.43189	1.99234	-1.72216
H	2.81569	2.15610	-2.82365
C	-0.74932	-0.34481	0.05364
C	-1.84047	-1.19015	-0.12676
C	-0.99961	1.01534	0.21313
C	-3.13162	-0.69412	-0.17947
C	-2.28860	1.51940	0.19092
C	-3.35631	0.66265	-0.01642
C	0.58131	-0.73752	2.50316
H	1.32115	-0.82833	3.29298
H	-0.04356	0.13693	2.69598
H	-0.04136	-1.63347	2.46901
F	-4.59324	1.13947	-0.05132
F	-2.50412	2.82098	0.34957
F	0.01018	1.85740	0.39995
F	-1.66358	-2.50096	-0.21938
F	-4.15988	-1.51695	-0.35836

Enolate from addition to methyl pentafluorophenyl PD, gamma to aryl (S21e)
E = -1619.417591
G = -1619.275042

C	1.37675	-0.36647	-0.96765
C	2.82723	0.02858	-0.95336
C	3.01028	1.45774	-0.60755
C	2.22395	2.07261	0.34060
H	3.88392	1.97745	-0.97836
O	0.90249	-1.19388	-1.73067
O	2.39297	3.18175	0.91005
N	1.02828	1.35347	0.77471
N	0.59133	0.31139	-0.07092
C	1.07492	0.91540	2.17476
H	1.39564	1.76859	2.76875
H	0.07468	0.61988	2.49883
H	1.76852	0.07859	2.31422
H	3.23807	-0.23279	-1.92902
S	3.69661	-1.10242	0.26268
C	3.52313	-2.71482	-0.55990
H	2.47803	-3.01726	-0.60691
H	3.94070	-2.66981	-1.56621
H	4.08548	-3.43891	0.02881
C	-0.80103	0.09611	-0.06090
C	-1.68795	1.09219	-0.45617
C	-1.32800	-1.11722	0.36507
C	-3.05668	0.89113	-0.41175
C	-2.69419	-1.33873	0.39565
C	-3.55955	-0.32877	0.01081
F	-4.87077	-0.52876	0.04587
F	-3.17953	-2.50633	0.80605
F	-0.51353	-2.08754	0.76648
F	-1.22995	2.25519	-0.90350

F	-3.89121	1.85295	-0.79402

6. SCXRD experiment

The angles between the least-square planes defining the PD and phenyl ring planes in **3** were calculated using the MPLA command in *SHELXL* for each molecule in the asymmetric unit:

Plane 1 (P1): N1-N2-C1-C2-C3-C4 (rms deviation of fitted atoms = 0.0109 Å)
Plane 2 (P2): C5-C6-C7-C8-C9-C10 (rms deviation of fitted atoms = 0.0058 Å)

ϕ(P1,P2)=74.15(3)°

Plane 2 (P3): N3-N4-C13-C14-C15-C16 (rms deviation of fitted atoms = 0.0151 Å)
Plane 3 (P4): C17-C18-C19-C20-C21-C22 (rms deviation of fitted atoms = 0.0079 Å)

ϕ(P3,P4)=87.79(3)°

*Table S9. Crystallographic and refinement parameters of **3***

	**3**
empirical formula	$C_{12}H_{12}N_2O_2$
M_r / g mol^{-1}	216.24
crystal system	monoclinic
space group	$P2_1/c$
a / Å	12.96490(10)
b / Å	8.25850(10)
c / Å	20.8574(2)
α / °	90
β / °	106.9280(10)
γ / °	90
V / Å^3	2136.45(4)
Z	8
ρ_{calc} / g cm^{-3}	1.345
T / K	150.0(1)
μ / mm^{-1}	0.763
F(000)	912
crystal size / mm^3	0.18 × 0.12 × 0.08
radiation	CuK_α (λ = 1.54184 Å)
2ϑ range for data collection / °	7.128 – 133.202
index ranges	$-15 \le h \le 15$<br>$-9 \le k \le 9$<br>$-24 \le l \le 24$
number of collected reflections	49635
unique reflections	3775
number of unique reflections	3373 [$I > 2\sigma(I)$]
R_{int}	0.0286
$R(F)$, $F > 2\sigma$(F)	0.0334
$wR(F^2)$, $F > 2\sigma$(F)	0.0372
$R(F)$, all data	0.0909
$wR(F^2)$, all data	0.0936
Δ_r (max., min.) e Å^{-3}	0.123/−0.223
CCDC deposition number	2296018

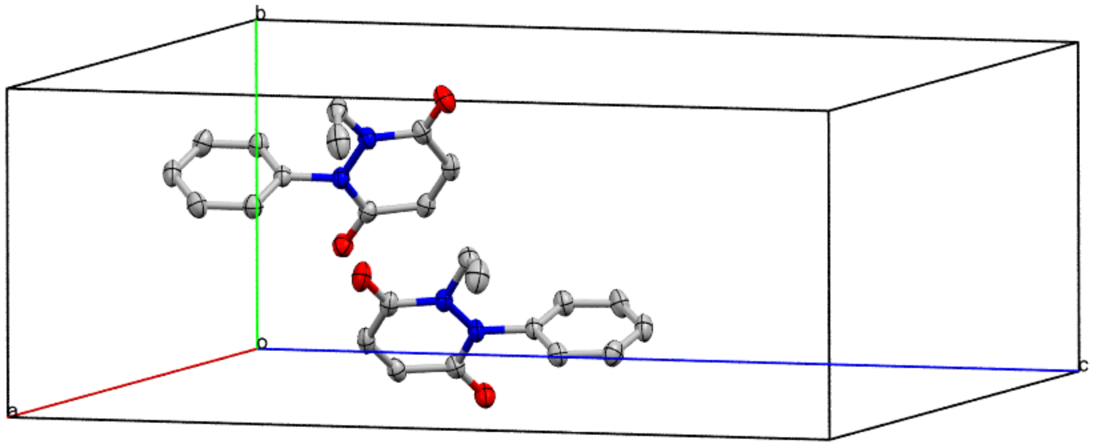

*Figure S11. The asymmetric unit of compound **3**. The thermal ellipsoids are drawn at the 50% probability level. Colour scheme: carbon - dark grey, nitrogen - blue, oxygen - red. The hydrogen atoms are omitted to enhance the clarity of the figure.*

7. References

1. R. K. Harris, E. D. Becker, S. M. De Menezes, P. Granger, R. E. Hoffman and K. W. Zilm, *Magn. Reson. Chem.*, 2008, **46**, 582-598.
2. Gaussian 16, Revision C.01, M. J. Frisch, G. W. Trucks, H. B. Schlegel, G. E. Scuseria, M. A. Robb, J. R. Cheeseman, G. Scalmani, V. Barone, G. A. Petersson, H. Nakatsuji, X. Li, M. Caricato, A. V. Marenich, J. Bloino, B. G. Janesko, R. Gomperts, B. Mennucci, H. P. Hratchian, J. V. Ortiz, A. F. Izmaylov, J. L. Sonnenberg, Williams, F. Ding, F. Lipparini, F. Egidi, J. Goings, B. Peng, A. Petrone, T. Henderson, D. Ranasinghe, V. G. Zakrzewski, J. Gao, N. Rega, G. Zheng, W. Liang, M. Hada, M. Ehara, K. Toyota, R. Fukuda, J. Hasegawa, M. Ishida, T. Nakajima, Y. Honda, O. Kitao, H. Nakai, T. Vreven, K. Throssell, J. A. Montgomery Jr., J. E. Peralta, F. Ogliaro, M. J. Bearpark, J. J. Heyd, E. N. Brothers, K. N. Kudin, V. N. Staroverov, T. A. Keith, R. Kobayashi, J. Normand, K. Raghavachari, A. P. Rendell, J. C. Burant, S. S. Iyengar, J. Tomasi, M. Cossi, J. M. Millam, M. Klene, C. Adamo, R. Cammi, J. W. Ochterski, R. L. Martin, K. Morokuma, O. Farkas, J. B. Foresman and D. J. Fox, Gaussian, Inc., Wallingford CT, 2019.
3. E. H. Krenske, R. C. Petter and K. N. Houk, *J. Org. Chem.*, 2016, **81**, 11726-11733.
4. K. Fukui, *Acc. Chem. Res.*, 1981, **14**, 363-368.
5. *CrysAllisPro, Agilent Technologies Inc.*, 2022.
6. G. M. Sheldrick, *Acta Crystallogr. A Found. Adv.*, 2015, **71**, 3-8.
7. O. V. Dolomanov, L. J. Bourhis, R. J. Gildea, J. A. K. Howard and H. Puschmann, *J. Appl. Crystallogr.*, 2009, **42**, 339-341.
8. G. M. Sheldrick, *Acta Crystallogr. C*, 2015, **71**, 3-8.
9. C. B. Hübschle, G. M. Sheldrick and B. Dittrich, *J. Appl. Crystallogr.*, 2011, **44**, 1281-1284.
10. V. Chudasama, M. E. Smith, F. F. Schumacher, D. Papaioannou, G. Waksman, J. R. Baker and S. Caddick, *Chem. Commun.*, 2011, **47**, 8781-8783.
11. C. Bahou, D. A. Richards, A. Maruani, E. A. Love, F. Javaid, S. Caddick, J. R. Baker and V. Chudasama, *Org. Biomol. Chem.*, 2018, **16**, 1359-1366.
12. C. Bahou, R. J. Spears, A. E. Aliev, A. Maruani, M. Fernandez, F. Javaid, P. A. Szijj, J. R. Baker and V. Chudasama, *Chem. Commun.*, 2019, **55**, 14829-14832.
13. P. Moody, M. E. Smith, C. P. Ryan, V. Chudasama, J. R. Baker, J. Molloy and S. Caddick, *Chembiochem*, 2012, **13**, 39-41.
14. M. K. Greene, D. A. Richards, J. C. F. Nogueira, K. Campbell, P. Smyth, M. Fernandez, C. J. Scott and V. Chudasama, *Chem. Sci.*, 2018, **9**, 79-87.
15. J. Dommerholt, S. Schmidt, R. Temming, L. J. Hendriks, F. P. Rutjes, J. C. van Hest, D. J. Lefeber, P. Friedl and F. L. van Delft, *Angew. Chem. Int. Ed. Engl.*, 2010, **49**, 9422-9425.
16. J. Meinecke and U. Koert, *Org. Lett.*, 2019, **21**, 7609-7612.
17. C. Y. Legault, CYLview20, Université de Sherbrooke, 2020, http://www.cylview.org.
